# Cationic Peptidomimetic Amphiphiles Having a *N*-Aryl- or *N*-Naphthyl-1,2,3-Triazole Core Structure Targeting *Clostridioides* (*Clostridium*) *difficile*: Synthesis, Antibacterial Evaluation, and an In Vivo *C. difficile* Infection Model

**DOI:** 10.3390/antibiotics10080913

**Published:** 2021-07-26

**Authors:** Muni Kumar Mahadari, Sreenu Jennepalli, Andrew J. Tague, Papanin Putsathit, Melanie L. Hutton, Katherine A. Hammer, Daniel R. Knight, Thomas V. Riley, Dena Lyras, Paul A. Keller, Stephen G. Pyne

**Affiliations:** 1 School of Chemistry and Biomolecular Science, University of Wollongong, Wollongong, NSW 2522, Australia; mkm933@uowmail.edu.au (M.K.M.); jennepal@ohsu.edu (S.J.); atague@uow.edu.au (A.J.T.); 2 School of Medical and Health Sciences, Edith Cowan University, Perth, WA 6027, Australia; papanin.putsathit@uwa.edu.au (P.P.); thomas.riley@uwa.edu.au (T.V.R.); 3 Infection and Immunity Program, Monash Biomedicine Discovery Institute and Department of Microbiology, Monash University, Clayton, VIC 3800, Australia; melanie.hutton@monash.edu (M.L.H.); dena.lyras@monash.edu (D.L.); 4 School of Biomedical Sciences, The University of Western Australia, Perth, WA 6009, Australia; katherine.hammer@uwa.edu.au (K.A.H.); Daniel.Knight@murdoch.edu.au (D.R.K.); 5 Biosecurity and One Health Research Centre, Harry Butler Institute, Murdoch University, Perth, WA 6150, Australia; 6 PathWest Laboratory Medicine, Queen Elizabeth II Medical Centre, Perth, WA 6009, Australia

**Keywords:** antibacterial, *Clostridioides* (*Clostridium*) *difficile*, peptidomimetic, triazole

## Abstract

*Clostridioides* (also known as *Clostridium*) *difficile* is a Gram-positive anaerobic, spore producing bacterial pathogen that causes severe gastrointestinal infection in humans. The current chemotherapeutic options are inadequate, expensive, and limited, and thus inexpensive drug treatments for *C. difficile* infection (CDI) with improved efficacy and specificity are urgently needed. To improve the solubility of our cationic amphiphilic 1,1′-binaphthylpeptidomimetics developed earlier that showed promise in an in vivo murine CDI model we have synthesized related compounds with an *N*-arytriazole or *N*-naphthyltriazole moiety instead of the 1,1′-biphenyl or 1,1′-binaphthyl moiety. This modification was made to increase the polarity and thus water solubility of the overall peptidomimetics, while maintaining the aromatic character. The dicationic *N*-naphthyltriazole derivative **40** was identified as a *C. difficile*-selective antibacterial with MIC values of 8 µg/mL against *C. difficile* strains ATCC 700057 and 132 (both ribotype 027). This compound displayed increased water solubility and reduced hemolytic activity (32 µg/mL) in an in vitro hemolysis assay and reduced cytotoxicity (CC_50_ 32 µg/mL against HEK293 cells) relative to lead compound **2**. Compound **40** exhibited mild efficacy (with 80% survival observed after 24 h compared to the DMSO control of 40%) in an in vivo murine model of *C. difficile* infection by reducing the severity and slowing the onset of disease.

## 1. Introduction

*Clostridioides* (also known as *Clostridium*) *difficile* is a Gram-positive, anaerobic spore-forming bacterium that causes mild to serious infections in the gastrointestinal tract (GIT) due to the production of potent exotoxins (TcdA, TcdB, and CDT) that cause severe gastrointestinal damage [1,2,3]. The resilient endospores contaminate healthcare environments and facilitate disease initiation, dissemination, and re-infection. In the GIT, spores require glycine and cholate derivatives for germination. In a healthy GIT, the microbiota metabolizes cholate derivatives preventing germination of *C. difficile* spores. CDI occurs when the normal GIT microbiota is disrupted or killed by conventional broad-spectrum antimicrobials [1]. Under these conditions the metabolism of cholate is significantly compromised, facilitating the germination of spores into *C. difficile* vegetative cells [4,5].

CDI has a mortality rate of up to 8% [2] with the reoccurrence of infections occurring in up to 20% of cases treated with vancomycin or metronidazole [6]. A 2019 Antibiotic Resistance Threat Report from the US Centers for Disease Control and Prevention indicated that in the USA in 2017 an estimated 223,900 cases of CDI in hospitalized patients resulted in 12,800 deaths and $1 billion in attributed healthcare costs [7]. Thus, there is a significant and important incentive to develop novel therapeutics that show selectivity for *C. difficile* over other gut bacteria to effectively combat CDI. While fecal microbiota transplantation can be effective for recurrent CDI, there can be adverse effects and the long-term impacts are unknown [1,2,8].

Fidaxomicin was specifically approved by the FDA in 2011 for treating CDI [9]; resulting in approximately 50% less CDI recurrence compared to vancomycin [10] most likely due to its greater selectivity for *C. difficile,* less impact on commensal enteric microflora (i.e., *Bacteroides* spp.), and its ability to reduce *C. difficile* sporulation [11]. There are many potential chemotherapeutics undergoing clinical trials for the treatment of CDI [12]. Other small molecule chemotherapeutics currently under investigation for use against *C. difficile*, include antimicrobial peptidomimetics [13,14,15], glycopeptides [16], bis-indoles [17], purine derivatives [18], tetramic acids [19], nitroheterocycles [20], macrolides [21], and nylon-3 polymers [22]. Two vaccines are being investigated in clinical trials (Pfizer and Intercell [23]), whereas bezlotoxumab (a monoclonal antibody targeting *C. difficile* TcdB) was given FDA approval in 2016 as adjunctive therapy for patients undergoing antimicrobial treatment who were at high risk of recurrent infection [24].

**Figure 1:**
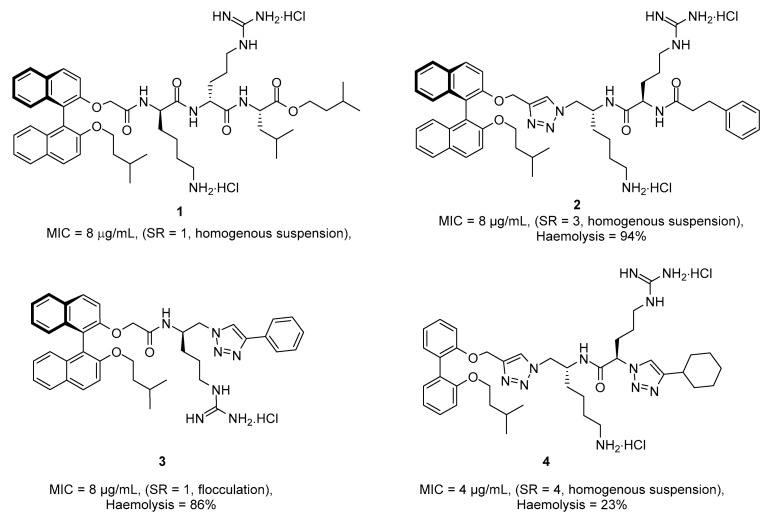
Previously published cationic amphiphilic hydrophobic anchored peptidomimetic antimicrobial agents. MIC values against *C. difficile* in µg/mL. SR = solubility ratio relative to that of compound **1**–see Ref [13].

**Figure 2:**
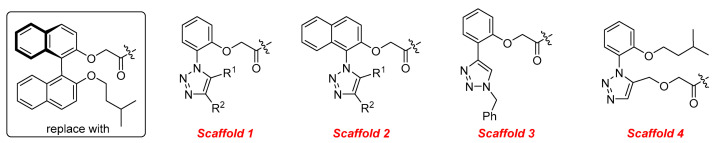
Hydrophobic scaffold replacements of the binaphthyl moiety for the target peptidomimetics.

In our earlier work on the development of the cationic amphiphilic 1,1′-binaphthylpeptidomimetics, we established the pharmacophoric importance of a hydrophobic head group (e.g., a binaphthyl moiety) connected to a dicationic peptide in the development of broad-spectrum antibacterial agents. This led to the identification of compound **1** with potent antibacterial activity against drug resistant Gram-positive bacteria with potential for topical applications (Figure 1) [25]. More recent work in our laboratory has identified compounds **2**–**4** from a class of small molecule cationic amphiphilic 1,1′-biarylpeptidomimetics that exert antibacterial activity through cytoplasmic membrane disruption [13,14]. These compounds have IC_50_ values of 4–8 μg/mL against *C. difficile* (Figure 1). The efficacy of these compounds at treating CDI in an in vivo murine CDI model was assessed against vancomycin as a positive control with 10% DMSO as the negative control. Compound **2** appeared to protect the mice from disease at the 24 h point with a 50% survival rate (2/4 mice) vs. 0% survival in the 10% DMSO group; this was not statistically significant due to the small sample size. These results clearly showed that compound **2** exhibited a notable positive effect in the treatment of CDI. Unfortunately compound **3** showed poor solubility with precipitation during preparation in a 10% DMSO solution, and high in vitro hemolytic activity against HEK293 cells. While compound **4** showed promising in vitro properties, it performed poorly in the *C. difficile* murine model with a survival rate of 60% after 24 h, but a 0% rate after 48 h [13], despite its low hemolytic activity. Despite some positive results, more water-soluble derivatives with lower hemolytic activity for further in vivo murine CDI model studies needed to be developed. To achieve this aim, we replaced the hydrophobic binaphthyl group found in **2** and **3** with an *N*-arytriazole or *N*-naphthyltriazole moiety as shown in Figure 2. These modifications should retain the aromatic character of these molecules while inducing a better polarity profile and thereby increasing the water solubility of the overall peptidomimetics. It was not clear at the start what effect these modifications would have on the antibacterial activities of these newly proposed compounds or their specificity for *C. difficile* over other pathogenic bacteria. Herein, we disclose the results of this investigation.

## 2. Results and Discussion

Preparation of the target *N*-arytriazole or *N*-naphthyltriazole peptidomimetics required the synthesis of the carboxylic acid derivatives **5**, **6**, **17**, **18**, **27**, and **28** based on scaffolds 1–4 (Figure 2); the syntheses of acid **17** is described in the experimental section with the other acid syntheses described in the Supporting Information.

**Scheme 1:**
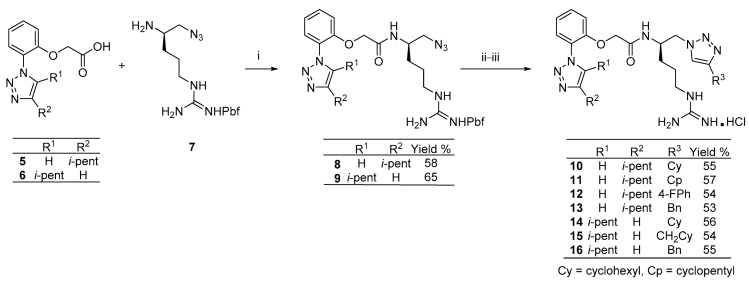
Synthesis of *N*-aryltriazole monocationic peptidomimetics **10**–**16**. i. HOBt (1.1 eq), EDCI.HCl (1.1 eq), Et_3_N (1.0 eq), CH_2_Cl_2_, rt, 16 h. ii. R^3^C≡CH, CuSO_4_.5H_2_O (0.2 eq), Na.ascorbate (0.4 eq), *t*-BuOH:H_2_O (4:1), rt, 16 h. iii. TFA/H_2_O/DCM, rt, 16 h; then HCl in Et_2_O.

**Scheme 2:**
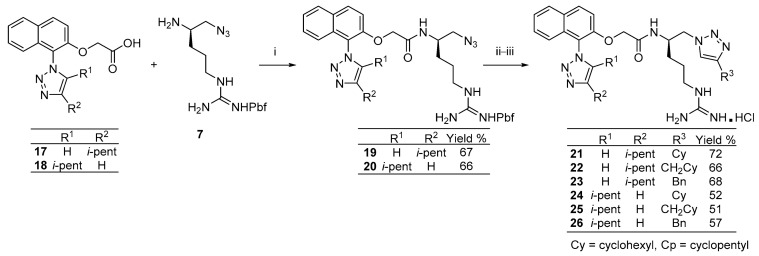
Synthesis of *N*-naphthyltriazole monocationic peptidomimetics **21**–**26**. i. HOBt (1.1 eq), EDC.HCl (1.1 eq), Et_3_N (1.0 eq), CH_2_Cl_2_, rt, 16 h. ii. R^3^C≡CH, CuSO_4_.5H_2_O (0.2 eq), Na.ascorbate (0.4 eq), *t*-BuOH:H_2_O (4:1), rt, 16 h. iii. TFA/H_2_O/DCM, rt, 16 h; then HCl in Et_2_O.

**Scheme 3:**
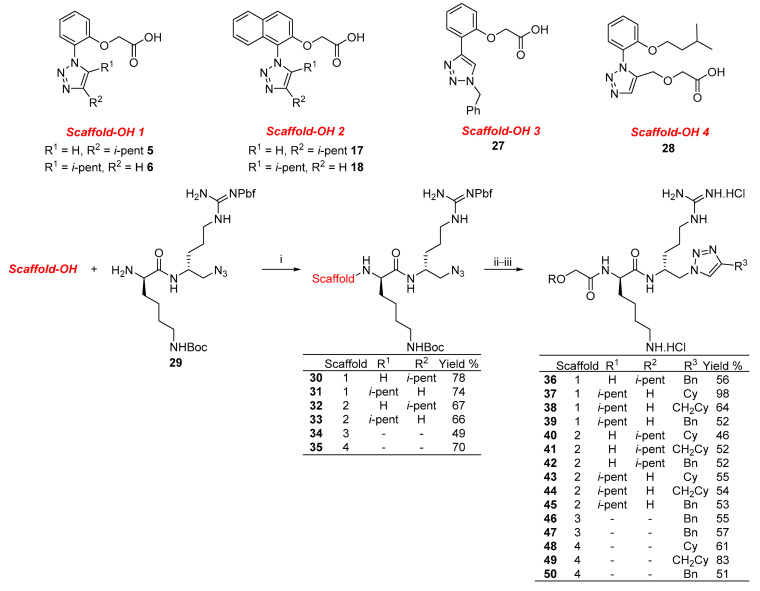
Synthesis of dicationic peptidomimetics **36**–**50**-i. HOBt (1.1 eq), EDC.HCl (1.1 eq), Et_3_N (1.0 eq), CH_2_Cl_2_, rt, 16 h. ii. R′C≡CH, CuSO_4_.5H_2_O (0.2 eq), Na.ascorbate (0.4 eq), *t*-BuOH:H_2_O (4:1), rt, 16 h. iii. TFA/H_2_O/DCM, rt, 16 h; then HCl in Et_2_O.

The synthesis of the new peptidomimetic derivatives is described in Scheme 1, Scheme 2 and Scheme 3. In a typical example, derivative **40** (Scheme 3) was generated starting from acid **17** coupling with the protected azidodipetide **29** under standard peptide coupling conditions (EDCI/HOBt) [26,27] to give amide **32** in 67% yield. This was followed by a standard copper-catalyzed azide-alkyne cycloaddition reactions [28] with ethenylcyclohexane to give the corresponding 1,4-disubstituted 1,2,3-triazole product which was deprotected using TFA/CH_2_Cl_2_/H_2_O followed by treatment with ethereal HCl to yield the dicationic amphiphile **40** in 46% yield over two steps. The synthesis of the additional mono- and dicationic peptidomimetic amphiphiles **10**–**16**, **21**–**26**, and **36**–**50** followed an analogous strategy and is summarized in Scheme 1, Scheme 2 and Scheme 3 with experimental and characterization details provided in the Supporting Information.

The *N*-arytriazole and *N*-naphthyltriazole peptidomimetics were subjected to antimicrobial screening. In the first instance, minimum inhibitory concentrations (MICs) were determined against a panel of Gram-positive (including two strains of *C. difficile*) and Gram-negative pathogenic bacteria with vancomycin and the commercially available peptide colistin as positive controls, respectively; the MICs are displayed in Table 1. The compounds were then tested against a second panel of Gram-positive and Gram-negative pathogenic bacteria and two fungi strains at the Community for Open Antimicrobial Drug Discovery (CO-ADD)-these results are reported in the Supporting Information (Table S1) [29]. A cytotoxicity concentration (CC_50_) assay was also performed by CO-ADD; the synthesized compounds were tested at concentrations ≤32 µg/mL on human embryonic kidney cells (HEK293 cells; ATCC CRL-1573) while hemolysis assays for lysis of human erythrocytes were also performed. Vancomycin, colistin, fluconazole, and tamoxifen were used as positive controls (see Table 1 for details). The CC_50_ and HC_50_ values are also shown in Table 1.

Preliminary screening revealed that compared to the previously synthesized compounds **1**–**4**, the new *N*-naphthyltriazole dicationic derivatives **40** and **42** showed the best activities against the two *C. difficile* RT 027 strains, ATCC 700,057 and 132 with a similar activity of 8 µg/mL compared to compounds **1**, **3**, and **4**. However, they were generally less active against the other Gram-positive and Gram-negative bacteria (Table 1). The relative solubility ratios (relative to compound **1**) [13] for **40** and **42** were 5 and 4 with CLogP values of 4.46 and 4.39, respectively, when compared to **1** with a ClogP of 7.47. Therefore, despite the better solubility profiles of these compounds, they failed to show better activity against *C. difficile*. However, the increased solubility (enhanced polarity) of derivatives **40**–**42** could be a factor in the reduced activities against the other bacteria, when compared to compounds **1**–**4** (see Table 2). None of the other derivatives synthesized in this study showed appreciable activity against *C. difficile* with MIC values ranging from 32 to 128 µg/mL (Table 1). Importantly, the remaining anti-bacterial results were generally poor, however for these specific derivatives, these reduced activities could indicate reduced capacity to interfere with normal GIT microbiota (Table 2). Compounds **40** and **42** showed a slight reduction in cytotoxicity against HEK293 cells compared to compounds **2** and **4**. The hemolytic activity of these compounds was 32 µg/mL against human erythrocytes, 2-fold more than their IC_50_ values against *C. difficile*.

Analysis of the anti-bacterial activities against other bacterial species indicated that the monocationic naphthyltriazole derivatives **21**–**26** showed appreciable activity against *Staphylococcus aureus* (including an MRSA strain) with MIC values between 4 and 8 µg/mL (Table 1). Additionally, compound **21** had notable MIC values of 4 µg/mL against *Enterococcus faecalis* and *Streptococcus pneumoniae*. An overview of activity shown in Table 1 showed “pockets” of activities focused on the naphthyl-based derivatives (**21**–**26** and **40**–**45**, columns 1–4), with the monocationic examples (**21**–**26**) producing better outcomes against the Gram positive strains. The second screening results (Table S1, Supporting Information) were consistent with these results with analogous trends in activity against an additional *S. aureus* strain.

The secondary testing (Table S1, Supporting Information) also identified compounds **21**, **25**, and **40–46** as having activity against the fungal strain *Cryptococcus neoformans* var*. grubii* (ATCC208821) (MIC 4-8 µg/mL).

## 3. In Vivo Assay: Murine Model of CDI

**Figure 3:**
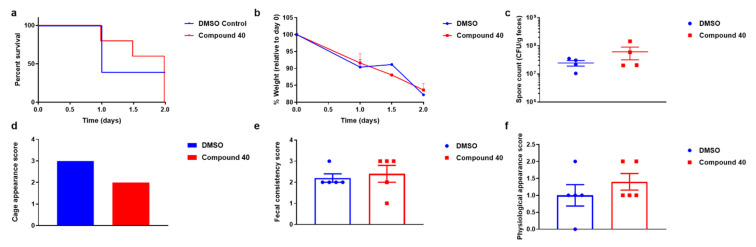
C57BL/6J mice (*n* = 5 per group) were infected with 10^5^ spores of *C. difficile* strain M7404 (RT027). Six hours post-infection and then every 12 h thereafter, mice were administered either 10% DMSO (blue) or 2.5 mg (100 mg/kg in 10% DMSO) of compound **40** (red) by oral gavage. Mice were monitored daily for survival (**a**) and weight loss relative to day 0, which was the day of infection (**b**). Fecal spore load at 1-day post-infection was determined by plating (**c**). Data are presented as CFU/gram feces, with each point representing a single mouse. Mouse cages were scored on day 1 post-infection for appearance (**d**) and mice were individually assessed for fecal consistency (**e**) and physiological appearance (**f**). Data represent the mean ±S.E.M. and statistical significance was assessed using a log-rank (Mantel–Cox) test or one-way ANOVA with a post hoc Tukey’s multiple comparison test.

Compound **40** was selected for further evaluation as an effective treatment for *C. difficile* using a murine model of CDI study because of its sustained antimicrobial potency against *C. difficile* and its better water solubility profile. The results from these studies are summarized in Figure 3.

The mice treated with compound **40** (red) showed delayed disease onset compared to mice treated with DMSO (blue; Figure 3), although they still succumbed to infection by day 2. Notably, at day 1 post-infection, mice treated with compound **40** showed 40% greater survival compared with mice treated with DMSO (Figure 3a), although there was no effect on mouse weight (Figure 3b), or spore numbers shed in the feces of these animals (Figure 3c), suggesting that compound **40** was not impacting *C. difficile* colonization. Furthermore, on day 1 post-infection, treatment with compound **40** resulted in a lower overall cage appearance score when compared to DMSO (Figure 3d), which suggested that this compound was delaying diarrheal onset although there was no significant difference in individual fecal score (Figure 3e) or physiological appearance score (Figure 3f) detected between the two groups of mice (Figure 3e). Thus, collectively these data suggest that compound **40** may reduce the severity of disease caused by *C. difficile.*

## 4. Materials and Methods

Synthetic methods and general characterization and analysis were as described previously [13].

***Notes and other considerations.*** Known reagents that were not available commercially were prepared as reported using known methods and is detailed in the Supporting Information, [14,32,33,34,35].

### 4.1. General Synthesis Procedures

#### 4.1.1. General Procedure I: Alkylation of Phenols (with Ethyl Bromoacetate)

A solution of the phenol (1 eq) in dry DMF (5 mL/mmol substrate) was stirred during the addition of K_2_CO_3_ (3 eq). Ethyl bromoacetate (1.3 eq) was added at room temperature and stirring was continued at rt for 12 h, before being diluted with EtOAc (2 × 50 mL). The resulting mixture was washed with water (2 × 50 mL), brine (2 × 50 mL), dried (MgSO_4_), filtered, and concentrated under vacuum. The residue was subjected to silica gel flash column chromatography to afford the desired ester product.

#### 4.1.2. General Procedure II: Ester Hydrolysis

A solution of the ester (1 eq) in ethanol (10 mL/mmol substrate) was stirred followed by the addition of 7% KOH solution (5 mL/mmol) at rt. The mixture was stirred at rt for 2 h, then acidified with 1 M HCl (25 mL). The resulting mixture was extracted with EtOAc (2 × 25 mL) and the combined extracts washed with brine (50 mL), dried (MgSO_4_), filtered, and concentrated under vacuum to afford the acid product.

#### 4.1.3. General Procedure III: Amide Coupling

A mixture of the amine (1.0 eq), carboxylic acid (1.0 eq), EDC.HCl (1.2 eq), HOBt (1.1 eq), and TEA (1 eq) in dichloromethane/acetonitrile solution (10 mL/mmol amine) was stirred at rt for the specified time. The mixture was concentrated (if >5.0 mL dichloromethane/acetonitrile), and then the resulting residue dissolved in EtOAc (25 mL for reactions that contained ≤1.0 mmol amine or 25 mL/mmol amine for larger scale reactions) and washed with aqueous HCl (1.0 M–2 × 25 mL), saturated aqueous NaHCO_3_ (3 × 25 mL), and brine (1 × 25 mL). The organic solution was dried (MgSO_4_), filtered, concentrated and subjected to further purification via flash chromatography (if required) to furnish the targeted amide product.

#### 4.1.4. General Procedure IV: Copper-Catalyzed Azide-Alkyne Cycloaddition

To a stirred solution of the azide (1.0 eq) and alkyne (2.0–3.0 eq) in *tert*-butanol/water (4:1) at rt was added CuSO_4_∙5H_2_O (0.2 eq), followed by sodium ascorbate (0.4 eq). The reaction was stirred at rt (unless noted otherwise) for the specified time. To the mixture was added aqueous saturated NH_4_Cl solution (1 mL), and water (20 mL) with the mixture then extracted with EtOAc (20 mL for reactions that contained ≤1.0 mmol azide or 20 mL/mmol azide for larger scale reactions). The organic layers were back-washed with water (2 × 25 mL), brine (2 × 25 mL), then dried (MgSO_4_), filtered, concentrated under vacuum and subjected to flash chromatography to afford the desired 1,4-disubstituted 1,2,3-triazole product.

#### 4.1.5. General Procedure VII: Amine Deprotection (*N*-Boc and/or *N*-Pbf Removal)

To a solution of the *N*-protected amine (1.0 eq) in CH_2_Cl_2_ (30 mL/mmol substrate) (if the substrate contained an *N*-Pbf moiety, H_2_O (20.0 eq) was added to the solution) was added TFA (30.0 mL/mmol substrate) and then stirred at rt overnight (>16 h). The solvent was removed and the resulting residue dissolved in CH_2_Cl_2_ (30 mL/mmol substrate). Excess anhydrous HCl (2.0 M in Et_2_O, 15 mL/mmol substrate, 30.0 eq) was added and the solvent was then removed. The residue was then dissolved in a minimal volume of CH_2_Cl_2_ (or MeOH) and excess Et_2_O (25 mL for ≤0.1 mmol substrate) was added, resulting in a precipitate of the hydrochloride salt of the amine. The reaction mixture was filtered; the resulting filtrate collected, concentrated, triturated with Et_2_O (3 × 20 mL); and the solids then dissolved in MeOH. The solution was concentrated and dried in vacuo to yield the mono or di-hydrochloride salt as a thin, translucent film that usually required scratching with a spatula, producing a fine hygroscopic powder or amorphous gum.

### 4.2. Representative Synthesis of Compound **40**

#### 4.2.1. Ethyl 2-((1-iodonaphthalen-2-yl)oxy)acetate


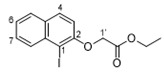


Following **General Procedure I**, 1-iodonaphthol (1.00 g, 3.70 mmol), K_2_CO_3_ (1.53 g, 11.11 mmol), and ethyl bromoacetate (0.80 g, 4.81 mmol) were stirred in DMF (8 mL) at rt for 16 h to give the titled ester (0.68 g, 52%) as a pale yellow waxy solid after flash chromatography over silica gel (EtOAc/*n*-hexane-10:90). TLC (EtOAc/*n*-hexane-20:80): *R*_f_ = 0.6; ^1^H NMR (400 MHz, CDCl_3_) δ 8.16 (d, *J* = 7.2 Hz, 1H, H8), 7.78 (d, *J* = 7.2 Hz, 1H, H5), 7.72 (d, *J* = 8.0 Hz, 1H, H4), 7.54 (t, *J* = 7.2 Hz, 1H, H7), 7.39 (t, *J* = 7.2 Hz, 1H, H6), 7.08 (d, *J* = 8.0 Hz, 1H, H3), 4.80 (s, 2H, H1′), 4.27 (q, *J* = 5.6 Hz, 2H, OCH_2_CH_3_), 1.29 (t, *J* = 5.6 Hz, 3H, OCH_2_CH_3_); ^13^C NMR (101 MHz, CDCl_3_) δ 168.7 (C = O), 155.6 (C2), 135.8 (C8a), 131.7 (C4a), 130.6 (C4), 130.5 (C8), 128.4 (C7), 128.3 (C5), 121.1 (C6), 114.4 (C3), 89.47 (C1), 67.6 (C1′), 61.7 (OCH_2_CH_3_) 14.3 (OCH_2_CH_3_); IR (neat) $\bar{\nu }$_max_ 2981, 1756, 1622, 1593, 1502, 1462, 1349, 1291, 1200, 1151, 1134, 1096, 1028, 801, 764, 747 cm^−1^; MS (ESI +ve) *m*/*z* 379 ([M + Na]^+^, 100%); HRMS (ESI + ve TOF) calcd for C_14_H_13_O_3_NaI 378.9807, found 378.9801 ([M + Na]^+^).

#### 4.2.2. Ethyl 2-((1-(4-isopentyl-1*H*-1,2,3-triazol-1-yl)naphthalen-2-yl)oxy)acetate


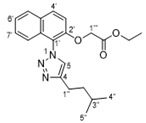


To a stirred solution of ethyl 2-(2-iodophenoxy)acetate (0.20 g, 0.54 mmol), 5-methyl-1-hexyne (0.16 g, 1.64 mmol), CuI (0.02 g, 0.11 mmol), NaN_3_ (0.04 g, 0.60 mmol), and sodium ascorbate (0.04 g, 0.22 mmol) in DMSO (2.5 mL) in H_2_O (0.5 mL) was added racemic *trans*-*N,N*′-dimethyl cyclohexane-1,2-diamine (0.016 g, 0.11 mmol) at rt under a nitrogen atmosphere. The reaction mixture was stirred and heated at 75 °C for 16 h. The reaction was cooled to rt and aqueous saturated NH_4_Cl solution (3 mL) was added, and the mixture was extracted with EtOAc (2 × 25 mL). The combined extracts were washed with water (25 mL), brine (25 mL) and dried (MgSO_4_). The solution was filtered, concentrated under vacuum and the residue was subjected to silica gel flash column chromatography (EtOAc/*n*-hexane-10:90 → 100:0) to afford the titled compound (0.05 g, 25%) as a yellow waxy solid. TLC (EtOAc/*n*-hexane-33:67); *R*_f_ = 0.4; ^1^H NMR (400 MHz, CDCl_3_) δ 7.97 (d, *J* = 7.2 Hz, 1H, H8′), 7.84 (d, *J* = 6.4 Hz, 1H, H5′), 7.67 (s, 1H, H5), 7.49–7.41 (m, 2H, H6′/H7′), 7.27–7.25 (m, 2H, H3′/H4′), 4.67 (s, 2H, H1′′′), 4.22 (q, *J* = 5.6 Hz, 2H, OCH_2_CH_3_), 2.89 (t, *J* = 5.6 Hz, 2H, H1′′), 1.73–1.67 (m, 3H, H2′′/H3′′), 1.26 (t, *J* = 5.6 Hz, 3H, OCH_2_OCH_3_), 0.99 (d, *J* = 4.0 Hz, 6H, H4′′/H5′′); ^13^C NMR (101 MHz, CDCl_3_) δ 168.5 (C = O), 150.5 (C2′), 148.1 (C8a′), 131.6 (C4), 131.3 (C4a′), 129.5 (C4′), 128.5 (C5′), 127.9 (C7′), 125.3 (C8′), 124.7 (C6′), 122.1 (C5), 121.3 (C3′), 114.3 (C1′), 66.7 (C1′′′), 61.6 (OCH_2_CH_3_), 38.6 (C2′′), 27.9 (C1′′), 23.8 (C3′′), 22.5 (C4′′/C5′′; Observed by gHMBC), 14.2 (OCH_2_CH_3_); IR (neat) $\bar{\nu }$_max_ 2954, 2928, 2868, 1748, 1632, 1600, 1513, 1483, 1454, 1430, 1366, 1288, 1206, 1150, 1117, 1087, 1042, 806, 749 cm^−1^; MS (ESI +ve) *m*/*z* 390 ([M +Na]^+^, 100%); HRMS (ESI +ve TOF) calcd for C_21_H_26_N_3_O_3_ 368.1974, found 368.1985 ([M + H]^+^).

#### 4.2.3. 2-((1-(4-Isopentyl-1*H*-1,2,3-triazol-1-yl)naphthalen-2-yl)oxy)acetic acid (**17**)


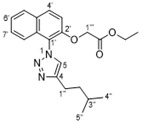


Following **General Procedure II**, ethyl 2-((1-(4-isopentyl-1*H*-1,2,3-triazol-1-yl)naphthalen-2-yl)oxy)acetate (0.07 g, 0.19 mmol) and 7% KOH solution (0.5 mL) were stirred in ethanol (2 mL) at rt for 2 h to give after acidification the acid **17** (0.04 g, 62%) as a white solid. M.P: 152–154 °C. TLC (EtOAc/*n*-hexane-100:0): *R*_f_ = 0.2; ^1^H NMR (500 MHz, CDCl_3_) δ 8.00 (d, *J* = 9.0 Hz, 1H, H8′), 7.88 (d, *J* = 7.5 Hz, 1H, H5′), 7.69 (s, 1H, H5), 7.54–7.46 (m, 2H, H6′/H7′), 7.47–7.29 (m, 2H, H3′/H4′), 4.78 (s, 2H, H1′′′), 2.91–2.87 (m, 2H, H1′′), 1.71–1.68 (m, 3H, H2′′/H3′′), 0.98 (d, *J* = 6.0 Hz, 6H, H4′′/H5′′), COOH resonance was not observed; ^13^C NMR (126 MHz, CDCl_3_) δ 170.6 (C = O), 150.4 (C2′), 148.4 (C8a′), 132.2 (C4), 130.7 (C4a′), 129.6 (C4′), 128.8 (C5′), 128.2 (C7′), 125.6 (C8′), 124.9 (C6′), 121.7 (C5), 120.9 (C3′), 114.4 (C1′), 66.8 (C1′′′), 38.5 (C2′′), 28.0 (C1′′), 23.7 (C3′′), 22.6 (C4′′/C5′′; Observed by gHMBC); IR (neat) $\bar{\nu }$_max_ 3147, 2954, 2929, 2868, 1731, 1631, 1600, 1514, 1483, 1429, 1366, 1284, 1213, 1151, 1118, 1087, 1062, 923, 806, 748 cm^−1^; MS (ESI +ve) *m*/*z* 362 ([M + Na]^+^, 40%), 340 ([M + H]^+^, 100%); HRMS (ESI + ve TOF) calcd for C_19_H_22_N_3_O_3_ 340.1661, found 340.1667 ([M + H]^+^).

#### 4.2.4. (9*H*-Fluoren-9-yl)methyl *tert*-butyl ((*R*)-6-(((*R*)-1-azido-5-(2-((2,2-dimethyl-2,3-dihydro benzofuran-5-yl)sulfonyl)guanidino)pentan-2-yl)amino)-6-oxohexane-1,5-diyl) dicarbamate


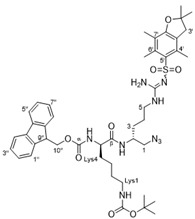


To a reaction vessel charged with azide **7** [30] (1.38 g, 3.16 mmol), Fmoc-L-Lys(Boc)-OH (1.62 g, 3.50 mmol), EDCI (0.67 g, 3.50 mmol) and HOBt (0.53 g, 3.50 mmol) was added CH_2_Cl_2_ (10 mL) and the mixture was stirred at rt for 12 h. The reaction mixture was concentrated and diluted with water (100 mL) and extracted with EtOAc (3 × 100 mL). The organic extracts were combined and washed with HCl (1 M–100 mL), aqueous NaHCO_3_ (100 mL), brine (25 mL), dried (MgSO_4_) and concentrated to give a pale-yellow residue. This residue was purified via flash chromatography over SiO_2_ (MeOH/CH_2_Cl_2_ = 4:96) to afford the titled compound as an off-white foam (1.50 g, 54%). TLC (MeOH/CH_2_Cl_2_–10:90) *R*_f_ = 0.52; ^1^H-NMR (400 MHz, CDCl_3_) δ 7.77–7.70 (m, 2H, H4′′/H5′′), 7.55 (d, *J* = 7.5 Hz, 2H, H1′′/H8′′), 7.55 (brs, 1H, βCONH), 7.41–7.32 (m, 2H, H2′′/H7′′), 7.29–7.21 (m, 2H, H3′′/H6′′), 7.17 (brs, 1H, αCONH), 6.31–6.24 (m, 2H, NH_2_ (guanidine)), 6.19–6.09 (brs, 1H, *N*^5^-H), 4.82–4.72 (brs, 1H, Lys*N*^1^-H), 4.33 (d, *J* = 7.4 Hz, 2H, H10′′), 4.25–4.07 (m, 2H, Lys5/H9′′), 4.07–3.97 (m, 1H, H2), 3.41–3.23 (m, 2H, H1), 3.23–2.98 (m, 4H, H5/Lys1), 2.89 (s, 2H, H3′), 2.55 (s, 3H, C6′-CH_3_), 2.48 (s, 3H, C4′-CH_3_), 2.06 (s, 3H, C7′-CH_3_), 1.67 (s, 6H, C2′-CH_3_), 1.55–1.35 (m, 19H, H3/H4/Lys2/Lys3/Lys4/C(CH_3_)_3_); ^13^C NMR (101 MHz, CDCl_3_) δ 172.7 (Cβ), 158.8 (C7a′), 156.7 (Cα), 156.4 (C = N), 156.2 (COOC(CH_3_)_3_), 143.85 (C1a′′or C8a′′), 143.83 (C8a′′ or C1a′′), 143.82 (C4a′′ or C5a′′), 143.6 (C5a′′ or C4a′′), 138.3 (C3a′), 132.8 (C6′), 132.2 (C4′), 127.8 (C3′′/C6′′), 127.1 (C4′′/C5′′), 125.0 (C2′′/C7′′), 124.7 (C5′), 120.0 (C1′′/C8′′), 117.6 (C7′), 86.4 (C2′), 79.3 (C(CH_3_)_3_), 67.3 (C10′′), 55.1 (Lys5), 54.8 (C1), 48.8 (C2), 47.0 (C9′′), 43.2 (C3′), 40.9 (C5), 39.9 (Lys1), 31.9 (Lys2), 29.5 (Lys4), 29.3 (C3), 28.6 (C2′-(CH_3_)_2_), 28.4 (C(CH_3_)_3_), 25.5 (C4), 22.5 (Lys3), 19.3 (C6′-CH_3_), 17.9 (C4′-CH_3_), 12.5 C7′-CH_3_); IR (neat) $\bar{\nu }$_max_ 3322, 2101, 1634, 1548, 1450, 1248, 1165, 1092, 739, 567 cm^−1^; MS (ESI +ve) *m*/*z* 888 ([M + H]^+^), 910 ([M + Na]^+^); HRMS (ESI +ve TOF) calcd for C_45_H_61_N_9_O_8_SNa 910.4262, found 910.4218 ([M + Na]^+^).

#### 4.2.5. *Tert*-butyl ((*R*)-5-amino-6-(((*R*)-1-azido-5-(2-((2,2-dimethyl-2,3-dihydrobenzofuran-5-yl)sulfonyl)guanidino)pentan-2-yl)amino)-6-oxohexyl)carbamate (**31**)


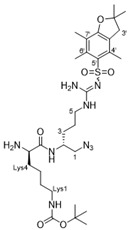


To a solution of the above Fmoc-protected amine (1.50 g, 1.69 mmol) in acetonitrile (15 mL) was added piperidine (0.25 mL, 1.5 eq.) and the reaction was stirred vigorously at rt for 12 h. The reaction mixture was diluted with MeOH (50 mL) and extracted with hexane (50 mL) multiple times until TLC analysis showed no byproduct (dibenzofulvene piperidine adduct) present in the MeOH layer. The MeOH extract was concentrated under reduced pressure to give **31** as an off-white foam (0.80 g, 71%). TLC (MeOH/CH_2_Cl_2_–10:90) *R*_f_ = 0.2; ^1^H-NMR (500 MHz, CDCl_3_) δ 7.61 (brs, 1H, N^2^-H), 6.42–6.20 (m, 3H, N^5^-H/NH_2_ (guanidine)), 4.82–4.72 (m, 1H, LysN^1^-H), 4.12–3.99 (m, 1H, Lys5), 3.46–3.29 (m, 3H, H1/H2), 3.29–3.14 (m, 2H, H5), 3.14–3.04 (m, 2H, Lys1), 2.96 (s, 2H, C3′), 2.58 (s, 3H, C6′-CH_3_), 2.52 (s, 3H, C4′-CH_3_), 2.10 (s, 3H, C7′-CH_3_), 1.62–1.31 (m, 25H, H3/H4/Lys2/Lys3/Lys4/C(CH_3_)_3_/C2′-(CH_3_)_2_), *N*^5^H_2_ resonance was not observed; ^13^C-NMR (126 MHz, CDCl_3_) δ 158.8 (C7a′), 156.6 (C = O), 156.4 (C = N), 138.5 (C3a′), 133.2 (C4′), 132.4 (C6′), 124.7 (C5′), 117.6 (C7′), 86.5 (C2′), 79.4 ((C(CH_3_)_3_), 55.1 (Lys5), 55.0 (C1), 46.9 (C2), 43.4 (C3′), 40.9 (C5), 40.4 (Lys1), 34.7 (Lys4), 30.1 (Lys2), 29.8 (C3), 28.8 (C2′-(CH_3_)_2_), 28.6 (C(CH_3_)_3_), 25.8 (C4), 22.7 (Lys3), 19.4 (C6′-CH_3_), 18.1 (C4′-CH_3_), 12.6 (C7′-CH_3_), COO(C(CH_3_)_3_) resonance was not observed; IR (neat) $\bar{\nu }$_max_ 3327, 2101, 1685, 1620, 1551, 1454, 1366, 1278, 1250, 1168, 1094, 665, 569 cm^−1;^ MS (ESI +ve) *m*/*z* 666 ([M + H]^+^); HRMS (ESI +ve TOF) calcd for C_30_H_52_N_9_O_6_S 666.3761, found 666.3741 ([M + H]^+^).

#### 4.2.6. *Tert*-butyl ((*R*)-6-(((*R*)-1-azido-5-(2-((2,2,4,6,7-pentamethyl-2,3-dihydrobenzofuran-5-yl)sulfonyl)guanidino)pentan-2-yl)amino)-5-(2-((1-(4-isopentyl-1*H*-1,2,3-triazol-1-yl)naphthalen-2-yl)oxy)acetamido)-6-oxohexyl)carbamate (**32**)


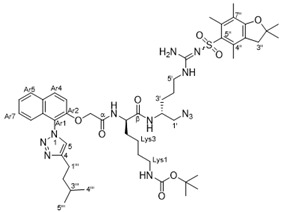


Following **General Procedure III**, 2-((1-(4-isopentyl-1*H*-1,2,3-triazol-1-yl)naphthalen-2-yl)oxy)acetic acid **17** (0.12 g, 0.35 mmol), *tert*-butyl ((*R*)-5-amino-6-(((*R*)-1-azido-5-(2-((2,2-dimethyl-2,3-dihydrobenzofuran-5-yl)sulfonyl)guanidino)pentan-2-yl)amino)-6-oxohexyl) carbamate **57** (0.24 g, 0.35 mmol), EDCI.HCl (0.08 g, 0.39 mmol), HOBt (0.06 g, 0.39 mmol), and TEA (0.03 g, 0.35 mmol) were stirred in CH_2_Cl_2_ (5 mL) at rt for 12 h to give the acetamide **65** (0.22 g, 64%) as an off-white solid. M.P: 236–238 °C. TLC (MeOH/CH_2_Cl_2_-10:90): *R*_f_ = 0.5; ^1^H NMR (400 MHz, CDCl_3_) δ 8.05 (d, *J* = 8.5 Hz, 1H, Ar8), 7.89 (d, *J* = 8.5 Hz, 1H, Ar5), 7.65 (s, 1H, H5), 7.54–7.47 (m, 2H, Ar4/βCONH), 7.37 (d, *J* = 8.5 Hz, 1H, Ar7), 7.26–7.19 (m, 2H, Ar6/Ar3), 6.85 (brs, 1H, αCONH), 6.36–6.08 (m, 3H, N^5′^-H/NH_2_ (guanidine)), 5.00 (brs, 1H, LysN^1^-H), 4.69 (ABq, *J* = 16.5 Hz, 2H, OCH_A_H_B_), 4.42–4.36 (m, 1H, Lys5), 4.02–3.96 (m, 1H, H2′), 3.44–2.94 (m, 6H, H1′/H5′/Lys1), 2.88 (s, 2H, H3′′), 2.88–2.84 (m, 2H, H1′′′), 2.55 (s, 3H, C4′′-CH_3_), 2.48 (s, 3H, C6′′-CH_3_), 2.06 (s, 3H, C7′′-CH_3_), 2.00–1.86 (m, 4H, H4′/Lys4), 1.84–1.60 (m, 7H, H3′/Lys3/H2′′′/H3′′′), 1.44 (s, 6H, C2′′(CH_3_)_2_), 1.39 (s, 9H, C(CH_3_)_3_), 1.32–1.22 (m, 2H, Lys2), 0.9 (d, *J* = 5.0 Hz, 6H, H4′′′/H5′′′); ^13^C NMR (101 MHz, CDCl_3_) δ 171.9 (βC = O), 168.1 (αC = O), 158.7 (C7a′′), 156.4 (C = N), 150.1 (Ar2), 149.1 (COOC(CH_3_)_3_), 138.4 (Ar8a), 133.4 (C4), 132.6 (C4′′), 132.46 (C6′′), 132.44 (C3a′′), 130.6 (C5′′), 129.4 (Ar4), 129.1 (Ar4a), 128.48 (C7′′), 128.47 (Ar5), 125.7 (Ar7), 124.7 (Ar8), 121.1 (C5), 120.3 (Ar6), 117.5 (Ar3), 113.8 (Ar1), 86.5 (C2′′), 79.2 (C(CH_3_)_3_), 68.0 (OCH_A_H_B_), 54.8 (Lys5), 53.6 (C1′), 43.4 (C2′), 40.8 (C2′′′), 40.2 (C5′), 38.6 (C3′′), 38.5 (Lys1), 31.79 (Lys4), 31.74 (C3′), 29.4 (Lys2), 28.7 (C2′′-(CH_3_)_2_), 28.6 ((CH_3_)_3_), 28.0 (C1′′′), 25.5 (C4′), 23.8 (C3′′′), 22.8 (C4′′′/C5′′′), 22.6 (Lys3), 19.4 (C4′′-CH_3_), 18.1 (C6′′-CH_3_), 12.6 (C7′′-CH_3_); IR (neat) $\bar{\nu }$_max_ 3405, 3317, 3415, 3057, 2953, 2868, 2100, 1664, 1631, 1600, 1546, 1514, 1484, 1452, 1406, 1390, 1366, 1265, 1247, 1165, 1106, 1090, 1044, 994, 970, 852, 781, 733, 661, 641 cm^−1^; MS (ESI +ve) *m*/*z* 987 ([M + H]^+^, 100%); HRMS (ESI +ve TOF) calcd for C_49_H_71_N_12_O_8_S 987.5239, found 987.5272 ([M + H]^+^).

#### 4.2.7. (*R*)-6-Amino-*N*-((*R*)-1-(4-cyclohexyl-1*H*-1,2,3-triazol-1-yl)-5-guanidinopentan-2-yl)-2-(2-((1-(4-isopentyl-1*H*-1,2,3-triazol-1-yl)naphthalen-2-yl)oxy)acetamido)hexanamide dihydrochloride (**40**)


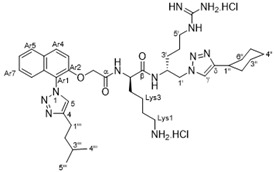


Following **General Procedure IV**, azide **32** (0.08 g, 0.08mmol), cyclohexylacetylene (0.03 g, 0.24 mmol), CuSO_4_∙5H_2_O (0.004 g, 0.01 mmol) and sodium ascorbate (0.006 g, 0.03 mmol) were stirred in *t*-BuOH (2.0 mL) and H_2_O (0.5 mL) for 16 h to give the triazole product as an off-white gum after flash chromatography over SiO_2_ gel (MeOH/CH_2_Cl_2_–0:100 → 8:92). Following **General Procedure VII**, the intermediate (0.06 g, 0.05 mmol) was dissolved in CH_2_Cl_2_ (2 mL), treated with H_2_O (0.02 g, 1.00 mmol) and CF_3_COOH (1 mL) followed by work-up with ethereal HCl (3 mL) to give the amine salt **40** (0.03 g, 46% over two steps) as an off-white solid that rapidly transitioned to a sticky gum. ${\left[\alpha \right]}_{D}^{23}$ + 59.1 (*c* 0.0052, MeOH); ^1^H NMR (400 MHz, CD_3_OD) δ 8.30 (s, 1H, H5), 8.29 (s, 1H, Hγ), 8.18 (d, *J* = 9.2 Hz, 1H, Ar8), 7.98 (d, *J* = 7.5 Hz, 1H, Ar5), 7.61 (ddd, *J* = 9.2, 9.2, 1.7 Hz, 1H, Ar7), 7.57–7.49 (m, 2H, Ar6/Ar4), 7.14 (d, *J* = 8.3 Hz, 1H, Ar3), 4.93–4.89 (m, 2H, OCH_A_H_B_), 4.77–4.72 (m, 1H, H1′), 4.59–4.53 (m, 1H, H1′), 4.37–4.32 (m, 1H, Lys5), 4.12–4.09 (m, 1H, H2′), 3.18–3.14 (m, 2H, H5′), 2.95–2.91 (m, 2H, Lys1), 2.84–2.78 (m, 3H, H1′′′/H1′′), 2.00–1.96 (m, 2H, Lys4), 1.74–1.60 (m, 14H, H2′′′/H3′′′/Lys2/H3′/H4′/H2′′/H3′′/H4′′/H5′′/H6′′), 1.48–1.21 (m, 7H, Lys3/H2′′/H3′′/H4′′/H5′′/H6′′), 1.01 (d, *J* = 6.2 Hz, 6H, H4′′′/H5′′′); ^13^C NMR (101 MHz, CD_3_OD) δ 173.0 (βC = O), 169.1 (αC = O), 157.1 (C = N), 150.8 (Ar2), 148.7 (C4), 147.6 (Cδ), 132.5 (Ar8a), 130.3 (Ar4), 129.1 (Ar4a), 128.6 (Ar5), 128.0 (Ar7), 126.9 (Ar8), 125.5 (C5), 125.1 (Cγ), 120.2 (Ar6), 119.1 (Ar3), 113.9 (Ar1), 67.4 (OCH_A_H_B_), 55.7 (C1′), 53.5 (Lys5), 49.3 (C2′), 40.4 (C5′), 39.0 (Lys1), 37.9 (C2′′′), 33.4 (C1′′), 31.6 (C2′′), 31.5 (C6′′), 30.9 (Lys4), 28.1 (Lys2), 27.5 (C1′′′), 26.5 (C3′), 25.2 (C4′′), 25.0 (C3′′/C5′′), 24.8 (C3′′′), 22.6 (C4′), 22.5 (C4′′′/C5′′′), 21.3 (Lys3); IR (neat) $\bar{\nu }$_max_ 3348, 3265, 3202, 3066, 2932, 2860, 1662, 1544, 1514, 1483, 1451, 1384, 1366, 1349, 1279, 1220, 1168, 1117, 1081, 1049, 816, 749, 668, 585 cm^−1^; MS (ESI + ve) *m*/*z* 743 ([M–2HCl + H]^+^, 60%), 372 ([M–2HCl + H]^2+^, 100%); HRMS (ESI + ve TOF) calcd for C_39_H_59_N_12_O_3_ 743.4833, found 743.4866 ([M–2HCl + H]^+^).

### 4.3. Microbiological Assays

**Primary screening (Gram-positive bacteria).** Primary MIC assays were performed as described by the Clinical and Laboratory Standards Institute for aerobic [36] and anaerobic [37] bacteria. MIC values for vancomycin were within acceptable QC ranges [38].

Secondary screening (MRSA and Gram-negative bacteria) and cytotoxicity assay–performed by the Community for Open Antimicrobial Drug Discovery (CO-ADD). Samples were provided to CO-ADD [29] for antimicrobial screening by whole cell growth inhibition assays.

**Bacterial Inhibition–MIC Assay.** These were performed as described previously [13,29].

**Cytotoxicity Assay.** These were performed as described previously [13,29].

**Haemolysis assay (sheep erythrocytes).** These were performed as described previously [13].

**Hemolysis assay (human erythrocytes)**–HC_50_ determination. These were performed as described previously [13,29].

### 4.4. In Vivo Murine Model of CDI Treatment

**Disease Treatment Model.** These experiments were performed as previously described [39,40,41,42]. Mice were humanely killed at the onset of severe disease or at the end of the experiment (day 4), as previously described [43].

**Statistical Analysis.** Statistical analysis was performed using Prism 7 (GraphPad Software). The Kaplan–Meier survival curves were assessed using a log-rank (Mantel–Cox) test. Weight loss, spore shedding, fecal consistency, and physiological appearance data were analyzed by one-way ANOVA with a post hoc Tukey’s multiple comparison test. Differences in data values were considered significant at a *p* value of <0.05.

## 5. Conclusions

This study reported the next generation of hydrophobic anchored cationic peptidomimetics as antibacterial agents, with a focus on targeting CDI. A major aim was to improve the solubility profile of these compounds to allow for sufficient solubility for efficient administration of the drug while maintaining gut availability and antibacterial activity. The naphthyltriazole derivates containing either a monocationic or dicationic amino acid side chain were generally the most effective, with compounds **40** and **42**, possessing terminal cyclohexyl and benzyl moieties, respectively, exhibiting MIC values of 8 µg/mL.

Naphthyltriazole **40** was selected for an in vivo murine model trials of CDI but exhibited only mild evidence of in vivo efficacy indicating that further investigation into the structural and biological parameters affecting the in vivo efficacy of these antibacterial peptidomimetics is required, as the observed in vitro efficacy did not translate directly into in vivo efficacy. We have already reported that a correlation exists between increased hemolytic activity and an increase in hydrophobic/cationic ratio [15]; unfortunately, compound **40** exhibited a slight increase in hemolytic activity relative to the majority of tested compounds in this class with an HC_50_ value of 32 µg/mL. While the selectivity ratio could be more substantial, this is acceptable for the future development of these gastrointestinal focused compounds. We have previously reported a comparative solubility assay for this class of antimicrobial agents with increasing numerical values corresponding to better aqueous solubility relative to compound **1** (which possesses a value of 1) [13]. Compound **40** showed a better solubility ratio with an assay value of 5, relative to our lead compound **2** with a value 3—this is also reflected in the CLogP values of 4.46 and 5.76 for **41** vs. **2**, respectively. These outcomes were confirmed with no issues during the mouse model trials with sufficient solubility in the dosage regimen. Variations on the triazole and *O*-naphthyl substituents could be made in future studies with the view of enhancing antibacterial activity against *C. difficile*.

## Figures and Tables

**Table 1:** Preliminary antibacterial screening ^a^.

	Compound	*C. difficile* ATCC 700057	*C. difficile* 132 ^b^ (RT027)	*S. aureus* ATCC 29213	*S. aureus* NCTC 10442 ^c^	*E. faecalis* ATCC 29212	*S. pneumoniae* ATCC 49619	*E. coli* ATCC 25922	CC_50_ ^d^	HC_50_ ^e^
1	**10**	32	64	32	32	32	16	128	>32	>32
2	**11**	32	64	32	32	32	16	128	>32	>32
3	**12**	32	64	32	32	32	16	128	>32	>32
4	**13**	32	64	32	32	32	16	128	>32	>32
5	**14**	128	128	32	32	32	32	>128	>32	>32
6	**15**	64	64	16	16	16	16	128	>32	>32
7	**16**	32	32	32	32	32	32	>128	>32	>32
8	**21**	128	>128	8	8	16	16	128	>32	>32
8	**22**	32	32	4	4	8	8	32	>32	>32
9	**23**	32	32	4	4	4	4	64	>32	>32
10	**24**	32	32	8	8	8	8	32	21.9	>32
11	**25**	32	64	4	4	8	8	16	>32	10.6
12	**26**	64	>128	8	8	16	4	64	23.5	>32
13	**36**	128	128	32	32	64	16	128	>32	>32
14	**37**	64	64	16	32	16	8	64	>32	>32
15	**38**	128	>128	128	128	>128	128	>128	32	32
16	**39**	64	32	32	64	32	16	128	>32	>32
17	**40**	8	8	16	16	32	16	64	32	32
18	**41**	16	16	8	8	8	16	128	16	32
19	**42**	8	8	8	8	8	8	32	32	32
20	**43**	128	128	16	16	64	4	64	>32	>32
21	**44**	32	32	8	4	16	4	64	>32	>32
22	**45**	128	128	16	16	64	4	128	>32	>32
23	**46**	128	128	32	32	64	16	128	>32	>32
24	**47**	128	128	32	32	64	16	128	>32	>32
25	**48**	>32	>32	>32	>32	>32	>32	>32	>32	24.5
26	**49**	>32	>32	32	>32	>32	>32	>32	>32	17.1
27	**50**	>32	>32	32	>32	>32	>32	>32	>32	19.8
	vanc	0.5	0.5	1	1	4	1	>16	-	-
	colistin				0.25	0.25	0.25	0.125		
	tamoxifen								13.1	

^a^ Values are reported as MIC values in μg/mL. ^b^ *C. difficile* PCR Ribotype (RT027). ^c^ Methicillin resistant *S. aureus* (MRSA). ^d^ Cytotoxicity; determined on HEK293 cells. ^e^ Hemolysis; HC50 values determined by lysis of human erythrocytes and % hemolysis was determined by lysis of sheep erythrocytes. Vanc = vancomycin. Coloured cells refer to the same activities.

**Table 2:** Antimicrobial, cytotoxicity, and hemolytic activities of the three most active derivatives synthesized in this study ^a^.

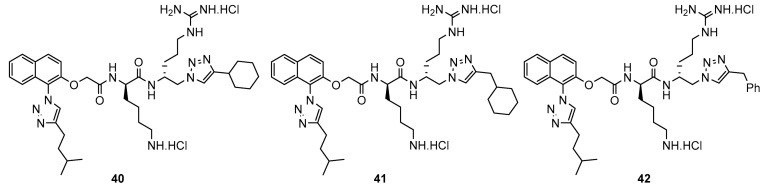										
Compound	*C. difficile* ATCC 700057	*C. difficile* 132 ^b^ (RT027)	*S. aureus* ATCC 29213	*S. aureus* ATCC 43300 ^c^	*S. aureus* NCTC 104422 ^c,d^	*E. faecalis* ATCC 29212	*S. pneumoniae* ATCC 49619	*E. coli* ATCC 25922	CC_50_ ^e^	HC_50_ ^f^
**40**	8	8	16	8	16	32	16	64	32	32
**41**	16	16	8	4	8	8	16	128	16	32
**42**	8	8	8	8	8	8	8	32	32	32
**1** [30]	8 ^g^	8 ^h^	2	-	-	2	2	16	-	-
**2** [31]	-	32	4	2	4	4	4	8	27.4	94% ^i^
**3** [19]	8 ^g^	8 ^h^	2	-	2	4	8	>128	-	-
**4** [31]	-	8	8	4	4	8	4	8	14.2	23% ^i^
vanc	0.5	0.5	1	-	1	4	1	>16	-	-

^a^ Values are reported as MIC values in μg/mL. ^b^ *C. difficile* PCR Ribotype (RT027). ^c^ Methicillin resistant *S. aureus* (MRSA). ^d^ Testing performed by the Community for Open Antimicrobial Drug Discovery (CO-ADD). ^e^ Cytotoxicity; determined on HEK293 cells. ^f^ Hemolysis; determined by lysis of sheep erythrocytes ^g^ *C. difficile strain* tested M7404 (RT027). ^h^ *C. difficile strain* tested R20291 (RT207). ^i^ % hemolysis at 50 µg/mL. vanc = vancomycin. Coloured cells refer to the same activities.

## Data Availability

The data presented in this study are available in supplementary material.

## Supplementary Materials

The following are available online at https://www.mdpi.com/article/10.3390/antibiotics10080913/s1, Figures S1–S85: Details of synthesis and characterization data for compounds; Table S1: Secondary antimicrobial screening ^a^–(bacteria and fungi), Murine model studies experimental procedures.

## References

[1] Leffler D.A., Lamont J.T. (2009). Treatment of *Clostridium difficile*-Associated Disease. Gastroenterology.

[2] Knight D.R., Elliott B., Chang B.J., Perkins T.T., Riley T.V. (2015). Diversity and Evolution in the Genome of *Clostridium difficile*. Clin. Microbiol. Rev..

[3] Eaton S.R., Mazuski J.E. (2013). Overview of Severe *Clostridium difficile* Infection. Crit. Care Clin..

[4] Di Bella S., Ascenzi P., Siarakas S., Petrosillo N., Di Masi A. (2016). *Clostridium difficile* Toxins A and B: Insights into Pathogenic Properties and Extraintestinal Effects. Toxins.

[5] Chandrasekaran R., Lacy D.B. (2017). The role of toxins in *Clostridium difficile* infection. FEMS Microbiol. Rev..

[6] Johnson A.P. (2010). New antibiotics for selective treatment of gastrointestinal infection caused by *Clostridium difficile*. Expert Opin. Ther. Pat..

[7] Centers for Disease Control and Prevention (2019). Clostridium Difficile Update. https://www.cdc.gov/drugresistance/pdf/threats-report/CRE-508.pdf.

[8] Stanley J.D., Bartlett J.G., Dart B.W., Ashcraft J. (2013). *Clostridium difficile* infection. Curr. Probl. Surg..

[9] Ritter A.S., Petri W.A. (2013). New developments in chemotherapeutic options for *Clostridium difficile* colitis. Curr. Opin. Infect. Dis..

[10] Cornely O.A., Miller M.A., Louie T.J., Crook D.W., Gorbach S.L. (2012). Treatment of First Recurrence of *Clostridium difficile* Infection: Fidaxomicin Versus Vancomycin. Clin. Infect. Dis..

[11] Hostler C.J., Chen L.F. (2013). Fidaxomicin for treatment of *Clostridium difficile*-associated diarrhea and its potential role for prophylaxis. Expert Opin. Pharmacother..

[12] Cho J.M., Pardi D.S., Khanna S. (2020). Update on Treatment of *Clostridioides difficile* Infection. Mayo Clin Proc..

[13] Tague A.J., Putsathit P., Hammer K.A., Wales S.M., Knight D.R., Riley T.V., Keller P.A., Pyne S.G. (2019). Cationic biaryl 1,2,3-triazolyl peptidomimetic amphiphiles targeting *Clostridioides* (*Clostridium*) *difficile*: Synthesis, antibacterial evaluation and an in vivo *C. difficile* infection model. Eur. J. Med. Chem..

[14] Wales S.M., Hammer K.A., King A.M., Tague A.J., Lyras D., Riley T.V., Keller P.A., Pyne S.G. (2015). Binaphthyl-1,2,3-triazole peptidomimetics with activity against *Clostridium difficile* and other pathogenic bacteria. Org. Biomol. Chem..

[15] Tague A.J., Putsathit P., Riley T.V., Keller P.A., Pyne S.G. (2021). Positional Isomers of Biphenyl Antimicrobial Peptidomimetic Amphiphiles. ACS Med. Chem. Lett..

[16] Zhang S.J., Yang Q., Xu L., Chang J., Sun X. (2012). Synthesis and antibacterial activity against *Clostridium difficile* of novel demethylvancomycin derivatives. Bioorg. Med. Chem. Lett..

[17] Butler M.M., Williams J.D., Peet N.P., Moir D.T., Panchal R.G., Bavari S., Shinabarger D.L., Bowlin T.L. (2010). Comparative In Vitro Activity Profiles of Novel Bis-Indole Antibacterials against Gram-Positive and Gram-Negative Clinical Isolates. Antimicrob. Agents Chemother..

[18] Dvoskin S., Xu W.-C., Brown N.C., Yanachkov I.B., Yanachkova M., Wright G.E. (2012). A Novel Agent Effective against *Clostridium difficile* Infection. Antimicrob. Agents Chemother..

[19] Ueda C., Tateda K., Horikawa M., Kimura S., Ishii Y., Nomura K., Yamada K., Suematsu T., Inoue Y., Ishiguro M. (2010). Anti-*Clostridium difficile* Potential of Tetramic Acid Derivatives from *Pseudomonas aeruginosa* Quorum-Sensing Autoinducers. Antimicrob. Agents Chemother..

[20] Ballard T.E., Wang X., Olekhnovich I., Koerner T., Seymour C., Hoffman P.S., Macdonald T.L. (2010). Biological Activity of Modified and Exchanged 2-Amino-5-Nitrothiazole Amide Analogues of Nitazoxanide. Bioorg. Med. Chem. Lett..

[21] Kirst H.A., Toth J.E., Debono M., Willard K.E., Truedell B.A., Ott J.L., Counter F.T., Felty-Duckworth A.M., Pekarek R.S. (1988). Synthesis and evaluation of tylosin-related macrolides modified at the aldehyde function: A new series of orally effective antibiotics. J. Med. Chem..

[22] Liu R., Suárez J.M., Weisblum B., Gellman S.H., McBride S.M. (2014). Synthetic Polymers Active against *Clostridium difficile* Vegetative Cell Growth and Spore Outgrowth. J. Am. Chem. Soc..

[23] Jarrad A.M., Karoli T., Blaskovich M.A.T., Lyras D., Cooper M.A. (2015). *Clostridium difficile* Drug Pipeline: Challenges in Discovery and Development of New Agents. J. Med. Chem..

[24] Lowes R. FDA Approves Zinplava for Preventing Return of *C. difficile*. https://www.medscape.com/viewarticle/870887.

[25] Bremner J.B., Keller P.A., Pyne S.G., Boyle T.P., Brkic Z., David D.M., Garas A., Morgan J., Robertson M., Somphol K. (2010). Binaphthyl-Based Dicationic Peptoids with Therapeutic Potential. Angew. Chem. Int. Ed..

[26] Bremner J.B., Keller P.A., Pyne S.G., Boyle T.P., Brkic Z., David D.M., Robertson M., Somphol K., Baylis D., Coates J.A. (2010). Synthesis and antibacterial studies of binaphthyl-based tripeptoids. Part 1. Bioorg. Med. Chem..

[27] Bremner J.B., Keller P.A., Pyne S.G., Boyle T.P., Brkic Z., Morgan J., Somphol K., Coates J.A., Deadman J., Rhodes D.I. (2010). Synthesis and antibacterial studies of binaphthyl-based tripeptoids. Part 2. Bioorg. Med. Chem..

[28] Mahadari M.K., Tague A.J., Keller P.A., Pyne S.G. (2021). Synthesis of sterically congested 1,5-disubstituted-1,2,3-Triazoles using chloromagnesium acetylides and hindered 1-naphthyl azides. Tetrahedron.

[29] Blaskovich M.A.T., Zuegg J., Elliott A.G., Cooper M.A. (2015). Helping chemists discover new antibiotics. ACS Infect. Dis..

[30] Wales S.M., Hammer K.A., Somphol K., Kemker I., Schröder D.C., Tague A.J., Brkic Z., King A.M., Lyras D., Riley T.V. (2019). Synthesis and antimicrobial activity of binaphthylbased, functionalized oxazole and thiazole peptidomimetics. Org. Biomol. Chem..

[31] Tague A.J., Putsathit P., Hammer K.A., Wales S.M., Knight D.R., Riley T.V., Keller P.A., Pyne S.G. (2019). Cationic biaryl 1,2,3-triazolyl peptidomimetic amphiphiles: Synthesis, antibacterial evaluation and preliminary mechanism of action studies. Eur. J. Med. Chem..

[32] Zhu D., Ma J., Luo K., Fu H., Zhang L., Zhu S. (2016). Enantioselective Intramolecular C-H Insertion of Donor and Donor/Donor Carbenes by a Nondiazo Approach. Angew. Chem. Int. Ed..

[33] Maehr H., Smallheer J. (1985). Total syntheses of rivularins D1 and D3. J. Am. Chem. Soc..

[34] Gamble A.B., Garner J., Gordon C.P., O’Conner S.M.J., Keller P.A. (2007). Aryl Nitro Reduction with Iron Powder or Stannous Chloride under Ultrasonic Irradiation. Synth. Commun..

[35] Zilla M.K., Nayak D., Vishwakarma R.A., Sharma P.R., Goswami A., Ali A. (2014). A convergent synthesis of alkyne-azide cycloaddition derivatives of 4-α,β-2-propyne podophyllotoxin depicting potent cytotoxic activity. Eur. J. Med. Chem..

[36] Clinical and Laboratory Standards Institute (2015). Methods for Dilution Antimicrobial Susceptibility Tests for Bacteria that Grow Aerobically.

[37] Clinical and Laboratory Standards Institute (2012). Methods for Antimicrobial Susceptibility Testing of Anaerobic Bacteria.

[38] Clinical Laboratory Standards Institute (2018). Performance Standards for Antimicrobial Susceptibility Testing.

[39] Carter G.P., Lyras D., Allen D.L., Mackin K.E., Howarth P.M., O’Connor J.R., Rood J.I. (2007). Binary toxin production in Clostridium difficile is regulated by CdtR, a LytTR family response regulator. J. Bacteriol..

[40] Hutton M.L., Cunningham B.A., Mackin K.E., Lyon S.A., James M.L., Rood J.I., Lyras D. (2017). Bovine antibodies targeting primary and recurrent *Clostridium difficile* disease are a potent antibiotic alternative. Sci. Rep..

[41] Lyon S.A., Hutton M.L., Rood J.I., Cheung J.K., Lyras D. (2016). CdtR regulates TcdA and TcdB production in *Clostridium difficile*. PLoS Pathog..

[42] Awad M.M., Hutton M.L., Quek A.J., Klare W.P., Mileto S.J., Mackin K., Ly D., Oorschot V., Bosnjak M., Jenkin G. (2020). Human Plasminogen Exacerbates *Clostridioides difficile* Enteric Disease and Alters the Spore Surface. Gastroenterology.

[43] Carter G.P., Chakravorty A., Pham Nguyen T.A., Mileto S., Schreiber F., Li L., Howarth P., Clare S., Cunningham B., Sambol S.P. (2015). Defining the roles of TcdA and TcdB in localized gastrointestinal disease, systemic organ damage, and the host response during *Clostridium difficile* infections. mBio.

