# Supplementary material for: Cationic Peptidomimetic Amphiphiles Having a N-Aryl- or N-Naphthyl-1,2,3-Triazole Core Structure Targeting Clostridioides (Clostridium) difficile: Synthesis, Antibacterial Evaluation, and an In Vivo C. difficile Infection Model
Source: Antibiotics (Basel). 2021 Jul 26;10(8):913. doi: 10.3390/antibiotics10080913 (PMC8388771; doi:10.3390/antibiotics10080913)
Supplement: Supplementary file 1 [file antibiotics-10-00913-s001.zip › C Diff peptidomimetics SI revised authorship.pdf]

antibiotics

MDPI

Article

Cationic Peptidomimetic Amphiphiles Having a *N*-Aryl- or *N*-Naphthyl-1,2,3-Triazole Core Structure Targeting *Clostridioides* (*Clostridium*) *difficile*: Synthesis, Antibacterial Evaluation, and an In Vivo *C. difficile* Infection Model

Muni Kumar Mahadari [1], Sreenu Jennepalli [1], Andrew J. Tague [1], Papanin Putsathit [2], Melanie L. Hutton [3], Katherine A. Hammer [4], Daniel R.Knight [4,5] Thomas V. Riley [2,4,5,6], Dena Lyras [3], Paul A. Keller [1,*] and Stephen G. Pyne [1,*]

[1] School of Chemistry and Biomolecular Science, University of Wollongong, Wollongong, NSW 2522, Australia; mkm933@uowmail.edu.au (M.K.M.); jennepal@ohsu.edu (S.J.); atague@uow.edu.au (A.J.T.)

[2] School of Medical and Health Sciences, Edith Cowan University, Perth, WA 6027, Australia; papanin.putsathit@uwa.edu.au (P.P.); thomas.riley@uwa.edu.au (T.V.R.)

[3] Infection and Immunity Program, Monash Biomedicine Discovery Institute and Department of Microbiology, Monash University, Clayton, VIC 3800, Australia; melanie.hutton@monash.edu (M.L.H.); dena.lyras@monash.edu (D.L.)

[4] School of Biomedical Sciences, The University of Western Australia, Perth, WA 6009, Australia; katherine.hammer@uwa.edu.au (K.A.H.); Daniel.Knight@murdoch.edu.au (D.R.K.)

[5] Biosecurity and One Health Research Centre, Harry Butler Institute, Murdoch University, Perth, WA 6150, Australia

[6] PathWest Laboratory Medicine, Queen Elizabeth II Medical Centre, Perth, WA 6009, Australia

* Correspondence: keller@uow.edu.au (P.A.K.); spyne@uow.edu.au (S.G.P.)

General synthetic procedures

<u>General Procedure I</u>: Alkylation of phenols (with ethyl bromoacetate)

To a stirred solution of the phenol (1 eq) in dry DMF (5 mL/mmol substrate) was added K_2CO_3 (3 eq), followed by ethyl bromoacetate (1.3 eq) at room temperature and the reaction was allowed to stir at rt for 12 h. The reaction mixture was diluted with EtOAc (2 x 50 mL), washed with water (2 x 50 mL), brine (2 x 50 mL) and dried ($MgSO_4$). The solution was filtered, concentrated under vacuum and the residue was subjected to silica gel flash column chromatography to afford the desired ester product.

<u>General Procedure II</u>: Ester hydrolysis (with KOH)

To a stirred solution of the ester (1 eq) in ethanol (10 mL/mmol substrate) was added 7% KOH solution (5 mL/mmol) at rt and the reaction was allowed to stir at rt for 2 h. The reaction mixture was acidified with 1 M HCl (25 mL) and extracted with EtOAc (2 x 25 mL). The combined organic extracts were washed with brine (50 mL) and dried ($MgSO_4$). The solution was filtered, concentrated under vacuum to afford the acid product.

<u>General Procedure III</u>: Amide coupling

The amine (1.0 eq), carboxylic acid (1.0 eq), EDC.HCl (1.2 eq), HOBt (1.1 eq) and TEA (1 eq) were combined in a dichloromethane/acetonitrile solution (10 mL/mmol amine) and stirred at rt for the specified time. The solvent was removed (not required for ≤ 5.0 mL dichloromethane/acetonitrile) and the residue was dissolved in EtOAc (25 mL for reactions that contained ≤ 1.0 mmol amine or 25 mL/mmol amine for larger scale reactions). The organic solution was washed successively with aqueous HCl (1.0 M – 2 x 25 mL), saturated aqueous $NaHCO_3$ (3 x 25 mL) and brine (1 x 25 mL). The EtOAc solution was dried (MgSO4), filtered and concentrated. If necessary, the residue was subjected to further purification *via* flash chromatography over silica gel to furnish the targeted amide product.

<u>General Procedure IV</u>: Copper-catalyzed azide-alkyne cycloaddition

To a stirred solution of the azide (1.0 eq) and alkyne (2.0 – 3.0 eq) in *tert*-butanol/water (4:1) at rt was added $CuSO_4{\cdot}5H_2O$ (0.2 eq), followed by sodium ascorbate (0.4 eq). Stirring was continued at rt (unless noted otherwise) for the specified time. Aqueous saturated NH_4Cl solution (1 mL), then water (20 mL) was added, and the mixture was extracted with EtOAc (20 mL for reactions that contained ≤ 1.0 mmol azide or 20 mL/mmol azide for larger scale reactions). The combined extracts were washed with water (2 x 25 mL), brine (2 x 25 mL) and dried ($MgSO_4$). The solution was filtered, concentrated under vacuum and the residue was subjected to flash chromatography over silica gel to afford the desired 1,4-disubstituted 1,2,3-triazole product.

<u>General Procedure V</u>: Ruthenium-catalyzed azide-alkyne cycloaddition

To a stirred solution of the azide (1 eq) in dry 1,4-dioxane (2 mL/mmol substrate), in a dried flask equipped with a condenser and under an atmosphere of nitrogen at room temperature, was added the alkyne (1.1 eq), followed by pentamethylcyclopentadienylbis(triphenylphosphine)ruthenium (II) chloride (0.1 eq). The mixture was stirred and heated at 70 °C for 12 h. The reaction mixture was cooled to room temperature and

saturated aqueous NH_4Cl solution (1 mL), then water (25 mL) was added, and the mixture was extracted with EtOAc (2 x 25 mL). The combined extracts were washed with water (2 x 25 mL), brine (2 x 25 mL) and dried ($MgSO_4$). The solution was filtered, concentrated under vacuum and the residue was subjected to flash chromatography over silica gel to afford the desired 1,5-disubstituted 1,2,3-triazole product.

General Procedure VI: Magnesium-promoted azide-alkyne cycloaddition

To a dried flask equipped with a condenser and containing a solution of EtMgCl (2 M in diethyl ether, 1.2 eq) in dry THF (10 mL/mmol) under a nitrogen atmosphere at room temperature, was added dropwise, *via* a syringe, the alkyne (1.1 eq). The mixture was stirred and heated at 50 °C for 30 min. The solution was cooled to rt and a solution of the azide (1 eq) in dry THF (10 mL/mmol) was added dropwise over a period of 5 min at rt. After a further 10 min, the reaction mixture was stirred and heated at 50 °C for 3-5 h. Aqueous saturated NH_4Cl solution (1 mL), then water (25 mL) was added, and the mixture was extracted with EtOAc (2 x 25 mL). The combined extracts were washed with water (2 x 25 mL), brine (2 x 25 mL) and dried ($MgSO_4$). The solution was filtered, concentrated under vacuum and the residue was subjected to silica gel flash column chromatography to afford the desired 1,5-disubstituted 1,2,3-triazole product.

General Procedure VII: Amine deprotection (*N*-Boc and/or *N*-Pbf removal)

The *N*-protected amine (1.0 eq) was dissolved in a CH_2Cl_2 (30 mL/mmol substrate) with magnetic stirring. If the substrate molecule contained an *N*-Pbf moiety then H_2O (20.0 eq) was also added to the solution. TFA (30.0 mL/mmol substrate) was then added and the reaction mixture was stirred at rt overnight (> 16 h) followed by removal of the solvent. The residue was dissolved in CH_2Cl_2 (30 mL/mmol substrate), an excess amount of anhydrous HCl (2.0 M in Et_2O, 15 mL/mmol substrate, 30.0 eq) was added and the solvent was then removed. The resulting residue was dissolved in a minimal volume of CH_2Cl_2 (or MeOH) and excess Et_2O (25 mL for ≤ 0.1 mmol substrate) was added to precipitate the hydrochloride salt of the amine. The reaction mixture was filtered, resulting filtrate was collected and concentrated, then triturated with Et_2O (3 x 20 mL). The product was collected by dissolution in MeOH; concentration followed by drying *in vacuo* gave the final mono or di-hydrochloride salt as a thin, translucent film that was routinely scratched with a spatula into a fine hygroscopic powder or amorphous gum.

1. Synthesis of acid scaffolds 5, 6, 17, 18, 27 and 28.

1.1 Synthesis of acid scaffold 5

Ethyl 2-(2-iodophenoxy)acetate[32]

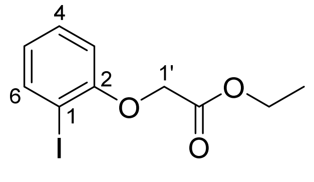

Following **General Procedure I**, 1-iodophenol (5.00 g, 22.72 mmol), K_2CO_3 (9.42 g, 68.18 mmol) and ethyl bromoacetate (4.93 g, 29.54 mmol) were stirred in dry DMF (40 mL) at rt for 16 h to give the ester **72** (6.52 g, 93%) as a pale brown oil

after flash chromatography over SiO_2 gel (EtOAc/*n*-hexane - 10:90). The spectroscopic data was found to be in agreement with those previously reported.[1] TLC (EtOAc/*n*-hexane - 20:80): R_f = 0.6; ^{1}H NMR (500 MHz, $CDCl_3$) δ 7.78 (d, *J* = 8.0 Hz, 1H, H6), 7.28-7.25 (m, 1H, H4), 6.78-6.71 (m, 2H, H3/H5), 4.67 (s, 2H, H1'), 4.25 (q, *J* = 7.0 Hz, 2H, OC<u>H</u>$_2$CH$_3$), 1.28 (t, *J* = 7.0 Hz, 3H, OCH$_2$C<u>H</u>$_3$); ^{13}C NMR (126 MHz, $CDCl_3$) δ 168.3 (C=O), 156.8 (C2), 139.7 (C6), 129.6 (C4), 123.6 (C5), 112.6 (C3), 86.5 (C1), 66.5 (C1') , 61.6 (O<u>C</u>H$_2$CH$_3$), 14.1 (OCH$_2$<u>C</u>H$_3$); IR (neat) $\bar{\nu}_{max}$ 3480, 2984, 2940, 1743, 1621, 1501, 1461, 1448, 1379, 1350, 1293, 1209, 1152, 1104, 802, 763 cm^{-1}; MS (ESI +ve) *m/z* 329 ([M + Na]$^+$, 100%).

Ethyl 2-(2-(4-isopentyl-1*H*-1,2,3-triazol-1-yl)phenoxy)acetate

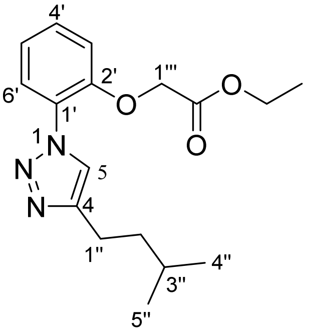

To a stirred solution of ethyl 2-(2-iodophenoxy)acetate (1.00 g, 3.26 mmol), 5-methyl-1-hexyne (0.94 g, 9.80 mmol), CuI (0.12 g, 0.65 mmol), NaN_3 (0.23 g, 3.59 mmol) and sodium ascorbate (0.26 g, 1.30 mmol) in DMSO (10 mL)/H_2O (2 mL) was added racemic *trans-N,N'*-dimethylcyclohexane-1,2-diamine (0.09 g, 0.65 mmol) at rt under nitrogen atmosphere. The reaction mixture was stirred and heated at 75 °C for 16 h. The reaction was cooled to rt and aqueous saturated NH_4Cl solution (10 mL) was added and the mixture was extracted with EtOAc (2 x 50 mL). The combined extracts were washed with water (50 mL), brine (50 mL) and dried ($MgSO_4$). The solution was filtered, concentrated under vacuum and the residue was subjected to silica gel flash column chromatography over silica gel (EtOAc/*n*-hexane - 10:90 → 100:0) to afford titled compound (0.91 g, 87%) as a yellow waxy solid. TLC (EtOAc/*n*-hexane - 20:80); R_f = 0.5; ^{1}H NMR (500 MHz, $CDCl_3$) δ 8.16 (s, 1H, H5), 7.87 (d, *J* = 7.5 Hz, 1H, H6'), 7.35 (apparent t, *J* = 7.5 Hz, 1H, H4'), 7.13 (apparent t, *J* = 7.5 Hz, 1H, H5'), 6.96 (d, *J* = 7.5 Hz, 1H, H3'), 4.68 (s, 2H, H1'''), 4.25 (q, *J* = 7.0 Hz, 2H, OC<u>H</u>$_2$CH$_3$), 2.81 (t, *J* = 7.5 Hz, 2H, H1''), 1.70-1.61 (m, 3H, H2''/H3''), 1.29 (t, *J* = 7.5 Hz, 3H, OCH$_2$C<u>H</u>$_3$), 0.96 (d, *J* = 6.0 Hz, 6H, H4''/H5''); ^{13}C NMR (126 MHz, $CDCl_3$) δ 168.0 (C=O), 149.0 (C2'), 148.1 (C4), 129.4 (C4'), 127.2 (C6'), 125.4 (C5'), 123.1 (C5), 122.4 (C3'), 113.3 (C1'), 65.9 (C1'''), 61.6 (O<u>C</u>H$_2$CH$_3$), 38.5 (C2''), 27.7 (C1''), 23.7 (C3''), 22.4 (C4''/C5''; Observed by gHMBC), 14.1 (OCH$_2$<u>C</u>H$_3$); IR (neat) $\bar{\nu}_{max}$ 2957, 2870, 1755, 1601, 1548, 1509, 1466, 1379, 1296, 1230, 1203, 1167, 1129, 1073, 977, 758 cm^{-1}; MS (ESI +ve) *m/z* 318 ([M + H]$^+$, 100%); HRMS (ESI +ve TOF) calcd for $C_{17}H_{24}N_3O_3$ 318.1818, found 318.1831 ([M + H]$^+$).

2-(2-(4-Isopentyl-1*H*-1,2,3-triazol-1-yl)phenoxy)acetic acid (5)

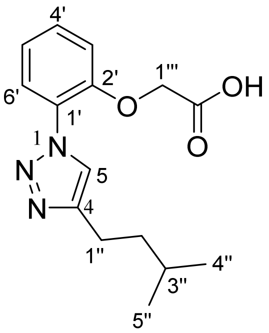

Following **General Procedure II**, ethyl 2-(2-(4-isopentyl-1*H*-1,2,3-triazol-1-yl)phenoxy) acetate (0.90 g, 2.84 mmol) and 7% KOH solution (3 mL) were stirred in ethanol (10 mL) at rt for 2 h to give the acid **5** (0.71 g, 85%) as a white solid. M.P: 118 - 120 °C. TLC (EtOAc/*n*-hexane - 100:0): R_f = 0.2; ^{1}H NMR (500 MHz, $CDCl_3$) δ 9.36 (brs, 1H, COO<u>H</u>), 8.19 (s, 1H, H5), 7.77 (d, *J* = 7.5 Hz, 1H, H6'), 7.39 (apparent t, *J* = 7.5 Hz, 1H, H4'), 7.13 (apparent t, *J* = 7.5 Hz, 1H, H5'), 7.04 (d, *J* = 7.5 Hz, 1H, H3'), 4.76

(s, 2H, H1'''), 2.80 (t, J = 7.5 Hz, 2H, H1''), 1.64-1.59 (m, 3H, H2''/H3''), 0.92 (d, J = 5.5 Hz, 6H, H4''/H5''); ^{13}C NMR (126 MHz, $CDCl_3$) δ 170.8 (C=O), 149.4 (C2'), 147.9 (C4), 130.2 (C4'), 126.7 (C6'), 125.4 (C5'), 123.7 (C5), 122.5 (C3'), 113.6 (C1'), 65.9 (C1'''), 38.3 (C2''), 27.7 (C1''), 23.2 (C3''), 22.4 (C4''/5''; Observed by gHMBC); IR (neat) $\bar{\nu}_{max}$ 3160, 2955, 2930, 1737, 1602, 1505 1471, 1439, 1294, 1219, 1181, 1129, 1065, 835, 753 cm^{-1}; MS (ESI +ve) *m/z* 290 ([M + H]$^+$, 100%); HRMS (ESI +ve TOF) calcd for $C_{15}H_{20}N_3O_3$ 290.1505, found 290.1513 ([M + H]$^+$).

1.2 Synthesis of acid scaffold 6

Ethyl 2-(2-azidophenoxy)acetate

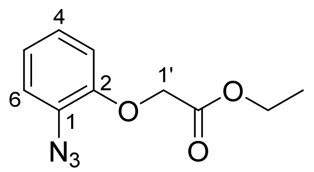

To a stirred solution of ethyl 2-(2-iodophenoxy)acetate (1.00 g, 3.26 mmol), CuI (0.06 g, 0.32 mmol), NaN_3 (0.24 g, 3.59 mmol) and sodium ascorbate (0.06 g, 0.32 mmol) in DMSO (10 mL)/H_2O (2 mL) was added racemic *trans-N,N'*-dimethylcyclohexane-1,2-diamine (0.07 g, 0.49 mmol) at rt under nitrogen atmosphere. The reaction mixture was stirred and heated at 80 °C for 5 h. The reaction was diluted with water (100 mL) and extracted with EtOAc (2 x 100 mL). The combined extracts were washed with water (100 mL), brine (50 mL) and dried ($MgSO_4$). The solution was filtered, concentrated under vacuum and the residue was subjected to silica gel flash column chromatography over silica gel (EtOAc/*n*-hexane - 10:90 → 100:0) to afford the titled compound (0.51 g, 70%) as a yellow waxy solid. TLC (EtOAc/CH_2Cl_2/*n*-hexane – 10:10:80); R_f = 0.5; ^{1}H NMR (400 MHz, $CDCl_3$) δ 7.08-6.95 (m, 3H, H4/H5/H6), 6.81 (dd, J = 8.1, 1.3 Hz, 1H, H3), 4.67 (s, 2H, H1'), 4.26 (q, J = 7.1 Hz, 2H, $OC\underline{H}_2CH_3$), 1.28 (t, J = 7.1 Hz, 3H, $OCH_2C\underline{H}_3$); ^{13}C NMR (101 MHz, $CDCl_3$) δ 168.3 (C=O), 150.2 (C2), 129.1 (C1), 125.4 (C6), 122.6 (C4), 120.7 (C5), 113.8 (C3), 66.2 (C1'), 61.4 ($OC\underline{H}_2CH_3$), 14.12 ($OCH_2C\underline{H}_3$); IR (neat) $\bar{\nu}_{max}$ 2984, 2116, 1756, 1738, 1592, 1495, 1473, 1454, 1379, 1297, 1199, 1106, 1072, 1020, 749 cm^{-1}; MS (ESI +ve) *m/z* 194 ([M + H – N_2]$^+$, 100%).

Ethyl 2-(2-(5-isopentyl-1*H*-1,2,3-triazol-1-yl)phenoxy)acetate

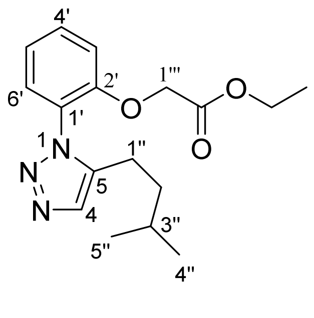

Following **General Procedure V**, ethyl 2-(2-azidophenoxy)acetate (0.53 g, 2.40 mmol), 5-methyl-1-hexyne (0.46 g, 4.80 mmol) and pentamethylcyclopentadienylbis(triphenylphosphine)ruthenium(II) chloride (0.19 g, 0.24 mmol) were stirred and heated in 1,4-dioxane at 70 °C for 12 h to give the titled ester (0.28 g, 37%) as a pale-yellow oil after flash chromatography over silica gel (EtOAc/*n*-hexane - 20:80). TLC (EtOAc/*n*-hexane – 50:50): R_f = 0.3; ^{1}H NMR (400 MHz, $CDCl_3$) δ 7.55 (s, 1H, H4), 7.46 (apparent t, J = 6.8 Hz, 1H, H4'), 7.37 (d, J = 6.4 Hz, 1H, H6'), 7.14 (apparent t, J = 6.4 Hz, 1H, H5'), 6.92 (d, J = 6.8 Hz, 1H, H3'), 4.55 (s, 2H, H1'''), 4.20 (q, J = 5.6 Hz, 2H, $OC\underline{H}_2CH_3$), 2.64-2.56 (m, 2H, H1''), 1.56-1.42 (m, 3H, H2''/H3''), 1.24 (t, J = 5.6 Hz, 3H, $OCH_2C\underline{H}_3$), 0.81 (d, J = 5.2 Hz, 6H, H4''/H5''); ^{13}C NMR (101 MHz, $CDCl_3$) δ 168.1 (C=O), 152.6 (C2'), 140.6 (C5), 131.5 (C4), 131.5 (C4'),

129.2 (C6'), 125.8 (C1'), 122.2 (C5'), 113.2 (C3'), 65.7 (C1'''), 61.6 ($O\underline{C}H_2CH_3$), 37.0 (C2''), 27.6 (C1''), 22.3 (C4''/C5''), 21.2 (C3''), 14.2 ($OCH_2\underline{C}H_3$); IR (neat) $\bar{\nu}_{max}$ 2957, 2935, 2870, 1755, 1602, 1509, 1466, 1445, 1381, 1369, 1295, 1203, 1167, 1129, 1073, 1025, 977, 757 cm^{-1}; MS (ESI +ve) *m/z* 340 ($[M + Na]^+$, 100%); HRMS (ESI +ve TOF) calcd for $C_{17}H_{23}N_3O_3Na$ 340.1637, found 340.1638 ($[M + Na]^+$).

2-(2-(5-Isopentyl-1*H*-1,2,3-triazol-1-yl)phenoxy)acetic acid (6)

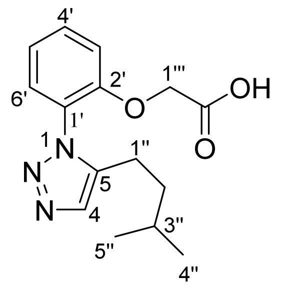

Following **General Procedure II**, ethyl 2-(2-(5-isopentyl-1*H*-1,2,3-triazol-1-yl)phenoxy)acetate (0.28 g, 0.88 mmol) and 7% KOH solution (4 mL) were stirred in ethanol (4 mL) at rt for 2 h to give the product acid **6** (0.21 g, 78%) as a pale brown solid. M.P: 128-130 °C. TLC (EtOAc/*n*-hexane - 100:0): $R_f = 0.2$; ^{1}H NMR (500 MHz, $CDCl_3$) δ 8.10 (brs, 1H, COOH), 7.62 (s, 1H, H4), 7.49 (apparent t, $J = 8.0$ Hz, 1H, H4'), 7.35 (d, $J = 7.5$ Hz, 1H, H6'), 7.15 (apparent t, $J = 7.5$ Hz, 1H, H5'), 7.03 (d, $J = 8.0$ Hz, 1H, H3'), 4.64 (s, 2H, H1'''), 2.61 (t, $J = 7.5$ Hz, 2H, H1''), 1.56-1.42 (m, 3H, H2''/H3''), 0.81 (d, $J = 6.5$ Hz, 6H, H4''/H5''); ^{13}C NMR (126 MHz, $CDCl_3$) δ 170.6 (C=O), 152.4 (C2'), 140.6 (C5), 131.9 (C4), 131.6 (C4'), 128.5 (C6'), 125.6 (C1'), 122.6 (C5'), 114.1 (C3'), 66.0 (C1'''), 37.1 (C2''), 27.6 (C1''), 22.3 (C4''/C5''; Observed by gHMBC), 21.3 (C3''); IR (neat) $\bar{\nu}_{max}$ 2960, 2929, 2871, 1722, 1602, 1509, 1465, 1371, 1296, 1231, 1164, 1110, 1072, 995, 840, 760 cm^{-1}; MS (ESI +ve) *m/z* 328 ($[M + K]^+$, 100%), 290 ($[M + H]^+$, 60%); HRMS (ESI +ve TOF) calcd for $C_{15}H_{19}N_3O_3Na$ 312.1324, found 312.1329 ($[M + Na]^+$).

1.3 Synthesis of acid scaffold 17

Ethyl 2-((1-iodonaphthalen-2-yl)oxy)acetate

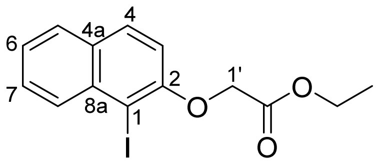

Following **General Procedure I**, 1-iodonaphthol (1.00 g, 3.70 mmol), K_2CO_3 (1.53 g, 11.11 mmol) and ethyl bromoacetate (0.80 g, 4.81 mmol) were stirred in DMF (8 mL) at rt for 16 h to give the titled ester (0.68 g, 52%) as a pale yellow waxy solid after flash chromatography over silica gel (EtOAc/*n*-hexane - 10:90). TLC (EtOAc/*n*-hexane - 20:80): $R_f = 0.6$; ^{1}H NMR (400 MHz, $CDCl_3$) δ 8.16 (d, $J = 7.2$ Hz, 1H, H8), 7.78 (d, $J = 7.2$ Hz, 1H, H5), 7.72 (d, $J = 8.0$ Hz, 1H, H4), 7.54 (t, $J = 7.2$ Hz, 1H, H7), 7.39 (t, $J = 7.2$ Hz, 1H, H6), 7.08 (d, $J = 8.0$ Hz, 1H, H3), 4.80 (s, 2H, H1'), 4.27 (q, $J = 5.6$ Hz, 2H, $OC\underline{H}_2CH_3$), 1.29 (t, $J = 5.6$ Hz, 3H, $OCH_2C\underline{H}_3$); ^{13}C NMR (101 MHz, $CDCl_3$) δ 168.7 (C=O), 155.6 (C2), 135.8 (C8a), 131.7 (C4a), 130.6 (C4), 130.5 (C8), 128.4 (C7), 128.3 (C5), 121.1 (C6), 114.4 (C3), 89.47 (C1), 67.6 (C1'), 61.7 ($O\underline{C}H_2CH_3$) 14.3 ($OCH_2\underline{C}H_3$); IR (neat) $\bar{\nu}_{max}$ 2981, 1756, 1622, 1593, 1502, 1462, 1349, 1291, 1200, 1151, 1134, 1096, 1028, 801, 764, 747 cm^{-1}; MS (ESI +ve) *m/z* 379 ($[M + Na]^+$, 100%); HRMS (ESI +ve TOF) calcd for $C_{14}H_{13}O_3NaI$ 378.9807, found 378.9801 ($[M + Na]^+$).

Ethyl 2-((1-(4-isopentyl-1*H*-1,2,3-triazol-1-yl)naphthalen-2-yl)oxy)acetate

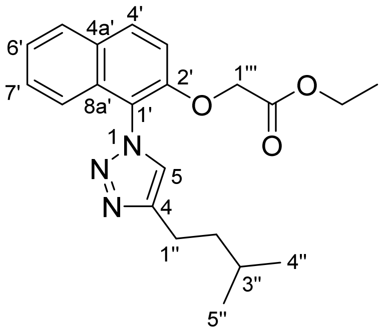

To a stirred solution of ethyl 2-(2-iodophenoxy)acetate (0.20 g, 0.54 mmol), 5-methyl-1-hexyne (0.16 g, 1.64 mmol), CuI (0.02 g, 0.11 mmol), NaN_3 (0.04 g, 0.60 mmol) and sodium ascorbate (0.04 g, 0.22 mmol) in DMSO (2.5 mL) H_2O (0.5 mL) was added racemic *trans-N,N'*-dimethyl cyclohexane-1,2-diamine (0.016 g, 0.11 mmol) at rt under a nitrogen atmosphere. The reaction mixture was stirred and heated at 75 °C for 16 h. The reaction was cooled to rt and aqueous saturated NH_4Cl solution (3 mL) was added and the mixture was extracted with EtOAc (2 x 25 mL). The combined extracts were washed with water (25 mL), brine (25 mL) and dried ($MgSO_4$). The solution was filtered, concentrated under vacuum and the residue was subjected to silica gel flash column chromatography (EtOAc/*n*-hexane - 10:90 → 100:0) to afford the titled compound (0.05 g, 25%) as a yellow waxy solid. TLC (EtOAc/*n*-hexane - 33:67); R_f = 0.4; ^{1}H NMR (400 MHz, $CDCl_3$) δ 7.97 (d, *J* = 7.2 Hz, 1H, H8'), 7.84 (d, *J* = 6.4 Hz, 1H, H5'), 7.67 (s, 1H, H5), 7.49-7.41 (m, 2H, H6'/H7'), 7.27-7.25 (m, 2H, H3'/H4'), 4.67 (s, 2H, H1'''), 4.22 (q, *J* = 5.6 Hz, 2H, OC$\underline{H}_2$$CH_3$), 2.89 (t, *J* = 5.6 Hz, 2H, H1''), 1.73-1.67 (m, 3H, H2''/H3''), 1.26 (t, *J* = 5.6 Hz, 3H, OCH_2OC$\underline{H}_3$), 0.99 (d, *J* = 4.0 Hz, 6H, H4''/H5''); ^{13}C NMR (101 MHz, $CDCl_3$) δ 168.5 (C=O), 150.5 (C2'), 148.1 (C8a'), 131.6 (C4), 131.3 (C4a'), 129.5 (C4'), 128.5 (C5'), 127.9 (C7'), 125.3 (C8'), 124.7 (C6'), 122.1 (C5), 121.3 (C3'), 114.3 (C1'), 66.7 (C1'''), 61.6 (O$\underline{C}H_2CH_3$), 38.6 (C2''), 27.9 (C1''), 23.8 (C3''), 22.5 (C4''/C5''; Observed by gHMBC), 14.2 ($OCH_2$$\underline{C}H_3$); IR (neat) $\bar{\nu}_{max}$ 2954, 2928, 2868, 1748, 1632, 1600, 1513, 1483, 1454, 1430, 1366, 1288, 1206, 1150, 1117, 1087, 1042, 806, 749 cm^{-1}; MS (ESI +ve) *m/z* 390 ([M +Na]$^+$, 100%); HRMS (ESI +ve TOF) calcd for $C_{21}H_{26}N_3O_3$ 368.1974, found 368.1985 ([M + H]$^+$).

2-((1-(4-Isopentyl-1*H*-1,2,3-triazol-1-yl)naphthalen-2-yl)oxy)acetic acid (17)

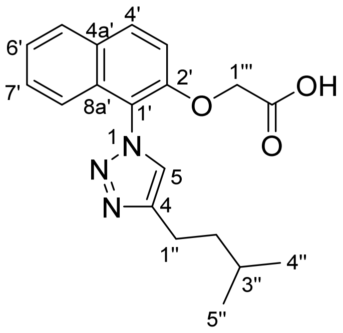

Following **General Procedure II**, ethyl 2-((1-(4-isopentyl-1*H*-1,2,3-triazol-1-yl)naphthalen-2-yl)oxy)acetate **76** (0.07 g, 0.19 mmol) and 7% KOH solution (0.5 mL) were stirred in ethanol (2 mL) at rt for 2 h to give after acidification the acid **54** (0.04 g, 62%) as a white solid. M.P: 152 – 154 °C. TLC (EtOAc/*n*-hexane - 100:0): R_f = 0.2; ^{1}H NMR (500 MHz, $CDCl_3$) δ 8.00 (d, *J* = 9.0 Hz, 1H, H8'), 7.88 (d, *J* = 7.5 Hz, 1H, H5'), 7.69 (s, 1H, H5), 7.54-7.46 (m, 2H, H6'/H7'), 7.47-7.29 (m, 2H, H3'/H4'), 4.78 (s, 2H, H1'''), 2.91-2.87 (m, 2H, H1''), 1.71-1.68 (m, 3H, H2''/H3''), 0.98 (d, *J* = 6.0 Hz, 6H, H4''/H5''), COOH resonance was not observed; ^{13}C NMR (126 MHz, $CDCl_3$) δ 170.6 (C=O), 150.4 (C2'), 148.4 (C8a'), 132.2 (C4), 130.7 (C4a'), 129.6 (C4'), 128.8 (C5'), 128.2 (C7'), 125.6 (C8'), 124.9 (C6'), 121.7 (C5), 120.9 (C3'), 114.4 (C1'), 66.8 (C1'''), 38.5 (C2''), 28.0 (C1''), 23.7 (C3''), 22.6 (C4''/C5''; Observed by gHMBC); IR (neat) $\bar{\nu}_{max}$ 3147, 2954, 2929, 2868, 1731, 1631, 1600, 1514, 1483, 1429, 1366, 1284, 1213, 1151, 1118, 1087, 1062, 923, 806, 748 cm^{-1}; MS (ESI +ve) *m/z* 362 ([M +

Na]$^+$, 40%), 340 ([M + H]$^+$, 100%); HRMS (ESI +ve TOF) calcd for $C_{19}H_{22}N_3O_3$ 340.1661, found 340.1667 ([M + H]$^+$).

1.4 Synthesis of acid scaffold 18

2-Methoxy-1-nitronaphthalene[33]

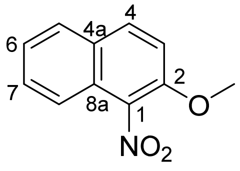

To an ice-cold solution of 2-methoxynaphthalene (3.00 g, 18.96 mmol) in acetic anhydride (35 mL) was added fuming HNO_3 (1.32 g, 20.85 mmol) dropwise at 0 °C. The mixture was stirred at rt for 1 h and placed in a freezer for 16 h. The deposited crystals were filtered, washed with acetic anhydride, water and dried under vacuum for 3 h to afford the titled nitro compound (0.86 g, 22%) as a yellow solid. TLC (EtOAc/*n*-hexane - 20:80): R_f = 0.5; The spectroscopic data were found to be in agreement with that previously reported.[2] ^{1}H NMR (400 MHz, $CDCl_3$) δ 7.96 (d, J = 8.2 Hz, 1H, H8), 7.84 (d, J = 9.1 Hz, 1H, H4), 7.68 (dd, J = 8.5, 0.8 Hz, 1H, H5), 7.61-7.57 (m, 1H, H7), 7.47-7.43 (m, 1H, H6), 7.34 (d, J = 9.1 Hz, 1H, H3), 4.03 (s, 3H, OMe); ^{13}C NMR (101 MHz, $CDCl_3$) δ 148.6 (C2), 132.1 (C8a), 129.1 (C4), 128.2 (C4a), 128.1 (C7), 128.0 (8), 125.6 (C6), 125.1 (C1), 120.4 (C5), 113.0 (C3), 57.0 (OMe); IR (neat) $\bar{\nu}_{max}$ 3030, 2944, 2846, 1636, 1602, 1515, 1459, 1436, 1357, 1280, 1259, 1220, 1155, 1079, 809, 795 cm^{-1}; MS (ESI +ve) *m/z* 204 ([M + H]$^+$, 100%).

2-Methoxynaphthalen-1-amine[34]

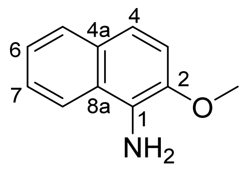

Iron powder (0.14 g, 0.49 mmol) was added to a stirred solution of 2-methoxy-1-nitronaphthalene (0.10 g, 0.49 mmol) in EtOH:H_2O:AcOH (2 mL : 1 mL : 2 mL) at rt and the mixture was sonicated for 2 h. The reaction was basified with aqueous KOH solution (3 mL) and extracted with EtOAc (2 x 25 mL). The combined extracts were washed with water (25 mL), brine (25 mL) and dried ($MgSO_4$). The solution was filtered, concentrated under vacuum and the residue was subjected to silica gel flash column chromatography (EtOAc/*n*-hexane - 10:90 → 100:0) to afford the titled compound (0.07 g, 82%) as a brown oil. TLC (EtOAc/*n*-hexane - 20:80); R_f = 0.5; The spectroscopic data were found to be in agreement with those previously reported.[3] ^{1}H NMR (400 MHz, $CDCl_3$) δ 7.76-7.73 (m, 2H, H8/H5), 7.43-7.39 (m, 1H, H7), 7.33-7.29 (m, 2H, H4/H6), 7.22 (d, J = 8.8 Hz, 1H, H3), 4.22 (brs, 2H, NH_2), 3.95 (s, 3H, OMe); ^{13}C NMR (101 MHz, $CDCl_3$) δ 142.5 (C2), 129.5 (C4), 129.4 (C4a), 128.3 (C8a), 125.0 (C5), 123.9 (C7), 123.5 (C8), 120.2 (C1), 118.4 (C6), 113.5 (C3), 56.7 (OMe); IR (neat) $\bar{\nu}_{max}$ 3452, 3366, 2936, 2837, 1683, 1609, 1513, 1475, 1407, 1268, 1242, 1097, 1047, 794, 744 cm^{-1}; MS (ESI +ve) *m/z* 174 ([M + H]$^+$, 60%), 142 (60%), 91 (100%).

1-Azido-2-methoxynaphthalene

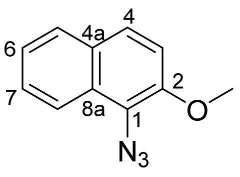

A solution of $NaNO_2$ (0.04 g, 0.60 mmol) in H_2O (0.5 mL) was added dropwise to a stirred suspension of 2-methoxynaphthalen-1-amine (0.07 g, 0.40 mmol) in 3 N HCl solution (2 mL) at 0 °C and stirring at 0 °C was continued for 15 min. Then NaN_3 (0.05 g,

0.80 mmol) in H_2O (0.5 mL) was added at 0 °C and after 10 min the mixture was continued stirring at rt for 3 h. The reaction was diluted with water (10 mL) and extracted with EtOAc (2 x 20 mL). The combined extracts were washed with water (20 mL), brine (20 mL) and dried ($MgSO_4$). The solution was filtered, concentrated under vacuum and the residue was subjected to silica gel flash column chromatography (EtOAc/*n*-hexane - 5:95 → 100:0) to afford titled azide (0.025 g, 31%) as a brown waxy solid. TLC (EtOAc/*n*-hexane – 20:80); $R_f = 0.7$; ^{1}H NMR (400 MHz, $CDCl_3$) δ 8.07 (dd, J = 8.5, 1.0 Hz, 1H, H8), 7.72 (d, J = 8.2 Hz, 1H, H5), 7.62 (d, J = 9.0 Hz, 1H, H4), 7.47-7.43 (m, 1H, H7), 7.37-7.33 (m, 1H, H6), 7.23 (d, J = 9.0 Hz, 1H, H3), 4.01 (s, 3H, OMe); ^{13}C NMR (101 MHz, $CDCl_3$) δ 150.3 (C2), 129.3 (C8a), 127.7 (C4), 127.5 (C4a), 126.5 (C5), 125.6 (C8), 124.4 (C7), 122.4 (C1), 121.4 (C6), 114.0 (C3), 57.1 (OMe); IR (neat) $\bar{\nu}_{max}$ 3058, 3005, 2938, 2841, 2103, 2045, 1591, 1507, 1441, 1375, 1290, 1146, 1084, 1017, 912, 806, 747 cm^{-1}; MS (ESI +ve) *m/z* 172 ($[M + H – N_2]^+$, 100%).

5-Isopentyl-1-(2-methoxynaphthalen-1-yl)-1*H*-1,2,3-triazole

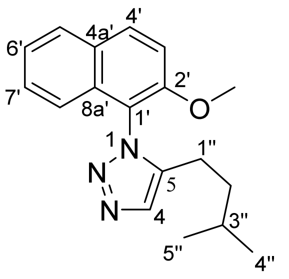

Following **General Procedure VI**, 1-azido-2-methoxynaphthalene (0.05 g, 0.25 mmol) was treated with 5-methyl-1-hexyne (0.03 g, 0.27 mmol) and EtMgCl (0.03 g, 0.3 mmol; 2 M in Et_2O) in dry THF (2 mL) at 50 °C for 3 h to give the triazole product (0.06 g, 81%) as a pale brown waxy solid after flash chromatography over silica gel (EtOAc/*n*-hexane - 20:80). TLC (EtOAc/*n*-hexane - 20:80): $R_f = 0.3$; ^{1}H NMR (400 MHz, $CDCl_3$) δ 8.03 (d, J = 9.1 Hz, 1H, H8'), 7.85 (dd, J = 7.4, 2.0 Hz, 1H, H5'), 7.71 (s, 1H, H4), 7.45-7.37 (m, 3H, H6', H7' and H4'), 6.97 (d, J = 9.0 Hz, 1H, H3'), 3.87 (s, 3H, OMe), 2.45-2.34 (m, 2H, H1''), 1.47-1.35 (m, 3H, H2'' and H3''), 0.73 (d, J = 7.9 Hz, 3H, H5''), 0.72 (d, J = 7.9 Hz, 3H, H4''); ^{13}C NMR (101 MHz, $CDCl_3$) δ 152.7 (C2'), 140.7 (C5), 132.0 (C8a'), 131.6 (C4), 131.4 (C4a'), 128.7 (C4'), 128.3 (C5'), 127.9 (C7'), 124.6 (C8'), 121.2 (C6'), 117.9 (C3'), 113.1 (C1'), 56.5 (OMe), 36.7 (C2''), 27.2 (C1''), 22.08 (C5''), 22.07 (C4''), 20.8 (C3''); IR (neat) $\bar{\nu}_{max}$ 2954, 2868, 1630, 1599, 1507, 1457, 1275, 1255, 1064, 1040, 809, 748 cm^{-1}; MS (ESI +ve) *m/z* 318 ($[M + Na]^+$, 50%), 296 ($[M + H]^+$, 100%); HRMS (ESI +ve TOF) calcd for $C_{18}H_{22}N_3O$ 296.1763, found 296.1750 ($[M + H]^+$).

1-(5-Isopentyl-1*H*-1,2,3-triazol-1-yl)naphthalen-2-ol

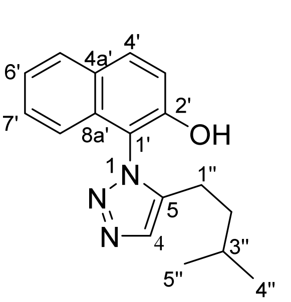

BBr_3 (0.13 g, 0.51 mmol) was added dropwise to a stirred solution of 5-isopentyl-1-(2-methoxynaphthalen-1-yl)-1*H*-1,2,3-triazole (0.05 g, 0.17 mmol) in CH_2Cl_2 (1 mL) at -78 °C and after 30 min the mixture was allowed to stir at rt for 36 h. The reaction was quenched with MeOH (0.5 mL), diluted with EtOAc (25 mL), washed with water (25 mL), brine (25 mL) and dried ($MgSO_4$). The solution was filtered, concentrated under vacuum and the residue was subjected to silica gel flash column chromatography (EtOAc/*n*-hexane - 20:80 → 100:0) to afford titled phenol (0.04 g, 82%) as a pale brown solid. M.P: 164 - 166 °C. TLC (EtOAc/*n*-hexane – 40:60); $R_f = 0.5$; ^{1}H NMR (400 MHz, $CDCl_3$) δ 10.31 (brs, 1H, OH),

7.78-7.74 (m, 2H, H8'/H5'), 7.62 (s, 1H, H4), 7.44 (d, *J* = 8.5 Hz, 1H, H4'), 7.38-7.29 (m, 2H, H7'/H6'), 6.84 (d, *J* = 8.5 Hz, 1H, H3'), 2.52-2.32 (m, 2H, H1"), 1.42-1.24 (m, 3H, H2"/H3"), 0.69 (d, *J* = 6.2 Hz, 3H, H5"), 0.65 (d, *J* = 6.2 Hz, 3H, H4"); ^{13}C NMR (101 MHz, $CDCl_3$) δ 151.6 (C2'), 141.5 (C5), 131.9 (C8a'), 131.4 (C4), 131.2 (C4a'), 128.3 (C4'), 128.0 (C5'), 127.9 (C7'), 123.8 (C8'), 120.4 (C6'), 119.1 (3'), 115.0 (C1'), 36.5 (C2"), 27.2 (C1"), 22.0 (C4"/C5"), 20.8 (C3"); IR (neat) $\bar{\nu}_{max}$ 3061, 2957, 2869, 1638, 1583, 1512, 1440, 1360, 1287, 1245, 1149, 997, 951, 818, 749 cm^{-1}; MS (ESI +ve) *m/z* 304 ($[M + Na]^+$, 100%); HRMS (ESI +ve TOF) calcd for $C_{17}H_{20}N_3O$ 282.1606, found 282.1602 ($[M + H]^+$).

Ethyl 2-((1-(5-isopentyl-1*H*-1,2,3-triazol-1-yl)naphthalen-2-yl)oxy)acetate

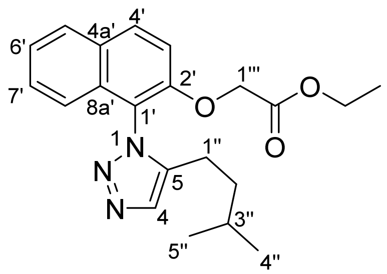

Following **General Procedure I**, 1-(5-isopentyl-1*H*-1,2,3-triazol-1-yl)naphthalen-2-ol (0.04 g, 0.14 mmol), K_2CO_3 (0.06 g, 0.42 mmol) and ethyl bromoacetate (0.03 g, 0.18 mmol) were stirred in DMF (1 mL) at rt for 16 h to give the ester product (0.03 g, 58%) as a pale brown oil after flash chromatography over silica gel (EtOAc/*n*-hexane - 20:80). TLC (EtOAc/*n*-hexane - 40:60): R_f = 0.6; ^{1}H NMR (400 MHz, $CDCl_3$) δ 8.00 (d, *J* = 7.2 Hz, 1H, H8'), 7.86 (dd, *J* = 7.3, 2.0 Hz, 1H, H5'), 7.71 (s, 1H, H4), 7.46-7.40 (m, 2H, H6'/H7'), 7.24 (d, *J* = 8.5 Hz, 1H, H4'), 7.02 (d, *J* = 8.5 Hz, H3'), 4.66 (ABq, *J* = 19.0 Hz, 2H, H1'''), 4.22 (q, *J* = 5.6 Hz, 2H, $OC\underline{H}_2CH_3$), 2.63-2.55 (m, 1H, H1"), 2.44-2.38 (m, 1H, H1"), 1.48-1.41 (m, 3H, H2"/H3"), 1.24 (t, *J* = 5.6 Hz, 3H, $OCH_2C\underline{H}_3$), 0.75 (d, *J* = 5.1 Hz, 3H, H5"), 0.72 (d, *J* = 5.0 Hz, 3H, H4"); ^{3}C NMR (101 MHz, $CDCl_3$) δ 168.2 (C=O), 151.0 (C2'), 141.2 (C5), 131.9 (C8a'), 131.6 (C4), 131.4 (C4'), 129.3 (C4a'), 128.4 (C5'), 127.9 (C7'), 125.1 (C8'), 121.5 (C6'), 118.9 (C3'), 113.8 (C1'), 66.2 (C1'''), 61.5 ($O\underline{C}H_2CH_3$), 36.6 (C2"), 27.3 (C1"), 22.1 (C5"), 22.0 (C4"), 20.8 (C3"), 14.1 ($OCH_2\underline{C}H_3$); IR (neat) $\bar{\nu}_{max}$ 2956, 2930, 2869, 1752, 1631, 1600, 1512, 1483, 1292, 1202, 1152, 1095, 1025, 808, 749 cm^{-1}; MS (ESI +ve) *m/z* 390 ($[M + Na]^+$, 100%); HRMS (ESI +ve TOF) calcd for $C_{21}H_{26}N_3O_3$ 368.2356, found 368.2363 ($[M + H]^+$).

2-((1-(5-Isopentyl-1*H*-1,2,3-triazol-1-yl)naphthalen-2-yl)oxy)acetic acid (18)

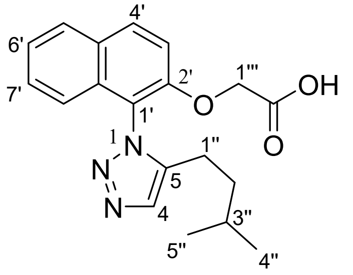

Following **General Procedure II**, ethyl 2-((1-(5-isopentyl-1*H*-1,2,3-triazol-1-yl)naphthalen-2-yl)oxy)acetate (0.28 g, 0.76 mmol) and 7% KOH solution (3 mL) were stirred in ethanol (3 mL) at rt for 2 h to give the acid product (0.22 g, 85%) as a white solid. M.P: 148 - 150 °C. TLC (EtOAc/*n*-hexane - 100:0): R_f = 0.2; ^{1}H NMR (400 MHz, $CDCl_3$) δ 8.01 (d, *J* = 9.0 Hz, 1H, H8'), 7.88-7.86 (m, 1H, H5'), 7.75 (s, 1H, H4), 7.49-7.42 (m, H6'/H7'), 7.30 (d, *J* = 8.5 Hz, 1H, H4'), 7.01 (d, *J* = 8.5 Hz, 1H, H3'), 4.72 (s, 2H, H1'''), 4.59 (brs, 1H, COOH), 2.57-2.49 (m, 1H, H1"), 2.44-2.37 (m, 1H, H1"), 1.46-1.33 (m, 3H, H2"/H3"), 0.72 (d, *J* = 6.2 Hz, 3H, H5"), 0.70 (d, *J* = 6.1 Hz, 3H, H4"); ^{13}C NMR (101 MHz, $CDCl_3$) δ 170.4 (C=O), 151.1 (C2'), 141.6 (C5), 132.3 (C8a'), 131.2 (C4), 131.2 (4'), 129.3 (C4a'), 128.6 (C5'), 128.1 (C7'), 125.2 (C8'), 121.3 (C6'), 118.6 (3'), 113.9 (C1'), 66.2 (C1'''), 36.5 (C2"), 27.2

(C1''), 22.0 (C4''/C5''), 20.9 (C3''); IR (neat) $\bar{\nu}_{max}$ 2956, 2935, 2869, 1738, 1631, 1601, 1513, 1484, 1428, 1277, 1218, 1152, 1093, 811, 751 cm^{-1}; MS (ESI +ve) *m/z* 362 ([M + Na]$^+$, 30%), 340 ([M + H]$^+$, 100%); HRMS (ESI +ve TOF) calcd for $C_{19}H_{22}N_3O_3$ 340.1661, found 340.1666 ([M + H]$^+$).

1.7 Synthesis of acid scaffold 27

2-(1-Benzyl-1*H*-1,2,3-triazol-4-yl)phenol

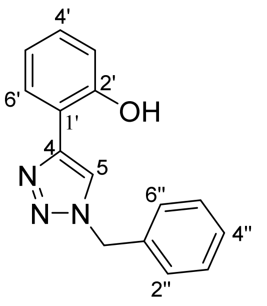

Following **General Procedure IV**, benzyl azide (1.00 g, 7.51 mmol, 0.5 M in CH_2Cl_2), 2-ethynylphenol (0.89 g, 7.51 mmol), $CuSO_4{\cdot}5H_2O$ (0.37 g, 1.50 mmol) and sodium ascorbate (0.59 g, 3.01 mmol) were stirred in *t*-BuOH (10 mL) and H2O (2.5 mL) at rt for 12 h to give the triazole product (1.51 g, 80%) as a translucent tan gum after flash chromatography over silica gel (EtOAc/*n*-hexane - 20:80). TLC (EtOAc/*n*-hexane - 40:60): R_f = 0.6; ^{1}H NMR (500 MHz, $CDCl_3$) δ 10.82 (brs, 1H, -OH), 7.72 (s, 1H, H5), 7.41-7.39 (m, 3H, H6', H3''/H5''), 7.34-7.30 (m, 3H, H2''/H4''/H6''), 7.23 (apparent t, *J* = 8.0 Hz, 1H, H4'), 7.04 (d, *J* = 8.0 Hz, 1H, H3'), 6.85 (apparent t, *J* = 8.0 Hz, 1H, H5'), 5.59 (s, 2H, C$\underline{H}_2$Ph); ^{13}C NMR (126 MHz, $CDCl_3$) δ 156.0 (C2'), 148.3 (C4), 134.2 (C1''), 129.9 (C4'), 129.4 (C3''/C5''), 129.2 (C2''/C6''), 128.3 (C6'), 125.9 (C5), 119.5 (C4''), 118.8 (C5'), 117.8 (C1'), 114.0 (C3'), 54.7 ($\underline{C}H_2$Bn); IR (neat) $\bar{\nu}_{max}$ 3021, 3138, 3033, 1617, 1580, 1478, 1449, 1356, 1277, 1247, 1235, 1213, 1157, 1071, 1056, 1033, 728, 692, 653 cm^{-1}; MS (ESI +ve) *m/z* 252 ([M + H]$^+$, 100%) ; HRMS (ESI +ve TOF) calcd for $C_{15}H_{14}N_3O$ 252.1137, found 252.1136 ([M+H]$^+$).

2-(2-(1-Benzyl-1*H*-1,2,3-triazol-4-yl)phenoxy)acetic acid (27)

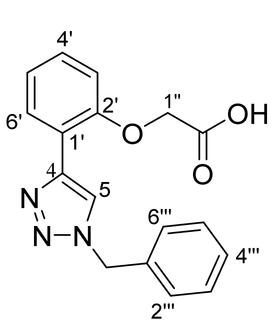

To a stirred solution of 2-(1-benzyl-1*H*-1,2,3-triazol-4-yl)phenol (1.50 g, 5.97 mmol) in dry THF (10 mL) was added K_2CO_3 (2.47 g, 17.92 mmol), followed by bromoacetic acid (1.24 g, 8.95 mmol) at rt and the reaction was stirred and heated at 80 °C for 12 h. The reaction mixture was cooled to rt, diluted with water (50 mL) and extracted with Et_2O (50 mL). The aqueous layer was cooled to 0 °C, acidified with 1 M HCl solution (25 mL) and the resultant white color precipitate was filtered and dried under vacuum to afford the acid product (1.56 g, 80%) as a white solid. M.P: 138 - 140 °C. TLC (MeOH/CH_2Cl_2 - 10:90): R_f = 0.5; ^{1}H NMR (500 MHz, $CDCl_3$) δ 8.64 (s, 1H, H5), 8.23 (d, *J* = 8.0 Hz, 1H, H6'), 7.40-7.33 (m, 5H, H2'''/H3'''/H4'''/H5'''/H6'''), 7.30 (apparent t, *J* = 8.3 Hz, 1H, H4'), 7.11 (apparent t, *J* = 8.0 Hz, 1H, H5'), 6.88 (d, *J* = 8.3 Hz, 1H, H3'), 5.60 (s, 2H, C$\underline{H}_2$Ph), 4.68 (s, 2H, H1''), COOH resonance was not observed; ^{13}C NMR (126 MHz, $CDCl_3$) δ 170.6 (C=O), 153.7 (C2'), 143.3 (C4), 134.9 (C1'''), 129.0 (C3'''/C5'''), 128.9 (C4'), 128.5 (C2'''/C6'''), 127.9 (C6'), 127.4 (C5), 124.3 (C4'''), 121.9 (C5'), 119.5 (C1'), 111.8 (C3'), 65.3 (C1''), 54.1 ($\underline{C}H_2$Ph); IR (neat) $\bar{\nu}_{max}$ 2950, 1735, 1608, 1586, 1550, 1490, 1438, 1351, 1290, 1224, 1126, 1078, 1047, 835,

756, 725, 696, 648 cm^{-1}; MS (ESI +ve) *m/z* 348 ($[M + K]^+$, 100%), 310 ($[M + H]^+$, 25%); HRMS (ESI +ve TOF) calcd for $C_{17}H_{15}N_3O_3Na$ 332.1011, found 332.1003 ($[M + Na]^+$).

1.8 Synthesis of acid scaffold 28

2-Azidophenol[35]

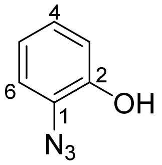

A solution of $NaNO_2$ (5.06 g, 73.39 mmol) in H_2O (5 mL) was added dropwise to a stirred suspension of 2-aminophenol (4.00 g, 36.69 mmol) in 3 N HCl solution (50 mL) at 0 °C and stirring was continued at 0 °C for 15 min. A solution of NaN_3 (4.77 g, 73.39 mmol) in H_2O (5 mL) was added at 0 °C and after 10 min the mixture was stirred at rt for 3 h. The reaction was diluted with water (50 mL) and extracted with EtOAc (2 x 100 mL). The combined extracts were washed with water (100 mL), brine (100 mL) and dried ($MgSO_4$). The solution was filtered, concentrated under vacuum and the residue was subjected to silica gel flash column chromatography (EtOAc/*n*-hexane - 10:90 → 100:0) to afford the azide (3.10 g, 60%) as a brown waxy solid. TLC (EtOAc/*n*-hexane – 20:80); $R_f = 0.5$; The spectroscopic data were found to be in agreement with those previously reported.[35] ^{1}H NMR (500 MHz, $CDCl_3$) δ 7.07-7.02 (m, 2H, H4/H6), 6.94-6.90 (m, 2H, H3/H5), 5.39 (brs, 1H, OH); ^{13}C NMR (126 MHz, $CDCl_3$) δ 147.1 (C2), 125.8 (C1), 125.8 (C4), 121.1 (C6), 118.2 (C5), 115.9 (C3); IR (neat) $\bar{\nu}_{max}$ 3341, 2121, 1586, 1576, 1496, 1297, 1206, 852, 747 cm^{-1}.

1-Azido-2-(isopentyloxy)benzene

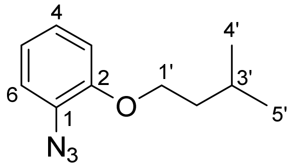

Following **General Procedure I**, 2-azidophenol (2.00 g, 14.80 mmol), K_2CO_3 (6.13 g, 44.40 mmol) and 1-bromo-3-methylbutane (2.46 g, 16.28 mmol) were stirred in dry DMF (16 mL) at rt for 48 h to give compound **88** (2.56 g, 84%) as a brown oil after flash chromatography over silica gel (EtOAc/*n*-hexane-10:90). TLC (EtOAc/*n*-hexane-20:80): $R_f = 0.6$; ^{1}H NMR (400 MHz, $CDCl_3$) δ 7.05-7.02 (m, 1H, H4), 6.92 (dd, J = 8.2, 1.8 Hz, 1H, H6), 6.87-6.84 (m, 2H, H3/H5), 4.01 (t, J = 6.7 Hz, 2H, H1'), 1.88-1.82 (m, 1H, H3'), 1.71 (q, J = 6.7 Hz, 2H, H2'), 0.96 (d, J = 6.6 Hz, 6H, H4'/H5'); ^{13}C NMR (126 MHz, $CDCl_3$) δ 152.0 (C2), 128.3 (C1), 125.7 (C6), 121.4 (C4), 120.8 (C5), 112.9 (C3), 67.4 (C1'), 37.8 (C2'), 25.1 (C3'), 22.6 (C4'/C5'); IR (neat) $\bar{\nu}_{max}$ 2956, 2871, 2107, 1592, 1495, 1451, 1386, 1304, 1239, 1149, 1042, 980, 741 cm^{-1}; MS (ESI +ve) *m/z* 178 ($[M – N_2 + H]^+$, 100%).

1-(2-(Isopentyloxy)phenyl)-5-(((tetrahydro-2*H*-pyran-2-yl)oxy)methyl)-1*H*-1,2,3-triazole

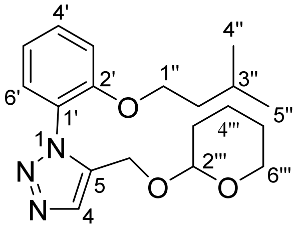

Following **General Procedure VI**, 1-azido-2-(isopentyloxy)benzene (1.30 g, 6.34 mmol) was treated with tetrahydro-2-(2-propynyloxy)-2*H*-pyran (0.98 g, 6.97 mmol) and EtMgCl (0.67 g, 7.61 mmol; 2 M in Et_2O) in dry THF (13 mL) at 50 °C for 48 h to give the triazole (1.20 g, 55%) as a pale brown oil after flash chromatography over silica gel (EtOAc/*n*-hexane - 10:90). TLC (EtOAc/*n*-hexane - 20:80): R_f

= 0.4; ^{1}H NMR (500 MHz, $CDCl_3$) δ 7.76 (s, 1H, H4), 7.48-7.44 (m, 1H, H4'), 7.39 (dd, *J* = 7.9, 1.7 Hz, 1H, H6'), 7.08-7.05 (m, 2H, H3'/H5'), 4.69 (d, *J* = 13.1 Hz, 1H, C<u>H</u>$_A$H$_B$OTHP), 4.52 (t, *J* = 3.2 Hz, 1H, H2'''), 4.46 (d, *J* = 13.1 Hz, 1H, CH$_A$<u>H</u>$_B$OTHP), 3.98 (t, *J* = 6.5 Hz, H1''), 3.56-3.52 (m, 1H, H6'''), 3.40-3.37 (m, 1H, H6'''), 1.69-1.64 (m, 1H, H3''), 1.61-1.37 (m, 8H, H2''/H3'''/H4'''/H5'''), 0.83 (d, *J* = 6.4 Hz, 6H, H4''/H5''); ^{13}C NMR (126 MHz, $CDCl_3$) δ 153.5 (C2'), 136.5 (C5), 132.8 (C4), 131.5 (C4'), 128.6 (C6'), 125.4 (C1'), 120.7 (C5'), 113.1 (C3'), 97.8 (C2'''), 67.5 (C1''), 61.6 (C6'''), 57.9 (<u>C</u>H$_A$H$_B$OTHP), 37.6 (C2''), 30.1 (C3'''), 25.3 (C5'''), 25.0 (C3''), 22.5 (C4''), 22.4 (C5''), 18.7 (C4'''); IR (neat) $\bar{\nu}_{max}$ 2952, 2871, 1602, 1509, 1465, 1387, 1350, 1287, 1261, 1247, 1123, 1056, 1033, 975, 871, 816, 755 cm^{-1}; MS (ESI +ve) *m/z* 346 ($[M + H]^+$, 100%); HRMS (ESI +ve TOF) calcd for $C_{19}H_{27}N_3O_3Na$ 368.1950, found 368.1964 ($[M + Na]^+$).

(1-(2-(Isopentyloxy)phenyl)-1*H*-1,2,3-triazol-5-yl)methanol

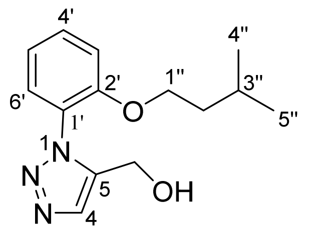

To a stirred solution of 1-(2-(isopentyloxy)phenyl)-5-(((tetrahydro-2*H*-pyran-2-yl)oxy)methyl)-1*H*-1,2,3-triazole (1.17 g, 3.39 mmol) in MeOH (15 mL) was added *p*-toluenesulfonic acid monohydrate (0.71 g, 3.73 mmol) at rt and the mixture was allowed to stir at rt for 16 h. The reaction mixture was concentrated under vacuum and the residue was subjected to silica gel flash column chromatography ($MeOH/CH_2Cl_2$ - 5:95 → 100:0) to afford the alcohol product (0.71 g, 80%) as a light brown oil. TLC ($MeOH/CH_2Cl_2$ – 10:90); R_f = 0.4; ^{1}H NMR (500 MHz, $CDCl_3$) δ 7.71 (s, 1H, H4), 7.50-7.46 (m, 1H, H4'), 7.37-7.35 (m, 1H, H6'), 7.09-7.06 (m, 2H, H3'/H5'), 4.58 (s, 2H, C<u>H</u>$_2$OH), 3.99 (t, *J* = 6.6 Hz, 2H, H1''), 3.95 (brs, 1H, OH), 1.55-1.47 (m, 3H, H2''/H3''), 0.81 (d, *J* = 6.4 Hz, 6H, H4''/H5''); ^{13}C NMR (126 MHz, $CDCl_3$) δ 153.2 (C2'), 139.4 (C5), 132.3 (C4), 131.8 (C4'), 128.7 (C6'), 125.0 (C1'), 121.2 (C5'), 113.5 (C3'), 67.9 (C1''), 53.9 (<u>C</u>H$_2$OH), 37.5 (C2''), 25.0 (C3''), 22.5 (C4''/C5''); IR (neat) $\bar{\nu}_{max}$ 2955, 2871, 1602, 1508, 1464, 1387, 1368, 1287, 1244, 1164, 1127, 1099, 1048, 1019, 978, 831, 756, 660 cm^{-1}; MS (ESI +ve) *m/z* 262 ($[M + H]^+$, 100%); HRMS (ESI +ve TOF) calcd for $C_{14}H_{20}N_3O_2$ 262.1569, found 262.1556 ($[M + H]^+$).

Ethyl 2-((1-(2-(isopentyloxy)phenyl)-1*H*-1,2,3-triazol-5-yl)methoxy)acetate

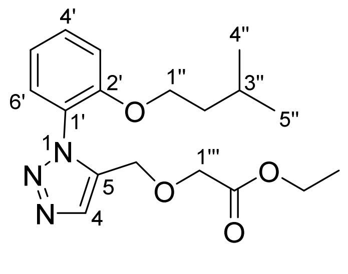

To a stirred solution of (1-(2-(isopentyloxy)phenyl)-1*H*-1,2,3-triazol-5-yl)methanol (0.25 g, 0.96 mmol) in dry THF (2 mL) at 0 °C under a nitrogen atmosphere was added NaH (0.06 g, 1.43 mmol; 60 %), followed by ethyl bromoacetate (0.21 g, 1.24 mmol). After 10 min the mixture was allowed to stir at rt for 16 h. The reaction was diluted with water (10 mL) and extracted with EtOAc (2 x 25 mL). The combined extracts were washed with water (2 x 25 mL), brine (2 x 25 mL) and dried ($MgSO_4$). The solution was filtered, concentrated under vacuum and the residue was subjected to silica gel flash column chromatography (EtOAc/*n*-hexane - 20:80 → 100:0) to afford the ester product (0.23 g, 69%) as a brown gummy solid. TLC (EtOAc/*n*-hexane – 40:60); R_f = 0.6; ^{1}H NMR (400 MHz, $CDCl_3$) δ 7.82 (s, 1H, H4), 7.50-7.47 (m, 1H, H6'), 7.41 (ddd, *J* = 7.7, 7.7, 1.6 Hz, 1H, H4'), 7.11-7.06 (m, 2H, H3'/H5'), 4.62 (s, 2H,

$C\underline{H}_2$OCOOEt), 4.16 (q, *J* = 7.1 Hz, 2H, $OC\underline{H}_2CH_3$), 3.98 (t, *J* = 6.6 Hz, 2H, H1''), 3.95 (s, 2H, H1'''), 1.56-1.48 (m, 3H, H2''/H3''), 1.24 (t, *J* = 7.1 Hz, 3H, $OCH_2C\underline{H}_3$), 0.83 (d, *J* = 6.4 Hz, 6H, H4''/H5''); ^{13}C NMR (101 MHz, $CDCl_3$) δ 170.0 (C=O), 153.6 (C2'), 135.7 (C5), 133.6 (C4), 132.0 (C4'), 128.8 (C6'), 125.2 (C1'), 121.2 (C5'), 113.4 (C3'), 67.8 (C1'''), 67.5 (C1''), 62.3 ($\underline{C}H_2O$), 61.4 ($O\underline{C}H_2CH_3$), 37.8 (C2''), 25.2 (C3''), 22.8 (C4''/C5''), 14.5 ($OCH_2\underline{C}H_3$); IR (neat) $\bar{\nu}_{max}$ 2957, 2872, 1750, 1602, 1509, 1465, 1386, 1369, 1287, 1265, 1247, 1207, 1135, 1048, 1025, 977, 756 cm^{-1}; MS (ESI +ve) *m/z* 370 ([M + Na]$^+$, 70%), 348 ([M + H]$^+$, 100%); HRMS (ESI +ve TOF) calcd for $C_{18}H_{26}N_3O_4$ 348.1923, found 348.1922 ([M + H]$^+$).

2-((1-(2-(Isopentyloxy)phenyl)-1*H*-1,2,3-triazol-5-yl)methoxy)acetic acid (28)

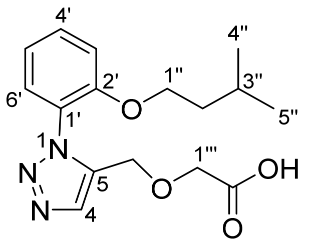

To a stirred solution of ethyl 2-((1-(2-(isopentyloxy)phenyl)-1*H*-1,2,3-triazol-5-yl)methoxy)acetate (0.23 g, 0.66 mmol) in THF:H_2O (3 mL:1mL) was added LiOH.H_2O (0.14 g, 3.31 mmol) at rt and the reaction was allowed to stir at rt for 16 h. The reaction mixture was diluted with water (5 mL), acidified with 1 M HCl (5 mL) and extracted with EtOAc (2 x 25 mL). The combined organic extracts were washed with brine (50 mL) and dried ($MgSO_4$). The solution was filtered, concentrated under vacuum to afford the acid (0.17 g, 81%) as a pale brown waxy solid. TLC (EtOAc/*n*-hexane – 100:0); R_f = 0.2; ^{1}H NMR (400 MHz, $CDCl_3$) δ 8.76 (brs, 1H, $COO\underline{H}$), 7.88 (s, 1H, H4), 7.50-7.46 (m, 1H, H4'), 7.40 (d, *J* = 7.6 Hz, 1H, H6'), 7.10-7.05 (m, 2H, H3'/H5'), 4.63 (s, 2H, H1'''), 4.00 (s, 2H, $C\underline{H}_2O$), 3.98-3.97 (m, 2H, H1''), 1.57-1.47 (m, 3H, H2''/H3''), 0.82 (d, *J* = 6.2 Hz, 6H, H4''/H5''); ^{13}C NMR (101 MHz, $CDCl_3$) δ 173.0 (C=O), 153.3 (C2'), 135.7 (C5), 132.7 (C4), 131.9 (C4'), 128.4 (C6'), 124.6 (C1'), 120.9 (C5'), 113.2 (C3'), 67.6 (C1'''), 67.0 (C1''), 61.9 ($\underline{C}$-C5), 37.5 (C2''), 24.9 (C3''), 22.4 (C4''/C5''); IR (neat) $\bar{\nu}_{max}$ 2955, 2931, 2871, 2533, 1733, 1602, 1508, 1464, 1429, 1368, 1287, 1246, 1163, 1116, 1049, 994, 846, 755, 662 cm^{-1}; MS (ESI +ve) *m/z* 342 ([M + Na]$^+$, 100%), 320 ([M + H]$^+$, 100%); HRMS (ESI +ve TOF) calcd for $C_{16}H_{22}N_3O4$ 320.1610, found 320.1603 ([M + H]$^+$).

2. Synthesis of *N*-protected β-azido-amine 29

(9*H*-Fluoren-9-yl)methyl *tert*-butyl ((*R*)-6-(((*R*)-1-azido-5-(2-((2,2-dimethyl-2,3-dihydro benzofuran-5-yl)sulfonyl)guanidino)pentan-2-yl)amino)-6-oxohexane-1,5-diyl) dicarbamate

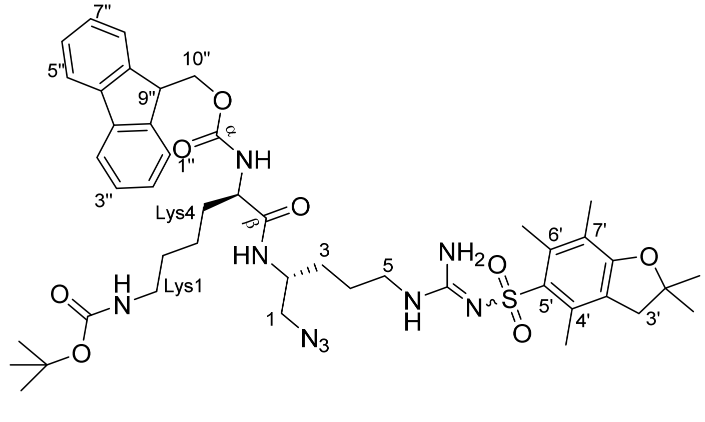

To a reaction vessel charged with azide **7**[19] (1.38 g, 3.16 mmol), Fmoc-L-Lys(Boc)-OH (1.62 g, 3.50 mmol), EDCI (0.67 g, 3.50 mmol) and HOBt (0.53 g, 3.50 mmol) was added CH_2Cl_2 (10 mL) and the mixture was stirred at rt for 12 h. The reaction mixture was concentrated and diluted with water (100 mL) and extracted with EtOAc (3 x 100 mL). The organic extracts were combined and washed with HCl (1 M – 100 mL), aqueous $NaHCO_3$ (100 mL), brine (25 mL), dried ($MgSO_4$) and concentrated to give a pale-yellow residue. This residue was purified *via* flash chromatography over silica gel (MeOH/ CH_2Cl_2 = 4:96) to afford the titled compound as an off-white foam (1.50 g, 54%). TLC (MeOH/CH_2Cl_2 – 10:90) R_f = 0.52; ^{1}H-NMR (400 MHz, $CDCl_3$) δ 7.77-7.70 (m, 2H, H4"/H5"), 7.55 (d, *J* = 7.5 Hz, 2H, H1"/H8"), 7.55 (brs, 1H, βCONH), 7.41-7.32 (m, 2H, H2"/H7"), 7.29-7.21 (m, 2H, H3"/H6"), 7.17 (brs, 1H, αCONH), 6.31-6.24 (m, 2H, NH_2 (guanidine)), 6.19-6.09 (brs, 1H, N^5-H), 4.82-4.72 (brs, 1H, LysN^1-H), 4.33 (d, *J* = 7.4 Hz, 2H, H10"), 4.25-4.07 (m, 2H, Lys5/H9"), 4.07-3.97 (m, 1H, H2), 3.41-3.23 (m, 2H, H1), 3.23-2.98 (m, 4H, H5/Lys1), 2.89 (s, 2H, H3'), 2.55 (s, 3H, C6'-C$\underline{H}_3$), 2.48 (s, 3H, C4'-C$\underline{H}_3$), 2.06 (s, 3H, C7'-C$\underline{H}_3$), 1.67 (s, 6H, C2'-C$\underline{H}_3$), 1.55-1.35 (m, 19H, H3/H4/Lys2/ Lys3/Lys4/C(C$\underline{H}_3$)$_3$); ^{13}C NMR (101 MHz, $CDCl_3$) δ 172.7 (Cβ), 158.8 (C7a'), 156.7 (Cα), 156.4 (C=N), 156.2 ($\underline{C}$OOC(CH_3)$_3$), 143.85 (C1a"or C8a"), 143.83 (C8a" or C1a"), 143.82 (C4a" or C5a"), 143.6 (C5a" or C4a"), 138.3 (C3a'), 132.8 (C6'), 132.2 (C4'), 127.8 (C3"/C6"), 127.1 (C4"/C5"), 125.0 (C2"/C7"), 124.7 (C5'), 120.0 (C1"/C8"), 117.6 (C7'), 86.4 (C2'), 79.3 ($\underline{C}$(CH_3)$_3$), 67.3 (C10"), 55.1 (Lys5), 54.8 (C1), 48.8 (C2), 47.0 (C9"), 43.2 (C3'), 40.9 (C5), 39.9 (Lys1), 31.9 (Lys2), 29.5 (Lys4), 29.3 (C3), 28.6 (C2'-($\underline{C}H_3$)$_2$), 28.4 (C($\underline{C}H_3$)$_3$), 25.5 (C4), 22.5 (Lys3), 19.3 (C6'-$\underline{C}H_3$), 17.9 (C4'-$\underline{C}H_3$), 12.5 C7'-$\underline{C}H_3$); IR (neat) $\bar{\nu}_{max}$ 3322, 2101, 1634, 1548, 1450, 1248, 1165, 1092, 739, 567 cm^{-1}; MS (ESI +ve) *m/z* 910 ([M + Na]$^+$, 80%), 888 ([M + H]$^+$, 100%),; HRMS (ESI +ve TOF) calcd for $C_{45}H_{61}N_9O_8SNa$ 910.4262, found 910.4218 ([M + Na]$^+$).

***Tert*-butyl ((*R*)-5-amino-6-(((*R*)-1-azido-5-(2-((2,2-dimethyl-2,3-dihydrobenzofuran-5-yl) sulfonyl)guanidino)pentan-2-yl)amino)-6-oxohexyl)carbamate (29)**

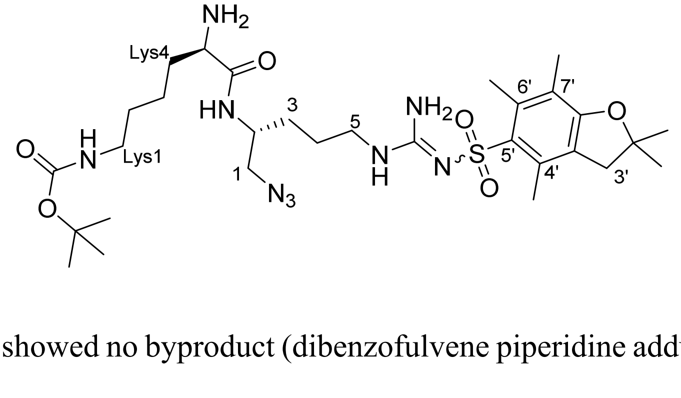

To a solution of the above mentioned Fmoc-protected amine (1.50 g, 1.69 mmol) in acetonitrile (15 mL) was added piperidine (0.25 mL, 1.5 eq.) and the reaction was stirred vigorously at rt for 12 h. The reaction mixture was diluted with MeOH (50 mL) and extracted with hexane (50 mL) multiple times until TLC analysis showed no byproduct (dibenzofulvene piperidine adduct) present in the MeOH layer. Then MeOH extract was

concentrated under reduced pressure to give **29** as an off-white foam (0.80 g, 71%). TLC (MeOH/CH_2Cl_2 – 10:90) *R*f =0.2; ^{1}H-NMR (500 MHz, $CDCl_3$) δ 7.61 (brs, 1H, N^2-H), 6.42-6.20 (m, 3H, N^5-H/ NH_2 (guanidine)), 4.82-4.72 (m, 1H, LysN^1-H), 4.12-3.99 (m, 1H, Lys5), 3.46-3.29 (m, 3H, H1/H2), 3.29-3.14 (m, 2H, H5), 3.14-3.04 (m, 2H, Lys1), 2.96 (s, 2H, C3'), 2.58 (s, 3H, C6'-C<u>H</u>3), 2.52 (s, 3H, C4'-C<u>H</u>3), 2.10 (s, 3H, C7'-C<u>H</u>3), 1.62-1.31 (m, 25H, H3/H4/Lys2/Lys3/Lys4/C(C<u>H</u>3)3/C2'-(C<u>H</u>3)2), *N*^{5}H2 resonance was not observed; ^{13}C-NMR (126 MHz, $CDCl_3$) δ 158.8 (C7a'), 156.6 (C=O), 156.4 (C=N), 138.5 (C3a'), 133.2 (C4'), 132.4 (C6'), 124.7 (C5'), 117.6 (C7'), 86.5 (C2'), 79.4 ((<u>C</u>(CH3)3), 55.1 (Lys5), 55.0 (C1), 46.9 (C2), 43.4 (C3'), 40.9 (C5), 40.4 (Lys1), 34.7 (Lys4), 30.1 (Lys2), 29.8 (C3), 28.8 (C2'-(<u>C</u>H3)2), 28.6 (C(<u>C</u>H3)3), 25.8 (C4), 22.7 (Lys3), 19.4 (C6'-<u>C</u>H3), 18.1 (C4'-<u>C</u>H3), 12.6 (C7'-<u>C</u>H3), <u>C</u>OO(C(CH3)3) resonance was not observed; IR (neat) $\bar{\nu}_{max}$ 3327, 2101, 1685, 1620, 1551, 1454, 1366, 1278, 1250, 1168, 1094, 665, 569 cm$^{-1;}$ MS (ESI +ve) *m/z* 688 ([M + Na]$^+$, 20%), 666 ([M + H]$^+$, 100%); HRMS (ESI +ve TOF) calcd for $C_{30}H_{52}N_9O_6S$ 666.3761, found 666.3741 ([M + H]$^+$).

3. Synthesis of azides, 8, 9, 19, 20 and 30-35.

(*R*)-*N*-(1-Azido-5-(2-((2,2,4,6,7-pentamethyl-2,3-dihydrobenzofuran-5-yl)sulfonyl) guanidino)pentan-2-yl)-2-(2-(4-isopentyl-1*H*-1,2,3-triazol-1-yl)phenoxy)acetamide (8)

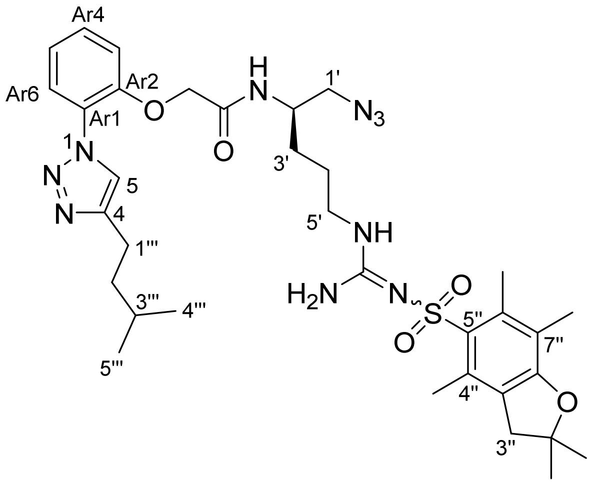

Following **General Procedure III**, the acid **5** (0.13 g, 0.46 mmol), amine **7**[14] (0.20 g, 0.46 mmol), EDCI.HCl (0.10 g, 0.50 mmol), HOBt (0.08 g, 0.50 mmol) and TEA (0.05 g, 0.46 mmol) were stirred in CH_2Cl_2 (3 mL) at rt for 14 h to give the titled product (0.19 g, 58%) as a pale-yellow solid. M.P: 234 - 236 °C. TLC (MeOH/ CH_2Cl_2 - 10:90): *R*f = 0.5; ^{1}H NMR (500 MHz, $CDCl_3$) δ 7.64 (s, 1H, H5), 7.42 (t, *J* = 7.5 Hz, 1H, Ar4), 7.39 (d, *J* = 8.0 MHz, 1H, Ar6), 7.22 (brs, 1H, CON<u>H</u>), 7.16 (t, *J* = 7.5 Hz, 1H, Ar5), 7.09 (d, *J* = 8.0 Hz, 1H, Ar3), 6.36 (brs, 1H, $N^{5'}$-H (guanidine)), 6.29 (brs, 2H, NH_2, (guanidine)), 4.61 (ABq, *J* = 9.5 Hz, 2H, OC<u>H</u>A<u>H</u>B), 4.08-3.94 (m, 1H, H2'), 3.40-3.30 (m, 2H, H1'), 3.30-3.20 (m, 2H, H5'), 2.94 (s, 2H, H3''), 2.80 (t, *J* = 8.0 Hz, 2H, H1'''), 2.57 (s, 3H, C4''-C<u>H</u>3), 2.51 (s, 3H, C6''-C<u>H</u>3), 2.07 (s, 3H, C7''-C<u>H</u>3), 1.70-1.44 (m, 7H, H2'''/H3'''/H3'/H4'), 1.44 (s, 6H, C2''-(C<u>H</u>3)2), 0.95 (d, *J* = 6.0 Hz, 6H, H5'''/H4'''); ^{13}C NMR (126 MHz, $CDCl_3$) δ 168.1 (C=O), 158.3 (C7a''), 156.3 (C=N), 151.8 (Ar2), 140.5 (C4''), 138.0 (C6''), 133.1 (C4), 133.0 (C3a''), 132.6 (C5''), 131.4 (Ar4), 127.8 (Ar6), 125.2 (C7''), 124.4 (Ar5), 122.2 (C5), 117.5 (Ar3), 113.3 (Ar1), 86.4 (C2''), 67.8 (O<u>C</u>HAHB), 55.2 (C1'), 47.6 (C2'), 43.8 (C2'''), 40.1 (C5'), 36.1 (C3''), 29.9 (C1'''), 29.6 (C3'), 26.1 (C2''-(<u>C</u>H3)2), 24.5 (C3'''), 22.5 (C4'), 22.4 (C4'''/C5'''), 20.9 (C4''-<u>C</u>H3), 18.6 (C6''-<u>C</u>H3), 12.8 (C7''-<u>C</u>H3); IR (neat) $\bar{\nu}_{max}$ 3430, 3419, 3338, 3332, 3152, 3076, 2956, 2869, 2101, 1680, 1618, 1550, 1508, 1457, 1407,

1385, 1369, 1288, 1252, 1162, 1153, 1106, 1093, 1056, 1038, 994, 978, 852, 815, 784, 760, 667, 642 cm^{-1}; MS (ESI +ve) *m/z* 731 ($[M + Na]^+$, 100%), 709 ($[M + H]^+$, 20%); HRMS (ESI +ve TOF) calcd for $C_{34}H_{48}O_5N_{10}SNa$ 731.3422, found 731.3430 ($[M + Na]^+$).

(*R*)-*N*-(1-Azido-5-(2-((2,2,4,6,7-pentamethyl-2,3-dihydrobenzofuran-5-yl)sulfonyl) guanidino)pentan-2-yl)-2-(2-(5-isopentyl-1*H*-1,2,3-triazol-1-yl)phenoxy)acetamide (9)

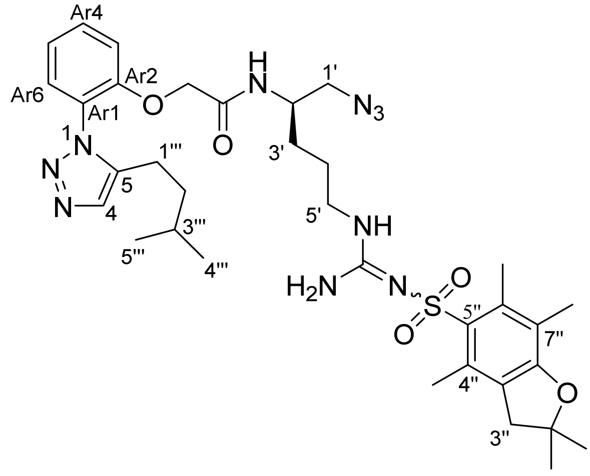

Following **General Procedure III**, the acid **6** (0.14 g, 0.48 mmol), amine **7** (0.21 g, 0.48 mmol), EDCI.HCl (0.10 g, 0.53 mmol), HOBt (0.08 g, 0.53 mmol) and TEA (0.05 g, 0.48 mmol) were stirred in CH_2Cl_2 (3 mL) at rt for 16 h to give the titled product (0.22 g, 65%) as an off-white solid. M.P: 228 - 230 °C. TLC (MeOH/ CH_2Cl_2 - 10:90): R_f = 0.5; ^1H NMR (400 MHz, $CDCl_3$) δ 7.67 (s, 1H, H4), 7.60-7.55 (m, 1H, Ar4), 7.33 (dd, *J* = 7.7, 1.5 Hz, 1H, Ar6), 7.24-7.20 (m, 1H, Ar5), 7.13 (d, *J* = 8.2 Hz, 1H, Ar3), 6.60 (brs, 1H, CON<u>H</u>), 6.36 (brs, 1H, $N^{5'}$-H (guanidine)), 6.28 (brs, 2H, NH_2 (guanidine)), 4.57 (ABq, *J* = 14.8 Hz, 2H, OC<u>H</u>$_A$<u>H</u>$_B$), 4.00-3.92 (m, 1H, H2'), 3.60-3.23 (m, 4H, H1'/H5'), 2.95 (s, 2H, H3''), 2.60 (s, 3H, C4''-C<u>H</u>$_3$), 2.58-2.54 (m, 2H, H1'''), 2.53 (s, 3H, C6''-C<u>H</u>$_3$), 2.08 (s, 3H, C7''-C<u>H</u>$_3$), 1.92-1.82 (m, 2H, H3'), 1.56-1.52 (m, 1H, H3'''), 1.52-1.42 (m, 4H, H2'''/H4'), 1.45 (s, 6H, C2''-(CH_3)$_2$), 0.83 (d, *J* = 6.3 Hz, 6H, H5'''/H4'''); ^{13}C NMR (101 MHz, $CDCl_3$) δ 167.4 (C=O), 158.5 (C7a''), 156.3 (C=N), 152.1 (Ar2), 140.7 (C5), 138.3 (C3a''), 133.3 (C4), 132.35 (C4''), 132.30 (C6''), 131.7 (C5''), 128.0 (Ar4), 124.8 (Ar6), 124.4 (C7''), 122.4 (Ar1), 117.3 (Ar5), 113.6 (Ar3), 86.2 (C2''), 67.1 (O<u>C</u>H$_A$H$_B$), 54.6 (C1'), 48.9 (C2'), 43.2 (C2'''), 40.7 (C5'), 37.0 (C3''), 29.1 (C3'), 28.6 (C2''-(<u>C</u>H$_3$)$_2$), 27.4 (C1'''), 25.3 (C4'), 22.1 (C4'''/C5'''), 22.0 (C3'''), 21.1 (C4''-<u>C</u>H$_3$), 19.2 (C6''-<u>C</u>H$_3$), 12.4 (C7''-<u>C</u>H$_3$); IR (neat) $\bar{\nu}_{max}$ 3448, 3419, 3153, 3076, 2957, 2869, 2101, 1680, 1619, 1550, 1508, 1456, 1407, 1385, 1369, 1288, 1256, 1163, 1153, 1107, 1092, 1056, 1037, 994, 978, 852, 783, 734, 669, 642 cm^{-1}; MS (ESI +ve) *m/z* 731 ($[M + Na]^+$, 100%); HRMS (ESI +ve TOF) calcd for $C_{34}H_{48}N_{10}O_5SNa$ 731.3422, found 731.3423 ($[M + Na]^+$).

(*R*)-*N*-(1-Azido-5-(2-((2,2,4,6,7-pentamethyl-2,3-dihydrobenzofuran-5-yl)sulfonyl) guanidino)pentan-2-yl)-2-((1-(4-isopentyl-1*H*-1,2,3-triazol-1-yl)naphthalen-2-yl)oxy) acetamide (19)

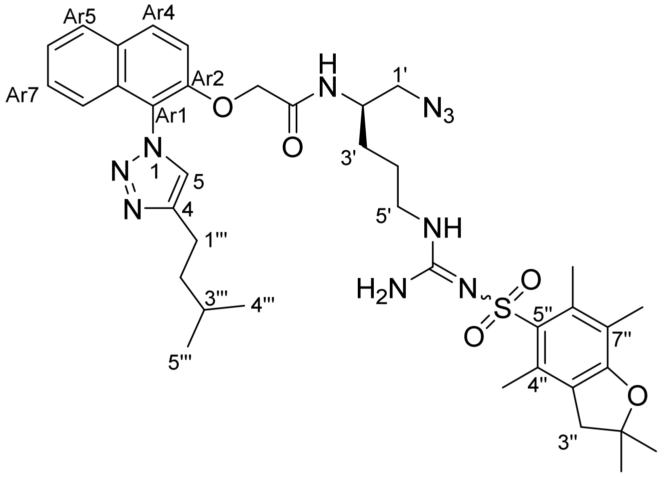

Following **General Procedure III,** the acid **17** (0.14 g, 0.41 mmol), amine **7** (0.18 g, 0.41 mmol), EDCI.HCl (0.09 g, 0.45 mmol), HOBt (0.07 g, 0.45 mmol) and TEA (0.04 g, 0.41 mmol) were stirred in CH_2Cl_2 (5 mL) at rt for 12 h to give the titled compound (0.21 g, 67%) as an off-white solid. M.P: 238 - 240 °C. TLC (MeOH/ CH_2Cl_2 - 10:90): R_f = 0.5; ^{1}H NMR (400 MHz, $CDCl_3$) δ 8.08 (d, *J* = 9.2 Hz, 1H, Ar8), 7.92 (d, *J* = 8.2 Hz, 1H, Ar5), 7.66 (s, 1H, H5), 7.56-7.47 (m, 2H, Ar6/Ar7), 7.35 (d, *J* = 8.2 Hz, 1H, Ar4), 7.19 (d, *J* = 8.2 Hz, 1H, Ar3), 6.66 (brs, 1H, CONH), 6.54-6.58 (m, 3H, $N^{5'}$-H/ NH_2 (guanidine)), 4.69 (ABq, 14.8 Hz, 2H, OCH_AH_B), 3.99-3.94 (m, 1H, H2'), 3.55-3.20 (m, 4H, H1'/H5'), 2.93 (s, 2H, H3''), 2.91-2.88 (m, 2H, H1'''), 2.58 (s, 3H, C4''-CH_3), 2.52 (s, 3H, C6''-CH_3), 2.07 (s, 3H, C7''-CH_3), 1.78-1.76 (m, 2H, H3'), 1.74-1.58 (m, 3H, H2'''/H3'''), 1.52-1.46 (m, 2H, H4'), 1.44 (s, 6H, C2''-$(CH_3)_2$), 1.00 (d, *J* = 6.2 Hz, 6H, H4'''/H5'''); ^{13}C NMR (101 MHz, $CDCl_3$) δ 167.5 (C=O), 158.5 (C7a''), 156.3 (Ar2), 149.9 (C=N), 149.1 (Ar8a), 138.3 (C3a''), 133.4 (C4''), 132.7 (C6''), 132.3 (C4), 130.3 (C5''), 129.2 (Ar4), 129.1 (Ar4a), 128.4 (C7''), 125.6 (Ar5), 124.9 (Ar7), 124.4 (Ar8), 120.6 (C5), 119.7 (Ar6), 117.3 (Ar3), 113.0 (Ar1), 86.2 (C2''), 67.6 (OCH_AH_B), 54.6 (C1'), 48.9 (C2'), 43.2 (C2'''), 40.7 (C5'), 38.4 (C3''), 29.1 (C3'), 28.5 (C2''-$(CH_3)_2$), 27.8 (C1'''), 25.3 (C4'), 23.5 (C3'''), 22.4 (C4'''/C5'''), 19.2 (C4''-CH_3), 17.9 (C6''-CH_3), 12.4 (C7''-CH_3); IR (neat) $\bar{\nu}_{max}$ 3435, 3418, 3332, 3141, 2954, 2869, 2100, 1677, 1627, 1600, 1548, 1483, 1452, 1407, 1383, 1369, 1278, 1254, 1150, 1107, 1091, 1044, 809, 782, 734, 664, 642, 619, cm^{-1}; MS (ESI +ve) *m/z* 781 ($[M + Na]^+$, 60%), 759 ($[M + H]^+$, 100%); HRMS (ESI +ve TOF) calcd for $C_{38}H_{50}N_{10}O_5SNa$ 781.3584, found 781.3620 ($[M + Na]^+$).

(*R*)-*N*-(1-Azido-5-(2-((2,2,4,6,7-pentamethyl-2,3-dihydrobenzofuran-5-yl)sulfonyl) guanidino)pentan-2-yl)-2-(2-(5-isopentyl-1*H*-1,2,3-triazol-1-yl)phenoxy)acetamide (20)

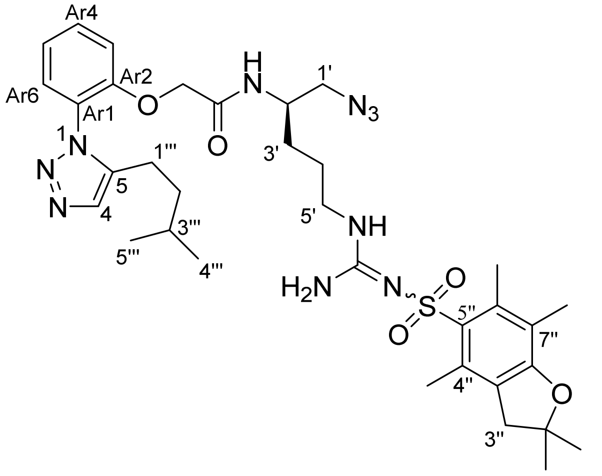

Following **General Procedure III**, the acid **18** (0.14 g, 0.48 mmol), amine **7** (0.21 g, 0.48 mmol), EDCI.HCl (0.10 g, 0.53 mmol), HOBt (0.08 g, 0.53 mmol) and TEA (0.05 g, 0.48 mmol) were stirred in CH_2Cl_2 (3 mL) at rt for 16 h to give the titled compound (0.22 g, 65%) as an off-white solid. M.P: 228 - 230 °C. TLC (MeOH/ CH_2Cl_2 - 10:90): R_f = 0.5; ^{1}H NMR (400 MHz, $CDCl_3$) δ 7.67 (s, 1H, H4), 7.60-7.55 (m, 1H, Ar4), 7.33 (dd, *J* = 7.7, 1.5 Hz, 1H, Ar6), 7.24-7.20 (m, 1H, Ar5), 7.13 (d, *J* = 8.2 Hz, 1H, Ar3), 6.60 (brs, 1H, CONH), 6.36 (brs, 1H, $N^{5'}$-H (guanidine)), 6.28 (brs, 2H, NH_2 (guanidine)), 4.57 (ABq, *J* = 14.8 Hz, 2H, $OC\underline{H}_A\underline{H}_B$), 4.00-3.92 (m, 1H, H2'), 3.60-3.23 (m, 4H, H1'/H5'), 2.95 (s, 2H, H3''), 2.60 (s, 3H, C4''-$C\underline{H}_3$), 2.58-2.54 (m, 2H, H1'''), 2.53 (s, 3H, C6''-$C\underline{H}_3$), 2.08 (s, 3H, C7''-$C\underline{H}_3$), 1.92-1.82 (m, 2H, H3'), 1.56-1.52 (m, 1H, H3'''), 1.52-1.42 (m, 4H, H2'''/H4'), 1.45 (s, 6H, C2''-$(CH_3)_2$), 0.83 (d, *J* = 6.3 Hz, 6H, H5'''/H4'''); ^{13}C NMR (101 MHz, $CDCl_3$) δ 167.4 (C=O), 158.5 (C7a''), 156.3 (C=N), 152.1 (Ar2), 140.7 (C5), 138.3 (C3a''), 133.3 (C4), 132.35 (C4''), 132.30 (C6''), 131.7 (C5''), 128.0 (Ar4), 124.8 (Ar6), 124.4 (C7''), 122.4 (Ar1), 117.3 (Ar5), 113.6 (Ar3), 86.2 (C2''), 67.1 ($O\underline{C}H_AH_B$), 54.6 (C1'), 48.9 (C2'), 43.2 (C2'''), 40.7 (C5'), 37.0 (C3''), 29.1 (C3'), 28.6 (C2''-$(\underline{C}H_3)_2$), 27.4 (C1'''), 25.3 (C4'), 22.1 (C4'''/C5'''), 22.0 (C3'''), 21.1 (C4''-$\underline{C}H_3$), 19.2 (C6''-$\underline{C}H_3$), 12.4 (C7''-$\underline{C}H_3$); IR (neat) $\bar{\nu}_{max}$ 3448, 3419, 3153, 3076, 2957, 2869, 2101, 1680, 1619, 1550, 1508, 1456, 1407, 1385, 1369, 1288, 1256, 1163, 1153, 1107, 1092, 1056, 1037, 994, 978, 852, 783, 734, 669, 642 cm^{-1}; MS (ESI +ve) *m/z* 731 ($[M + Na]^+$, 100%); HRMS (ESI +ve TOF) calcd for $C_{34}H_{48}N_{10}O_5SNa$ 731.3422, found 731.3423 ($[M + Na]^+$).

***Tert*-butyl ((*R*)-6-(((*R*)-1-azido-5-(2-((2,2,4,6,7-pentamethyl-2,3-dihydrobenzofuran-5-yl) sulfonyl)guanidino)pentan-2-yl)amino)-5-(2-(2-(4-isopentyl-1*H*-1,2,3-triazol-1-yl)phenoxy) acetamido)-6-oxohexyl)carbamate (30)**

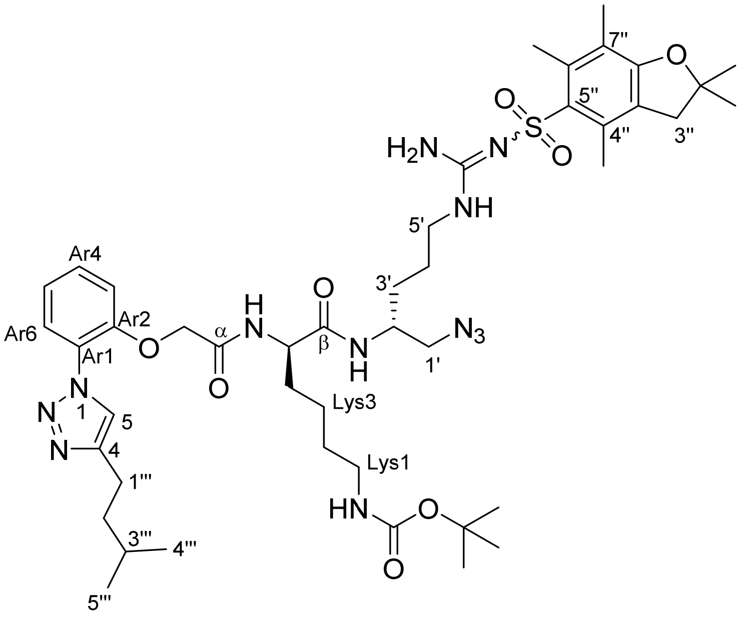

Following **General Procedure III**, the acid **5** (0.10 g, 0.34 mmol), amine **29** (0.23 g, 0.34 mmol), EDCI.HCl (0.07 g, 0.38 mmol), HOBt (0.06 g, 0.38 mmol) and TEA (0.03 g, 0.34 mmol) were stirred in CH_2Cl_2 (5 mL) at rt for 16 h to give the titled compound (0.25 g, 78%) as an off-white solid. M.P: 256 - 258 °C. TLC (MeOH/ CH_2Cl_2 - 10:90): R_f = 0.5; ^{1}H NMR (400 MHz, $CDCl_3$) δ 7.78-7.74 (m, 1H, Ar6), 7.70 (s, 1H, H5), 7.54-7.36 (m, 3H, Ar4/Ar5/βCONH), 7.20-7.04 (m, 2H, αCONH/Ar3), 6.40-6.10 (m, 3H, $N^{5'}$-H/NH_2 (guanidine)), 5.04-4.96 (m, 1H, LysN^1-H), 4.65-4.56 (m, 2H, OCH_AH_B), 4.44-4.38 (m, 1H, Lys5), 4.04-3.96 (m, 1H, H2'), 3.32-3.24 (m, 2H, H1'), 3.22-2.98 (m, 4H, H5'/Lys1), 2.92 (s, 2H, H3''), 2.84-2.74 (m, 2H, H1'''), 2.54 (s, 3H, C4''-CH_3), 2.48 (s, 3H, C6''-CH_3), 2.06 (s, 3H, C7''-CH_3), 1.90-1.70 (m, 2H, Lys4), 1.64-1.58 (m, 4H, H4'/Lys2), 1.56-1.26 (m, 7H, H3'/ Lys3/H2'''/ H3'''), 1.46 (s, 9H, $C(CH_3)_3$), 1.44 (s, 6H, C2''-$(CH_3)_2$), 0.33 (d, J = 5.5 Hz, 6H, H4'''/H5'''); ^{13}C NMR (101 MHz, $CDCl_3$) δ 168.5 (βC=O), 168.1 (αC=O), 158.1 (C7a''), 156.4 (C=N), 150.4 ($COO(CH_3)_3$), 145.9 (Ar2), 138.4 (C4), 132.4 (C4''), 131.1 (C6''), 130.8 (C3a''), 129.6 (C5''), 126.6 (Ar4), 126.4 (C7''), 124.7 (Ar6), 122.6 (Ar5), 117.5 (C5), 117.3 (Ar3), 111.3 (Ar1), 86.4 (C2''), 79.1 ($C(CH_3)_3$), 67.4 (OCH_AH_B), 54.9 (Lys5), 53.7 (C1'), 43.4 (C2'), 40.9 (C2'''), 38.5 (C5'), 31.5 (C3''), 31.3 (Lys1), 29.8 (Lys4), 29.4 (C3'), 28.7 (Lys2), 28.5 (C1'''), 27.85 ($C(CH_3)_3$), 27.83 (C2''-$(CH_3)_2$), 25.5 (C4'), 23.5 (C3'''), 22.9 (Lys3), 22.5 (C4'''/C5'''), 19.3 (C4''-CH_3), 18.0 (C6''-CH_3), 12.5 (C7''-CH_3); IR (neat) $\bar{\nu}_{max}$ 3316, 3071, 2953, 2932, 2868, 2100, 1662, 1619, 1602, 1546, 1508, 1459, 1420, 1389, 1365, 1250, 1195, 1165, 1109, 1056, 1026, 982, 913, 834, 816, 755, 665, 644, 622 cm^{-1}; MS (ESI +ve) *m/z* 959 ($[M + Na]^+$, 100%); HRMS (ESI +ve TOF) calcd for $C_{45}H_{69}N_{12}O_8S$ 937.5082, found 937.5098 ($[M+H]^+$).

***Tert*-butyl ((*R*)-6-(((*R*)-1-azido-5-(2-((2,2,4,6,7-pentamethyl-2,3-dihydrobenzofuran-5-yl)sulfonyl)guanidino)pentan-2-yl)amino)-5-(2-(2-(5-isopentyl-1*H*-1,2,3-triazol-1-yl) phenoxy)acetamido)-6-oxohexyl)carbamate (31)**

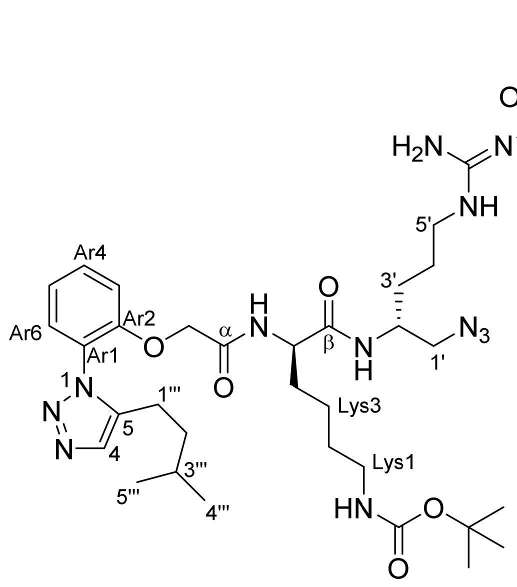

Following **General Procedure III**, the acid **6** (0.03 g, 0.10 mmol), amine **29** (0.07 g, 0.10 mmol), EDCI.HCl (0.02 g, 0.11 mmol), HOBt (0.02 g, 0.11 mmol) and TEA (0.01 g, 0.10 mmol) were stirred in CH_2Cl_2 (2 mL) at rt for 12 h to give the titled compound (0.07 g, 74%) as an off-white solid. M.P: 242 - 244 °C. TLC (MeOH/ CH_2Cl_2 - 10:90): R_f = 0.5; ^{1}H NMR (400 MHz, $CDCl_3$) δ 7.60 (s, 1H, H4), 7.54 (t, *J* = 8.1 Hz, 1H, Ar4), 7.52 (brs, 1H, βCONH), 7.34-7.14 (m, 3H, Ar6/Ar5/Ar3), 7.04-6.98 (brs, 1H, αCONH), 6.12-6.08 (m, 3H, $N^{5'}$-H/NH_2 (guanidine)), 5.20-5.14 (m, 1H, LysN^1-H), 4.65-4.53 (m, 2H, OCH_AH_B), 4.38-4.31 (m, 1H, Lys5), 3.99-3.96 (m, 1H, H2'), 3.30-3.24 (m, 2H, H1'), 3.10-3.01 (m, 4H, H5'/Lys1), 2.94 (s, 2H, H3''), 2.56 (s, 3H, C4''-CH_3), 2.49 (s, 3H, C6''-CH_3), 2.49-2.45 (m, 2H, H1'''), 2.07 (s, 3H, C7''-CH_3), 1.80-1.70 (m, 2H, Lys4), 1.54-1.40 (m, 9H, H3'/H4'/Lys2/H2'''/H3'''), 1.45 (s, 9H, $C(CH_3)_3$), 1.42 (s, 6H, C2''-$(CH_3)_2$), 1.30-1.26 (m, 2H, Lys3), 0.83 (d, *J* = 6.4 Hz, 3H, H4'''), 0.81 (d, *J* = 6.4 Hz, 3H, H5'''); ^{13}C NMR (101 MHz, $CDCl_3$) δ 171.8 (βC=O), 168.4 (αC=O), 158.6 (C7a''), 156.2 (C=N), 152.1 ($COOC(CH_3)_3$), 140.2 (Ar2), 138.2 (C5), 133.1 (C4''), 132.1 (C6''), 132.0 (C3a''), 131.9 (C5''), 128.1 (C4), 128.0 (Ar4), 124.9 (C7''), 124.5 (Ar6), 122.4 (Ar1), 117.3 (Ar5), 113.8 (Ar3), 86.3 (C2''), 78.9 ($C(CH_3)_3$), 67.3 (OCH_AH_B), 54.6 (Lys5), 53.4 (C1'), 53.4 (C2'), 48.7 (C2'''), 43.2 (C5'), 40.6 (C3''), 40.1 (Lys1), 37.0 (Lys4), 31.5 (C3'), 29.2 (Lys2), 28.5 ($C(CH_3)_3$), 28.4 (C2''-$(CH_3)_2$), 28.2 (C1'''), 27.4 (C4'), 25.4 (C3'''), 22.8 (C4'''), 22.7 (C5'''), 22.1 (Lys3), 19.2 (C4''-CH_3), 17.9 (C6''-CH_3), 12.4 (C7''-CH_3); IR (neat) $\bar{\nu}_{max}$ 3319, 3073, 2953, 2933, 2869, 2101, 1662, 1602, 1546, 1508, 1459, 1420, 1390, 1366, 1251, 1195, 1165, 1109, 1057, 1024, 984, 913, 882, 833, 755, 665, 622, 580 cm^{-1}; MS (ESI +ve) *m/z* 959 ([M + Na]$^+$, 100%), 937 ([M + H]$^+$, 60%); HRMS (ESI +ve TOF) calcd for $C_{45}H_{69}N_{12}O_8S$ 937.5076, found 937.5077 ([M + H]$^+$).

***Tert*-butyl ((*R*)-6-(((*R*)-1-azido-5-(2-((2,2,4,6,7-pentamethyl-2,3-dihydrobenzofuran-5-yl)sulfonyl)guanidino)pentan-2-yl)amino)-5-(2-((1-(4-isopentyl-1*H*-1,2,3-triazol-1-yl) naphthalen-2-yl)oxy)acetamido)-6-oxohexyl)carbamate (32)**

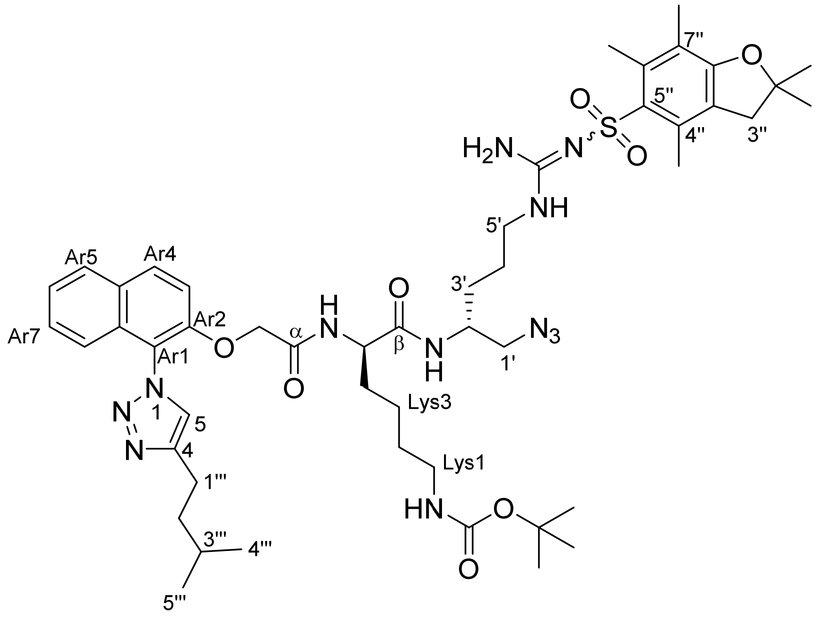

Following **General Procedure III**, the acid **17** (0.12 g, 0.35 mmol), amine **31** (0.24 g, 0.35 mmol), EDCI.HCl (0.08 g, 0.39 mmol), HOBt (0.06 g, 0.39 mmol) and TEA (0.03 g, 0.35 mmol) were stirred in CH_2Cl_2 (5 mL) at rt for 12 h to give the titled compound (0.22 g, 64%) as an off-white solid. M.P: 236 – 238 °C. TLC (MeOH/ CH_2Cl_2 - 10:90): R_f = 0.5; ^{1}H NMR (400 MHz, $CDCl_3$) δ 8.05 (d, *J* = 8.5 Hz, 1H, Ar8), 7.89 (d, *J* = 8.5 Hz, 1H, Ar5), 7.65 (s, 1H, H5), 7.54-7.47 (m, 2H, Ar4/βCONH), 7.37 (d, *J* = 8.5 Hz, 1H, Ar7), 7.26-7.19 (m, 2H, Ar6/Ar3), 6.85 (brs, 1H, αCONH), 6.36-6.08 (m, 3H, $N^{5'}$-H/NH_2 (guanidine)), 5.00 (brs, 1H, $LysN^1$-H), 4.69 (ABq, *J* = 16.5 Hz, 2H, OCH_AH_B), 4.42-4.36 (m, 1H, Lys5), 4.02-3.96 (m, 1H, H2'), 3.44-2.94 (m, 6H, H1'/H5'/Lys1), 2.88 (s, 2H, H3"), 2.88-2.84 (m, 2H, H1'''), 2.55 (s, 3H, C4"-CH_3), 2.48 (s, 3H, C6"-CH_3), 2.06 (s, 3H, C7"-CH_3), 2.00-1.86 (m, 4H, H4'/Lys4), 1.84-1.60 (m, 7H, H3'/Lys3/H2'''/H3'''), 1.44 (s, 6H, C2"(CH_3)$_2$), 1.39 (s, 9H, C(CH_3)$_3$), 1.32-1.22 (m, 2H, Lys2), 0.9 (d, *J* = 5.0 Hz, 6H, H4'''/H5'''); ^{13}C NMR (101 MHz, $CDCl_3$) δ 171.9 (βC=O), 168.1 (αC=O), 158.7 (C7a"), 156.4 (C=N), 150.1 (Ar2), 149.1 (COOC(CH_3)$_3$), 138.4 (Ar8a), 133.4 (C4), 132.6 (C4"), 132.46 (C6"), 132.44 (C3a"), 130.6 (C5"), 129.4 (Ar4), 129.1 (Ar4a), 128.48 (C7"), 128.47 (Ar5), 125.7 (Ar7), 124.7 (Ar8), 121.1 (C5), 120.3 (Ar6), 117.5 (Ar3), 113.8 (Ar1), 86.5 (C2"), 79.2 (C(CH_3)$_3$), 68.0 (OCH_AH_B), 54.8 (Lys5), 53.6 (C1'), 43.4 (C2'), 40.8 (C2'''), 40.2 (C5'), 38.6 (C3"), 38.5 (Lys1), 31.79 (Lys4), 31.74 (C3'), 29.4 (Lys2), 28.7 (C2"-(CH_3)$_2$), 28.6 ((CH_3)$_3$), 28.0 (C1'''), 25.5 (C4'), 23.8 (C3'''), 22.8 (C4'''/C5'''), 22.6 (Lys3), 19.4 (C4"-CH_3), 18.1 (C6"-CH_3), 12.6 (C7"-CH_3); IR (neat) $\bar{\nu}_{max}$ 3405, 3317, 3415, 3057, 2953, 2868, 2100, 1664, 1631, 1600, 1546, 1514, 1484, 1452, 1406, 1390, 1366, 1265, 1247, 1165, 1106, 1090, 1044, 994, 970, 852, 781, 733, 661, 641 cm^{-1}; MS (ESI +ve) *m/z* 987 ([M + H]$^+$, 100%); HRMS (ESI +ve TOF) calcd for $C_{49}H_{71}N_{12}O_8S$ 987.5239, found 987.5272 ([M + H]$^+$).

***Tert*-Butyl ((5*R*)-6-(((*R*)-1-azido-5-(2-((2,2,4,6,7-pentamethyl-2,3-dihydrobenzofuran-5-yl) sulfonyl)guanidino)pentan-2-yl)amino)-5-(2-((1-(5-isopentyl-1*H*-1,2,3-triazol-1-yl) naphthalen-2-yl)oxy)acetamido)-6-oxohexyl)carbamate (33)**

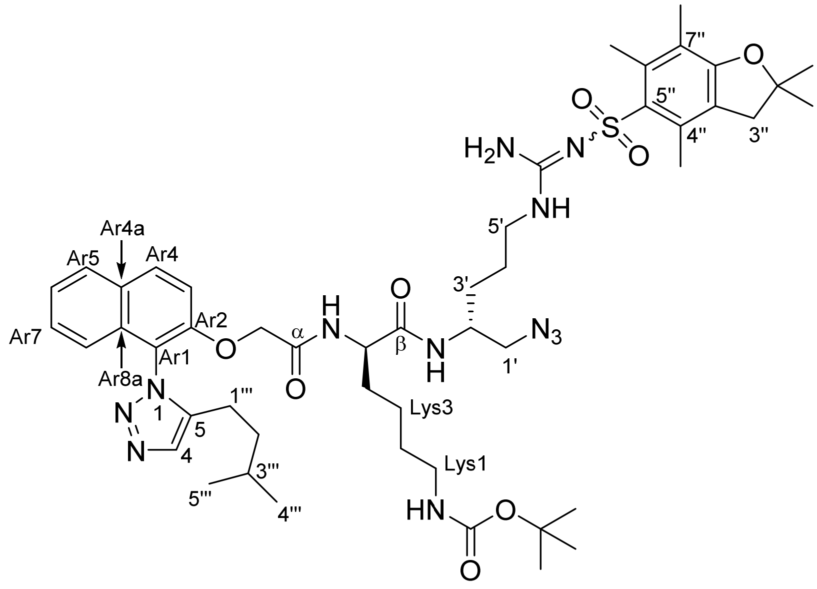

Following **General Procedure III**, the acid **18** (0.13 g, 0.38 mmol), amine **29** (0.26 g, 0.38 mmol), EDCI.HCl (0.08 g, 0.42 mmol), HOBt (0.06 g, 0.42 mmol) and TEA (0.04 g, 0.38 mmol) were stirred in CH_2Cl_2 (5 mL) at rt for 16 h to give the titled compound (0.25 g, 67%) as a pale brown solid. M.P: 242 - 244 °C. TLC (MeOH/ CH_2Cl_2 - 10:90): R_f = 0.5; ^{1}H NMR (400 MHz, $CDCl_3$) δ 8.14-8.09 (m, 1H, Ar8), 7.93 (d, J = 7.3 Hz, 1H, Ar5), 7.83 (s, 1H, H4), 7.66-7.36 (m, 3H, Ar4/βCONH/Ar7), 7.08-6.95 (m, 2H, Ar6/Ar3), 6.54-6.52 (m, 1H, αCONH), 6.34-6.24 (m, 3H, $N^{5'}$-H/NH_2 (guanidine)), 5.13 (brs, 1H, LysN^1-H), 4.64 (ABq, J = 16.5 Hz, 2H, OCH_AH_B), 4.42-4.36 (m, 1H, Lys5), 4.02-3.96 (m, 1H, H2'), 3.30-2.94 (m, 6H, H1'/H5'/Lys1), 2.94 (s, 2H, H3''), 2.57 (s, 3H, C4''-CH_3), 2.51 (s, 3H, C6''-CH_3), 2.48-2.32 (m, 2H, H1'''), 2.08 (s, 3H, C7''-CH_3), 1.82-1.76 (m, 2H, H4'), 1.58-1.16 (m, 11H, H3'/Lys2/Lys3/Lys4/H2'''/H3'''), 1.44 (s, 6H, C2''-$(CH_3)_2$), 1.40 (s, 9H, $C(CH_3)_3$), 0.74 (d, J = 5.5 Hz, 3H, H4'''), 0.71 (d, J = 6.0 Hz, 3H, H5'''); ^{13}C NMR (101 MHz, $CDCl_3$, major rotamer) δ 171.4 (βC=O), 167.6 (αC=O), 158.5 (C7a''), 156.2 (C=N), 150.6 (Ar2), 150.5 ($COOC(CH_3)_3$), 141.4 (C5), 138.2 (Ar8a), 133.2 (C4), 133.0 (C4''), 132.2 (C6''), 130.6 (C3a''), 129.3 (C5''), 129.0 (Ar4), 128.8 (Ar4a), 128.4 (C7''), 125.6 (Ar5), 124.5 (Ar7), 120.8 (Ar8), 118.2 (Ar6), 117.3 (Ar3), 113.5 (Ar1), 86.3 (C2''), 78.9 ($C(CH_3)_3$), 67.7 (OCH_AH_B), 54.5 (Lys5), 53.4 (C1'), 43.2 (C2'), 40.7 (C2'''), 40.2 (C5'), 36.7 (C3''), 31.8 (Lys1), 29.3 (Lys4), 29.1 (C3'), 28.5 (Lys2), 28.4 (C2''-$(CH_3)_2$), 28.4 ($(CH_3)_3$), 27.2 (C1'''), 25.4 (C4'), 22.9 (C3'''), 22.0 (C4'''/C5'''), 20.9 (Lys3), 19.2 (C4''-CH_3), 17.9 (C6''-CH_3), 12.4 (C7''-CH_3); IR (neat) $\bar{\nu}_{max}$ 3406, 3317, 3144, 3057, 2953, 2868, 2100, 1664, 1631, 1600, 1545, 1514, 1484, 1452, 1406, 1390, 1366, 1265, 1165, 1151, 1090, 1044, 1024, 994, 913, 852, 807, 781, 733, 660, 641, 566 cm^{-1}; MS (ESI +ve) m/z 988 ([M + H]$^+$, 100%); HRMS (ESI +ve TOF) calcd for $C_{49}H_{72}N_{12}O_8S$ 988.5317, found 988.5337 ([M+H]$^+$).

***Tert*-butyl ((*R*)-6-(((*R*)-1-azido-5-(2-((2,2,4,6,7-pentamethyl-2,3-dihydrobenzofuran-5-yl) sulfonyl)guanidino)pentan-2-yl)amino)-5-(2-(2-(1-benzyl-1*H*-1,2,3-triazol-4-yl)phenoxy) acetamido)-6-oxohexyl)carbamate (34)**

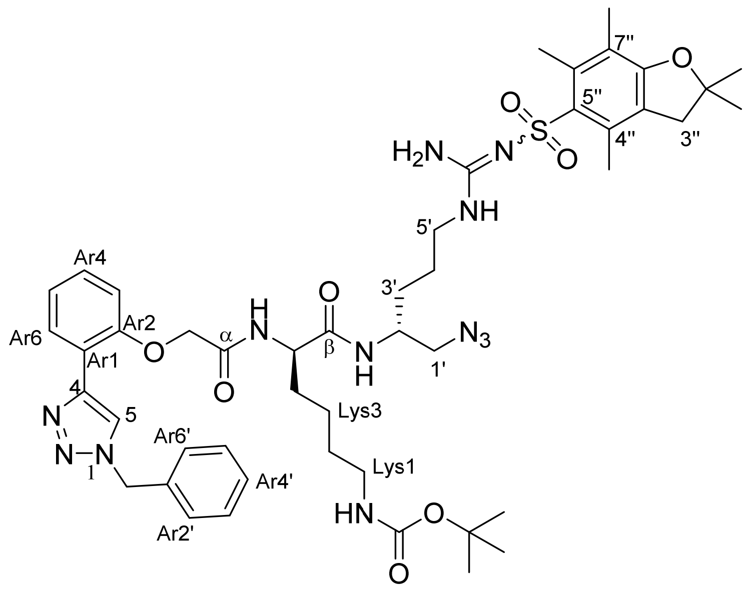

Following **General Procedure III**, the acid **27** (0.09 g, 0.30 mmol), amine **29** (0.20 g, 0.30 mmol), EDCI.HCl (0.06 g, 0.33 mmol), HOBt (0.05 g, 0.33 mmol) and TEA (0.03 g, 0.30 mmol) were stirred in CH_2Cl_2 (3 mL) at rt for 16 h to give the titled compound (0.14 g, 49%) as a white solid. M.P: 244 - 246 °C. TLC (MeOH/ CH_2Cl_2 - 10:90): R_f = 0.5; ^{1}H NMR (400 MHz, $CDCl_3$) δ 8.98 (brs, 1H, βCONH), 7.93 (s, 1H, H5), 7.69-7.67 (m, 1H, αCONH), 7.36-7.26 (m, 7H, Ar6/Ar4/Ar2'/Ar3'/Ar4'/Ar5'/Ar6'), 7.11-7.10 (m, 1H, LysN^1-H), 7.04 (t, J = 7.5 Hz, 1H, Ar5), 6.92 (d, J = 8.5 Hz, 1H, Ar3), 6.28-6.20 (m, 2H, NH_2 (guanidine)), 6.18-6.10 (m, 1H, N$^{5'}$-H (guanidine)), 5.61 (ABq, J = 15.0 Hz, 2H, $OC\underline{H}_A\underline{H}_B$), 4.80-4.72 (m, 1H, Lys5), 4.64-4.60 (m, 1H, H1'), 4.64 (s, 2H, $C\underline{H}_2$Ph), 4.56-4.46 (m, 1H, H1'), 4.08-3.98 (m, 1H, H2'), 3.32-3.20 (m, 2H, H5'), 3.20-3.06 (m, 2H, Lys1), 2.92 (s, 2H, H3''), 2.56 (s, 3H, C4''-$C\underline{H}_3$), 2.49 (s, 3H, C6''-$C\underline{H}_3$), 2.07 (s. 3H, C7''-$C\underline{H}_3$), 2.02-1.80 (m, 6H, Lys4/H3'/H4'), 1.48-1.42 (m, 4H, Lys2/Lys3), 1.44 (s, 9H, $C(C\underline{H}_3)_3$), 1.40 (s, 6H, C2''-$(C\underline{H}_3)_2$); ^{13}C NMR (101 MHz, $CDCl_3$) δ 172.6 (βC=O), 169.5 (αC=O), 158.8 (C7a''), 156.4 (C=N), 156.3 (Ar2), 154.1 ($\underline{C}OOC(CH_3)_3$), 145.0 (C4), 138.4 (Ar1'), 135.0 (C4''), 133.2 (C6''), 132.4 (C3a''), 129.9 (Ar4), 129.3 (C5''), 129.2 (C5), 129.1 (C7''), 129.0 (Ar3'), 128.8 (Ar5'), 128.2 (Ar2'), 124.7 (Ar6'), 122.5 (Ar4'), 122.4 (Ar6), 119.6 (Ar5), 117.6 (Ar3), 113.3 (Ar1), 86.5 (C2''), 79.2 ($\underline{C}(CH_3)_3$), 67.8 ($O\underline{C}H_AH_B$), 60.6 (Lys5), 55.0 ($\underline{C}H_2$Ph), 54.3 (C1'), 53.9 (C2'), 53.6 (C5'), 43.4 (C3''), 40.9 (Lys1), 40.2 (Lys4), 31.6 (C3'), 29.5 (Lys2), 28.7 ($C(\underline{C}H_3)_3$), 28.6 (C2''-$(\underline{C}H_3)_2$), 25.6 (C4'), 23.1 (Lys3), 19.4 (C4''-$\underline{C}H_3$), 18.1 (C6''-$\underline{C}H_3$), 12.6 (C7''-$\underline{C}H_3$); IR (neat) $\bar{\nu}_{max}$ 3426, 3322, 3156, 3065, 2973, 2932, 2865, 2100, 1660, 1619, 1547, 1489, 1454, 1406, 1392, 1366, 1247, 1165, 1105, 1092, 1042, 994, 976, 941, 904, 852, 783, 756, 698, 642 cm$^{-1;}$ MS (ESI +ve) *m/z* 979 ([M + Na]$^+$, 100%); HRMS (ESI +ve TOF) calcd for $C_{47}H_{64}N_{12}O_8SNa$ 979.4583, found 979.4587 ([M + Na]$^+$).

***Tert*-Butyl ((*R*)-6-(((*R*)-1-azido-5-(2-((2,2,4,6,7-pentamethyl-2,3-dihydrobenzofuran-5-yl) sulfonyl)guanidino)pentan-2-yl)amino)-5-(2-((1-(2-(isopentyloxy)phenyl)-1*H*-1,2,3-triazol-5-yl)methoxy)acetamido)-6-oxohexyl)carbamate (35)**

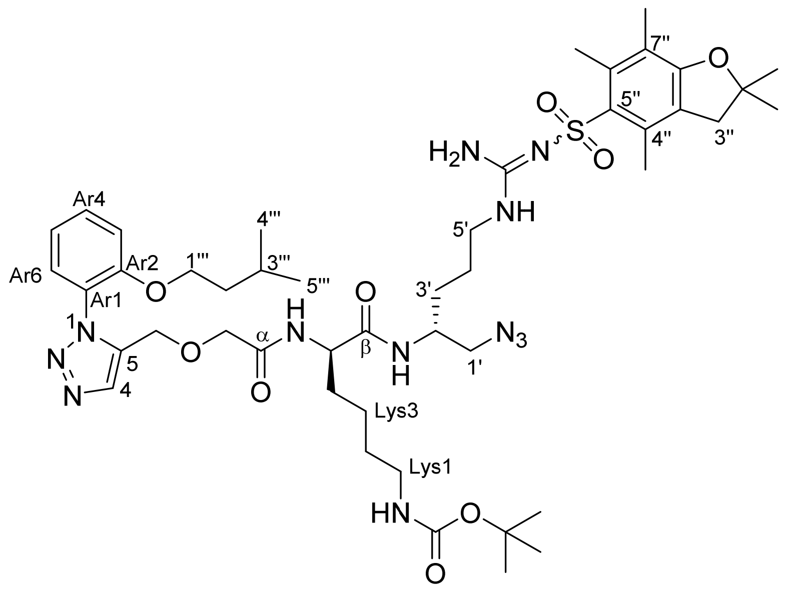

Following **General Procedure III**, the acid **28** (0.12 g, 0.37 mmol), amine **29** (0.25 g, 0.37 mmol), EDCI.HCl (0.08 g, 0.41 mmol), HOBt (0.06 g, 0.41 mmol) and TEA (0.04 g, 0.37 mmol) were stirred in CH_2Cl_2 (3 mL) at rt for 16 h to give the titled compound (0.25 g, 70%) as a pale brown solid. M.P: 220 - 222 °C. TLC (MeOH/ CH_2Cl_2 - 10:90): R_f = 0.6; ^{1}H NMR (400 MHz, $CDCl_3$) δ 7.82 (s, 1H, H4), 7.49 (ddd, *J* = 8.2, 1.6 Hz, 1H, Ar4), 7.40 (dd, *J* = 8.0, 1.6 Hz, 1H, Ar6), 7.11-7.0 (m, 3H, Ar5/Ar3/βCON$\underline{H}$), 6.97 (brs, 1H, αCON$\underline{H}$), 6.34-6.24 (m, 3H, $N^{5'}$-H/NH_2 (guanidine)), 4.75 (t, *J* = 5.8 Hz, 1H, LysN^1-H), 4.63-4.55 (m, 2H, C5-C$\underline{H}_2$), 4.35 (t, *J* = 6.5 Hz, 1H, Lys5), 4.01-3.74 (m, 3H, H2'/H1'''), 3.89-3.74 (m, 2H, OC$\underline{H}_A\underline{H}_B$), 3.38-3.28 (m, 2H, H1'), 3.44-3.40 (m, 2H, H5'), 3.07-3.00 (m, 2H, Lys1), 2.94 (s, 2H, H3''), 2.56 (s, 3H, C4''-C$\underline{H}_3$), 2.50 (s, 3H, C6''-CH_3), 2.08 (s. 3H, C7''-C$\underline{H}_3$), 1.85-1.80 (m, 2H, Lys4), 1.64-1.45 (m, 9H, H2'''/H3'''/Lys2/H3'/H4'), 1.45 (s, 6H, C2''(C$\underline{H}_3$)$_2$), 1.41 (s, 9H, C(C$\underline{H}_3$)$_3$), 1.36-1.20 (m, 2H, Lys3), 0.82 (d, *J* = 6.3 Hz, 6H, H4'''/H5'''); ^{13}C NMR (101 MHz, $CDCl_3$) δ 171.7 (βC=O), 169.1 (αC=O), 158.7 (C7a''), 156.2 (C=N), 153.1 ($\underline{C}$OO(CH_3)$_3$), 138.3 (Ar2), 135.0 (C5), 133.1 (C4''), 132.9 (C6''), 132.2 (C3a''), 131.8 (C4), 128.3 (C5''), 124.9 (C7''), 124.8 (Ar4), 124.6 (Ar6), 121.1 (Ar1), 117.4 (Ar5), 113.3 (Ar3), 86.4 (C2''), 79.2 ($\underline{C}$(CH_3)$_3$), 69.1 (O$\underline{C}H_AH_B$), 67.7 (C1'''), 62.1 (C5-$\underline{C}H_2$), 54.6 (Lys5), 52.7 (C1'), 43.2 (C2'), 40.7 (C5'), 39.9 (C3''), 37.5 (Lys1), 32.0 (C2'''), 29.7 (Lys4), 29.3 (Lys2), 29.2 (C3'), 28.6 (C2''-($\underline{C}H_3$)$_2$), 28.4 (($\underline{C}H_3$)$_3$), 25.5 (C4'), 24.9 (C3'''), 22.5 (C4'''/C5'''), 22.4 (Lys3), 19.2 (C4''-$\underline{C}H_3$), 17.9 (C6''-$\underline{C}H_3$), 12.4 (C7''-$\underline{C}H_3$); IR (neat) $\bar{\nu}_{max}$ 3325, 3147, 3076, 2963, 2930, 2869, 2100, 1661, 1620, 1549, 1510, 1459, 1404, 1390, 1367, 1286, 1250, 1166, 1107, 1050, 1036, 996, 978, 852, 808, 783, 757, 734, 663, 642, 569 cm^{-1}; MS (ESI +ve) *m/z* 989 ([M + Na]$^+$, 100%); HRMS (ESI +ve TOF) calcd for $C_{46}H_{70}N_{12}O_9SNa$ 989.5007, found 989.5029 ([M + Na]$^+$).

4. Synthesis of final derivatives 10– 16, 21-26 and 36-50.

(*R*)-*N*-(1-(4-Cyclohexyl-1*H*-1,2,3-triazol-1-yl)-5-guanidinopentan-2-yl)-2-(2-(4-isopentyl-1*H*-1,2,3-triazol-1-yl)phenoxy)acetamide hydrochloride (10)

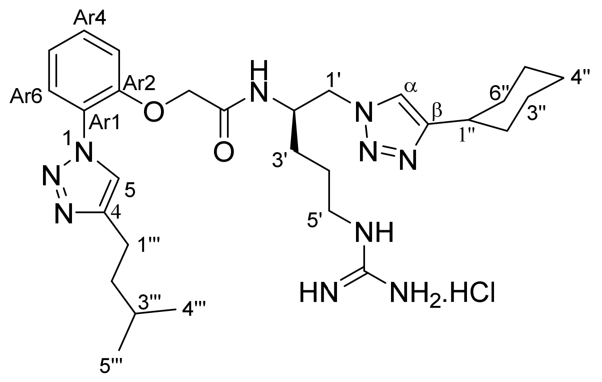

Following **General Procedure IV**, azide **8** (0.07 g, 0.09 mmol), cyclohexylacetylene (0.03 g, 0.27 mmol), $CuSO_4{\cdot}5H_2O$ (0.005 g, 0.02 mmol) and sodium ascorbate (0.008 g, 0.04 mmol) were stirred in *t*-BuOH (2 mL) and H_2O (0.5 mL) for 16 h to give the intermediate triazole as an off-white solid after flash chromatography over silica gel ($MeOH/CH_2Cl_2$ – 0:100 → 3:97). Following **General Procedure VII**, the intermediate trizole (0.06 g, 0.07 mmol) was dissolved in CH_2Cl_2 (2 mL), treated with H_2O (0.03 g, 1.46 mmol) and CF_3COOH (1 mL) followed by work-up with ethereal HCl (2 mL) to give the amine salt **10** (0.03 g, 55% over two steps) as a pale brown solid that rapidly transitioned to a sticky gum. $[\alpha]_D^{23}$ +68.1 (*c* 0.0047, MeOH). ^1H NMR (400 MHz, CD_3OD) δ 8.46 (s, 1H, H5), 8.36 (s, 1H, Hα), 7.65 (apparent t, *J* = 7.5 Hz, 1H, Ar4), 7.55 (d, *J* = 7.3 Hz, 1H, Ar6), 7.28-7.18 (m, 2H, Ar5/Ar3), 4.84-4.78 (m, 1H, H1'), 4.72-4.58 (m, 3H, $OC\underline{H}_A\underline{H}_B$/H1'), 4.50-4.40 (m, 1H, H2'), 3.30-3.20 (m, 2H, H5'), 2.90-2.80 (m, 2H, H1'''), 2.04-1.94 (m, 2H, H3'), 1.86-1.50 (m, 11H, H4'/H2'''/H3'''/ H1''/H2''/H3''/H4''/H5''/ H6''), 1.48-1.20 (m, 5H, H2''/H3''/H4''/H5''/H6''), 0.99 (d, *J* = 5.0 Hz, 6H, H4'''/H5'''); ^{13}C NMR (101 MHz, CD_3OD) δ 170.9 (C=O), 158.7 (C=N), 152.1 (Ar2), 147.9 (Cβ), 132.8 (C4), 127.7 (Ar4), 127.3 (Ar6), 125.9 (C5), 125.3 (Cα), 123.8 (Ar5), 122.3 (Ar3), 115.7 (Ar1), 69.1 (C1'), 67.0 ($O\underline{C}H_AH_B$), 57.2 (C2'), 50.7 (C2'''), 42.1 (C5'), 39.6 (C1''), 33.3 (C2''), 33.2 (C6''), 29.8 (C3'), 29.0 (C1'''), 26.8 (C4''), 26.7 (C3''), 26.5 (C5''), 24.1 (C3'''), 23.6 (C4'), 22.9 (C4'''/C5'''); IR (neat) $\bar{\nu}_{max}$ 3339, 3276, 3180, 2931, 2857, 2481, 1906, 1670, 1603, 1549, 1507, 1465, 1451, 1386, 1368, 1289, 1230, 1168, 1130, 1048, 981, 937, 855, 821, 800, 761, 701, 669 cm^{-1}; MS (ESI +ve) *m/z* 565 ($[M - HCl + H]^+$, 100%); HRMS (ESI +ve TOF) calcd for $C_{29}H_{45}N_{10}O_2$ 565.3727, found 565.3731 ($[M - HCl + H]^+$).

(*R*)-*N*-(1-(4-Cyclopentyl-1*H*-1,2,3-triazol-1-yl)-5-guanidinopentan-2-yl)-2-(2-(4-isopentyl-1*H*-1,2,3-triazol-1-yl)phenoxy)acetamide hydrochloride (11)

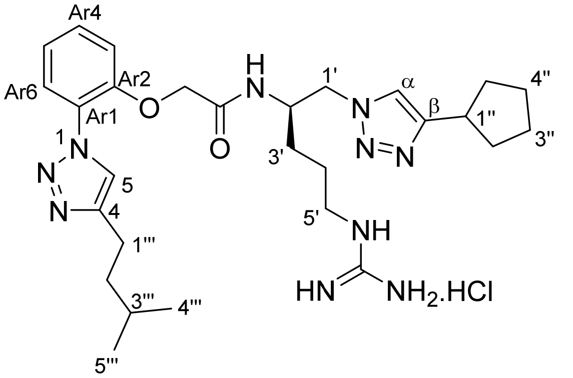

Following **General Procedure IV**, azide **8** (0.07 g, 0.09 mmol), cyclopentylacetylene (0.03 g, 0.29 mmol), $CuSO_4{\cdot}5H_2O$ (0.005 g, 0.02 mmol) and sodium ascorbate (0.008 g, 0.04 mmol) were stirred in *t*-BuOH (2 mL) and H_2O (0.5 mL) for 16 h to give the intermediate triazole as an off-white solid after flash chromatography over silica gel (MeOH/CH_2Cl_2 – 0:100 → 3:97). Following **General Procedure VII**, the intermediate **135** (0.06 g, 0.07 mmol) was dissolved in CH_2Cl_2 (2 mL), treated with H_2O (0.03 g, 1.4 mmol) and CF_3COOH (1 mL) followed by work-up with ethereal HCl (2 mL) to give the amine salt **23** (0.025 g, 57% over two steps) as a brown solid that rapidly transitioned to a sticky gum. $[\alpha]_D^{23}$ +58.0 (*c* 0.0052, MeOH). ^{1}H NMR (400 MHz, CD_3OD) δ 8.58 (s, 1H, H5), 8.45 (brs, 1H, Hα), 7.68-7.58 (m, 1H, Ar6), 7.56-7.46 (m, 1H, Ar4), 7.22-7.06 (m, 2H, Ar5/Ar3), 4.70-4.56 (m, 2H, OC$\underline{H_A}\underline{H_B}$), 4.50-4.40 (m, 1H, H1'), 4.38-4.30 (m, 1H, H1'), 4.20-4.12 (m, 1H, H2'), 3.14-3.02 (m, 3H, H5'/H1''), 2.76-2.62 (m, 2H, H1'''), 1.96-1.84 (m, 2H, H3'), 1.68-1.38 (m, 13H, H4'/H2'''/H3'''/H2''/ H3''/H4''/H5''), 0.90 (d, *J* = 5.6 Hz, 6H, H4'''/ H5'''); ^{13}C NMR (101 MHz, CD_3OD) δ 167.2 (C=O), 156.7 (C=N), 150.6 (Ar2), 149.6 (Cβ), 146.7 (C4), 129.9 (Ar4), 125.9 (Ar6), 125.1 (Cα), 123.5 (C5), 121.6 (Ar5), 121.5 (Ar3), 113.9 (Ar1), 69.7 (C1'), 58.4 (O$\underline{C}H_AH_B$), 50.7 (C2'), 42.3 (C2'''), 39.2 (C5'), 36.1 (C1''), 33.9 (C3'), 29.8 (C1'''), 28.9 (C2''/C5''), 26.7 (C3''/C4''), 26.2 (C3'''), 24.0 (C4'), 22.9 (C4'''/C5'''); IR (neat) $\bar{\nu}_{max}$ 3344, 3276, 3184, 2957, 2871, 2478, 2110, 1895, 1722, 1668, 1603, 1551, 1505, 1469, 1453, 1385, 1368, 1288, 1229, 1168, 1131, 1080, 1050, 993, 939, 854, 801, 760, 653, 586 cm^{-1}; MS (ESI +ve) *m/z* 551 ([M – HCl + H]$^+$, 100%), 276 ([M – HCl + H]$^{2+}$, 80%); HRMS (ESI +ve TOF) calcd for $C_{28}H_{43}N_{10}O_2$ 551.3565, found 551.3572 ([M – HCl + H]$^+$).

(*R*)-*N*-(1-(4-(4-Fluorophenyl)-1*H*-1,2,3-triazol-1-yl)-5-guanidinopentan-2-yl)-2-(2-(4-isopentyl-1*H*-1,2,3-triazol-1-yl)phenoxy)acetamide hydrochloride (12)

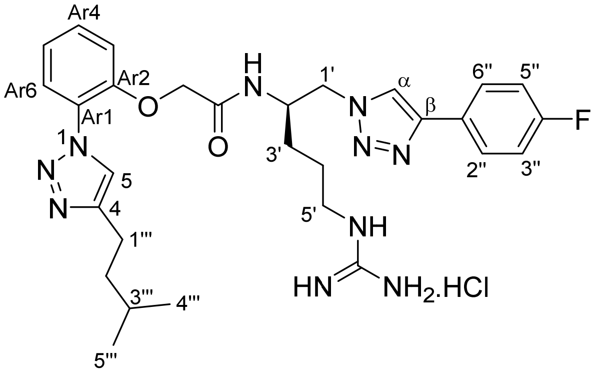

Following **General Procedure IV**, azide **8** (0.06 g, 0.09 mmol), 1-ethynyl-4-fluoro benzene (0.03 g, 0.27 mmol), $CuSO_4{\cdot}5H_2O$ (0.005 g, 0.02 mmol) and sodium ascorbate (0.008 g, 0.04 mmol) were stirred in *t*-BuOH (2 mL) and H_2O (0.5 mL) for 16 h to give the intermediate triazole as an off-white solid after flash chromatography over silica gel (MeOH/CH_2Cl_2 – 0:100 → 3:97). Following **General Procedure VII**, the intermediate triazole (0.06 g, 0.07 mmol) was dissolved in CH_2Cl_2 (2 mL), treated with H_2O (0.03 g, 1.4 mmol) and CF_3COOH (1 mL) followed by work-up with ethereal HCl (2 mL) to give the amine salt **12** (0.03 g, 54% over two steps) as an off-white solid that rapidly transitioned to a sticky gum. $[\alpha]_D^{23}$ +74.2 (*c* 0.0051, MeOH). ^{1}H NMR (400 MHz, DMSO-d_6) δ 8.51 (s, 1H, H5), 8.39 (brs, 2H, N^5-H/C=N<u>H</u>), 7.86-7.78 (m, 3H, N<u>H2</u>.<u>H</u>Cl), 7.59 (d, *J* = 7.5 Hz, 1H, Ar6), 7.50-7.38 (m, 1H, CON<u>H</u>), 7.32-7.22 (m, 4H, Hα/Ar4/H2"/H6"), 7.08 (t, *J* = 7.5 Hz, 1H, Ar3), 7.02 (d, $J_{H\text{-}F}$ = 8.5 Hz, 2H, H3"/H5"), 7.02-6.90 (m, 1H, Ar5), 4.65 (ABq, *J* = 15.8 Hz, 2H, OC<u>H</u>$_A$<u>H</u>$_B$), 4.54-4.40 (m, 2H, H1'), 4.28-4.18 (m, 1H, H2'), 3.12-3.02 (m, 2H, H5'), 2.64 (t, *J* = 7.5 Hz, 2H, H1'''), 1.62-1.40 (m, 7H, H3'/H4'/H2'''/H3'''), 0.88 (d, *J* = 6.5 Hz, 6H, H4'''/H5'''); ^{13}C NMR (101 MHz, DMSO-d_6) δ 167.5 (C=O), 161.7 (d, $J_{C\text{-}F}$ = 196.4 Hz, C4"), 156.9 (C=N), 149.7 (Ar2), 146.9 (Cβ), 145.3 (C4), 130.0 (Ar4), 127.3 (d, $J_{C\text{-}F}$ = 3.0 Hz, C1"), 127.2 (Ar6), 127.1 (Cα), 126.1 (C5), 125.3 (Ar5), 123.7 (Ar3), 121.8 (d, $J_{C\text{-}F}$ = 22.3 Hz, C2"/C6"), 115.8 (d, $J_{C\text{-}F}$ = 16.9 Hz, C3"/C5"), 114.0 (Ar1), 67.3 (C1'), 64.9 (O<u>C</u>H$_A$H$_B$), 52.7 (C2'), 48.7 (C2'''), 40.3 (C5'), 28.3 (C3'), 27.1 (C1'''), 25.0 (C3'''), 22.9 (C4'), 22.3 (C4'''/C5'''); IR (neat) $\bar{\nu}_{max}$ 3342, 3273, 3182, 2957, 2871, 2489, 1895, 1667, 1559, 1501, 1469, 1414, 1387, 1367, 1286, 1230, 1191, 1166, 1132, 1081, 1049, 993, 975, 941, 843, 816, 759, 666, 654, 600, 585 cm^{-1}; MS (ESI +ve) *m/z* 577 ([M – HCl + H]$^+$, 100%), 289 ([M – HCl + H]$^{2+}$, 20%); HRMS (ESI +ve TOF) calcd for $C_{29}H_{37}N_{10}O_2ClF$ 611.2768, found 611.2771 ([M + H]$^+$).

(*R*)-*N*-(1-(4-Benzyl-1*H*-1,2,3-triazol-1-yl)-5-guanidinopentan-2-yl)-2-(2-(4-isopentyl-1*H*-1,2,3-triazol-1-yl)phenoxy)acetamide hydrochloride (13)

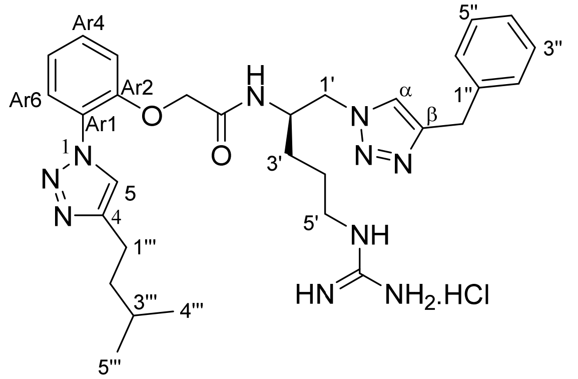

Following **General Procedure IV**, azide **8** (0.07 g, 0.09 mmol), 3-phenyl-1-propyne (0.03 g, 0.27 mmol), $CuSO_4 \cdot 5H_2O$ (0.005 g, 0.02 mmol) and sodium ascorbate (0.008 g, 0.04 mmol) were stirred in *t*-BuOH (2 mL) and H_2O (0.5 mL) for 16 h to give the intermediate triazole as an off-white gum after flash chromatography over silica gel ($MeOH/CH_2Cl_2$ – 0:100 → 3:97). Following **General Procedure VII**, the intermediate triazole (0.06 g, 0.07 mmol) was dissolved in CH_2Cl_2 (2 mL), treated with H_2O (0.03 g, 1.4 mmol) and CF_3COOH (1 mL) followed by work-up with ethereal HCl (2 mL) to give the amine salt **13** (0.029 g, 53% over two steps) as a brown solid that rapidly transitioned to a sticky gum. $[\alpha]_D^{23}$ +68.1 (*c* 0.0042, MeOH). ^{1}H NMR (400 MHz, CD_3OD) δ 8.76 (brs, 1H, H5), 8.38 (brs, 1H, Hα), 7.68 (d, *J* = 7.5 Hz, 1H, Ar6), 7.55 (t, *J* = 8.0 Hz, 1H, Ar4), 7.32-7.20 (m, 6H, Ar5/H2''/H3''/H4''/H5''/H6''), 7.15 (d, *J* = 8.0 Hz, 1H, Ar3), 4.82-4.76 (m, 1H, $OC\underline{H}_AH_B$), 4.68-4.56 (m, 3H, $OCH_A\underline{H}_B$/H1'), 4.48-4.40 (m, 1H, H2'), 4.15 (s, 2H, $C\underline{H}_2Ph$), 3.28-3.16 (m, 2H, H5'), 2.92-2.84 (m, 2H, H1'''), 1.84-1.50 (m, 7H, H3'/H4'/H2'''/H3'''), 0.99 (d, *J* = 4.0 Hz, 6H, H4'''/H5'''); ^{13}C NMR (101 MHz, CD_3OD) δ 170.4 (C=O), 158.6 (C=N), 151.8 (Ar2), 137.6 (Cβ), 133.1 (C4), 131.1 (C1''), 130.8 (Ar4), 130.0 (C2''/C6''), 129.8 (Ar6), 129.7 (C3''/C5''), 128.34 (C5), 128.33 (C4''), 127.2 (Cα), 123.64 (Ar5), 123.63 (Ar3), 115.4 (Ar1), 68.7 (C1'), 66.8 ($O\underline{C}H_AH_B$), 56.8 (C2'), 50.5 (C2'''), 41.9 (C5'), 38.8 (C3'), 29.5 ($\underline{C}H_2Ph$), 28.8 (C1'''), 26.2 (C3'''), 23.4 (C4'), 22.6 (C4'''/C5'''); IR (neat) $\bar{\nu}_{max}$ 3338, 3274, 3182, 3073, 2957, 2934, 2870, 2470, 1902, 1669, 1603, 1549, 1507, 1465, 1386, 1368, 1288, 1230, 1167, 1132, 1049, 983, 936, 855, 799, 760, 721, 697, 669, 644 cm^{-1}; MS (ESI +ve) *m/z* 573 ([M – HCl + H]$^+$, 100%); HRMS (ESI +ve TOF) calcd for $C_{30}H_{41}N_{10}O_2$ 573.3414, found 573.3434 ([M – HCl + H]$^+$).

(*R*)-*N*-(1-(4-Cyclohexyl-1*H*-1,2,3-triazol-1-yl)-5-guanidinopentan-2-yl)-2-(2-(5-isopentyl-1*H*-1,2,3-triazol-1-yl)phenoxy)acetamide hydrochloride (14)

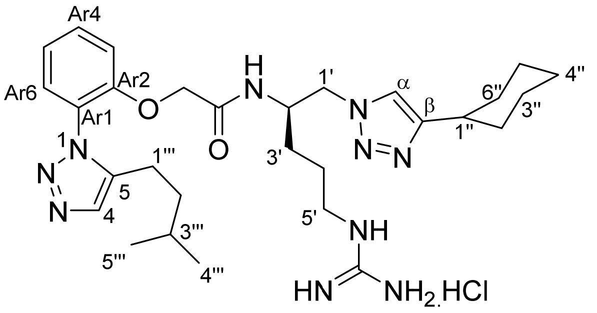

Following **General Procedure IV**, azide **9** (0.07 g, 0.09 mmol), cyclohexylacetylene (0.03 g, 0.27 mmol), $CuSO_4{\cdot}5H_2O$ (0.005 g, 0.02 mmol) and sodium ascorbate (0.008 g, 0.04 mmol) were stirred in *t*-BuOH (2 mL) and H_2O (0.5 mL) for 16 h to give the intermediate triazole as a pale brown solid after flash chromatography over silica gel (MeOH/CH_2Cl_2 – 0:100 → 5:95). Following **General Procedure VII**, the intermediate triazole (0.06 g, 0.07 mmol) was dissolved in CH_2Cl_2 (2 mL), treated with H_2O (0.03 g, 1.46 mmol) and CF_3COOH (1 mL) followed by work-up with ethereal HCl (3 mL) to give the amine salt **14** (0.03 g, 56% over two steps) as a pale brown solid that rapidly transitioned to a sticky gum. $[\alpha]_D^{23}$ +59.7 (*c* 0.0058, MeOH). ^{1}H NMR (400 MHz, CD_3OD) δ 7.98 (d, *J* = 8.4 Hz, 1H, Ar4), 7.75 (s, 1H, H4), 7.74 (s, 1H, Hα), 7.63-7.57 (m, 1H, Ar6), 7.48 (dd, *J* = 7.7, 1.6 Hz, 1H, Ar5), 7.09 (d, *J* = 8.4 Hz, 1H, Ar3), 4.59 (ABq, *J* = 15.1 Hz, 2H, OC$\underline{H}_A\underline{H}_B$), 4.51-4.46 (m, 1H, H1'), 4.40-4.33 (m, 1H, H1'), 4.26-4.16 (m, 1H, H2'), 3.18-3.08 (m, 2H, H1'''), 2.72-2.62 (m, 2H, H5'), 2.00-1.88 (m, 2H, H3'), 1.84-1.68 (m, 3H, H1''/H2'''), 1.62-1.18 (m, 13H, H3'''/H2''/H3''/ H4''/H5''/H6''/H4'), 0.82 (d, *J* = 6.4 Hz, 6H, H4'''/H5'''); ^{13}C NMR (101 MHz, CD_3OD) δ 167.4 (C=O), 157.3 (C=N), 152.8 (Ar2), 152.0 (Cβ), 140.4 (C5), 133.4 (C4), 132.0 (Ar4), 131.6 (Ar6), 128.9 (Ar1), 125.0 (Cα), 121.8 (Ar5), 114.0 (Ar3), 67.1 (O$\underline{C}H_AH_B$), 53.0 (C1'), 49.0 (C2'), 36.8 (C5'), 34.8 (C2'''), 32.89 (C1''), 32.85 (C2''/C6''), 28.9 (C3'), 27.3 (C3'''), 26.0 (C1'''), 25.9 (C4''), 25.3 (C3''/C5''), 22.4 (C4'), 20.7 (C4'''/C5'''); IR (neat) $\bar{\nu}_{max}$ 3339, 3276, 3185, 2932, 2858, 2476, 1907, 1670, 1603, 1549, 1507, 1465, 1451, 1386, 1368, 1289, 1230, 1169, 1131, 1048, 983, 954, 938, 855, 822, 800, 762, 700, 669, 643 cm^{-1}; MS (ESI +ve) *m/z* 565 ([M – HCl + H]$^+$, 100%); HRMS (ESI +ve TOF) calcd for $C_{29}H_{45}N_{10}O_2$ 565.3727, found 565.3734 ([M + H]$^+$).

(*R*)-*N*-(1-(4-(Cyclohexylmethyl)-1*H*-1,2,3-triazol-1-yl)-5-guanidinopentan-2-yl)-2-(2-(5-isopentyl-1*H*-1,2,3-triazol-1-yl)phenoxy)acetamide hydrochloride (15)

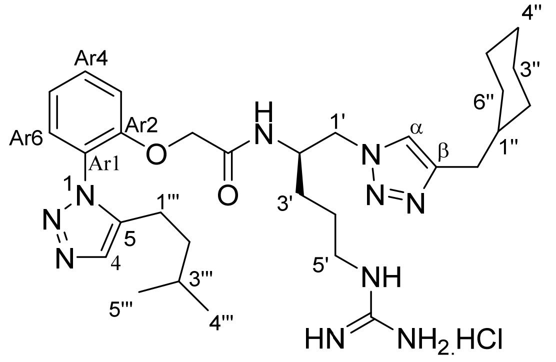

Following **General Procedure IV**, azide **9** (0.07 g, 0.09 mmol), 3-cyclohexyl-1-propyne (0.03 g, 0.27 mmol), $CuSO_4{\cdot}5H_2O$ (0.005 g, 0.02 mmol) and sodium ascorbate (0.008 g, 0.04 mmol) were stirred in *t*-BuOH (2 mL) and H_2O (0.5 mL) for 16 h to give the intermediate triazole as a white solid after flash chromatography over silica gel ($MeOH/CH_2Cl_2$ – 0:100 → 5:95). Following **General Procedure VII**, the intermediate triazole (0.07 g, 0.08 mmol) was dissolved in CH_2Cl_2 (2 mL), treated with H_2O (0.03 g, 1.6 mmol) and CF_3COOH (1 mL) followed by work-up with ethereal HCl (3 mL) to give the amine salt **15** (0.03 g, 54% over two steps) as a pale-yellow solid that rapidly transitioned to a sticky gum. $[\alpha]_D^{23}$ +62.1 (*c* 0.0051, MeOH). ^{1}H NMR (400 MHz, CD_3OD) δ 8.27 (brs, 1H, H4), 7.95 (brs, 1H, Hα), 7.63 (t, *J* = 7.4 Hz, 1H, Ar4), 7.47 (d, *J* = 7.4 Hz, 1H, Ar6), 7.30-7.22 (m, 2H, Ar5/Ar3), 4.79-4.69 (m, 1H, H1'), 4.63-4.49 (m, 3H, OC<u>H</u>$_A$<u>H</u>$_B$/H1'), 4.47-4.39 (m, 1H, H2'), 3.22-3.10 (m, 2H, H1'''), 2.77-2.61 (m, 4H, βC-C<u>H</u>$_2$/H5'), 1.84-1.36 (m, 13H, H3'/H4'/H2'''/H3'''/H1''/H2''/H3''/H4''/H5''/H6''), 1.34-1.09 (m, 3H, H2''/H4''/H6''), 1.04-0.90 (m, 2H, H3''/H5''), 0.81 (d, *J* = 6.1 Hz, 6H, H4'''/H5'''); ^{13}C NMR (101 MHz, CD_3OD) δ 168.7 (C=O), 157.2 (C=N), 152.5 (Ar2), 145.6 (Cβ), 140.8 (C5), 140.1 (Ar4), 132.6 (C4), 131.7 (Ar6), 128.5 (Ar1), 123.8 (Cα), 122.2 (Ar5), 113.9 (Ar3), 67.1 (O<u>C</u>H$_A$H$_B$), 56.1 (C1'), 49.1 (C2'), 40.5 (C5'), 37.3 (C2'''), 36.6 (C1''), 32.2 (βC-<u>C</u>H$_2$), 30.6 (C2''/C6''), 28.2 (C3'), 27.2 (C3'''), 25.7 (C1'''), 25.6 (C4''), 24.9 (C3''/C5''), 21.1 (C4'), 20.9 (C4'''/C5'''); IR (neat) $\bar{\nu}_{max}$ 3345, 3275, 3186, 2953, 2927, 2854, 2482, 1909, 1670, 1603, 1550, 1507, 1465, 1450, 1386, 1368, 1288, 1229, 1168, 1130, 1084, 1049, 983, 958, 935, 854, 761, 669, 648, 585 cm^{-1}; MS (ESI +ve) *m/z* 579 ([M – HCl + H]$^+$, 100%); HRMS (ESI +ve TOF) calcd for $C_{30}H_{47}N_{10}O_2$ 579.3877, found 579.3876 ([M – HCl + H]$^+$).

(*R*)-*N*-(1-(4-Benzyl-1*H*-1,2,3-triazol-1-yl)-5-guanidinopentan-2-yl)-2-(2-(5-isopentyl-1*H*-1,2,3-triazol-1-yl)phenoxy)acetamide hydrochloride (16)

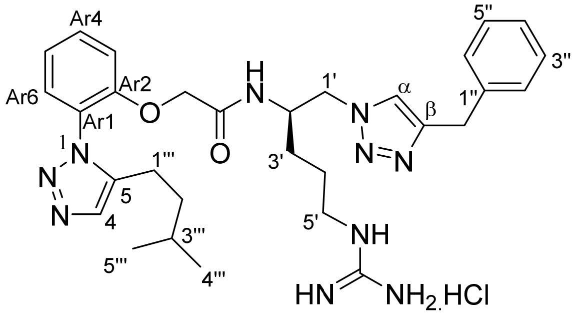

Following **General Procedure IV**, azide **9** (0.07 g, 0.09 mmol), 3-phenyl-1-propyne (0.03 g, 0.27 mmol), $CuSO_4{\cdot}5H_2O$ (0.005 g, 0.02 mmol) and sodium ascorbate (0.008 g, 0.04 mmol) were stirred in *t*-BuOH (2 mL) and H_2O (0.5 mL) for 16 h to give the intermediate triazole as an off-white gum after flash chromatography over silica gel (MeOH/CH_2Cl_2 – 0:100 → 5:95). Following **General Procedure VII**, the intermediate triazole (0.07 g, 0.08 mmol) was dissolved in CH_2Cl_2 (2 mL), treated with H_2O (0.03 g, 1.6 mmol) and CF_3COOH (1 mL) followed by work-up with ethereal HCl (3 mL) to give the amine salt **16** (0.03 g, 55% over two steps) as a pale brown solid that rapidly transitioned to a sticky gum. $[\alpha]_D^{23}$ +73.6 (*c* 0.0046, MeOH). ^{1}H NMR (400 MHz, CD_3OD) δ 8.22 (brs, 1H, H4), 8.11 (brs, 1H, Hα), 7.62 (t, *J* = 7.7 Hz, 1H, Ar4), 7.48 (d, *J* = 7.3 Hz, 1H, Ar6), 7.31-7.22 (m, 6H, Ar5/H2''/H3''/H4''/H5''/H6''), 7.17 (d, *J* = 7.7 Hz, 1H, Ar3), 4.80-4.70 (m, 1H, H1'), 4.52-4.44 (m, 3H, OC<u>H</u>$_A$<u>H</u>$_B$/H1'), 4.42-4.32 (m, 1H, H2'), 4.16 (s, 2H, C<u>H</u>$_2$Ph), 3.24-3.12 (m, 2H, H1'''), 2.72-2.62 (m, 2H, H5'), 1.83-1.42 (m, 7H, H3'/H4'/ H2'''/H3'''), 0.81 (d, *J* = 5.6 Hz, 6H, H4'''/H5'''); ^{13}C NMR (101 MHz, CD_3OD) δ 167.4 (C=O), 157.3 (C=N), 152.8 (Ar2), 146.1 (Cβ), 140.4 (C5), 139.9 (C1''), 132.0 (C4), 131.6 (Ar4), 128.9 (C2''/C6''), 128.8 (C3''/C5''), 128.7 (Ar6), 126.5 (C4''), 125.0 (Ar1), 123.6 (Cα), 121.9 (Ar5), 114.0 (Ar3), 67.0 (O<u>C</u>H$_A$H$_B$), 55.3 (C1'), 52.9 (C2'), 40.5 (C5'), 36.7 (C2'''), 31.6 (<u>C</u>H$_2$Ph), 28.9 (C3'), 27.3 (C3'''), 25.3 (C1'''), 22.4 (C4'), 20.7 (C4'''/C5'''); IR (neat) $\bar{\nu}_{max}$ 3341, 3276, 3181, 3088, 3075, 2956, 2934, 2870, 2470, 1914, 1669, 1603, 1549, 1507, 1465, 1385, 1368, 1288, 1229, 1168, 1131, 1049, 982, 936, 855, 799, 759, 721, 698, 669, 645 cm^{-1}; MS (ESI +ve) *m/z* 573 ([M – HCl + H]$^+$, 100%); HRMS (ESI +ve TOF) calcd for $C_{30}H_{41}N_{10}O_2$ 573.3414, found 573.3420 ([M – HCl + H]$^+$).

(*R*)-*N*-(1-(4-Cyclohexyl-1*H*-1,2,3-triazol-1-yl)-5-guanidinopentan-2-yl)-2-((1-(4-isopentyl-1*H*-1,2,3-triazol-1-yl)naphthalen-2-yl)oxy)acetamide hydrochloride (21)

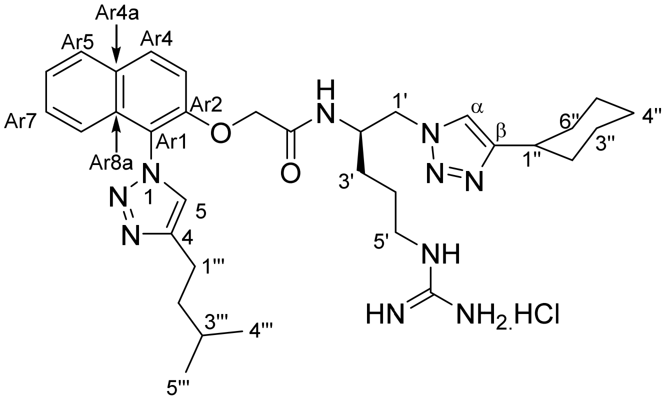

Following **General Procedure IV**, azide **19** (0.07 g, 0.09 mmol), cyclohexyl acetylene (0.03 g, 0.27 mmol), $CuSO_4{\cdot}5H_2O$ (0.005 g, 0.01 mmol) and sodium ascorbate (0.008 g, 0.03 mmol) were stirred in *t*-BuOH (2.0 mL) and H_2O (0.5 mL) for 16 h to give the intermediate triazole as an off-white gum after flash chromatography over silica gel (MeOH/CH_2Cl_2 – 0:100 → 6:94). Following **General Procedure VII**, the intermediate triazole (0.06 g, 0.06 mmol) was dissolved in CH_2Cl_2 (2 mL), treated with H_2O (0.03 g, 1.26 mmol) and CF_3COOH (1 mL) followed by work-up with ethereal HCl (3 mL) to give the amine salt **21** (0.03 g, 72% over two steps) as a pale brown solid that rapidly transitioned to a sticky gum. $[\alpha]_D^{23}$ +58.1 (*c* 0.0061, MeOH); ^{1}H NMR (400 MHz, CD_3OD) δ 8.36 (brs, 1H, H5), 8.18 (s, 1H, Hα), 8.19 (d, *J* = 8.9 Hz, 1H, Ar8), 8.00 (d, *J* = 7.9 Hz, 1H, Ar5), 7.59-7.46 (m, 3H, Ar4/Ar6/Ar7), 7.16 (d, *J* = 8.1 Hz, 1H, Ar3), 4.80-4.68 (m, 3H, OC<u>H</u>$_A$<u>H</u>$_B$/H1'), 4.62-4.60 (m, 1H, H1'), 4.58-4.42 (m, 1H, H2'), 3.15-3.14 (m, 2H, H5'), 2.94-2.91 (m, 2H, H1'''), 2.72 (brs, 1H, H1''), 1.91-1.88 (m, 2H, H3'), 1.79-1.57 (m, 10H, H4'/H2'''/H3'''/H2''/H3''/H4''/H5''/H6''), 1.20-1.16 (m, 5H, H2''/H3''/H4''/H5''/H6''), 1.01 (d, *J* = 5.8 Hz, 6H, H4'''/H5'''); ^{13}C NMR (101 MHz, CD_3OD) δ 169.2 (C=O), 157.1 (C=N), 150.6 (Ar2), 149.3 (C4), 147.6 (Cβ), 132.7 (Ar8a), 130.2 (Ar4), 129.3 (Ar4a), 128.2 (Ar5), 128.1 (Ar7), 127.0 (Ar8), 125.7 (C5), 125.2 (Cα), 120.2 (Ar6), 119.3 (Ar3), 113.8 (Ar1), 67.7 (O<u>C</u>H$_A$H$_B$), 55.7 (C1'), 49.1 (C2'), 40.4 (C5'), 37.9 (C2'''), 33.3 (C1''), 31.5 (C2''), 31.4 (C6''), 28.1 (C1'''), 27.5 (C3'), 25.16 (C3'''), 25.13 (C4''), 25.0 (C3''), 24.8 (C5''), 22.6 (C4'), 21.3 (C4'''/C5'''); IR (neat) $\bar{\nu}_{max}$ 3342, 3275, 3181, 2931, 2856, 1669, 1633, 1601, 1550, 1513, 1483, 1451, 1384, 1367, 1280, 1231, 1172, 1152, 1118, 1081, 1049, 860, 812, 778, 749, 668, 648, 588 cm^{-1}; MS (ESI +ve) *m/z* 615 ([M – HCl + H]$^+$, 100%); HRMS (ESI +ve TOF) calcd for $C_{33}H_{47}N_{10}O_2$ 615.5300, found 615.3877 ([M – HCl + H]$^+$).

(*R*)-*N*-(1-(4-(Cyclohexylmethyl)-1*H*-1,2,3-triazol-1-yl)-5-guanidinopentan-2-yl)-2-((1-(4-isopentyl-1*H*-1,2,3-triazol-1-yl)naphthalen-2-yl)oxy)acetamide hydrochloride (22)

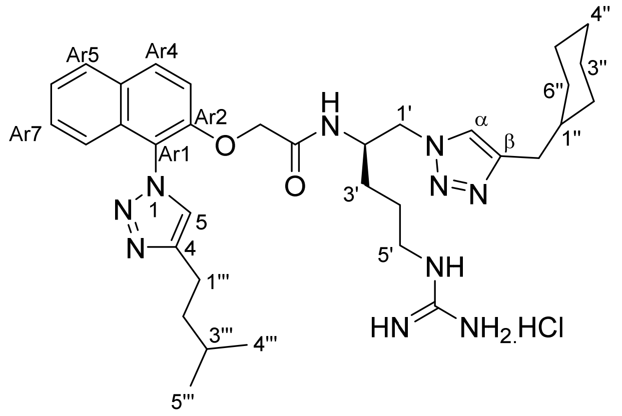

Following **General Procedure IV**, azide **19** (0.07 g, 0.09 mmol), 3-cyclohexyl-1-propyne (0.03 g, 0.27 mmol), $CuSO_4{\cdot}5H_2O$ (0.005 g, 0.02 mmol) and sodium ascorbate (0.008 g, 0.04 mmol) were stirred in *t*-BuOH (2 mL) and H_2O (0.5 mL) for 16 h to give the intermediate triazole as an off-white gum after flash chromatography over silica gel ($MeOH/CH_2Cl_2$ – 0:100 → 5:95). Following **General Procedure VII**, the intermediate triazole (0.06 g, 0.06 mmol) was dissolved in CH_2Cl_2 (2 mL), treated with H_2O (0.03 g, 1.36 mmol) and CF_3COOH (1 mL) followed by work-up with ethereal HCl (3 mL) to give the amine salt **22** (0.03 g, 66% over two steps) as a pale-yellow solid that rapidly transitioned to a sticky gum. $[\alpha]_D^{23}$ +74.1 (*c* 0.0056, MeOH). ^{1}H NMR (400 MHz, CD_3OD) δ 8.19 (s, 1H, H5), 8.14 (d, *J* = 8.0 Hz, 1H, Ar8), 8.00 (t, *J* = 8.9 Hz, 1H, Ar5), 7.70-7.62 (m, 1H, Ar6), 7.61 (s, 1H, Hα), 7.58-7.45 (m, 1H, Ar7), 7.39 (d, *J* = 8.1 Hz, 1H, Ar4), 6.99 (d, *J* = 8.1 Hz, 1H, Ar3), 4.62 (s, 2H, $OC\underline{H}_A\underline{H}_B$), 4.48-4.36 (m, 1H, H1'), 4.36-4.24 (m, 1H, H1'), 4.22-4.08 (m, 1H, H2'), 3.08-2.96 (m, 2H, H5'), 2.80-2.70 (m, 2H, H1'''), 2.37 (d, *J* = 6.2 Hz, 2H, βC-C$\underline{H}_2$), 1.70-1.32 (m, 13H, H3'/H4'/H2'''/H3'''/H1''/H2''/H3''/H4''/H5''/H6''), 1.16-0.99 (m, 3H, H2''/ H4''/H6''), 0.92 (d, *J* = 2.4 Hz, 6H, H4'''/H5'''), 0.81-0.78 (m, 2H, H3''/H5''); ^{13}C NMR (101 MHz, CD_3OD) δ 167.8 (C=O), 157.4 (C=N), 150.9 (Ar2), 147.4 (C4), 145.2 (Cβ), 132.1 (Ar8a), 130.7 (Ar4), 128.96 (Ar4a), 128.93 (Ar5), 128.5 (Ar7), 125.9 (Ar8), 125.3 (C5), 123.7 (Cα), 121.3 (Ar6), 119.9 (Ar3), 115.2 (Ar1), 68.1 ($O\underline{C}H_AH_B$), 53.1 (C1'), 49.1 (C2'), 40.7 (C5', Observed by gHMBC), 38.5 (C2'''), 37.8 (βC-$\underline{C}H_2$), 33.0 (C1'', Observed by gHMBC), 32.9 (C2''), 32.7 (C6''), 28.9 (C1'''), 27.6 (C3'), 26.4 (C3'''), 26.4 (C4'', Observed by gHMBC), 26.0 (C3''), 25.4 (C5''), 23.5 (C4'), 22.8 (C4'''/C5'''); IR (neat) $\bar{\nu}_{max}$ 3339, 3273, 3177, 2953, 2927, 2854, 1907, 1668, 1634, 1601, 1549, 1514, 1483, 1450, 1383, 1367, 1350, 1281, 1231, 1221, 1153, 1117, 1083, 1052, 1025, 964, 919, 862, 811, 778, 750, 647, 608 cm^{-1}; MS (ESI +ve) *m/z* 629 ([M – HCl + H]$^+$, 100%); HRMS (ESI +ve TOF) calcd for $C_{34}H_{49}N_{10}O_2$ 629.4034, found 629.4032 ([M – HCl + H]$^+$).

(*R*)-*N*-(1-(4-Benzyl-1*H*-1,2,3-triazol-1-yl)-5-guanidinopentan-2-yl)-2-((1-(4-isopentyl-1*H*-1,2,3-triazol-1-yl)naphthalen-2-yl)oxy)acetamide hydrochloride (23)

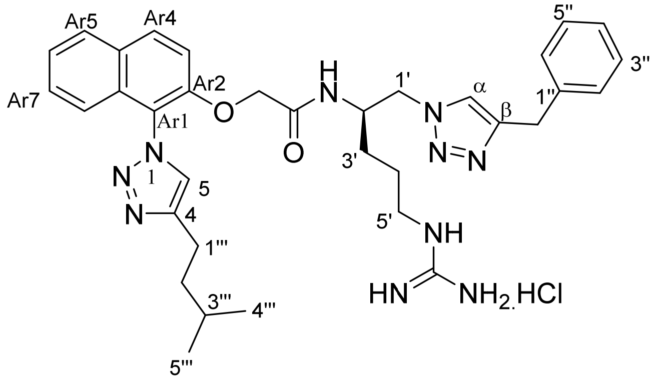

Following **General Procedure IV**, azide **19** (0.07 g, 0.09 mmol), 3-phenyl-1-propyne (0.03 g, 0.27 mmol), $CuSO_4{\cdot}5H_2O$ (0.005 g, 0.02 mmol) and sodium ascorbate (0.008 g, 0.04 mmol) were stirred in *t*-BuOH (2 mL) and H_2O (0.5 mL) for 16 h to give the intermediate triazole as a pale-yellow solid gum after flash chromatography over silica gel (MeOH/CH_2Cl_2 – 0:100 → 4:96). Following **General Procedure VII**, the intermediate triazole (0.06 g, 0.06 mmol) was dissolved in CH_2Cl_2 (2 mL), treated with H_2O (0.03 g, 1.2 mmol) and CF_3COOH (1 mL) followed by work-up with ethereal HCl (3 mL) to give the amine salt **23** (0.028 g, 68% over two step) as a pale brown solid that rapidly transitioned to a sticky gum. $[\alpha]_D^{23}$ +66.1 (*c* 0.0048, MeOH). ^{1}H NMR (400 MHz, CD_3OD) δ 8.42 (s, 1H, H5), 8.21 (s, 1H, Hα), 8.16 (d, *J* = 8.9 Hz, 1H, Ar8), 7.99 (d, *J* = 7.9 Hz, 1H, Ar5), 7.60-7.48 (m, 2H, Ar6/Ar7), 7.41 (d, *J* = 9.0 Hz, 1H, Ar4), 7.27-7.15 (m, 6H, Ar3/H2''/H3''/H4''/H5''/ H6''), 4.78-4.72 (m, 1H, H1'), 4.67 (ABq, *J* = 17.6 Hz, 2H, OC$\underline{H}_A\underline{H}_B$), 4.60-4.52 (m, 1H, H1'), 4.42-4.34 (m, 1H, H2'), 4.08 (s, 2H, C$\underline{H}_2$Ph), 3.22-3.08 (m, 2H, H5'), 3.00-2.88 (m, 2H, H1'''), 1.84-1.46 (m, 7H, H3'/H4'/H2'''/H3'''), 0.99 (d, *J* = 5.7 Hz, 6H, H4'''/H5'''); ^{13}C NMR (101 MHz, CD_3OD) δ 169.1 (C=O), 157.1 (C=N), 150.6 (Ar2), 147.0 (C4), 144.4 (Cβ), 136.0 (C1''), 132.9 (Ar8a), 130.0 (Ar4), 129.2 (Ar4a), 128.9 (Ar5), 128.68 (C2''), 128.65 (C6''), 128.3 (C3''/C5''), 128.1 (Ar7), 127.8 (C4''), 127.0 (Ar8), 126.8 (C5), 125.3 (Cα), 120.1 (Ar6), 118.8 (Ar3), 113.7 (Ar1), 67.7 (O$\underline{C}H_AH_B$), 55.4 (C1'), 49.2 (C2'), 40.5 (C5'), 37.6 (C2'''), 34.9 ($\underline{C}H_2$Ph, Observed by gHMBC), 29.3 (C1'''), 28.1 (C3'), 27.5 (C3'''), 24.8 (C4'), 22.3 (C4'''/C5'''); IR (neat) $\bar{\nu}_{max}$ 3339, 3272, 3182, 3067, 2956, 2869, 1901, 1669, 1634, 1601, 1550, 1513, 1496, 1482, 1454, 1433, 1382, 1367, 1281, 1232, 1221, 1153, 1120, 1080, 1049, 967, 931, 863, 811, 778, 750, 721, 698, 674, 648 cm^{-1}; MS (ESI +ve) *m/z* 623 ($[M - HCl + H]^+$, 100%); HRMS (ESI +ve TOF) calcd for $C_{34}H_{43}N_{10}O_2$ 623.3564, found 623.3561 ($[M - HCl + H]^+$).

***N*-((*R*)-1-(4-Cyclohexyl-1*H*-1,2,3-triazol-1-yl)-5-guanidinopentan-2-yl)-2-((1-(5-isopentyl-1*H*-1,2,3-triazol-1-yl)naphthalen-2-yl)oxy)acetamide hydrochloride (24)**

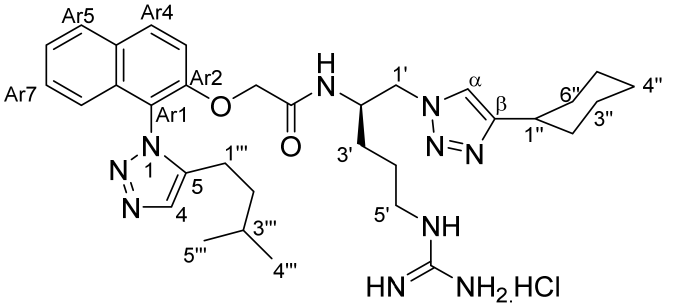

Following **General Procedure IV**, azide **20** (0.07 g, 0.09 mmol), cyclohexyl acetylene (0.03 g, 0.27 mmol), $CuSO_4 \cdot 5H_2O$ (0.005 g, 0.02 mmol) and sodium ascorbate (0.008 g, 0.04 mmol) were stirred in *t*-BuOH (2 mL) and H_2O (0.5 mL) for 16 h to give the intermediate triazole as a brown gum after flash chromatography over silica gel ($MeOH/CH_2Cl_2$ – 0:100 → 4:96). Following **General Procedure VII**, the intermediate triazole (0.07 g, 0.08 mmol) was dissolved in CH_2Cl_2 (2 mL), treated with H_2O (0.03 g, 1.6 mmol) and CF_3COOH (1 mL) followed by work-up with ethereal HCl (3 mL) to give the amine salt **24** (0.036 g, 52% over two steps) as a pale brown solid that rapidly transitioned to a sticky gum. $[\alpha]_D^{23}$ +65.6 (*c* 0.0062, MeOH). ^1H NMR (400 MHz, CD_3OD) δ 8.41 (brs, 1H, H4), 8.34-8.16 (m, 2H, Hα/HAr8), 8.08-8.00 (m, 2H, Ar5/Ar4), 7.60-7.50 (m, 2H, Ar6/Ar7), 6.99 (d, *J* = 9.6 Hz, 1H, Ar3), 4.86-4.78 (m, 2H, $OC\underline{H}_A\underline{H}_B$), 4.70-4.56 (m, 2H, H1'), 4.44-4.32 (m, 1H, H2'), 3.05 (t, *J* = 7.0 Hz, 2H, H5'), 2.66-2.58 (m, 1H, H1''), 2.56-2.44 (m, 2H, H1'''), 2.04-1.50 (m, 10H, H4'/H2'''/H3'''/H2''/H3''/H4''/H5''/H6''), 1.48-1.06 (m, 7H, H3'/H2''/H3''/H4''/H5''/H6''), 0.69 (d, *J* = 7.0 Hz, 3H, H4'''), 0.67 (d, *J* = 7.1 Hz, 3H, H5'''); ^{13}C NMR (101 MHz, CD_3OD) δ 169.1 (C=O), 157.2 (C=N), 151.3 (Ar2), 142.9 (Cβ), 133.1 (C5), 131.7 (Ar8a), 130.5 (C4), 129.3 (Ar4), 128.9 (Ar4a), 128.3 (Ar5), 126.2 (Ar7), 125.3 (Ar8), 120.26 (Ar6), 120.23 (Cα), 117.7 (Ar3), 113.8 (Ar1), 67.6 ($O\underline{C}H_AH_B$), 55.9 (C1'), 49.3 (C2'), 40.6 (C5'), 36.5 (C2'''), 33.3 (C1''), 31.5 (C2''), 31.4 (C6''), 28.3 (C1'''), 27.0 (C3'), 25.2 (C3'''), 25.1 (C4''), 25.0 (C3''), 24.8 (C5''), 20.9 (C4'), 20.6 (C4'''/C5'''); IR (neat) $\bar{\nu}_{max}$ 3338, 3268, 3179, 3073, 2932, 2857, 2481, 1903, 1669, 1632, 1601, 1549, 1512, 1482, 1451, 1385, 1367, 1351, 1272, 1234, 1224, 1170, 1154, 1132, 1090, 1048, 990, 972, 941, 920, 858, 816, 750, 652 cm^{-1}; MS (ESI +ve) *m/z* 616 ($[M - HCl + H]^+$, 100%); HRMS (ESI +ve TOF) calcd for $C_{33}H_{47}N_{10}O_2$ 615.3883, found 615.3873 ($[M - HCl + H]^+$).

N-((R)-1-(4-(Cyclohexylmethyl)-1H-1,2,3-triazol-1-yl)-5-guanidinopentan-2-yl)-2-((1-(5-isopentyl-1H-1,2,3-triazol-1-yl)naphthalen-2-yl)oxy)acetamide hydrochloride (25)

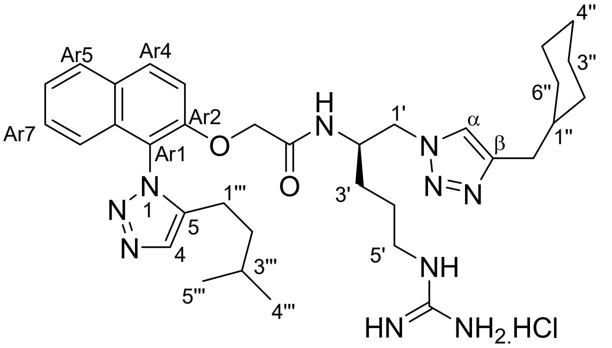

Following **General Procedure IV**, azide **20** (0.07 g, 0.09 mmol), 3-cyclohexyl-1-propyne (0.03 g, 0.27 mmol), $CuSO_4 \cdot 5H_2O$ (0.005 g, 0.02 mmol) and sodium ascorbate (0.008 g, 0.04 mmol) were stirred in *t*-BuOH (2 mL) and H_2O (0.5 mL) for 16 h to give the intermediate triazole as an off-white gum after flash chromatography over silica gel ($MeOH/CH_2Cl_2$ – 0:100 → 4:96). Following **General Procedure VII**, the intermediate triazole (0.07 g, 0.08 mmol) was dissolved in CH_2Cl_2 (2 mL), treated with H_2O (0.03 g, 1.6 mmol) and CF_3COOH (1 mL) followed by work-up with ethereal HCl (3 mL) to give the amine salt **25** (0.03 g, 51% over two steps) as a pale brown solid that rapidly transitioned to a sticky gum. $[\alpha]_D^{23}$ +58.1 (*c* 0.0044, MeOH). ^1H NMR (400 MHz, CD_3OD) δ 8.35 (s, 1H, H4), 8.19 (s, 1H, Hα), 8.08-8.00 (m, 2H, Ar8/Ar5), 7.58-7.50 (m, 3H, Ar4/Ar6/Ar7), 6.99 (d, *J* = 9.6 Hz, 1H, Ar3), 4.78 (ABq, *J* = 19.0 Hz, 2H, OC$\underline{H}_A\underline{H}_B$), 4.67-4.54 (m, 2H, H1'/H2'), 4.44-4.30 (m, 1H, H1'), 3.26-3.20 (m, 1H, H1'''), 3.11-2.93 (m, 1H, H1'''), 2.67 (d, *J* = 12.8 Hz, 1H, βC-C$\underline{H}_2$), 2.54-2.44 (m, 3H, H5'/βC-C$\underline{H}_2$), 1.85-1.30 (m, 13H, H3'/H4'/H2'''/H3'''/H1"/H2"/H3"/H4"/H5"/H6"), 1.29-1.04 (m, 3H, H2"/H4"/H6"), 1.02-0.78 (m, 2H, H3"/H5"), 0.72-0.62 (m, 6H, H4'''/H5'''); ^{13}C NMR (101 MHz, CD_3OD) δ 168.8 (C=O), 157.0 (C=N), 151.0 (Ar2), 142.9 (C5), 132.9 (Ar8a), 131.6 (C4), 130.6 (Cβ), 129.4 (Ar4), 128.9 (Ar4a), 128.3 (Ar5), 126.8 (Ar7), 125.3 (Ar8), 120.3 (Ar6), 120.2 (Cα), 117.7 (Ar3), 113.8 (Ar1), 67.6 (O$\underline{C}H_AH_B$), 55.5 (C1'), 49.3 (C2'), 37.5 (C2'''), 36.5 (C5'), 33.9 (C1"), 33.0 (Cβ-$\underline{C}H_2$), 32.3 (C2"), 32.2 (C6"), 30.9 (C3'), 30.7 (3'''), 28.3 (C1'''), 28.1 (C4"), 27.0 (C3"), 25.7 (C5"), 25.7 (C4'), 25.6 (C4'''/5'''); IR (neat) $\bar{\nu}_{max}$ 3341, 3271, 3180, 3082, 2953, 2927, 2854, 2468, 1916, 1670, 1632, 1601, 1549, 1512, 1483, 1450, 1384, 1368, 1349, 1223, 1169, 1154, 1132, 1091, 1049, 1025, 991, 973, 935, 918, 863, 814, 780, 750, 678, 651 cm^{-1}; MS (ESI +ve) *m/z* 630 ([M – HCl + H]$^+$, 100%); HRMS (ESI +ve TOF) calcd for $C_{34}H_{49}N_{10}O_2$ 629.4040, found 629.4059 ([M – HCl + H]$^+$).

***N*-((*R*)-1-(4-Benzyl-1*H*-1,2,3-triazol-1-yl)-5-guanidinopentan-2-yl)-2-((1-(5-isopentyl-1*H*-1,2,3-triazol-1-yl)naphthalen-2-yl)oxy)acetamide hydrochloride (26)**

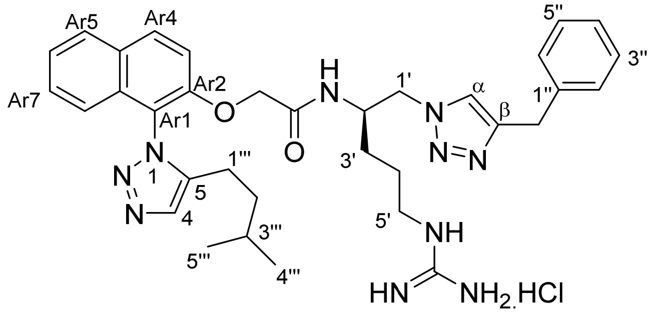

Following **General Procedure IV**, azide **20** (0.07 g, 0.09 mmol), 3-phenyl-1-propyne (0.03 g, 0.27 mmol), $CuSO_4{\cdot}5H_2O$ (0.005 g, 0.02 mmol) and sodium ascorbate (0.008 g, 0.04 mmol) were stirred in *t*-BuOH (2 mL) and H_2O (0.5 mL) for 16 h to give the intermediate triazole as an off-white gum after flash chromatography over silica gel ($MeOH/CH_2Cl_2$ – 0:100 → 6:94). Following **General Procedure VII**, the intermediate triazole (0.07 g, 0.08 mmol) was dissolved in CH_2Cl_2 (2 mL), treated with H_2O (0.03 g, 1.6 mmol) and CF_3COOH (1 mL) followed by work-up with ethereal HCl (3 mL) to give the amine salt **26** (0.034 g, 57% over two steps) as a brown solid that rapidly transitioned to a sticky gum. $[\alpha]_D^{23}$ +73.7 (*c* 0.0051, MeOH). ^{1}H NMR (400 MHz, CD_3OD) δ 8.23-8.08 (m, 3H, H4/Hα/Ar8), 8.02 (d, *J* = 8.2 Hz, 1H, Ar5), 7.60-7.38 (m, 3H, Ar4/Ar6/Ar7), 7.31-7.13 (m, 5H, H2''/H3''/ H4''/H5''/H6''), 7.00 (d, *J* = 8.5 Hz, 1H, Ar3), 4.71 (ABq, *J* = 18.2 Hz, 2H, $OC\underline{H}_A\underline{H}_B$), 4.64-4.56 (m, 1H, H1'), 4.56-4.46 (m, 1H, H2'), 4.42-4.28 (m, 1H, H1'), 4.15 (s, 1H, $C\underline{H}_2Ph$), 4.01 (s, 1H, $C\underline{H}_2Ph$), 3.21-3.15 (m, 1H, H1'''), 3.10-2.99 (m, 1H, H1'''), 2.51 (m, 2H, H5'), 1.82-1.52 (m, 3H, H2'''/H3'''), 1.50-1.29 (m, 4H, H3'/H4'), 0.69 (d, *J* = 5.7 Hz, 3H, H4'''), 0.66 (d, *J* = 7.2 Hz, 3H, H5'''); ^{13}C NMR (101 MHz, CD_3OD) δ 167.5 (C=O), 157.8 (C=N), 151.8 (Ar2), 149.6 (C5), 140.4 (Ar8a), 138.5 (C1''), 133.3 (C4), 132.5 (Cβ), 130.3 (Ar4), 129.2 (Ar4a), 128.86 (C2''/C6''), 128.82 (Ar5), 128.74 (C3''/C5''), 128.71 (C4''), 128.6 (Ar7), 126.5 (Ar8), 125.3 (Ar6), 121.1 (Cα), 115.5 (Ar3), 115.4 (Ar1), 68.3 ($O\underline{C}H_AH_B$), 62.7 (C1'), 53.0 (C2'), 41.1 (C2'''), 40.8 (C5'), 36.8 ($\underline{C}H_2Ph$), 31.8 (C3'), 31.7 (3'''), 29.1 (C1'''), 25.3 (C4'), 22.1 (C4'''/5'''); IR (neat) $\bar{\nu}_{max}$ 3343, 3273, 3181, 3065, 2956, 2934, 2869, 2469, 1906, 1669, 1633, 1601, 1549, 1512, 1497, 1482, 1454, 1384, 1367, 1351, 1277, 1234, 1223, 1169, 1153, 1132, 1086, 1049, 992, 972, 934, 919, 863, 813, 780, 751, 722, 698, 652 cm^{-1}; MS (ESI +ve) *m/z* 624 ($[M – HCl + H]^+$, 100%); HRMS (ESI +ve TOF) calcd for $C_{34}H_{43}N_{10}O_2$ 623.3570, found 623.3581 ($[M – HCl + H]^+$).

(*R*)-6-Amino-*N*-((*R*)-1-(4-benzyl-1*H*-1,2,3-triazol-1-yl)-5-guanidinopentan-2-yl)-2-(2-(2-(4-isopentyl-1*H*-1,2,3-triazol-1-yl)phenoxy)acetamido)hexanamide dihydro chloride (36)

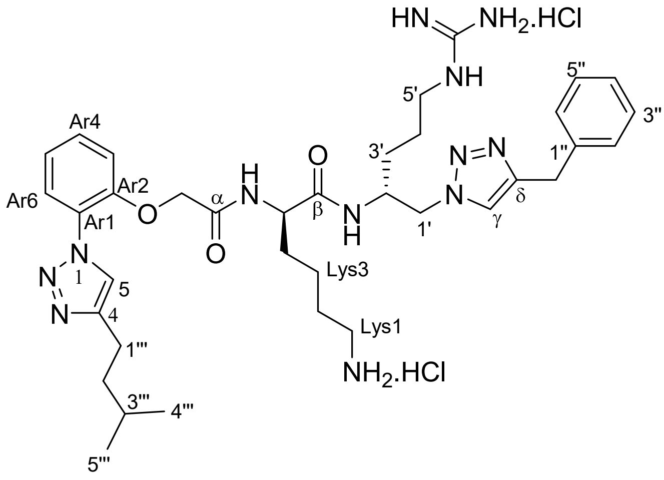

Following **General Procedure IV**, azide **30** (0.07 g, 0.07 mmol), 3-phenyl-1-propyne (0.03 g, 0.21 mmol), $CuSO_4{\cdot}5H_2O$ (0.004 g, 0.01 mmol) and sodium ascorbate (0.006 g, 0.02 mmol) were stirred in *t*-BuOH (2 mL) and H_2O (0.5 mL) for 16 h to give the intermediate triazole as an off-white solid after flash chromatography over silica gel (MeOH/CH_2Cl_2 – 0:100 → 5:95). Following **General Procedure VII**, the intermediate triazole (0.06 g, 0.06 mmol) was dissolved in CH_2Cl_2 (2 mL), treated with H_2O (0.02 g, 1.10 mmol) and CF_3COOH (1 mL) followed by work-up with ethereal HCl (3 mL) to give the amine salt **36** (0.030 g, 56% over two steps) as a pale-yellow solid that rapidly transitioned to a sticky gum. $[\alpha]_D^{23}$ +68.1 (*c* 0.0052, MeOH). ^{1}H NMR (400 MHz, CD_3OD) δ 8.54 (s, 1H, H5), 8.12 (s, 1H, Hγ), 7.65 (t, *J* = 7.5 Hz, 1H, Ar4), 7.54 (d, *J* = 8.0 Hz, 1H, Ar6), 7.32-7.20 (m, 7H, Ar5/Ar3/H2''/H3''/ H4''/H5''/H6''), 4.84-4.74 (m, 2H, OC<u>H</u>$_A$<u>H</u>$_B$), 4.68-4.62 (m, 1H, H1'), 4.58-4.48 (m, 1H, H1'), 4.36-4.28 (m, 1H, Lys5), 4.20-4.12 (m, 1H, H2'), 4.12 (s, 2H, δC-C<u>H</u>$_2$), 3.24-3.10 (m, 2H, H5'), 2.94-2.80 (m, 4H, Lys1/H1'''), 1.82-1.50 (m, 11H, H2'''/H3'''/H3'/Lys4/H4'/Lys2), 1.36-1.24 (m, 2H, Lys3), 0.96 (d, *J* = 5.0 Hz, 6H, H4'''/H5'''); ^{13}C NMR (101 MHz, CD_3OD) δ 173.9 (βC=O), 170.4 (αC=O), 158.4 (C=N), 152.0 (Ar2), 137.5 (Cδ), 133.5 (C4), 131.1 (C1''), 130.2 (C2''/C6''), 129.9 (C3''/C5''), 127.6 (C4''/Ar4), 127.4 (Ar6), 126.2 (C5), 125.9 (Cγ), 123.9 (Ar5), 123.3 (Ar1), 115.7 (Ar3), 69.7 (O<u>C</u>H$_A$H$_B$), 57.6 (C1'), 54.9 (Lys5), 49.6 (C2'), 42.6 (C2'''), 40.7 (C5'), 38.5 (Lys1), 32.8 (δC-<u>C</u>H$_2$), 31.6 (C3'), 29.8 (Lys4), 29.6 (C1'''), 28.6 (Lys2), 28.2 (C3'''), 25.8 (C4'), 23.2 (C4'''/C5'''), 22.8 (Lys3); IR (neat) $\bar{\nu}_{max}$ 3375, 2956, 2932, 2870, 1653, 1572, 1548, 1508, 1462, 1381, 1367, 1288, 1254, 1228, 1150, 1113, 1058, 1005, 914, 883, 837, 793, 759, 721, 697, 670, 657 cm^{-1}; MS (ESI +ve) *m/z* 701 ([M –2HCl + H]$^+$, 100%), 351 ([M – 2HCl + H]$^{2+}$, 40%); HRMS (ESI +ve TOF) calcd for $C_{36}H_{55}N_{12}O_3Cl_2$ 773.1236, found 773.1240 ([M + H]$^+$).

(*R*)-6-Amino-*N*-((*R*)-1-(4-cyclohexyl-1*H*-1,2,3-triazol-1-yl)-5-guanidinopentan-2-yl)-2-(2-(2-(5-isopentyl-1*H*-1,2,3-triazol-1-yl)phenoxy)acetamido)hexanamide dihydrochloride (37)

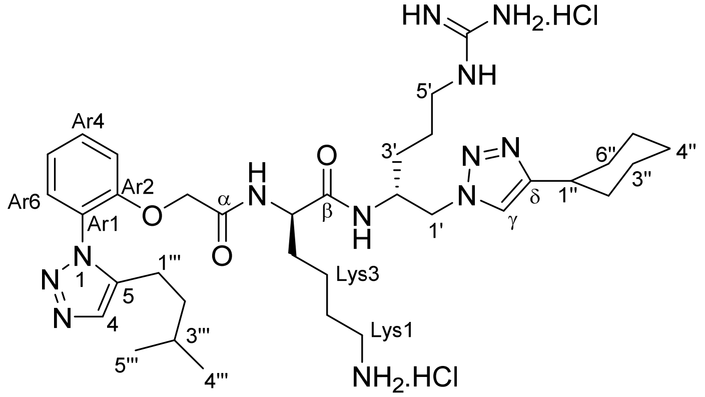

Following **General Procedure IV**, azide **31** (0.02 g, 0.02 mmol), cyclohexylacetylene (0.007 g, 0.06 mmol), $CuSO_4·5H_2O$ (0.001 g, 0.004 mmol) and sodium ascorbate (0.002 g, 0.008 mmol) were stirred in *t*-BuOH (1.2 mL) and H_2O (0.3 mL) for 16 h to give the intermediate triazole as an off-white gum after flash chromatography over silica gel ($MeOH/CH_2Cl_2$ – 0:100 → 4:96). Following **General Procedure VII**, the intermediate triazole (0.02 g, 0.02 mmol) was dissolved in CH_2Cl_2 (2 mL), treated with H_2O (0.01 g, 0.40 mmol) and CF_3COOH (1 mL) followed by work-up with ethereal HCl (2 mL) to give the amine salt **37** (0.015 g, 98% over two steps) as an off-white solid that rapidly transitioned to a sticky gum. $[\alpha]_D^{23}$ +65.8 (*c* 0.0026, MeOH). ^{1}H NMR (400 MHz, CD_3OD) δ 8.35 (s, 1H, H4), 7.93 (s, 1H, Hγ), 7.68-7.60 (m, 1H, Ar4), 7.47 (d, *J* = 7.0 Hz, 1H, Ar6), 7.33 (t, *J* = 7.0 Hz, 1H, Ar5), 7.28-7.20 (m, 1H, Ar3), 4.80-4.68 (m, 3H, OC<u>H</u>$_A$<u>H</u>$_B$/H1'), 4.60-4.50 (m, 1H, H1'), 4.42-4.32 (m, 1H, Lys5), 4.16-4.08 (m, 1H, H2'), 3.28-3.14 (m, 2H, H1'''), 3.00-2.84 (m, 3H, H5'/H1''), 2.70-2.62 (m, 2H, Lys1), 1.88-1.56 (m, 13H, H2'''/H3'''/H3'/Lys4/H4'/Lys2/Lys3), 1.54-1.38 (m, 8H, H2''/H3''/H4''/H5''/H6''), 1.38-1.24 (m, 2H, H3''/H5''), 0.81 (d, *J* = 6.0 Hz, 6H, H4'''/H5'''); ^{13}C NMR (101 MHz, CD_3OD) δ 174.4 (βC=O), 170.2 (αC=O), 158.8 (C=N), 154.2 (Ar2), 143.4 (Cδ), 139.4 (C5), 134.0 (C4), 129.8 (Ar4), 127.1 (Ar6), 125.8 (Ar1), 123.56 (Cγ), 123.52 (Ar5), 115.5 (Ar3), 68.6 (O<u>C</u>H$_A$H$_B$), 57.3 (C1'), 54.9 (Lys5), 50.9 (C2'), 42.1 (C2'''), 40.7 (C5'), 38.2 (Lys1), 35.1 (C1''), 33.3 (C3'), 32.6 (C2''/C6''), 29.7 (Lys4), 29.6 (Lys2), 28.8 (C3'''), 28.1 (C1'''), 26.9 (C4''), 26.7 (C3''/C5''), 24.2 (C4'), 23.6 (C4'''/C5'''), 22.7 (Lys3); IR (neat) $\bar{\nu}_{max}$ 3378, 3350, 3265, 3066, 2932, 2864, 1915, 1664, 1603, 1548, 1508, 1464, 1452, 1384, 1367, 1286, 1272, 1250, 1230, 1167, 1114, 1090, 1057, 983, 956, 915, 892, 882, 837, 819, 802, 794, 761, 720, 667, 645, 628, 581 cm^{-1}; MS (ESI +ve) *m/z* 693 ([M – 2HCl + H]$^+$, 10%), 347 ([M – 2HCl + H]$^{2+}$, 100%); HRMS (ESI +ve TOF) calcd for $C_{35}H_{59}N_{12}O_3Cl_2$ 765.4210, found 765.4248 ([M + H]$^+$).

(*R*)-6-Amino-*N*-((*R*)-1-(4-(cyclohexylmethyl)-1*H*-1,2,3-triazol-1-yl)-5-guanidinopen- tan-2-yl)-2-(2-(2-(5-isopentyl-1*H*-1,2,3-triazol-1-yl)phenoxy)acetamido)hexanamide dihydrochloride (38)

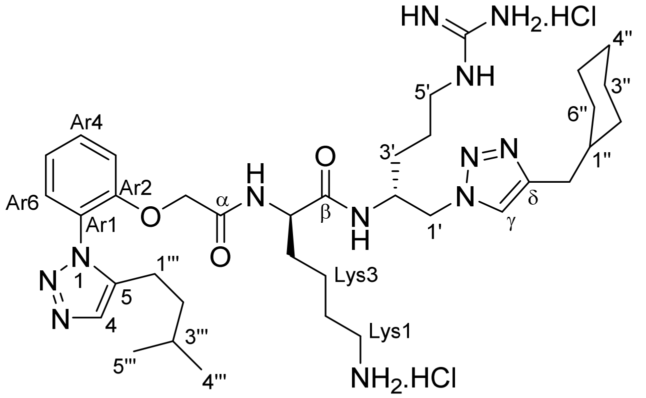

Following **General Procedure IV**, azide **31** (0.10 g, 0.10 mmol), 3-cyclohexyl-1-propyne (0.04 g, 0.32 mmol), $CuSO_4 \cdot 5H_2O$ (0.005 g, 0.02 mmol) and sodium ascorbate (0.008 g, 0.04 mmol) were stirred in *t*-BuOH (2 mL) and H_2O (0.5 mL) for 16 h to give the intermediate triazole as an off-white solid after flash chromatography over silica gel (MeOH/CH_2Cl_2 – 0:100 → 4:96). Following **General Procedure VII**, the intermediate triazole (0.07 g, 0.06 mmol) was dissolved in CH_2Cl_2 (2 mL), treated with H_2O (0.02 g, 1.32 mmol) and CF_3COOH (1 mL) followed by work-up with ethereal HCl (3 mL) to give the amine salt **38** (0.050 g, 64% over two steps) as an off-white solid that rapidly transitioned to a sticky gum. $[\alpha]_D^{23}$ +61.4 (*c* 0.0048, MeOH). ^{1}H NMR (400 MHz, CD_3OD) δ 8.43 (s, 1H, H4), 8.18 (s, 1H, Hγ), 7.67 (apparent t, *J* = 8.9 Hz, 1H, Ar4), 7.51 (d, *J* = 9.1 Hz, 1H, Ar6), 7.35 (d, *J* = 9.1 Hz, 1H, Ar5), 7.26 (t, *J* = 8.9 Hz, 1H, Ar3), 4.74-4.60 (m, 3H, OC$\underline{H}_A\underline{H}_B$/H1'), 4.51-4.43 (m, 1H, H1'), 4.38-4.30 (m, 1H, Lys5), 4.14-4.08 (m, 1H, H2'), 3.22-3.13 (m, 2H, H1'''), 2.96-2.87 (m, 2H, H5'), 2.66-2.60 (m, 4H, δC-C$\underline{H}_2$/Lys1), 1.78-1.54 (m, 14H, H1"/Lys4/Lys2 /Lys3/H3'/H4'/H2'''/H3'''), 1.50-1.36 (m, 5H, H2"/H3"/H4"/H5"/H6"), 1.33-1.12 (m, 3H, H2"/H4"/ H6"), 1.05-0.91 (m, 2H, H3"/H5"), 0.80 (d, *J* = 6.4 Hz, 6H, H4'''/H5'''); ^{13}C NMR (101 MHz, CD_3OD) δ 172.9 (βC=O), 168.5 (αC=O), 157.2 (C=N), 152.5 (Ar2), 152.4 (Cδ), 142.9 (C5), 132.9 (C4), 129.3 (Ar4), 128.3 (Ar6), 127.8 (Ar1), 123.7 (Cγ), 121.9 (Ar5), 114.0 (Ar3), 67.1 (O$\underline{C}H_AH_B$), 56.3 (C1'), 53.4 (Lys5), 49.4 (C2'), 40.5 (C2'''), 39.2 (C5'), 37.3 (Lys1), 36.4 (C1"), 32.3 (δC-$\underline{C}H_2$), 32.2 (C2"), 31.0 (C6"), 30.4 (C3'), 28.1 (Lys4), 27.2 (Lys2), 26.5 (C3'''), 25.7 (C1'''), 25.6 (C4"), 24.8 (C3"/C5"), 22.6 (C4'), 21.1 (C4'''/C5'''), 20.9 (Lys3); IR (neat) $\bar{\nu}_{max}$ 3351, 3268, 3195, 3066, 2927, 2856, 2667, 2067, 1916, 1662, 1543, 1508, 1465, 1451, 1386, 1368, 1290, 1229, 1168, 1129, 1051, 985, 958, 936, 916, 846, 762, 668, 646, 585 cm^{-1}; MS (ESI +ve) *m/z* 707 ([M – 2HCl + H]$^+$, 100%); HRMS (ESI +ve TOF) calcd for $C_{36}H_{59}N_{12}O_3$ 707.4827, found 707.4824 ([M – 2HCl + H]$^+$).

(*R*)-6-Amino-*N*-((*R*)-1-(4-benzyl-1*H*-1,2,3-triazol-1-yl)-5-guanidinopentan-2-yl)-2-(2-(2-(5-isopentyl-1*H*-1,2,3-triazol-1-yl)phenoxy)acetamido)hexanamide dihydrochloride (39)

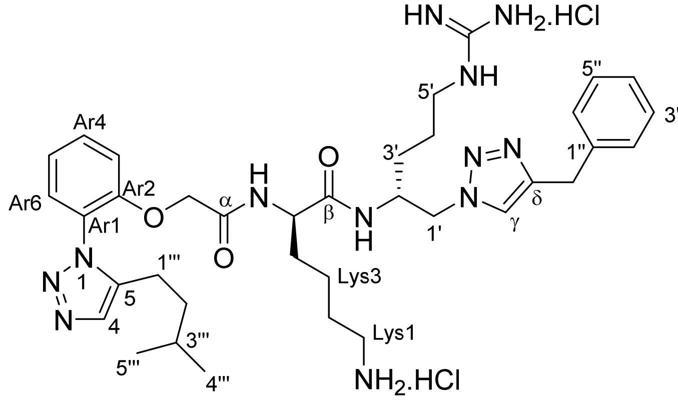

Following **General Procedure IV**, azide **31** (0.03 g, 0.03 mmol), 3-phenyl-1-propyne (0.01 g, 0.09 mmol), $CuSO_4{\cdot}5H_2O$ (0.002 g, 0.006 mmol) and sodium ascorbate (0.003 g, 0.012 mmol) were stirred in *t*-BuOH (1.5 mL) and H_2O (0.3 mL) for 16 h to give the intermediate triazole as an off-white gum after flash chromatography over silica gel ($MeOH/CH_2Cl_2$ – 0:100 → 4:96). Following **General Procedure VII**, the intermediate triazole (0.03 g, 0.03 mmol) was dissolved in CH_2Cl_2 (2 mL), treated with H_2O (0.01 g, 0.56 mmol) and CF_3COOH (1 mL) followed by work-up with ethereal HCl (2 mL) to give the amine salt **39** (0.012 g, 52% over two steps) as an off-white solid that rapidly transitioned to a sticky gum. $[\alpha]_D^{23}$ +66.5 (*c* 0.0028, MeOH). ^1H NMR (400 MHz, CD_3OD) δ 8.16 (s, 1H, H4), 7.64 (s, 1H, Hγ), 7.63 (apparent t, *J* = 7.0 Hz, 1H, Ar4), 7.48-7.41 (m, 1H, Ar6), 7.36-7.20 (m, 7H, Ar5/Ar3/H2''/H3''/H4''/H5''/H6''), 4.80-4.58 (m, 3H, OC<u>H</u>$_A$<u>H</u>$_B$/H1'), 4.57-4.44 (m, 1H, H1'), 4.36-4.28 (m, 1H, Lys5), 4.20-4.08 (m, 3H, H2'/H1'''), 3.26-3.14 (m, 2H, H5'), 2.92-2.82 (m, 2H, C<u>H</u>$_2$Ph), 2.68-2.60 (m, 2H, Lys1), 1.80-1.36 (m, 11H, Lys4/Lys2/H3'/H4'/H2'''/H3'''), 1.30-1.20 (m, 2H, Lys3), 0.79 (d, *J* = 5.5 Hz, 3H, H4'''), 0.76 (d, *J* = 6.0 Hz, 3H, H5'''); ^{13}C NMR (101 MHz, CD_3OD) δ 174.2 (βC=O), 170.0 (αC=O), 158.7 (C=N), 154.1 (Ar2), 154.0 (Cδ), 138.4 (C5), 134.1 (C1''), 130.1 (C4), 130.0 (Ar4), 129.9 (C2''/C6''), 129.89 (Ar6), 129.86 (C3''/C5''), 128.3 (C4''), 125.7 (Ar1), 123.5 (Cγ), 115.4 (Ar5), 111.5 (Ar3), 68.6 (O<u>C</u>H$_A$H$_B$), 56.4 (C1'), 54.8 (Lys5), 50.8 (C2'), 42.1 (C2'''), 40.6 (C5'), 38.1 (Lys1), 32.5 (<u>C</u>H$_2$Ph), 31.4 (C3'), 29.9 (Lys4), 29.8 (Lys2), 28.7 (C3'''), 28.1 (C1'''), 26.4 (C4'), 23.9 (Lys3), 22.7 (C4'''/C5'''); IR (neat) $\bar{\nu}_{max}$ 3374, 3075, 2956, 2932, 2870, 1654, 1572, 1548, 1508, 1462, 1381, 1367, 1288, 1253, 1229, 1150, 1113, 1058, 1005, 914, 883, 838, 793, 759, 720, 697, 670, 657 cm^{-1}; MS (ESI +ve) *m/z* 701 ([M – 2HCl + H]$^+$, 70%), 351 ([M – 2HCl + H]$^{2+}$, 100%); HRMS (ESI +ve TOF) calcd for $C_{36}H_{55}N_{12}O_3Cl_2$ 773.3897, found 773.3914 ([M + H]$^+$).

(*R*)-6-Amino-*N*-((*R*)-1-(4-cyclohexyl-1*H*-1,2,3-triazol-1-yl)-5-guanidinopentan-2-yl)-2-(2-((1-(4-isopentyl-1*H*-1,2,3-triazol-1-yl)naphthalen-2-yl)oxy)acetamido)hexanamide dihydrochloride (40)

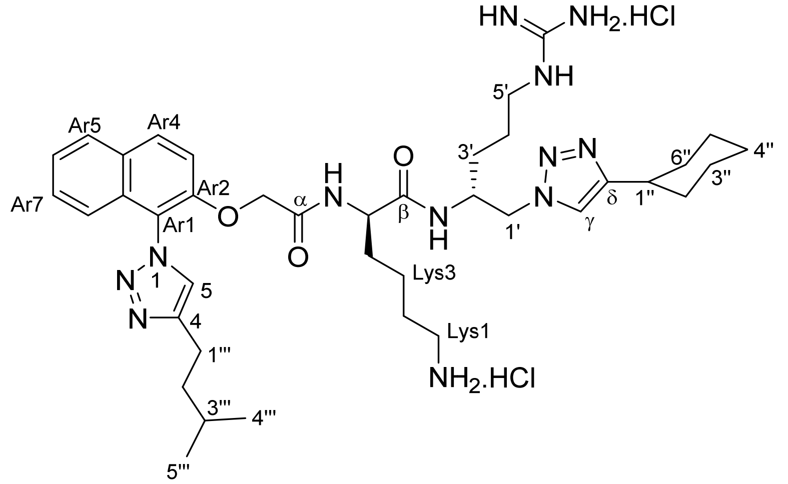

Following **General Procedure IV**, azide **32** (0.08 g, 0.08mmol), cyclohexylacetylene (0.03 g, 0.24 mmol), $CuSO_4{\cdot}5H_2O$ (0.004 g, 0.01 mmol) and sodium ascorbate (0.006 g, 0.03 mmol) were stirred in *t*-BuOH (2.0 mL) and H_2O (0.5 mL) for 16 h to give the intermediate triazole as an off-white gum after flash chromatography over silica gel ($MeOH/CH_2Cl_2$ – 0:100 → 8:92). Following **General Procedure VII**, the intermediate triazole (0.06 g, 0.05 mmol) was dissolved in CH_2Cl_2 (2 mL), treated with H_2O (0.02 g, 1.00 mmol) and CF_3COOH (1 mL) followed by work-up with ethereal HCl (3 mL) to give the amine salt **40** (0.03 g, 46% over two steps) as an off-white solid that rapidly transitioned to a sticky gum. $[\alpha]_D^{23}$ +59.1 (*c* 0.0052, MeOH); ^{1}H NMR (400 MHz, CD_3OD) δ 8.30 (s, 1H, H5), 8.29 (s, 1H, Hγ), 8.18 (d, *J* = 9.2 Hz, 1H, Ar8), 7.98 (d, *J* = 7.5 Hz, 1H, Ar5), 7.61 (ddd, *J* = 9.2, 9.2, 1.7 Hz, 1H, Ar7), 7.57-7.49 (m, 2H, Ar6/Ar4), 7.14 (d, *J* = 8.3 Hz, 1H, Ar3), 4.93-4.89 (m, 2H, $OC\underline{H}_A\underline{H}_B$), 4.77-4.72 (m, 1H, H1'), 4.59-4.53 (m, 1H, H1'), 4.37-4.32 (m, 1H, Lys5), 4.12-4.09 (m, 1H, H2'), 3.18-3.14 (m, 2H, H5'), 2.95-2.91 (m, 2H, Lys1), 2.84-2.78 (m, 3H, H1'''/H1"), 2.00-1.96 (m, 2H, Lys4), 1.74-1.60 (m, 14H, H2'''/H3'''/Lys2/H3'/H4'/H2"/H3"/H4"/H5"/H6"), 1.48-1.21 (m, 7H, Lys3/H2"/H3"/ H4"/H5"/H6"), 1.01 (d, *J* = 6.2 Hz, 6H, H4'''/H5'''); ^{13}C NMR (101 MHz, CD_3OD) δ 173.0 (βC=O), 169.1 (αC=O), 157.1 (C=N), 150.8 (Ar2), 148.7 (C4), 147.6 (Cδ), 132.5 (Ar8a), 130.3 (Ar4), 129.1 (Ar4a), 128.6 (Ar5), 128.0 (Ar7), 126.9 (Ar8), 125.5 (C5), 125.1 (Cγ), 120.2 (Ar6), 119.1 (Ar3), 113.9 (Ar1), 67.4 ($O\underline{C}H_AH_B$), 55.7 (C1'), 53.5 (Lys5), 49.3 (C2'), 40.4 (C5'), 39.0 (Lys1), 37.9 (C2'''), 33.4 (C1"), 31.6 (C2"), 31.5 (C6"), 30.9 (Lys4), 28.1 (Lys2), 27.5 (C1'''), 26.5 (C3'), 25.2 (C4"), 25.0 (C3"/C5"), 24.8 (C3'''), 22.6 (C4'), 22.5 (C4'''/C5'''), 21.3 (Lys3); IR (neat) $\bar{\nu}_{max}$ 3348, 3265, 3202, 3066, 2932, 2860, 1662, 1544, 1514, 1483, 1451, 1384, 1366, 1349, 1279, 1220, 1168, 1117, 1081, 1049, 816, 749, 668, 585 cm^{-1}; MS (ESI +ve) *m/z* 743 ([M – 2HCl + H]$^+$, 60%), 372 ([M – 2HCl + H]$^{2+}$, 100%); HRMS (ESI +ve TOF) calcd for $C_{39}H_{59}N_{12}O_3$ 743.4833, found 743.4866 ([M – 2HCl + H]$^+$).

(*R*)-6-Amino-*N*-((*R*)-1-(4-(cyclohexylmethyl)-1*H*-1,2,3-triazol-1-yl)-5-guanidinopen- tan-2-yl)-2-(2-((1-(4-isopentyl-1*H*-1,2,3-triazol-1-yl)naphthalen-2-yl)oxy)acetamido)hexanamide dihydrochloride (41)

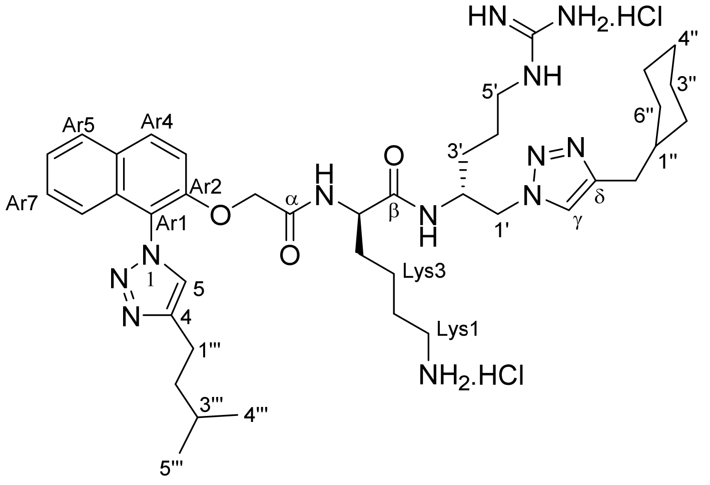

Following **General Procedure IV**, azide **32** (0.07 g, 0.07 mmol), 3-cyclohexyl-1-propyne (0.03 g, 0.21 mmol), $CuSO_4 \cdot 5H_2O$ (0.004 g, 0.01 mmol) and sodium ascorbate (0.006 g, 0.02 mmol) were stirred in *t*-BuOH (2 mL) and H_2O (0.5 mL) for 16 h to give the intermediate triazole as a light brown waxy solid after flash chromatography over silica gel ($MeOH/CH_2Cl_2$ – 0:100 → 5:95). Following **General Procedure VII**, the intermediate triazole (0.05 g, 0.05 mmol) was dissolved in CH_2Cl_2 (2 mL), treated with H_2O (0.02 g, 0.10 mmol) and CF_3COOH (1 mL) followed by work-up with ethereal HCl (2 mL) to give the amine salt **40** (0.03 g, 52% over two steps) as a pale brown solid that rapidly transitioned to a sticky gum. $[\alpha]_D^{23}$ +65.3 (*c* 0.0052, MeOH). ^{1}H NMR (400 MHz, CD_3OD) δ 8.23 (s, 2H, H5/Hγ), 8.13 (d, *J* = 8.2 Hz, 1H, Ar8), 7.90 (d, *J* = 7.5 Hz, 1H, Ar5), 7.55 (ddd, *J* = 8.2, 8.2, 1.6 Hz, 1H, Ar7), 7.52-7.40 (m, 2H, Ar6/Ar4), 7.08 (d, *J* = 7.5 Hz, 1H, Ar3), 4.88-4.80 (m, 2H, $OC\underline{H}_A\underline{H}_B$), 4.76-4.66 (m, 1H, H1'), 4.56-4.46 (m, 1H, H1'), 4.34-4.26 (m, 1H, Lys5), 4.10-4.02 (m, 1H, H2'), 3.16-3.06 (m, 2H, H5'), 2.90-2.82 (m, 2H, Lys1), 2.78-2.74 (m, 2H, H1'''), 2.62-2.56 (m, 3H, H1''/δC-C$\underline{H}_2$), 1.80-1.50 (m, 18H, Lys4/Lys2/Lys3/H2'''/H3'''/H3'/H4'/ H2''/H3''/H4''/ H5''/H6''), 1.26-1.08 (m, 5H, H2''/H3''/H4''/H5''/H6''), 0.95 (d, *J* = 4.0 Hz, 6H, H4'''/ H5'''); ^{13}C NMR (101 MHz, CD_3OD) δ 172.9 (βC=O), 169.1 (αC=O), 157.1 (C=N), 150.8 (Ar2), 147.6 (C4), 143.4 (Cδ), 132.5 (Ar8a), 130.4 (Ar4), 129.1 (Ar4a), 128.6 (Ar5), 128.0 (Ar7), 127.0 (Ar8), 126.8 (C5), 125.1 (Cγ), 120.3 (Ar6), 119.1 (Ar3), 113.9 (Ar1), 67.5 ($O\underline{C}H_AH_B$), 65.5 (C1'), 55.7 (Lys5), 53.5 (C2'), 49.3 (C5'), 40.5 (Lys1), 39.0 (C2'''), 38.0 (C1''), 37.3 (δC-$\underline{C}H_2$), 32.3 (C2''), 32.2 (C6''), 30.8 (Lys4), 30.7 (Lys2), 28.1 (C4''), 27.5 (C1'''), 26.5 (C3'), 25.7 (C3''), 25.6 (C5''), 24.8 (C3'''), 22.7 (C4'), 22.6 (C4'''/C5'''), 21.4 (Lys3); IR (neat) $\bar{\nu}_{max}$ 3346, 3266, 3193, 3063, 2950, 2927, 2855, 1664, 1602, 1543, 1514, 1483, 1451, 1383, 1367, 1349, 1280, 1219, 1168, 1154, 1117, 1080, 1050, 963, 933, 916, 863, 815, 779, 749, 673, 648, 586 cm^{-1}; MS (ESI +ve) *m/z* 757 ([M – 2HCl + H]$^+$, 50%), 379 ([M – 2HCl + H]$^{2+}$, 100%); HRMS (ESI +ve TOF) calcd for $C_{40}H_{61}N_{12}O_3$ 757.4990, found 757.4969 ([M – 2HCl + H]$^+$).

(*R*)-6-Amino-*N*-((*R*)-1-(4-benzyl-1*H*-1,2,3-triazol-1-yl)-5-guanidinopentan-2-yl)-2-(2-((1-(4-isopentyl-1*H*-1,2,3-triazol-1-yl)naphthalen-2-yl)oxy)acetamido)hexanamide dihydrochloride (42)

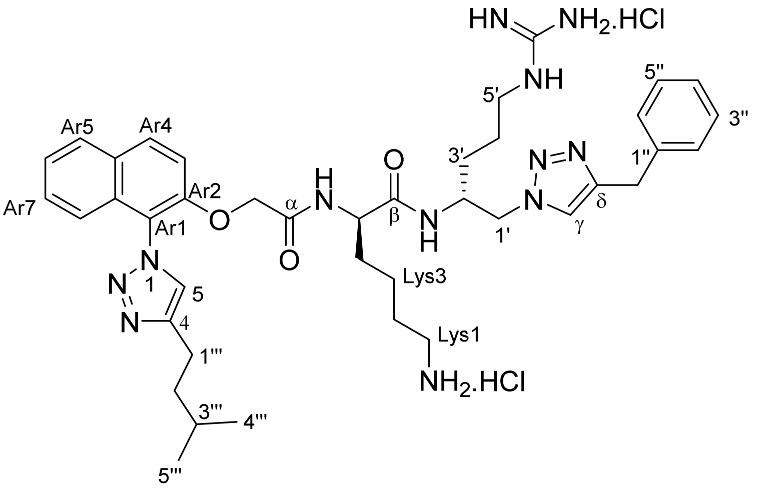

Following **General Procedure IV**, azide **32** (0.07 g, 0.07 mmol), 3-phenyl-1-propyne (0.02 g, 0.21 mmol), $CuSO_4{\cdot}5H_2O$ (0.004 g, 0.01 mmol) and sodium ascorbate (0.006 g, 0.02 mmol) were stirred in *t*-BuOH (2 mL) and H_2O (0.5 mL) for 16 h to give the intermediate triazole as a pale brown solid after flash chromatography over silica gel ($MeOH/CH_2Cl_2$ – 0:100 → 6:94). Following **General Procedure VII**, the intermediate triazole (0.060 g, 0.05 mmol) was dissolved in CH_2Cl_2 (2 mL), treated with H_2O (0.02 g, 1.08 mmol) and CF_3COOH (1 mL) followed by work-up with ethereal HCl (3 mL) to give the amine salt **42** (0.03 g, 52% over two steps) as a brown solid that rapidly transitioned to a sticky gum. $[\alpha]_D^{23}$ +57.3 (*c* 0.0042, MeOH). ^{1}H NMR (400 MHz, CD_3OD) δ 8.41 (brs, 1H, H5), 8.28-8.16 (m, 2H, H2''/H6''), 7.99 (brs, 1H, Hγ), 7.66-7.48 (m, 4H, Ar8/ Ar5/Ar7/Ar6), 7.34-7.22 (m, 4H, H3''/H5''/Ar4/Ar3), 7.20-7.14 (m, 1H, H4''), 4.92-4.80 (m, 2H, OC$\underline{H}_A\underline{H}_B$), 4.74-4.66 (m, 1H, H1'), 4.60-4.52 (m, 1H, H1'), 4.42-4.32 (m, 1H, Lys5), 4.28-4.14 (m, 3H, C$\underline{H}_2$Ph/H2'), 3.22-3.12 (m, 2H, H5'), 3.02-2.84 (m, 2H, Lys1), 2.84-2.76 (m, 2H, H1'''), 1.82-1.52 (m, 10H, Lys4/Lys2/H3'/H4'/H2'''), 1.50-1.42 (m, 1H, H3'''), 1.34-1.20 (m, 2H, Lys3), 1.01 (d, J = 4.8 Hz, 6H, H4'''/H5'''); ^{13}C NMR (101 MHz, CD_3OD) δ 172.0 (βC=O), 168.1 (αC=O), 156.3 (C=N), 150.0 (Ar2), 149.9 (C4), 143.7 (Cδ), 135.5 (C1''), 132.0 (Ar8a), 129.4 (Ar4), 128.9 (Ar4a), 128.3 (Ar5), 128.0 (Ar7), 127.8 (C2''/C6''), 127.6 (C3''/C5''), 127.2 (Ar8), 126.1 (C5), 125.8 (C4''), 124.3 (Cγ), 119.3 (Ar6), 118.0 (Ar3), 113.1 (Ar1), 66.8 (O$\underline{C}H_AH_B$), 64.7 (C1'), 54.5 (Lys5), 52.6 (C2'), 48.5 (C5'), 39.7 (Lys1), 38.2 (C2'''), 36.9 ($\underline{C}H_2$Ph), 30.1 (Lys4), 28.7 (Lys2), 27.4 (C1'''), 26.7 (C3'), 25.7 (C3'''), 24.0 (C4'), 21.6 (C4'''/C5'''), 20.6 (Lys3); IR (neat) $\bar{\nu}_{max}$ 3349, 3266, 3195, 3063, 2954, 2869, 1662, 1602, 1545, 1515, 1497, 1484, 1454, 1382, 1367, 1347, 1279, 1232, 1221, 1166, 1153, 1116, 1079, 1050, 969, 926, 909, 865, 812, 779, 750, 722, 699, 675, 667, 647 cm^{-1}; MS (ESI +ve) *m/z* 751 ([M – 2HCl + H]$^+$, 20%), 376 ([M – 2HCl + H]$^{2+}$, 100%); HRMS (ESI +ve TOF) calcd for $C_{40}H_{56}N_{12}O_3Cl$ 787.4287, found 787.4312 ([M – HCl + H]$^+$).

(2*R*)-6-Amino-*N*-((*R*)-1-(4-cyclohexyl-1*H*-1,2,3-triazol-1-yl)-5-guanidinopentan-2-yl)-2-(2-((1-(5-isopentyl-1*H*-1,2,3-triazol-1-yl)naphthalen-2-yl)oxy)acetamido)hexanamide dihydrochloride (43)

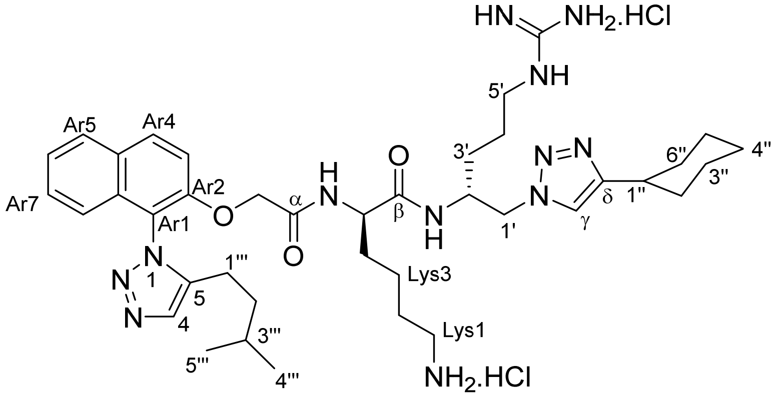

Following **General Procedure IV**, azide **33** (0.08 g, 0.08 mmol), cyclohexylacetylene (0.03 g, 0.24 mmol), $CuSO_4{\cdot}5H_2O$ (0.004 g, 0.02 mmol) and sodium ascorbate (0.006 g, 0.04 mmol) were stirred in *t*-BuOH (2 mL) and H_2O (0.5 mL) for 16 h to give the intermediate triazole as an off-white gum after flash chromatography over silica gel ($MeOH/CH_2Cl_2$ – 0:100 → 5:95). Following **General Procedure VII**, the intermediate triazole (0.07 g, 0.06 mmol) was dissolved in CH_2Cl_2 (2 mL), treated with H_2O (0.02 g, 1.27 mmol) and CF_3COOH (1 mL) followed by work-up with ethereal HCl (3 mL) to give the amine salt **43** (0.036 g, 55% over two steps) as a pale brown solid that rapidly transitioned to a sticky gum. $[\alpha]_D^{23}$ +71.9 (*c* 0.0052, MeOH). ^{1}H NMR (400 MHz, CD_3OD) δ 8.25 (s, 1H, H4), 8.25-8.21 (m, 1H, Ar8), 7.99 (s, 1H, Hγ), 8.03-7.97 (m, 1H, Ar5), 7.64 (ddd, *J* = 9.2, 2.3 Hz, 1H, Ar7), 7.56-7.50 (m, 2H, Ar6/Ar4), 6.99-6.95 (m, 1H, Ar3), 5.01-4.65 (m, 3H, OC$\underline{H}_A\underline{H}_B$/H1'), 4.58-4.48 (m, 1H, H1'), 4.40-4.28 (m, 1H, Lys5), 4.12-4.04 (m, 1H, H2'), 3.23-3.12 (m, 2H, H5'), 3.00-2.76 (m, 2H, H1'''), 2.72-2.63 (m, 1H, H1''), 2.54-2.44 (m, 2H, Lys1), 2.08-1.91 (m, 2H, Lys4), 1.90-1.08 (m, 21H, H2'''/H3'''/H3'/H4'/Lys2/Lys3/H2''/H3''/H4''/H5''/H6''), 0.71-0.66 (m, 6H, H4'''/H5'''); ^{13}C NMR (101 MHz, CD_3OD) δ 176.7 (βC=O), 172.6 (αC=O), 161.0 (C=N), 155.3 (Ar2), 146.0 (Cδ), 136.7 (C5), 136.6 (Ar8a), 135.3 (C4), 134.7 (Ar4), 133.3 (Ar4a), 132.5 (Ar5), 132.1 (Ar7), 129.0 (Ar8), 128.1 (Ar6), 124.2 (Cγ), 121.2 (Ar3), 118.0 (Ar1), 71.3 (O$\underline{C}H_AH_B$), 58.8 (C1'), 57.4 (Lys5), 53.2 (C2'), 44.4 (C2'''), 43.0 (C5'), 40.5 (Lys1), 37.9 (C1''), 36.0 (Lys4), 35.9 (C3'), 34.9 (Lys2), 30.9 (C2''), 30.5 (C6''), 29.4 (C4''), 29.3 (C3''), 29.2 (C5''), 28.7 (C3'''), 26.4 (1'''), 26.3 (C4'), 24.9 (C4'''/C5'''), 24.4 (Lys3); IR (neat) $\bar{\nu}_{max}$ 3343, 3264, 3185, 3063, 2931, 2860, 1663, 1601, 1541, 1513, 1483, 1451, 1385, 1367, 1347, 1278, 1220, 1170, 1154, 1131, 1082, 1049, 990, 973, 931, 919, 859, 818, 779, 750, 678, 652 cm^{-1}; MS (ESI +ve) *m/z* 744 ([M – 2HCl + H]$^+$, 100%); HRMS (ESI +ve TOF) calcd for $C_{39}H_{59}N_{12}O_3$ 743.4833, found 743.4839 ([M – 2HCl + H]$^+$).

(2*R*)-6-Amino-*N*-((*R*)-1-(4-(cyclohexylmethyl)-1*H*-1,2,3-triazol-1-yl)-5-guanidinopen-tan-2-yl)-2-(2-((1-(5-isopentyl-1*H*-1,2,3-triazol-1-yl)naphthalen-2-yl)oxy)acetamido)hexanamide dihydrochloride (44)

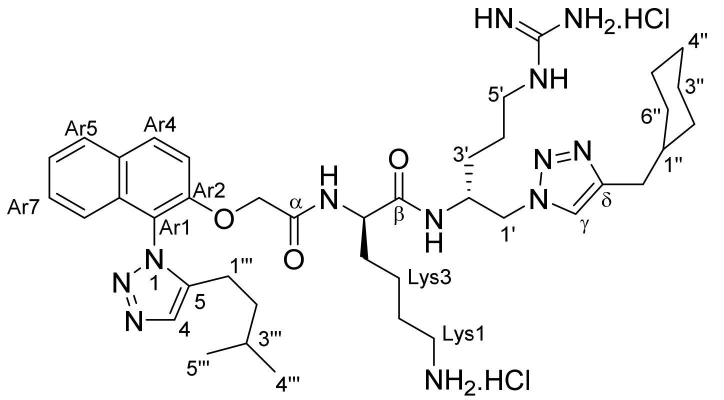

Following **General Procedure IV**, azide **33** (0.08 g, 0.08 mmol), 3-cyclohexyl-1-propyne (0.03 g, 0.24 mmol), $CuSO_4{\cdot}5H_2O$ (0.004 g, 0.02 mmol) and sodium ascorbate (0.006 g, 0.04 mmol) were stirred in *t*-BuOH (2 mL) and H_2O (0.5 mL) for 16 h to give the intermediate triazole as an off-white gum after flash chromatography over silica gel

(MeOH/CH_2Cl_2 – 0:100 → 5:95). Following **General Procedure VII**, the intermediate triazole (0.06 g, 0.05 mmol) was dissolved in CH_2Cl_2 (2 mL), treated with H_2O (0.02 g, 1.05 mmol) and CF_3COOH (1 mL) followed by work-up with ethereal HCl (3 mL) to give the amine salt **44** (0.036 g, 54% over two steps) as a brown solid that rapidly transitioned to a sticky gum. $[\alpha]_D^{23}$ +66.1 (*c* 0.0050, MeOH). ^1H NMR (400 MHz, CD_3OD) δ 8.33 (s, 1H, H4), 8.29 (s, 1H, Hγ), 8.25-8.21 (m, 1H, Ar8), 8.06-8.00 (m, 1H, Ar5), 7.64 (ddd, *J* = 9.2, 9.2, 2.3 Hz, 1H, Ar7), 7.58-7.49 (m, 2H, Ar6/Ar4), 7.00-6.94 (m, 1H, Ar3), 5.04-4.68 (m, 3H, $OC\underline{H}_A\underline{H}_B$/H1'), 4.62-4.48 (m, 1H, H1'), 4.40-4.28 (m, 1H, Lys5), 4.12-4.04 (m, 1H, H2'), 3.23-3.10 (m, 2H, H5'), 3.01-2.85 (m, 1H, H1'''), 2.77-2.58 (m, 3H, H1'''/Lys1), 2.54-2.44 (m, 2H, δC-$C\underline{H}_2$), 1.85-1.49 (m, 14H, H2'''/H3'''/H3'/H4'/Lys2/Lys3/ Lys4/H1''), 1.46-1.08 (m, 8H, H2''/H3''/H4''/H5''/H6''), 1.03-0.80 (m, 2H, H3''/H5''), 0.74-0.64 (m, 6H, H4'''/H5'''); ^{13}C NMR (101 MHz, CD_3OD) δ 171.5 (βC=O), 167.3 (αC=O), 157.9 (C=N), 151.9 (Ar2), 145.5 (Cδ), 141.1 (C5), 132.4 (Ar8a), 131.7 (C4), 131.4 (Ar4), 129.2 (Ar4a), 128.7 (Ar5), 128.5 (Ar7), 125.3 (Ar8), 123.4 (Ar6), 121.1 (Cγ), 118.4 (Ar3), 115.5 (Ar1), 68.4 ($O\underline{C}H_AH_B$), 53.1 (C1'), 49.5 (Lys5), 39.1 (C2'), 37.86 (C2'''), 37.83 (C5'), 36.9 (Lys1), 32.99 (δC-$\underline{C}H_2$), 32.92 (C1''), 31.8 (Lys4), 29.1 (C3'), 27.1 (Lys2), 26.98 (C2''), 26.95 (C6''), 26.4 (C4''), 26.03 (C3''), 26.01 (C5''), 25.3 (C3'''), 22.5 (C1'''), 22.4 (C4'), 22.2 (C4'''/C5'''), 20.7 (Lys3); IR (neat) $\bar{\nu}_{max}$ 3345, 3264, 3187, 3061, 2952, 2927, 2855, 2666, 1663, 1634, 1602, 1542, 1513, 1483, 1450, 1385, 1368, 1347, 1279, 1220, 1168, 1154, 1131, 1082, 1050, 1025, 992, 972, 932, 863, 818, 780, 749, 680, 651 cm^{-1}; MS (ESI +ve) *m/z* 758 ($[M – 2HCl + H]^+$, 70%), 380 ($[M – 2HCl + H]^{2+}$, 100%); HRMS (ESI +ve TOF) calcd for $C_{40}H_{61}N_{12}O_3$ 757.4990, found 757.5007 ($[M – 2HCl + H]^+$).

(2*R*)-6-Amino-*N*-((*R*)-1-(4-benzyl-1*H*-1,2,3-triazol-1-yl)-5-guanidinopentan-2-yl)-2-(2-((1-(5-isopentyl-1*H*-1,2,3-triazol-1-yl)naphthalen-2-yl)oxy)acetamido)hexanamide dihydrochloride (45)

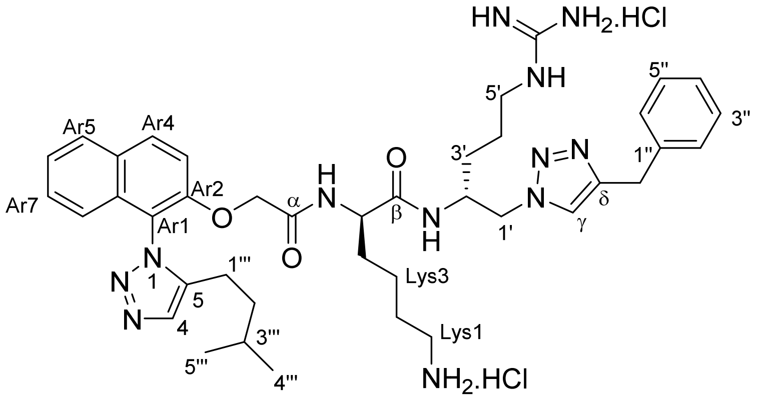

Following **General Procedure IV**, azide **33** (0.08 g, 0.08 mmol), 3-phenyl-1-propyne (0.03 g, 0.24 mmol), $CuSO_4{\cdot}5H_2O$ (0.004 g, 0.02 mmol) and sodium ascorbate (0.006 g, 0.04 mmol) were stirred in *t*-BuOH (2 mL) and H_2O (0.5 mL) for 16 h to give the intermediate triazole as an off-white gum after flash chromatography over silica gel (MeOH/CH_2Cl_2 – 0:100 → 6:94). Following **General Procedure VII**, the intermediate triazole (0.07 g, 0.06 mmol) was dissolved in CH_2Cl_2 (2 mL), treated with H_2O (0.02 g, 1.27 mmol) and CF_3COOH (1 mL) followed by work-up with ethereal HCl (3 mL) to give the amine salt **45** (0.035 g, 53% over two steps) as a white solid that rapidly transitioned to a sticky gum. $[\alpha]_D^{23}$ +58.9 (*c* 0.0052, MeOH). ^1H NMR (400 MHz, CD_3OD) δ 8.25-8.18 (m, 1H, Ar8), 8.03-7.93 (m, 2H, Ar5/Ar7), 7.95 (s, 1H, H4), 7.63-7.50 (m, 3H, Ar6/Ar4/Hγ), 7.33-7.17 (m, 5H,

H2''/H3''/H4''/H5''/H6''), 6.99-6.95 (m, 1H, Ar3), 4.99-4.68 (m, 2H, OC<u>H</u>A<u>H</u>B), 4.62-4.55 (m, 1H, H1'), 4.49-4.39 (m, 1H, H1'), 4.36-4.27 (m, 1H, Lys5), 4.17-4.02 (m, 3H, H2'/δC-C<u>H</u>2), 3.18-3.12 (m, 2H, H5'), 2.92-2.84 (m, 1H, H1'''), 2.77-2.67 (m, 1H, H1'''), 2.58-2.42 (m, 2H, Lys1), 1.77-1.44 (m, 8H, H3'/H4'/Lys2/Lys4), 1.43-1.30 (m, 3H, H2'''/H3'''), 1.28-1.00 (m, 2H, Lys3), 0.71-0.66 (m, 6H, H4'''/H5'''); ^{13}C NMR (101 MHz, CD_3OD) δ 171.5 (βC=O), 167.2 (αC=O), 157.9 (C=N), 151.8 (Ar2), 146.0 (Cδ), 141.2 (C5), 141.1 (Ar8a), 140.0 (C4), 132.0 (C1''), 131.7 (Ar4), 131.4 (Ar4a), 129.2 (C2''), 128.9 (C6''), 128.79 (C3''), 128.74 (C5''), 128.5 (Ar5), 126.48 (Ar7), 126.46 (Ar8), 125.3 (Ar6), 125.2 (C4''), 123.4 (Cγ), 121.1 (Ar3), 115.5 (Ar1), 68.4 (O<u>C</u>HAHB), 53.0 (C1'), 49.5 (Lys5), 39.1 (C2'), 36.9 (C2'''), 31.8 (δC-<u>C</u>H2), 29.1 (C5'), 27.1 (Lys1), 26.99 (Lys4), 26.96 (C3'), 25.3 (Lys2), 22.5 (C3'''), 22.4 (1'''), 22.23 (C4'), 22.20 (C4'''/C5'''), 20.7 (Lys3); IR (neat) $\bar{\nu}_{max}$ 3342, 3264, 3190, 3064, 2955, 2935, 2869, 1662, 1601, 1543, 1513, 1483, 1455, 1384, 1367, 1346, 1279, 1221, 1170, 1154, 1131, 1080, 1049, 991, 972, 930, 864, 815, 780, 750, 723, 698, 678, 652 cm^{-1}; MS (ESI +ve) *m/z* 752 ([M – 2HCl + H]$^+$, 100%); HRMS (ESI +ve TOF) calcd for $C_{40}H_{55}N_{12}O_3$ 751.4520, found 751.4544 ([M – 2HCl + H]$^+$).

(*R*)-6-Amino-*N*-((*R*)-1-(4-benzyl-1*H*-1,2,3-triazol-1-yl)-5-guanidinopentan-2-yl)-2-(2-(2-(1-benzyl-1*H*-1,2,3-triazol-4-yl)phenoxy)acetamido)hexanamide dihydrochloride (46)

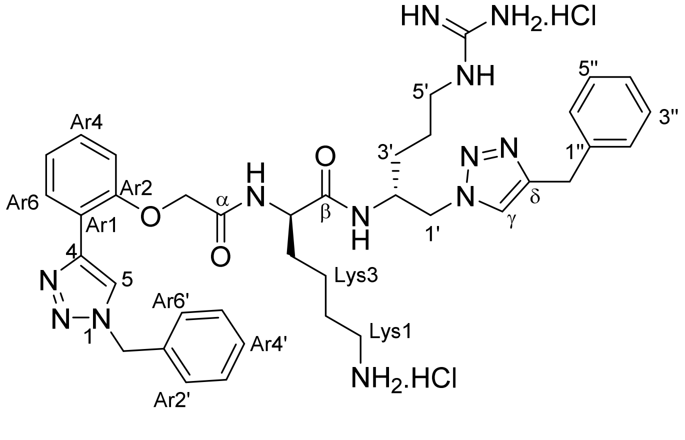

Following **General Procedure IV**, azide **34** (0.07 g, 0.07 mmol), 3-phenyl-1-propyne (0.03 g, 0.22 mmol), $CuSO_4{\cdot}5H_2O$ (0.004 g, 0.01 mmol) and sodium ascorbate (0.006 g, 0.03 mmol) were stirred in *t*-BuOH (2 mL) and H_2O (0.5 mL) for 16 h to give the intermediate triazole as an off-white solid after flash chromatography over SiO_2 gel ($MeOH/CH_2Cl_2$ – 0:100 → 6:94). Following **General Procedure VII**, the intermediate triazole (0.06 g, 0.056 mmol) was dissolved in CH_2Cl_2 (2 mL), treated with H_2O (0.02 g, 1.11 mmol) and CF_3COOH (1 mL) followed by work-up with ethereal HCl (2 mL) to give the amine salt **46** (0.03 g, 55% over two steps) as a pale brown solid that rapidly transitioned to a sticky gum. $[\alpha]_D^{23}$ +65.3 (*c* 0.0052, MeOH). ^1H NMR (400 MHz, CD_3OD) δ 8.74 (s, 1H, H5), 8.23-7.83 (m, 2H, Hγ/Ar4), 7.50-7.14 (m, 13H, Ar6/Ar5/Ar3/Ar2'/Ar3'/Ar4'/Ar5'/Ar6'/H2"/H3"/H4"/H5"/H6"), 5.69 (s, 2H, C<u>H</u>$_2$Ph), 4.92-4.76 (m, 2H, OC<u>H</u>$_A$<u>H</u>$_B$), 4.84-4.70 (m, 2H, H1'), 4.40-4.32 (m, 1H, Lys5), 4.30-4.22 ((m, 1H, H2'), 4.03 (s, 2H, δC-C<u>H</u>$_2$), 3.23-3.07 (m, 2H, H5'), 2.95-2.78 (m, 2H, Lys1), 1.80-1.50 (m, 8H, Lys4/H3'/Lys2 /H4'), 1.36-1.20 (m, 2H, Lys3); ^{13}C NMR (101 MHz, CD_3OD) δ 174.5 (βC=O), 169.7 (αC=O), 158.7 (C=N), 156.0 (Ar2), 143.1 (C4), 139.0 (Ar1'), 136.5 (C1"), 131.7 (Ar6), 130.3 (Cδ), 130.0 (C5), 129.9 (Ar4), 129.8 (C2"/C6"), 129.5 (Ar3'/Ar5'/C3"/ C5"), 129.4 (Ar2'/Ar6'), 128.9 (Ar4'/C4"), 128.1 (Cγ), 123.4 (Ar5), 119.3 (Ar1), 114.4 (Ar3), 69.0 (O<u>C</u>H$_A$H$_B$), 56.1 (C1'), 55.9 (Lys5), 55.0 (<u>C</u>H$_2$Ph), 50.9 (C2'), 42.1 (C5'), 40.7 (Lys1), 32.5 (δC-<u>C</u>H$_2$), 31.8 (Lys4), 30.0 (C3'), 28.1 (Lys2), 26.4 (C4'), 24.0 (Lys3); IR (neat) $\bar{\nu}_{max}$ 3351, 3265, 3203, 3065, 2950, 2870, 1658, 1585, 1546, 1490, 1455, 1441, 1360, 1290, 1229, 1181, 1165, 1125, 1075, 1053, 978, 907, 840, 804, 758, 725, 698, 670, 644, 586 cm^{-1}; MS (ESI +ve) *m/z* 721 ($[M – 2HCl + H]^+$, 10%), 361 ($[M – 2HCl + H]^{2+}$, 100%); HRMS (ESI +ve TOF) calcd for $C_{38}H_{49}N_{12}O_3$ 721.4045, found 721.4046 ($[M – 2HCl + H]^+$).

(*R*)-6-Amino-2-(2-(2-(1-benzyl-1*H*-1,2,3-triazol-4-yl)phenoxy)acetamido)-*N*-((*R*)-1-(4-(cyclohexylmethyl)-1*H*-1,2,3-triazol-1-yl)-5-guanidinopentan-2-yl)hexanamide dihydrochloride (47)

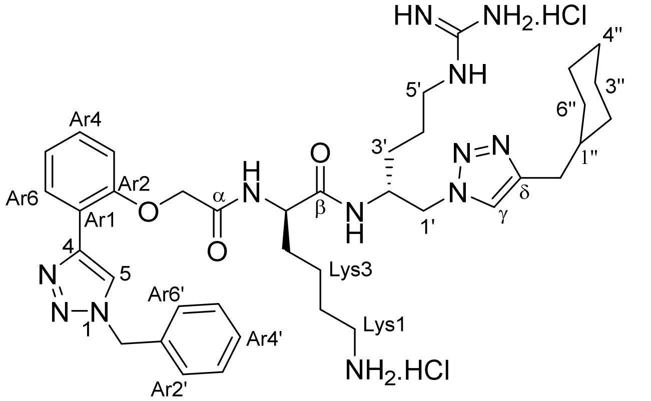

Following **General Procedure IV**, azide **34** (0.09 g, 0.09 mmol), 3-cyclohexyl-1-propyne (0.03 g, 0.28 mmol), $CuSO_4{\cdot}5H_2O$ (0.005 g, 0.02 mmol) and sodium ascorbate (0.007 g, 0.04 mmol) were stirred in *t*-BuOH (2 mL) and H_2O (0.5 mL) for 16 h to give the intermediate triazole as an off-white gum after flash chromatography over silica gel (MeOH/CH_2Cl_2 – 0:100 → 6:94). Following **General Procedure VII**, the intermediate triazole (0.06 g, 0.055 mmol) was dissolved in CH_2Cl_2 (2 mL), treated with H_2O (0.02 g, 1.10 mmol) and CF_3COOH (1 mL) followed by work-up with ethereal HCl (2 mL) to give the amine salt **47** (0.025 g, 35% over two steps) as a pale-yellow solid that rapidly transitioned to a sticky gum. $[\alpha]_D^{23}$ +57.6 (*c* 0.0054, MeOH). ^{1}H NMR (400 MHz, CD_3OD) δ 8.82 (s, 1H, H5), 8.25 (s, 1H, Hγ), 7.91 (apparent t, *J* = 7.0 Hz, 1H, Ar4), 7.44-7.36 (m, 6H, Ar6/Ar5/Ar2'/Ar3'/Ar5'/ Ar6'), 7.16-7.10 (m, 2H, Ar4'/Ar3), 5.75 (s, 2H, C<u>H</u>2Ph), 4.88-4.70 (m, 3H, OC<u>H</u>A<u>H</u>B/H1'), 4.66-4.50 (m, 1H, H1'), 4.46-4.32 (m, 1H, Lys5), 4.28-4.19 (m, 1H, H2'), 3.24-3.10 (m, 2H, δC-C<u>H</u>2), 2.92-2.84 (m, 2H, H5'), 2.66-2.60 (m, 2H, Lys1), 1.82-1.58 (m, 14H, Lys4/H3'/ Lys2/H4'/H1"/H2"/H3"/H4"/H5"/H6"), 1.44-1.28 (m, 2H, H2"/H6"), 1.26-1.12 (m, 3H, H3"/H4"/ H5"), 0.94-0.86 (m, 2H, Lys3); ^{13}C NMR (101 MHz, CD_3OD) δ 174.5 (βC=O), 171.6 (αC=O), 158.7 (C=N), 156.1 (Ar2), 145.1 (C4), 144.3 (Ar1'), 136.5 (Cδ), 131.8 (Ar6), 130.3 (C5/Ar4), 130.0 (Ar3'/Ar5'), 129.5 (Ar4'), 128.5 (Ar2'/Ar6'), 126.4 (Cγ), 123.3 (Ar5), 119.1 (Ar1), 114.4 (Ar3), 68.8 (O<u>C</u>HAHB), 57.2 (C1'), 55.9 (Lys5), 55.3 (<u>C</u>H2Ph), 50.9 (C2'), 42.1 (C5'), 40.7 (Lys1), 38.9 (C1"), 33.9 (C2"/C6"), 33.8 (δC-<u>C</u>H2), 32.4 (Lys4), 32.3 (C4"), 29.7 (C3'), 28.1 (Lys2), 27.3 (C3"), 27.1 (C5"), 26.4 (C4'), 24.3 (Lys3); IR (neat) $\bar{\nu}_{max}$ 3348, 3266, 3192, 3066, 2927, 2854, 2667, 1659, 1586, 1545, 1490, 1450, 1382, 1351, 1227, 1167, 1126, 1076, 1050, 977, 937, 911, 846, 805, 760, 725, 696, 675, 647, 585 cm^{-1}; MS (ESI +ve) *m/z* 727 ([M – 2HCl + H]$^+$, 20%), 364 ([M – 2HCl + H]$^{2+}$, 100%); HRMS (ESI +ve TOF) calcd for $C_{38}H_{55}N_{12}O_3$ 727.4514, found 727.4513 ([M – 2HCl + H]$^+$).

(*R*)-6-Amino-*N*-((*R*)-1-(4-cyclohexyl-1*H*-1,2,3-triazol-1-yl)-5-guanidinopentan-2-yl)-2-(2-((1-(2-(isopentyloxy)phenyl)-1*H*-1,2,3-triazol-5-yl)methoxy)acetamido)hexanamide dihydrochloride (48)

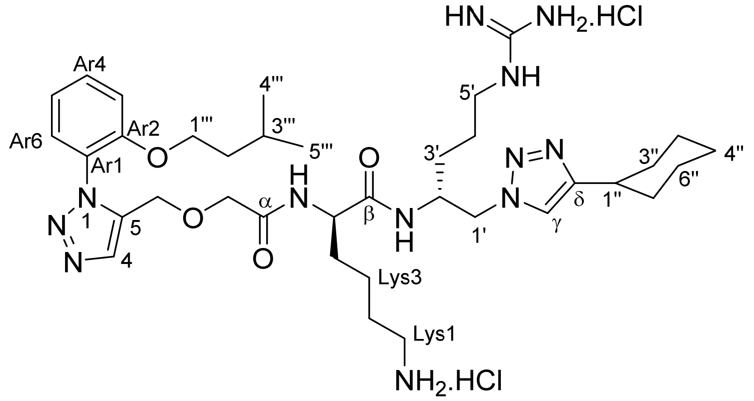

Following **General Procedure IV**, azide **35** (0.07 g, 0.07 mmol), cyclohexylacetylene (0.02 g, 0.21 mmol), $CuSO_4{\cdot}5H_2O$ (0.003 g, 0.01 mmol) and sodium ascorbate (0.006 g, 0.02 mmol) were stirred in *t*-BuOH (2 mL) and H_2O (0.5 mL) for 16 h to give the intermediate triazole as an off-white gum after flash chromatography over silica gel ($MeOH/CH_2Cl_2$ – 0:100 → 6:94). Following **General Procedure VII**, the intermediate triazole (0.06 g, 0.05 mmol) was dissolved in CH_2Cl_2 (2 mL), treated with H_2O (0.02 g, 1.11 mmol) and CF_3COOH (1 mL) followed by work-up with ethereal HCl (3 mL) to give the amine salt **48** (0.034 g, 61% over two steps) as a light brown solid that rapidly transitioned to a sticky gum. $[\alpha]_D^{23}$ +57.1 (*c* 0.0052, MeOH). ^{1}H NMR (400 MHz, CD_3OD) δ 8.05 (s, 2H, H4/Hγ), 7.57 (ddd, *J* = 8.0, 8.0, 3.0 Hz, 1H, Ar4), 7.45-7.42 (m, 1H, Ar6), 7.27-7.25 (m, 1H, Ar5), 7.16-7.11 (m, 1H, Ar3), 4.66 (m, 3H, C5-C<u>H</u>2/H1'), 4.46 (apparent t, *J* = 10.5 Hz, 1H, H1'), 4.40-4.31 (m, 1H, Lys5), 4.19-4.15 (m, 1H, H2'), 4.06 (t, *J* = 6.0 Hz, 2H, H1'''), 3.88 (s, 2H, OC<u>H</u>A<u>H</u>B), 3.20-3.17 (m, 2H, H5'), 2.93-2.90 (m, 2H, Lys1), 2.84-2.74 (m, 1H, H1''), 2.08-1.96 (m, 2H, H3'), 1.86-1.79 (m, 2H, Lys4), 1.78-1.55 (m, 9H, Lys2/H4'/H2''/H3''/H4''/H5''/H6''), 1.54-1.37 (m, 8H, H2'''/H3'''/H2''/H3''/ H4''/H5''/H6''), 1.35-1.18 (m, 2H, Lys3), 0.82 (d, *J* = 6.2 Hz, 6H, H4'''/H5'''); ^{13}C NMR (101 MHz, CD_3OD) δ 172.6 (βC=O), 170.2 (αC=O), 157.2 (C=N), 153.5 (Ar2), 132.06 (C5), 132.02 (Cδ), 128.4 (C4), 124.8 (Ar4), 120.66 (Ar6), 120.61 (Cγ), 115.7 (Ar1), 113.3 (Ar5), 111.8 (Ar3), 68.8 (O<u>C</u>HAHB), 67.2 (C1'''), 61.5 (C5-<u>C</u>H2), 54.2 (C1'), 52.8 (Lys5), 49.1 (C2'), 40.5 (C5'), 39.1 (Lys1), 37.3 (C2'''), 34.5 (C1''), 32.3 (C2''/C6''), 31.3 (Lys4), 28.5 (C3'), 26.6 (Lys2), 25.6 (C4''), 25.4 (C3''/C5''), 24.8 (C3'''), 24.7 (C4'), 22.3 (C4'''/C5'''), 21.3 (Lys3); IR (neat) $\bar{\nu}_{max}$ 3396, 3372, 3358, 3285, 3274, 3263, 3212, 3198, 3067, 2931, 2865, 2162, 2126, 1978, 1965, 1659, 1533, 1509, 1464, 1386, 1368, 1345, 1288, 1247, 1229, 1161, 1129, 1052, 979, 759, 672, 618, 572 cm^{-1}; MS (ESI +ve) *m/z* 723 ($[M - 2HCl + H]^+$, 100%); HRMS (ESI +ve TOF) calcd for $C_{36}H_{59}N_{12}O_4$ 723.4782, found 723.4810 ($[M - 2HCl + H]^+$).

(*R*)-6-Amino-*N*-((*R*)-1-(4-(cyclohexylmethyl)-1*H*-1,2,3-triazol-1-yl)-5-guanidinopen- tan-2-yl)-2-(2-((1-(2-(isopentyloxy)phenyl)-1*H*-1,2,3-triazol-5-yl)methoxy)acetamido)hexanamide dihydrochloride (49)

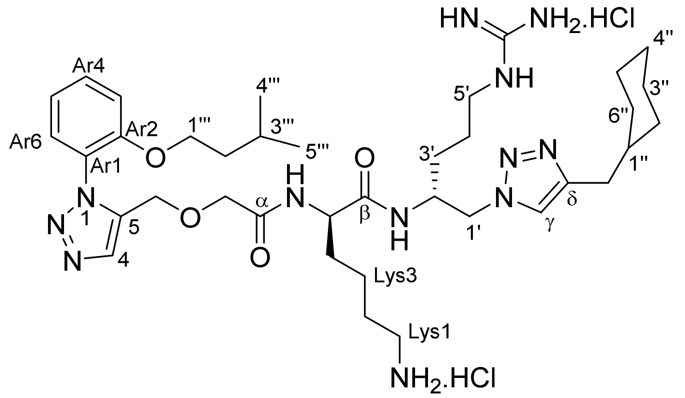

Following **General Procedure IV**, azide **35** (0.07 g, 0.07 mmol), 3-cyclohexyl-1-propyne (0.03 g, 0.21 mmol), $CuSO_4 \cdot 5H_2O$ (0.003 g, 0.01 mmol) and sodium ascorbate (0.006 g, 0.02 mmol) were stirred in *t*-BuOH (2 mL) and H_2O (0.5 mL) for 16 h to give the intermediate triazole as an off-white gum after flash chromatography over silica gel ($MeOH/CH_2Cl_2$ – 0:100 → 5:95). Following **General Procedure VII**, the intermediate triazole (0.06 g, 0.05 mmol) was dissolved in CH_2Cl_2 (2 mL), treated with H_2O (0.02 g, 1.10 mmol) and CF_3COOH (1 mL) followed by work-up with ethereal HCl (3 mL) to give the amine salt **49** (0.037 g, 83% over two steps) as a pale brown solid that rapidly transitioned to a sticky gum. $[\alpha]_D^{23}$ +62.3 (*c* 0.0052, MeOH). ^{1}H NMR (400 MHz, CD_3OD) δ 8.19 (s, 1H, H4), 7.97 (s, 1H, Hγ), 7.61-7.56 (m, 1H, Ar4), 7.46-7.44 (m, 1H, Ar6), 7.27-7.25 (m, 1H, Ar5), 7.15-7.11 (m, 1H, Ar3), 4.72-4.44 (m, 4H, C5-C$\underline{H}_2$/H1'), 4.42-4.26 (m, 1H, Lys5), 4.22-4.12 (m, 1H, H2'), 4.06 (t, *J* = 7.6 Hz, 2H, H1'''), 3.91 (s, 2H, OC$\underline{H}_A\underline{H}_B$), 3.24-3.16 (m, 2H, H5'), 2.97-2.87 (m, 2H, Lys1), 2.70-2.62 (m, 2H, δC-C$\underline{H}_2$), 1.78-1.54 (m, 14H, H3'/Lys2/Lys4/H4'/H1"/H2"/H3"/H4"/H5"/H6"), 1.58-1.44 (m, 3H, H2"/H4"/H6"), 1.38-1.10 (m, 5H, H2'''/H3'''/Lys3), 1.06-0.91 (m, 2H, H3"/H5"), 0.82 (d, *J* = 7.7 Hz, 6H, H4'''/H5'''); ^{13}C NMR (101 MHz, CD_3OD) δ 172.7 (βC=O), 170.2 (αC=O), 157.2 (C=N), 153.6 (Ar2), 132.0 (C5), 128.1 (Cδ), 125.9 (C4), 124.5 (Ar4), 120.67 (Ar6), 120.64 (Cγ), 115.5 (Ar1, Observed by gHMBC), 113.39 (Ar5), 113.36 (Ar3), 68.9 (O$\underline{C}H_AH_B$), 67.3 (C1'''), 61.4 (C5-$\underline{C}H_2$), 55.0 (C1'), 53.1 (Lys5), 52.9 (C2'), 49.3 (δC-$\underline{C}H_2$), 40.5 (C5'), 39.1 (Lys1), 37.6 (C2'''), 37.3 (C1"), 32.4 (C2"), 32.3 (C6"), 31.2 (Lys4), 28.3 (C3'), 26.5 (Lys2), 25.8 (C4"), 25.7 (C3"), 25.6 (C5"), 24.8 (C3'''), 24.7 (C4'), 22.4 (C4'''/C5'''), 21.4 (Lys3); IR (neat) $\bar{\nu}_{max}$ 3381, 3359, 3352, 3333, 3284, 3270, 3260, 3250, 3202, 3074, 3063, 2926, 2869, 2176, 2123, 2061, 1999, 1659, 1529, 1509, 1465, 1450, 1386, 1369, 1288, 1246, 1229, 1137, 1056, 998, 981, 849, 756, 667, 638, 619, 588, 572 cm^{-1}; MS (ESI +ve) *m/z* 737 ([M – 2HCl + H]$^+$, 100%); HRMS (ESI +ve TOF) calcd for $C_{37}H_{61}N_{12}O_4$ 737.4939, found 737.4940 ([M – 2HCl + H]$^+$).

(*R*)-6-Amino-*N*-((*R*)-1-(4-benzyl-1*H*-1,2,3-triazol-1-yl)-5-guanidinopentan-2-yl)-2-(2-((1-(2-(isopentyloxy)phenyl)-1*H*-1,2,3-triazol-5-yl)methoxy)acetamido)hexanamide dihydrochloride (50)

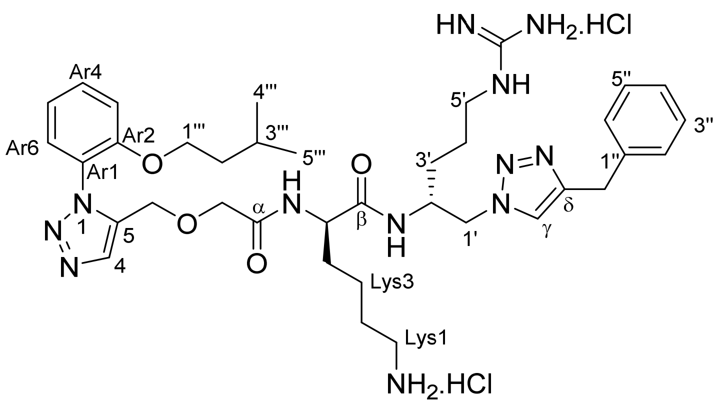

Following **General Procedure IV**, azide **35** (0.08 g, 0.08 mmol), 3-phenyl-1-propyne (0.03 g, 0.24 mmol), $CuSO_4{\cdot}5H_2O$ (0.004 g, 0.01 mmol) and sodium ascorbate (0.006 g, 0.03 mmol) were stirred in *t*-BuOH (2 mL) and H_2O (0.5 mL) for 16 h to give the intermediate triazole as an off-white gum after flash chromatography over silica gel ($MeOH/CH_2Cl_2$ – 0:100 → 5:95). Following **General Procedure VII**, the intermediate triazole (0.06 g, 0.05 mmol) was dissolved in CH_2Cl_2 (2 mL), treated with H_2O (0.02 g, 1.10 mmol) and CF_3COOH (1 mL) followed by work-up with ethereal HCl (3 mL) to give the amine salt **52** (0.033 g, 51% over two steps) as a pale brown solid that rapidly transitioned to a sticky gum. $[\alpha]_D^{23}$ +68.1 (*c* 0.0048, MeOH). ^{1}H NMR (400 MHz, CD_3OD) δ 8.15 (s, 1H, H4), 8.05 (s, 1H, Hγ), 7.58 (t, *J* = 7.7 Hz, 1H, Ar4), 7.45 (d, *J* = 7.6 Hz, 1H, Ar6), 7.33-7.20 (m, 6H, Ar5/H2''/H3''/H4''/ H5''/H6''), 7.13 (d, *J* = 7.5 Hz, 1H, Ar3), 4.73-4.54 (m, 3H, C5-C<u>H</u>2/H1'), 4.56-4.4.45 (m, 1H, H1'), 4.40-4.26 (m, 1H, Lys5), 4.22-4.10 (m, 3H, H2'/OC<u>H</u>A<u>H</u>B), 4.10-4.02 (m, 2H, H5'), 3.91 (t, *J* = 5.9 Hz, 2H, H1'''), 3.25-3.11 (m, 2H, Lys1), 2.98-2.81 (m, 2H, δC-C<u>H</u>2), 1.73-1.34 (m, 11H, H3'/Lys2/Lys4/ H4'/H2'''/H3'''), 1.31-1.16 (m, 2H, Lys3), 0.82 (d, *J* = 6.1 Hz, 6H, H4'''/H5'''); ^{13}C NMR (101 MHz, CD_3OD) δ 172.7 (βC=O), 170.1 (αC=O), 157.2 (C=N), 153.5 (Ar2), 137.09 (C5), 137.03 (Cδ), 132.2 (C1''), 128.67 (C2''), 128.65 (C6''), 128.5 (C3''), 128.4 (C5''), 128.1 (C4), 126.9 (Ar4), 126.8 (C4''), 126.7 (Ar6), 125.8 (Cγ), 124.3 (Ar1), 120.6 (Ar5), 113.3 (Ar3), 68.9 (O<u>C</u>HAHB), 67.3 (C1'''), 61.4 (C5-<u>C</u>H2), 54.9 (C1'), 52.8 (Lys5), 49.2 (C2'), 40.5 (C5'), 39.1 (Lys1), 37.3 (C2'''), 31.2 (δC-<u>C</u>H2), 29.9 (Lys4), 28.3 (C3'), 26.5 (Lys2), 24.8 (C3'''), 24.7 (C4'), 22.5 (C4'''/C5'''), 21.4 (Lys3); IR (neat) $\bar{\nu}_{max}$ 3358, 3273, 3195, 3068, 2955, 2871, 2165, 2101, 2063, 1658, 1533, 1509, 1464, 1386, 1368, 1288, 1245, 1227, 1164, 1137, 1050, 999, 979, 875, 850, 757, 724, 699667, 621, 590 cm^{-1}; MS (ESI +ve) *m/z* 731 ([M – 2HCl + H]$^+$, 100%); HRMS (ESI +ve TOF) calcd for $C_{37}H_{55}N_{12}O_4$ 731.4469, found 731.4444 ([M – 2HCl + H]$^+$).

References

14 Wales, S. M.; Hammer, K. A.; King, A. M.; Tague, A. J.; Lyras, D.; Riley, T. V.; Keller, P. A.; Pyne, S. G. Binaphthyl-1,2,3-triazole peptidomimetics with activity against *Clostridium difficile* and other pathogenic bacteria. *Org. Biomol. Chem.* **2015**, *13*, 5743-5756. doi: 10.1039/c5ob01638j.

32 Zhu, D.; Ma, J.; Luo, K.; Fu, H.; Zhang, L.; Zhu, S. Enantioselective Intramolecular C-H Insertion of Donor and Donor/Donor Carbenes by a Nondiazo Approach. *Angew. Chem., Int. Ed.,* **2016**, *55*, 8452-8456. doi: 10.1002/anie.201604211

33 Maehr H.; Smallheer, J. Total syntheses of rivularins D1 and D3. *J. Am. Chem. Soc.* **1985**, *107*, 2943-2945. https://doi.org/10.1021/ja00296a018

34 Gamble, A. B.; Garner, J.; Gordon, C. P.; O'Conner, S. M. J.; Keller P. A. Aryl Nitro Reduction with Iron Powder or Stannous Chloride under Ultrasonic Irradiation. *Synth. Commun.,* **2007**, *37*, 2777-2786. https://doi.org/10.1080/00397910701481195

35 M. K. Zilla, D. Nayak, R. A. Vishwakarma, P. R. Sharma, A. Goswami, A. Ali A convergent synthesis of alkyne-azide cycloaddition derivatives of 4-α,β-2-propyne podophyllotoxin depicting potent cytotoxic activity. *Eur. J. Med. Chem.* **2014**, 77, 47-55. doi: 10.1016/j.ejmech.2014.02.030

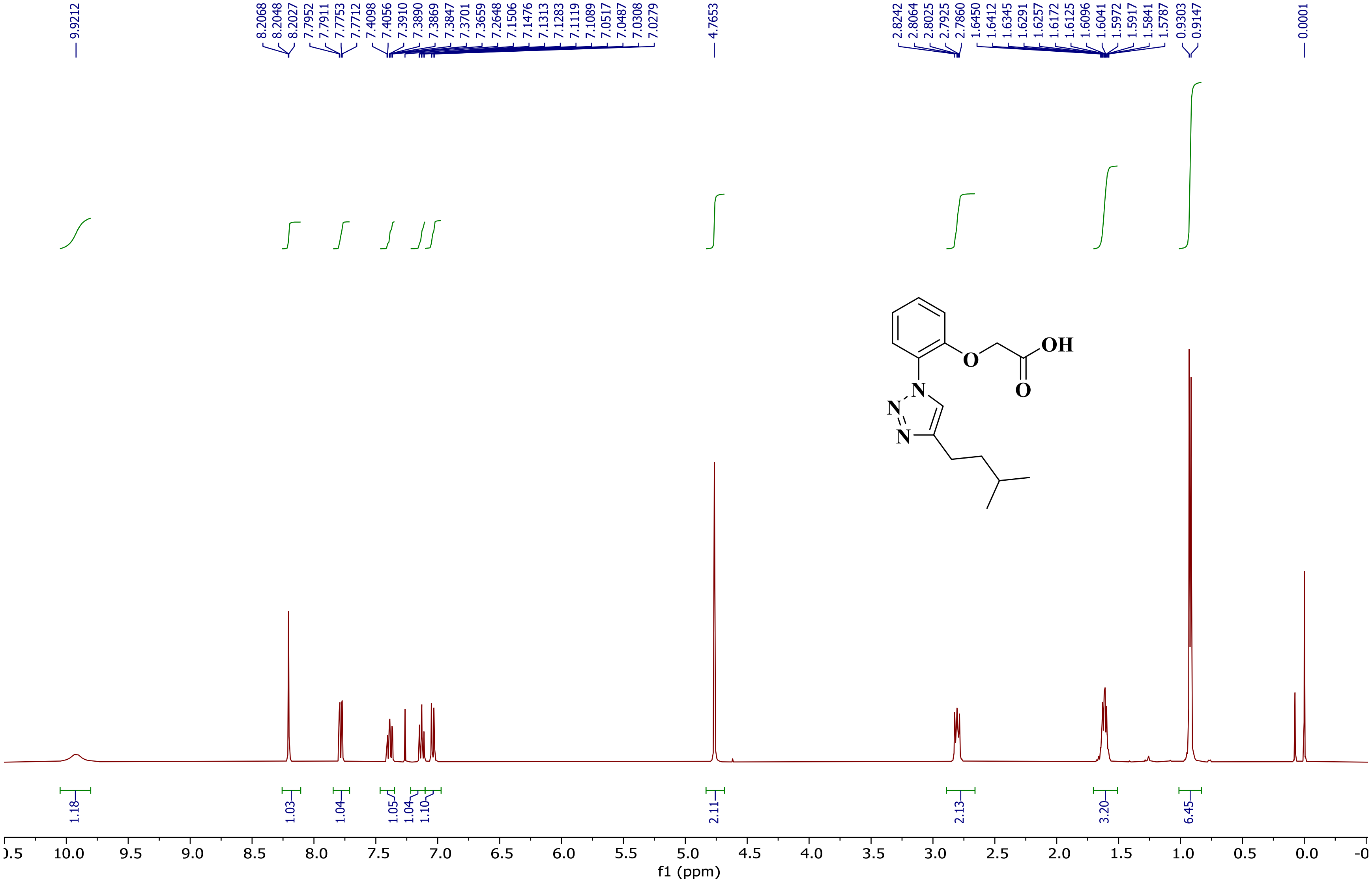

Figure S1: ^{1}H NMR of compound 5 (400 MHz, $CDCl_3$)

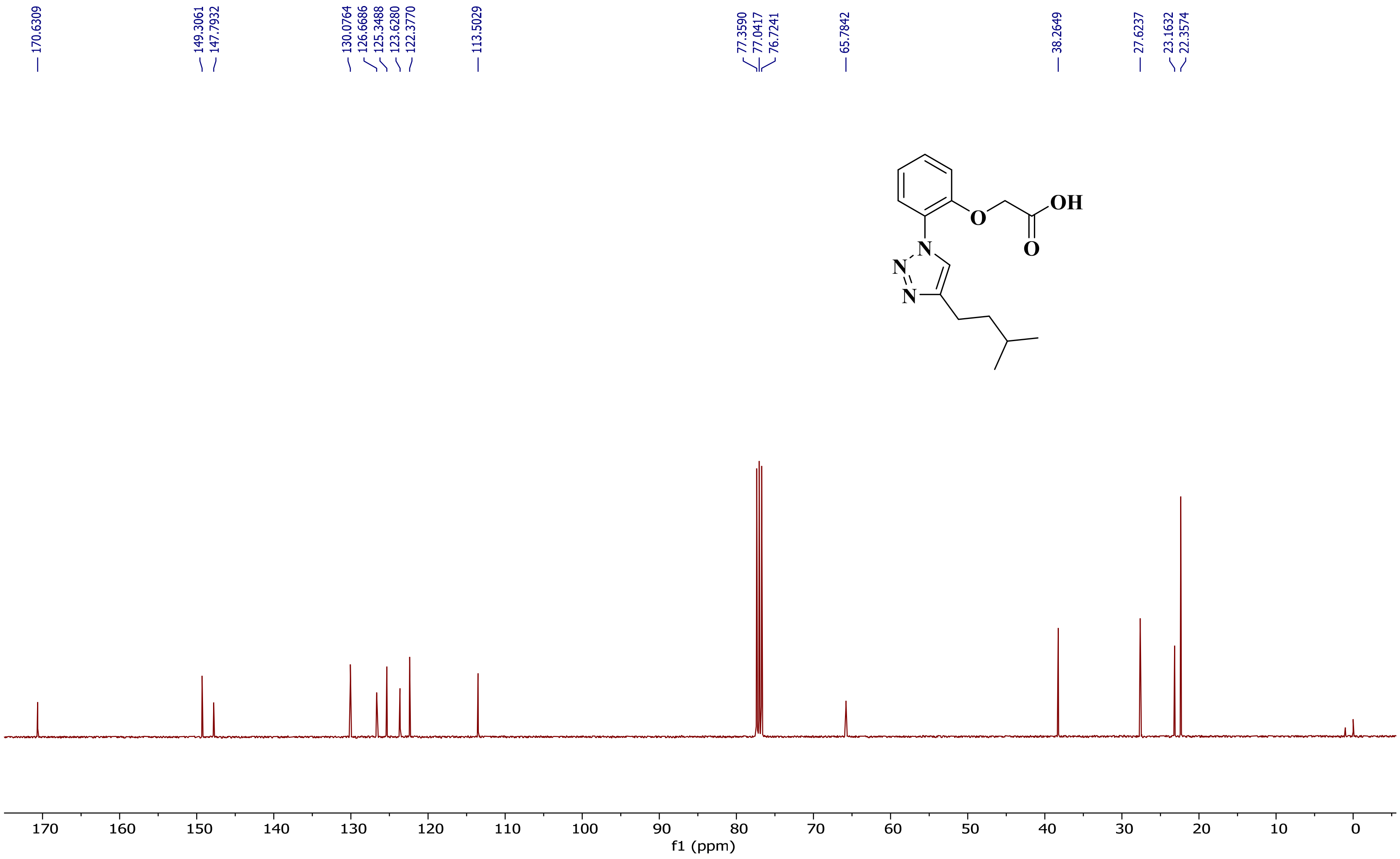

Figure S2: ^{13}C NMR of compound 5 (101 MHz, $CDCl_3$)

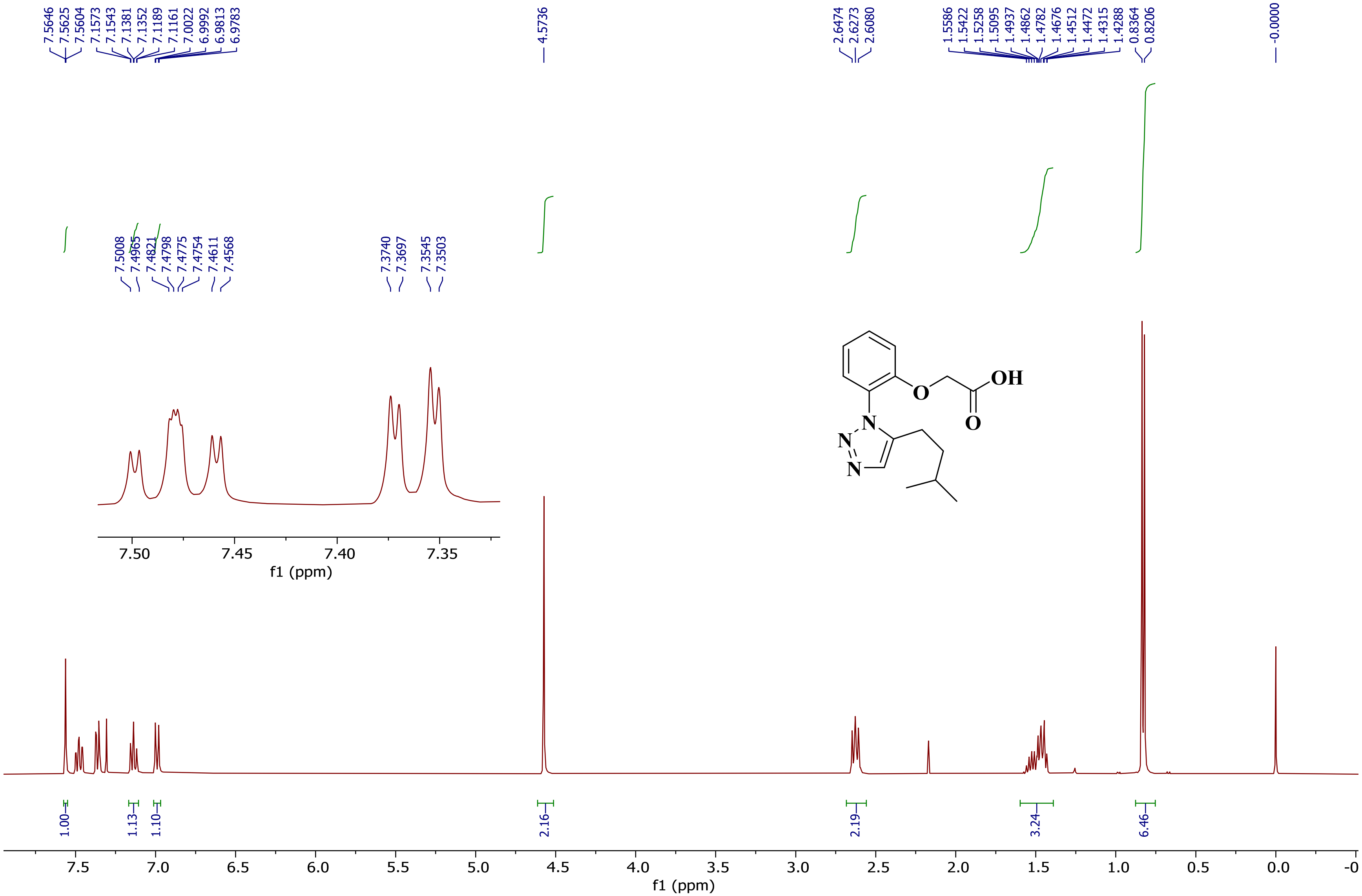

Figure S3: ^{1}H NMR of compound 6 (400 MHz, $CDCl_3$)

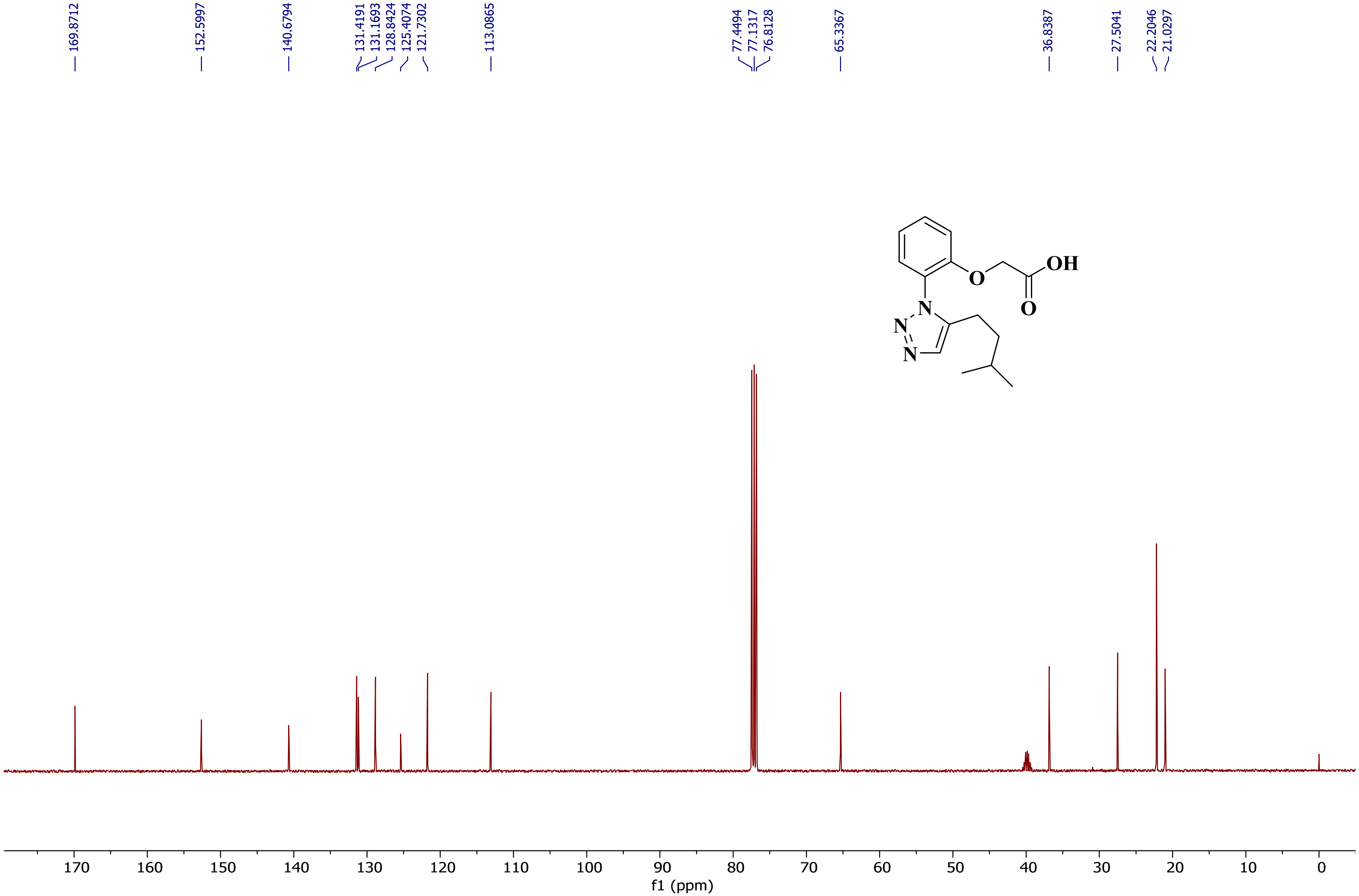

Figure S4: ^{13}C NMR of compound 6 (101 MHz, $CDCl_3$)

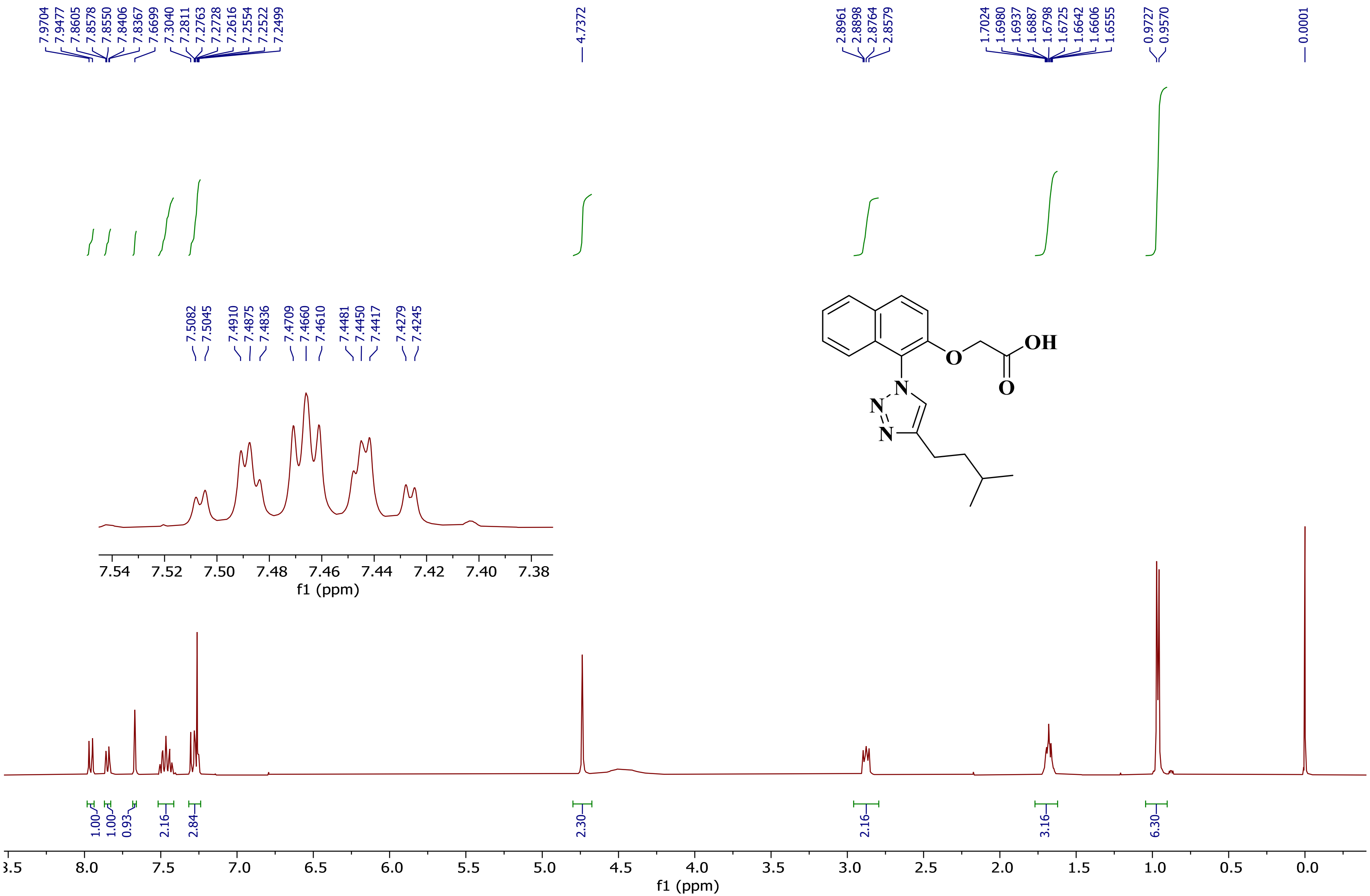

Figure S5: ¹H NMR of compound 17 (400 MHz, $CDCl_3$)

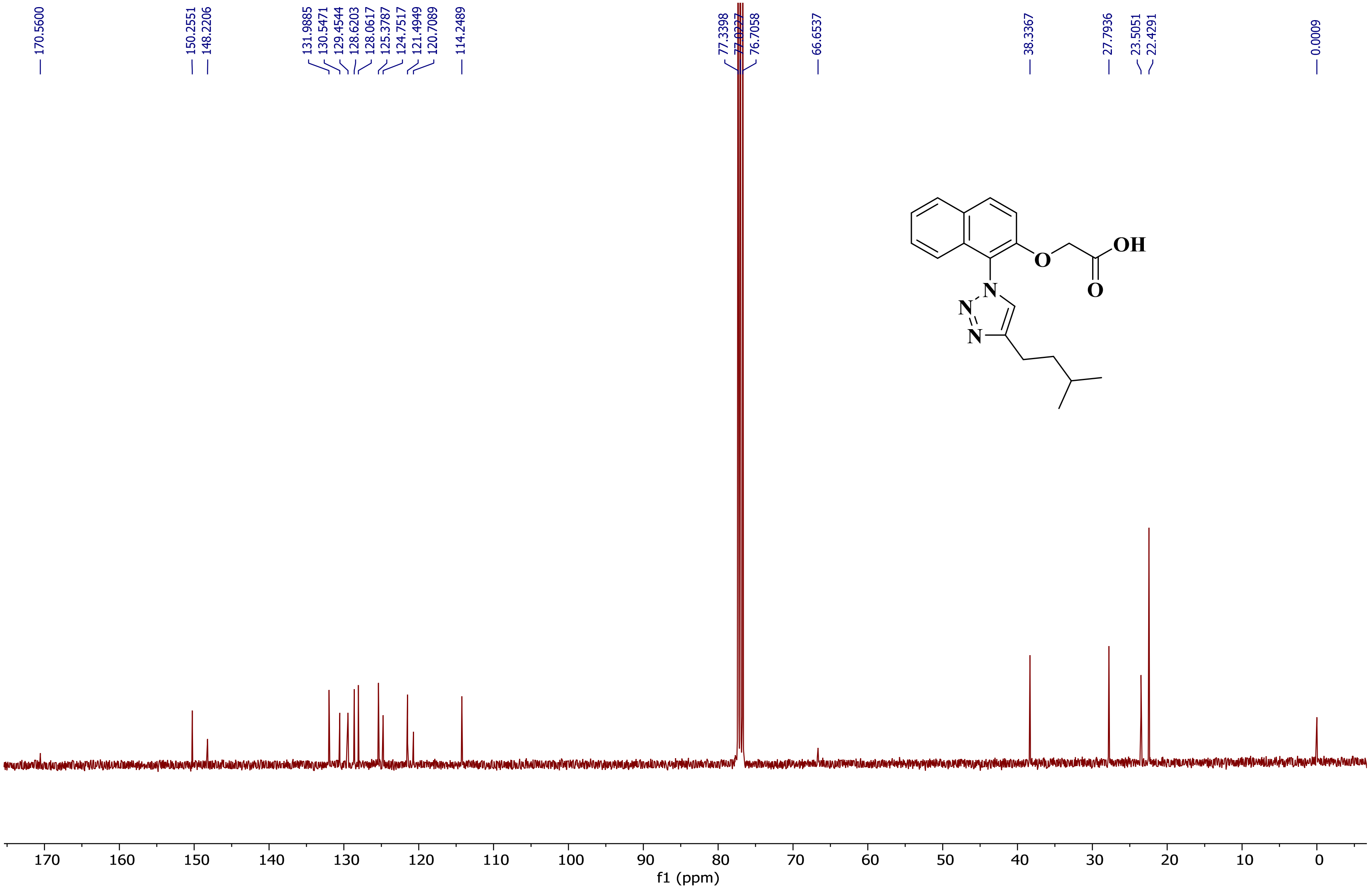

Figure S6: ^{13}C NMR of compound 17 (101 MHz, $CDCl_3$)

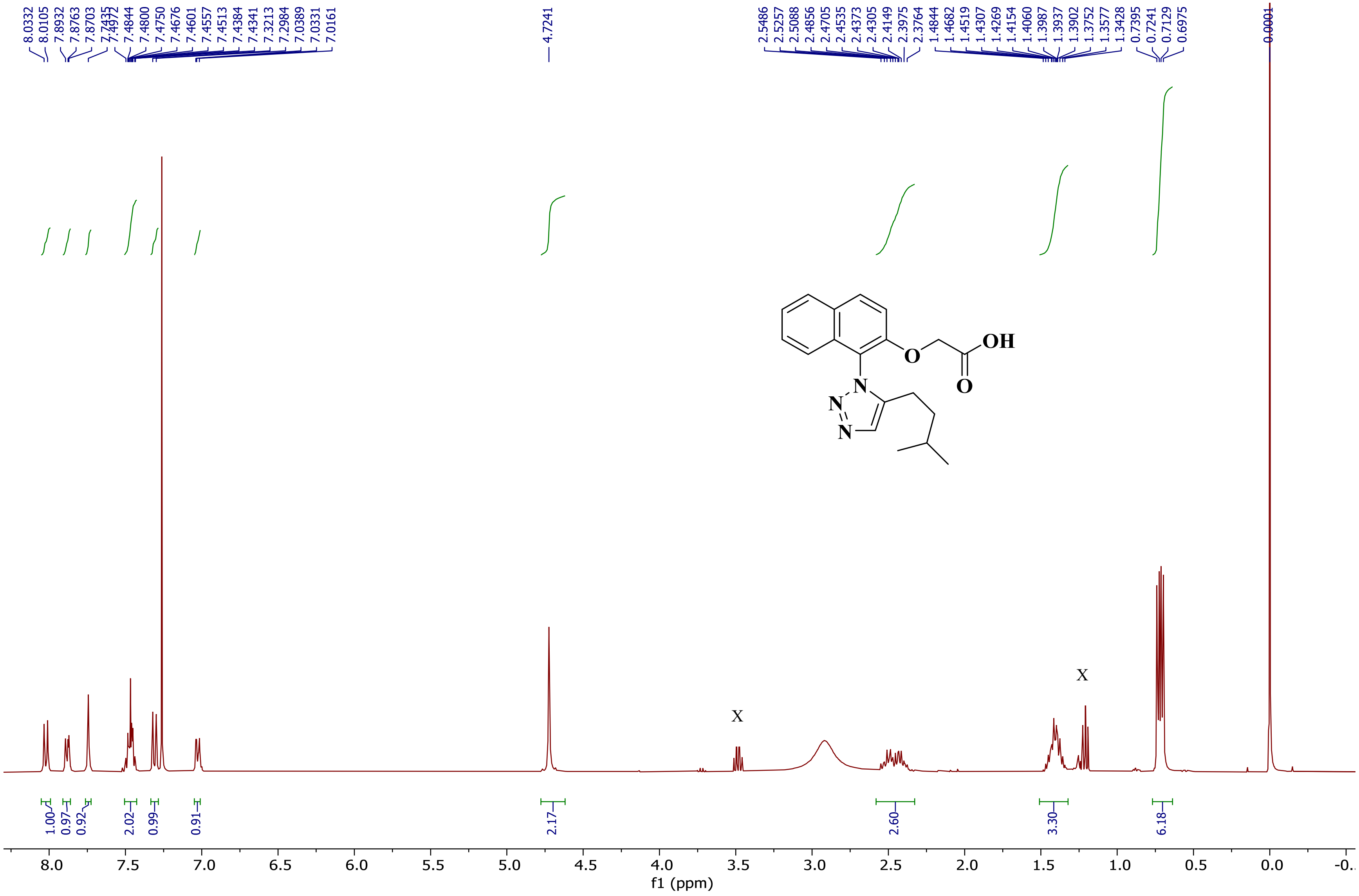

Figure S7: ^{1}H NMR of compound 18 (400 MHz, $CDCl_3$). X = diethyl ether solvent

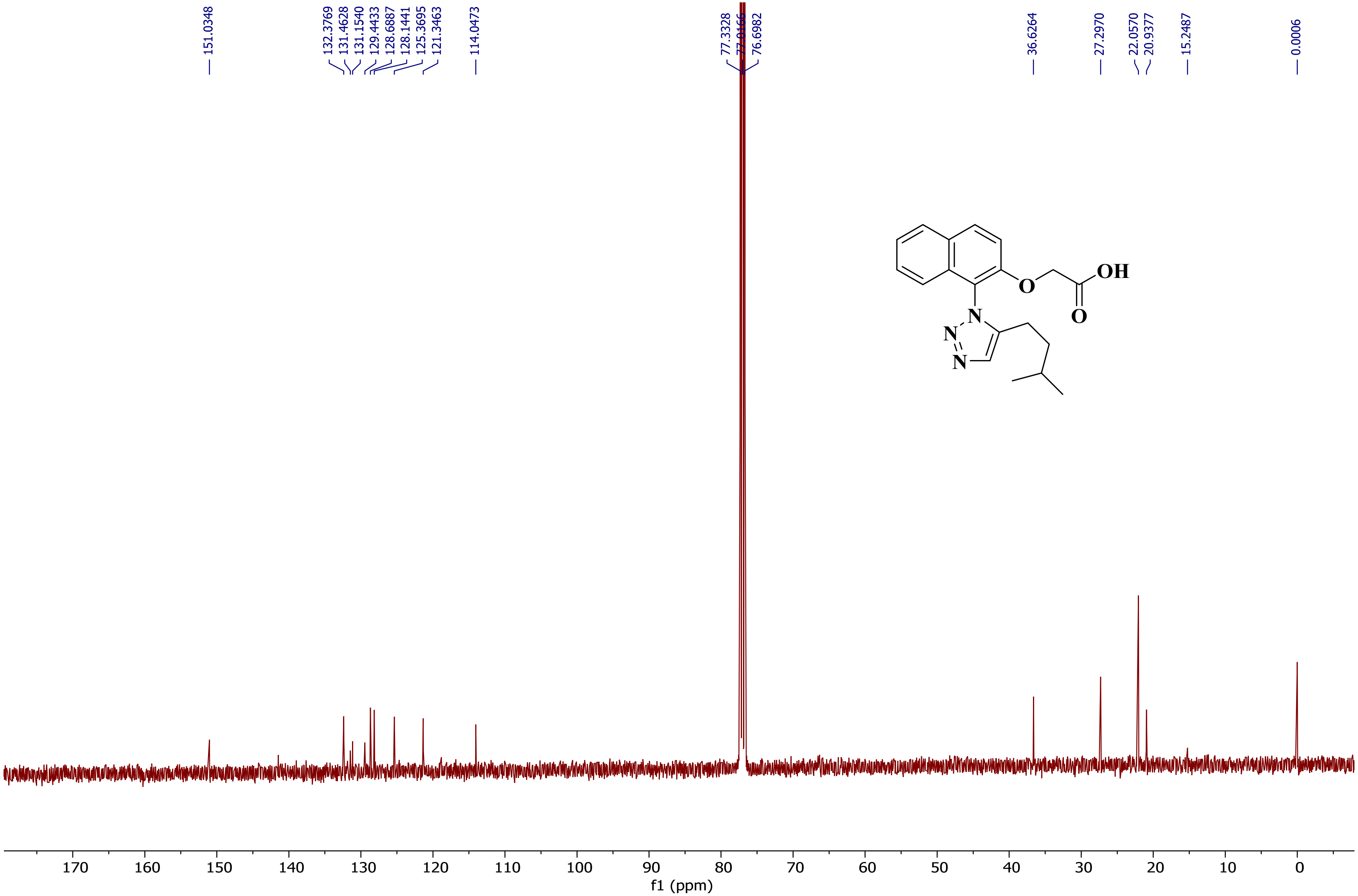

Figure S8: ^{13}C NMR of compound 18 (101 MHz, $CDCl_3$)

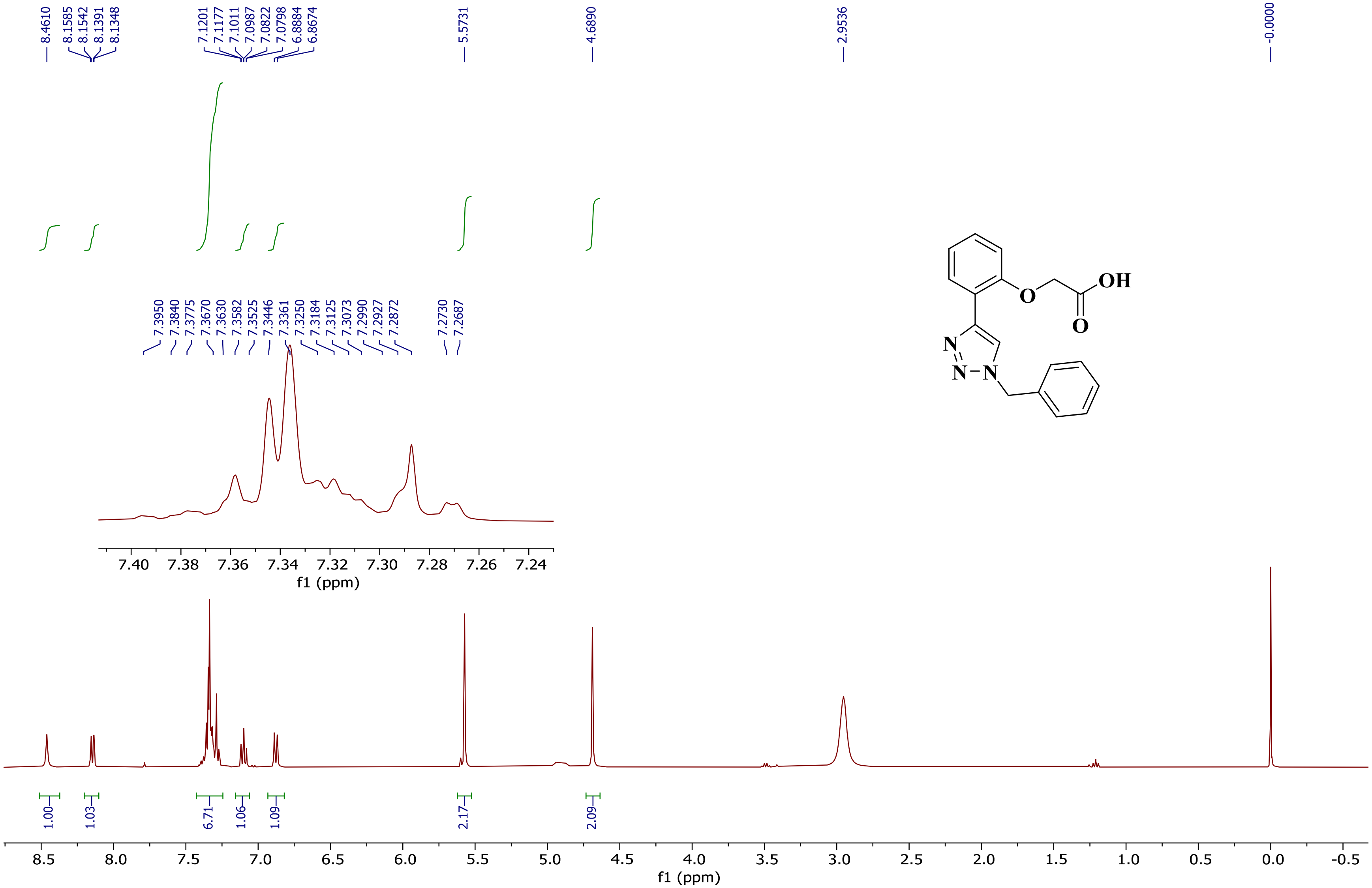

Figure S9: ^{1}H NMR of compound 27 (400 MHz, $CDCl_3$)

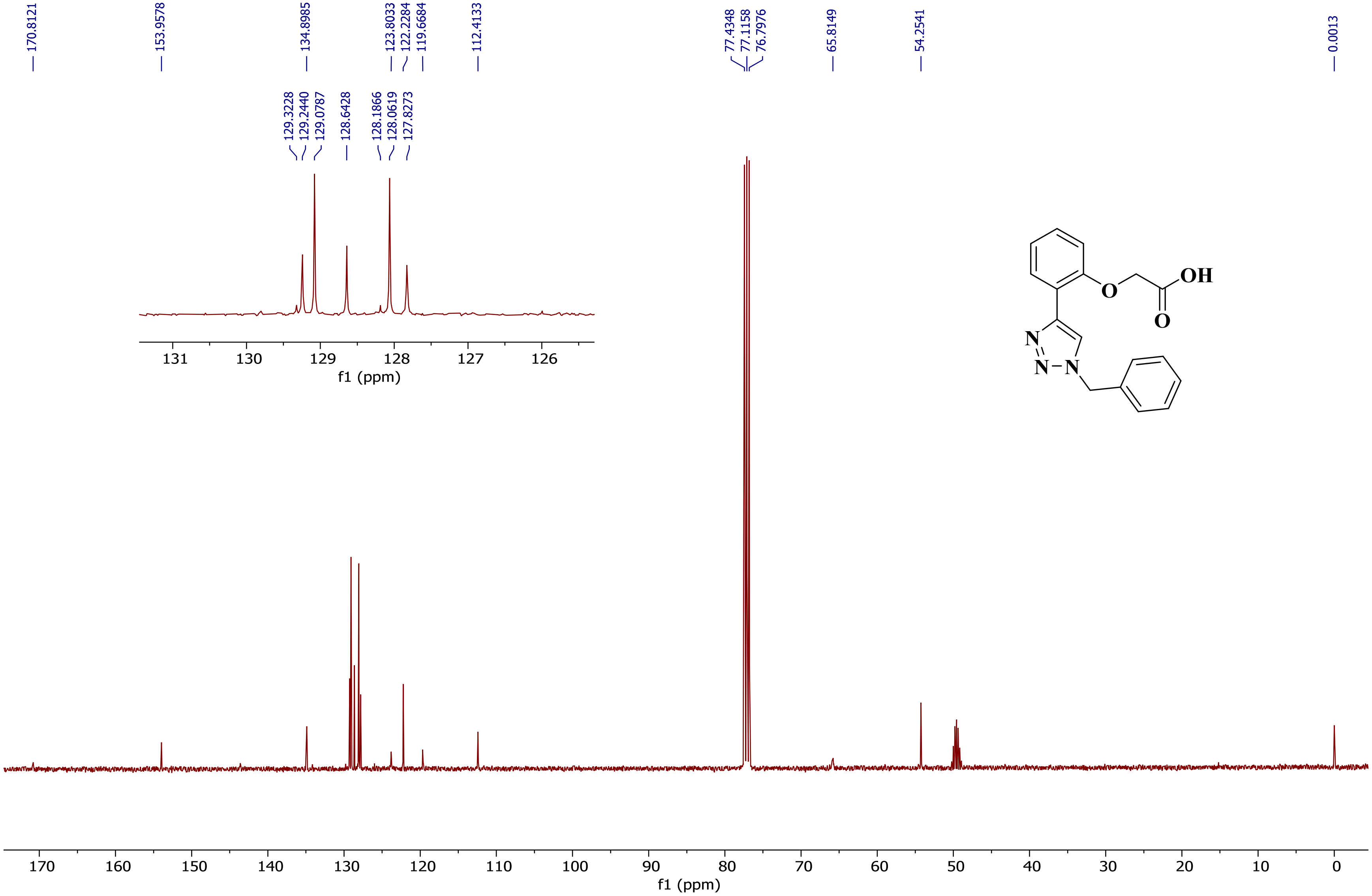

Figure S10: ^{13}C NMR of compound 27 (101 MHz, $CDCl_3$)

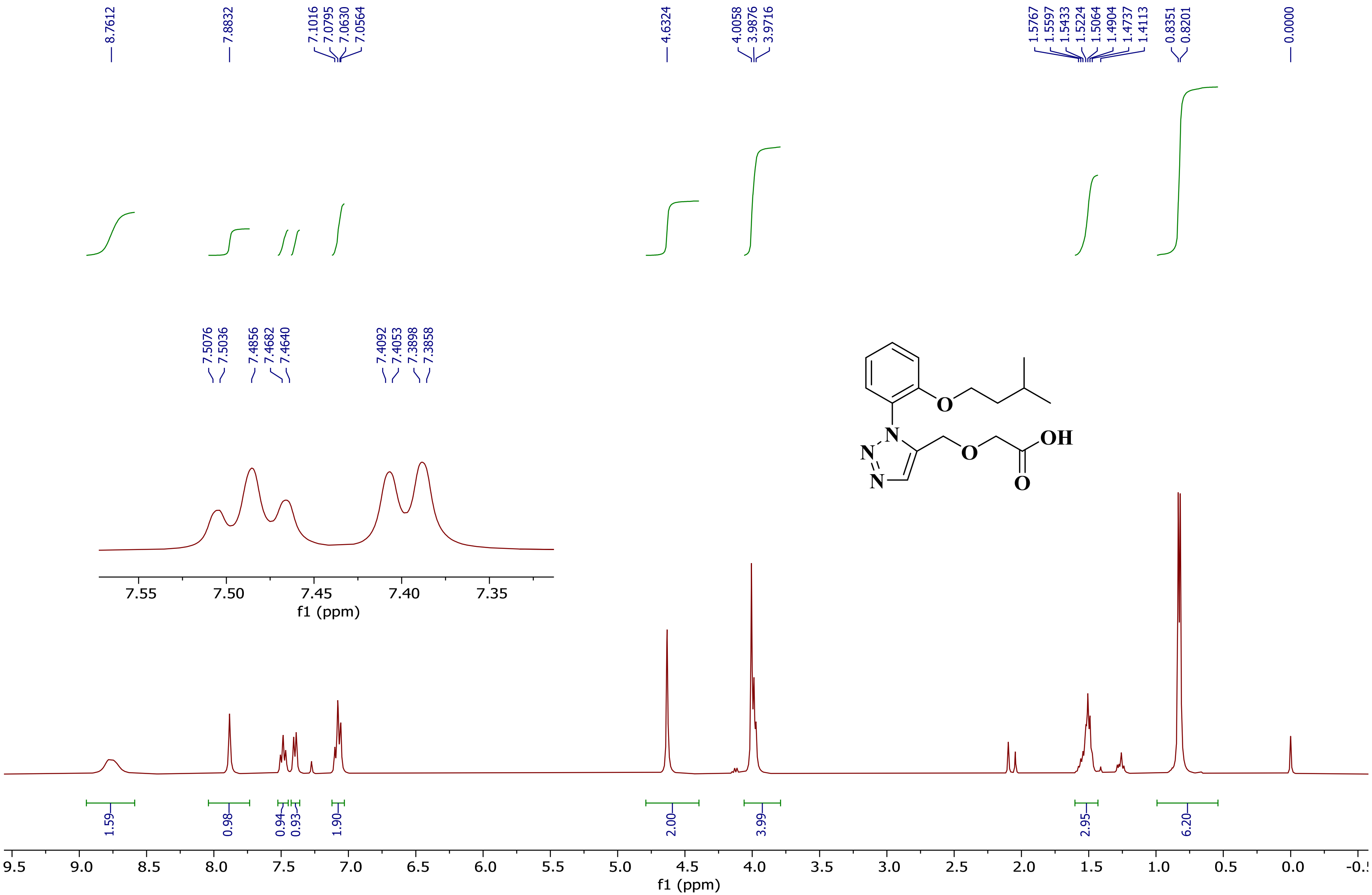

Figure S11: ^{1}H NMR of compound 28 (400 MHz, $CDCl_3$)

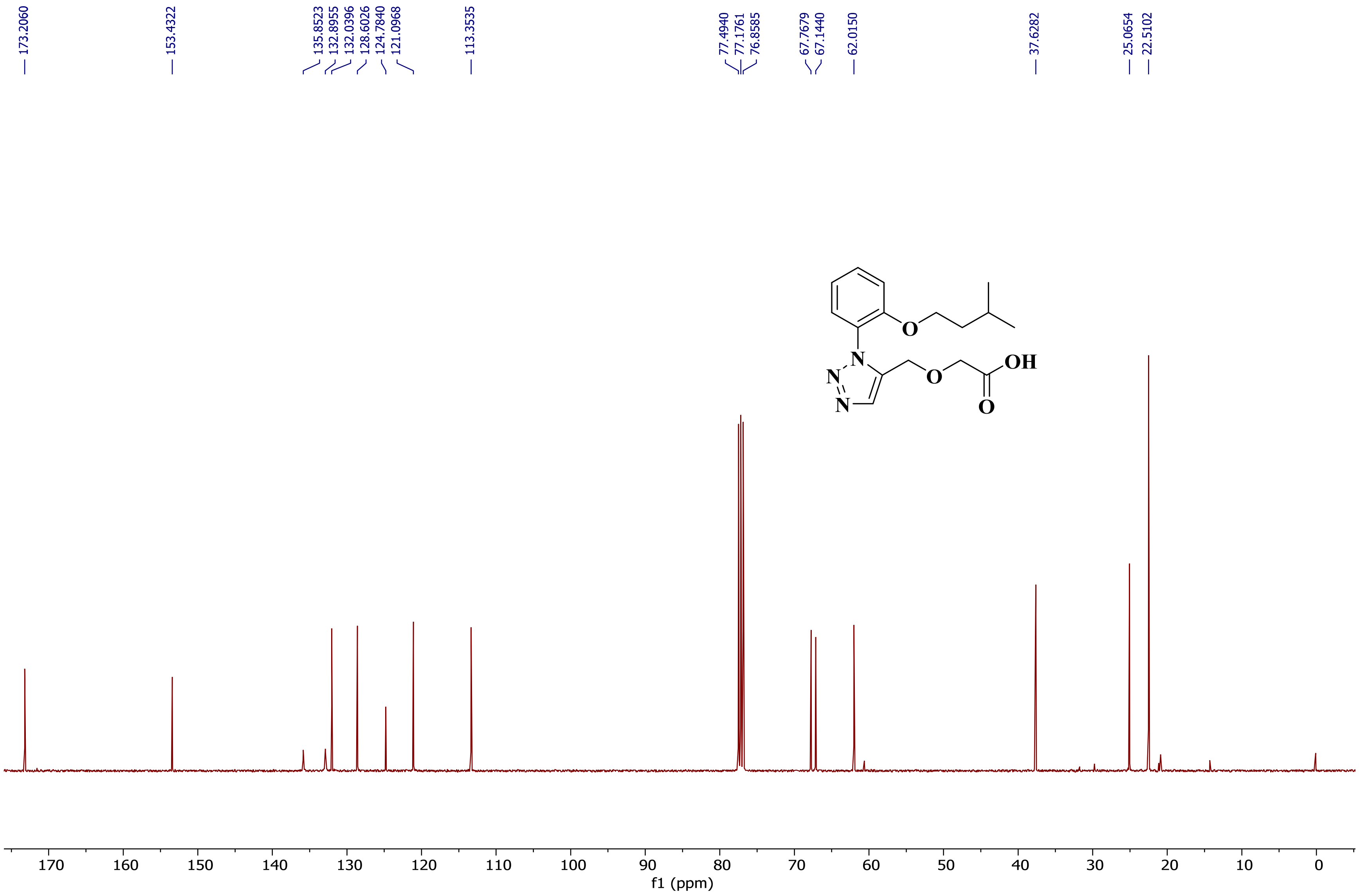

Figure S12: ^{13}C NMR of compound 28 (101 MHz, $CDCl_3$)

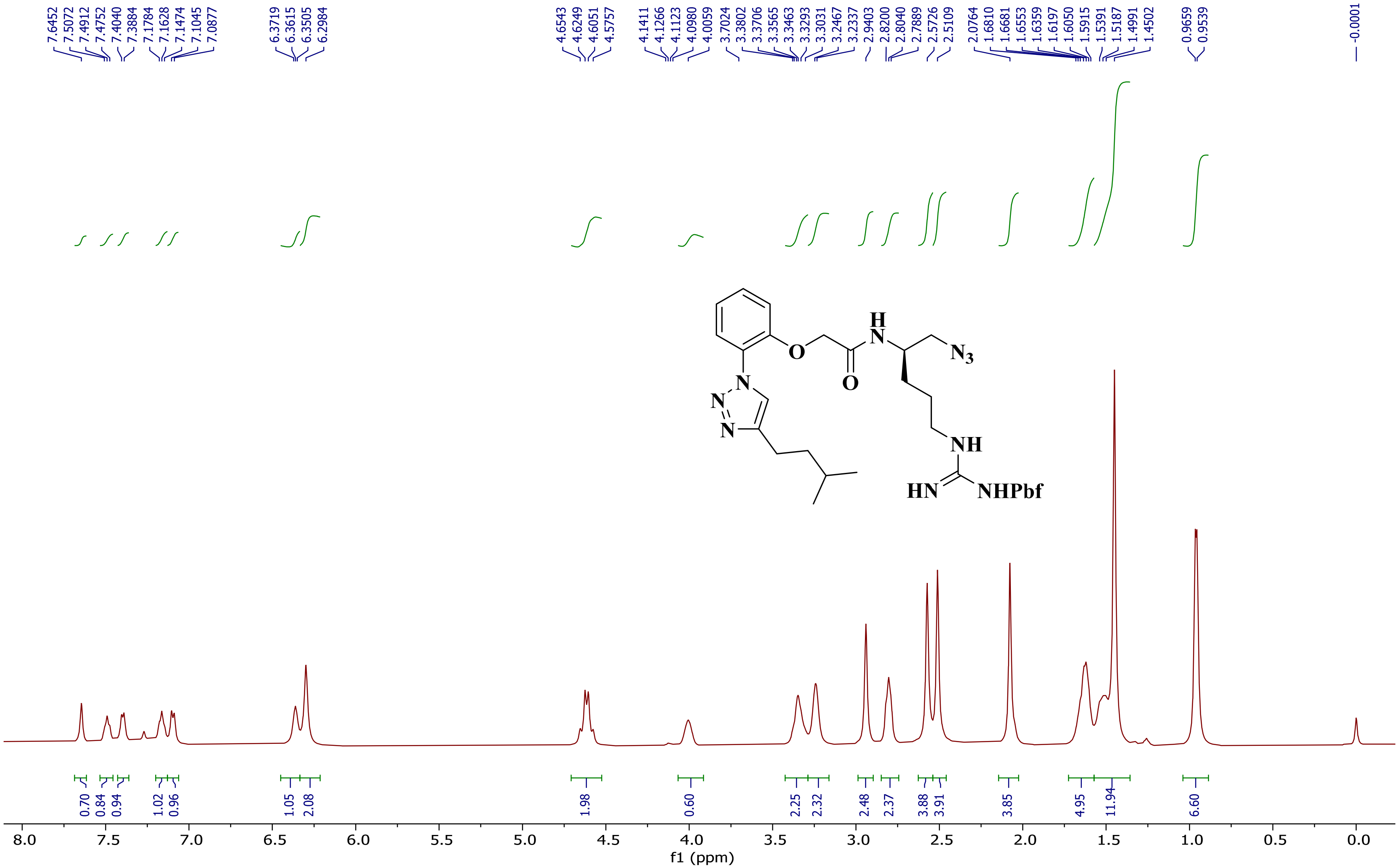

Figure S13: ^{1}H NMR of compound 8 (400 MHz, $CDCl_3$)

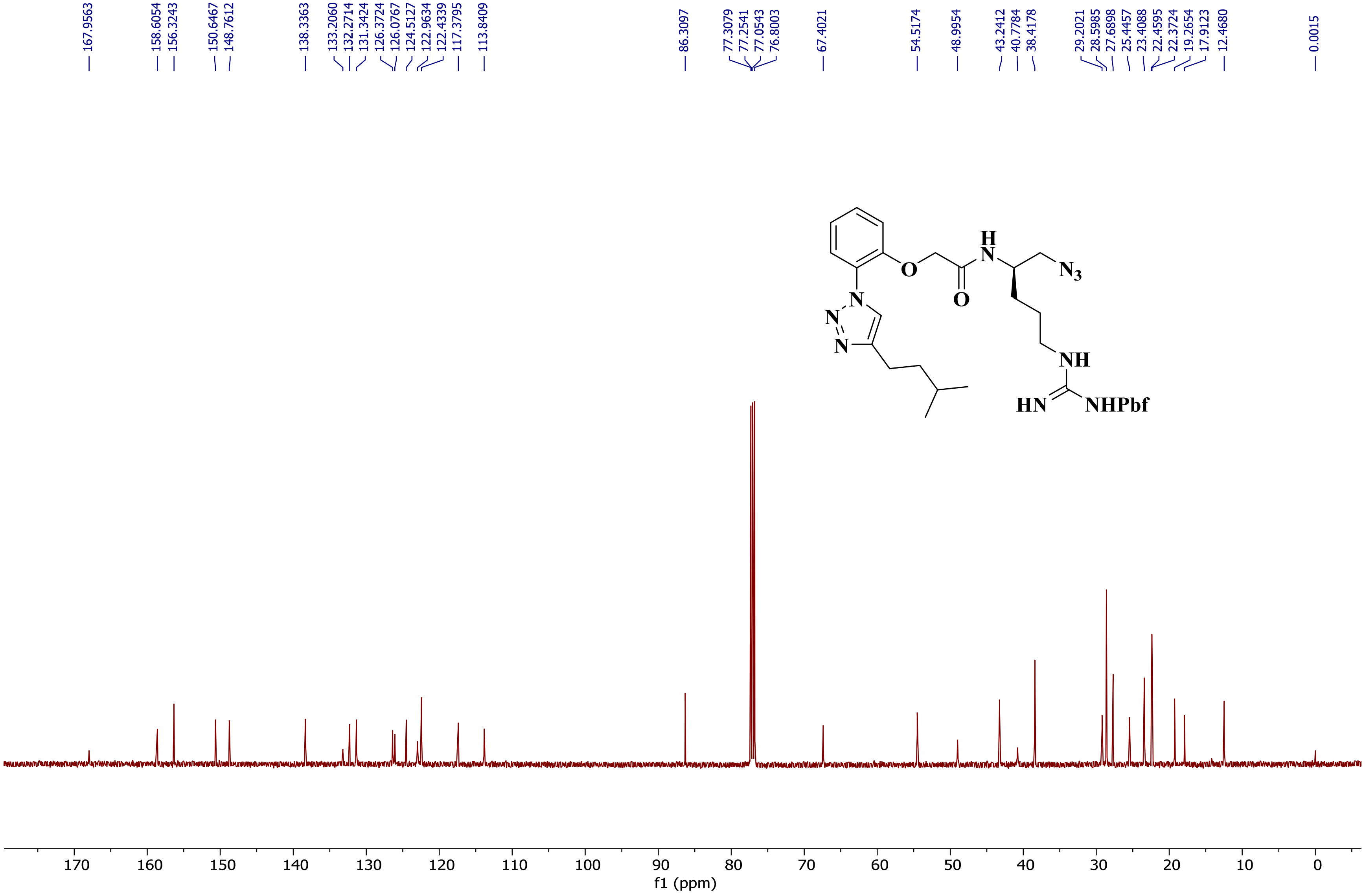

Figure S14: ^{13}C NMR of compound 8 (101 MHz, $CDCl_3$)

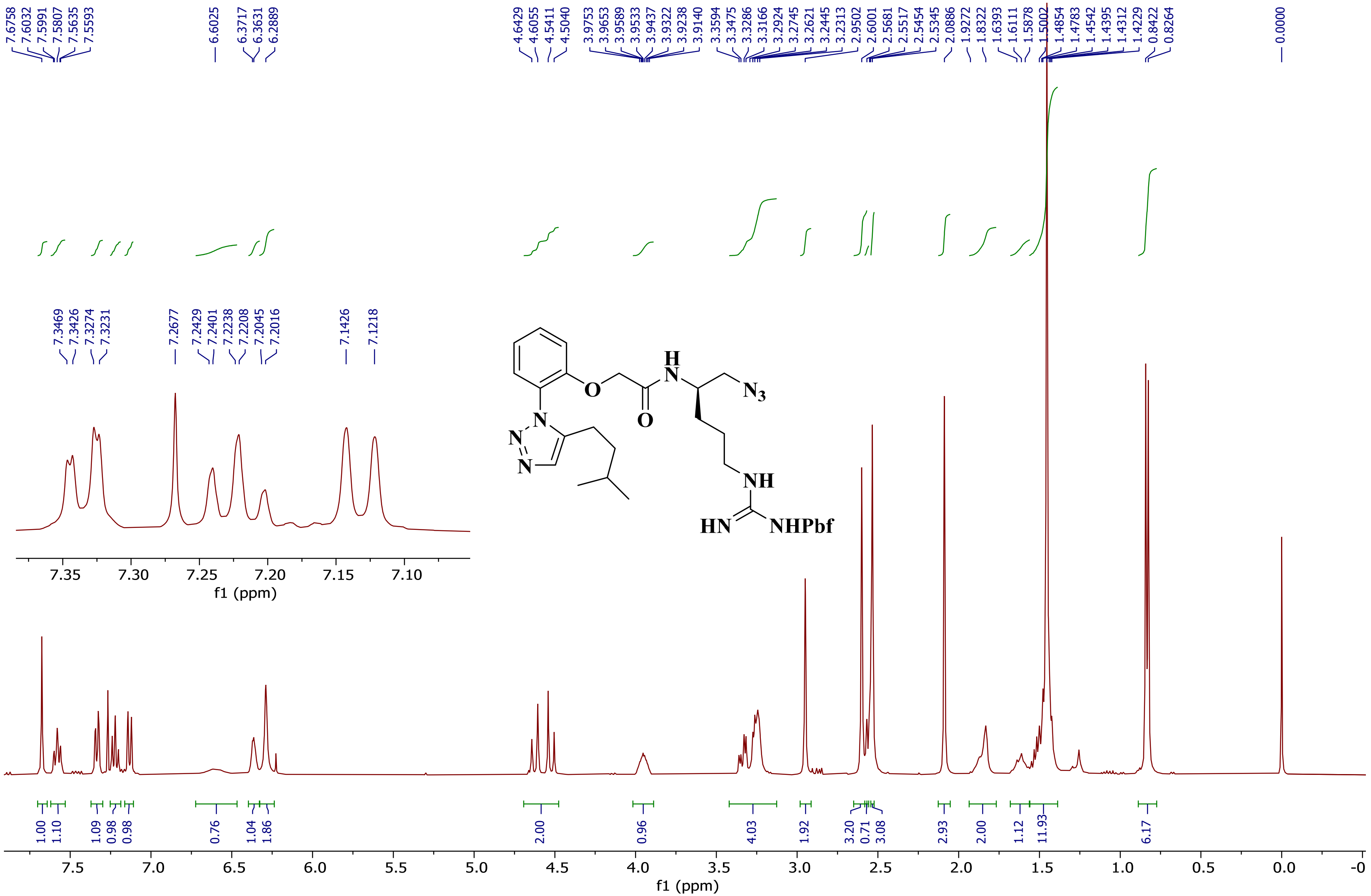

Figure S15: ^{1}H NMR of compound 9 (400 MHz, $CDCl_3$)

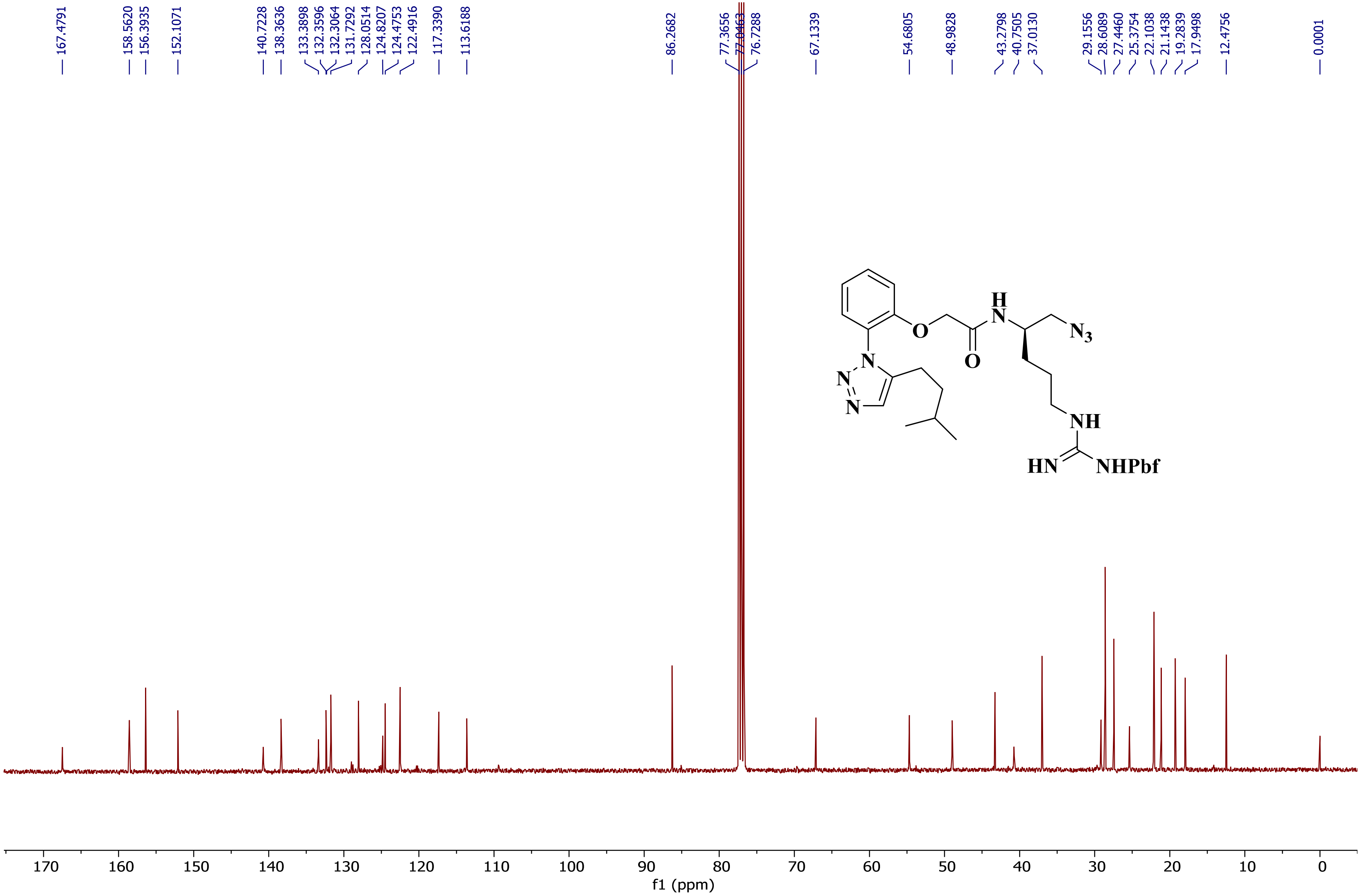

Figure S16: ^{13}C NMR of compound 9 (101 MHz, $CDCl_3$)

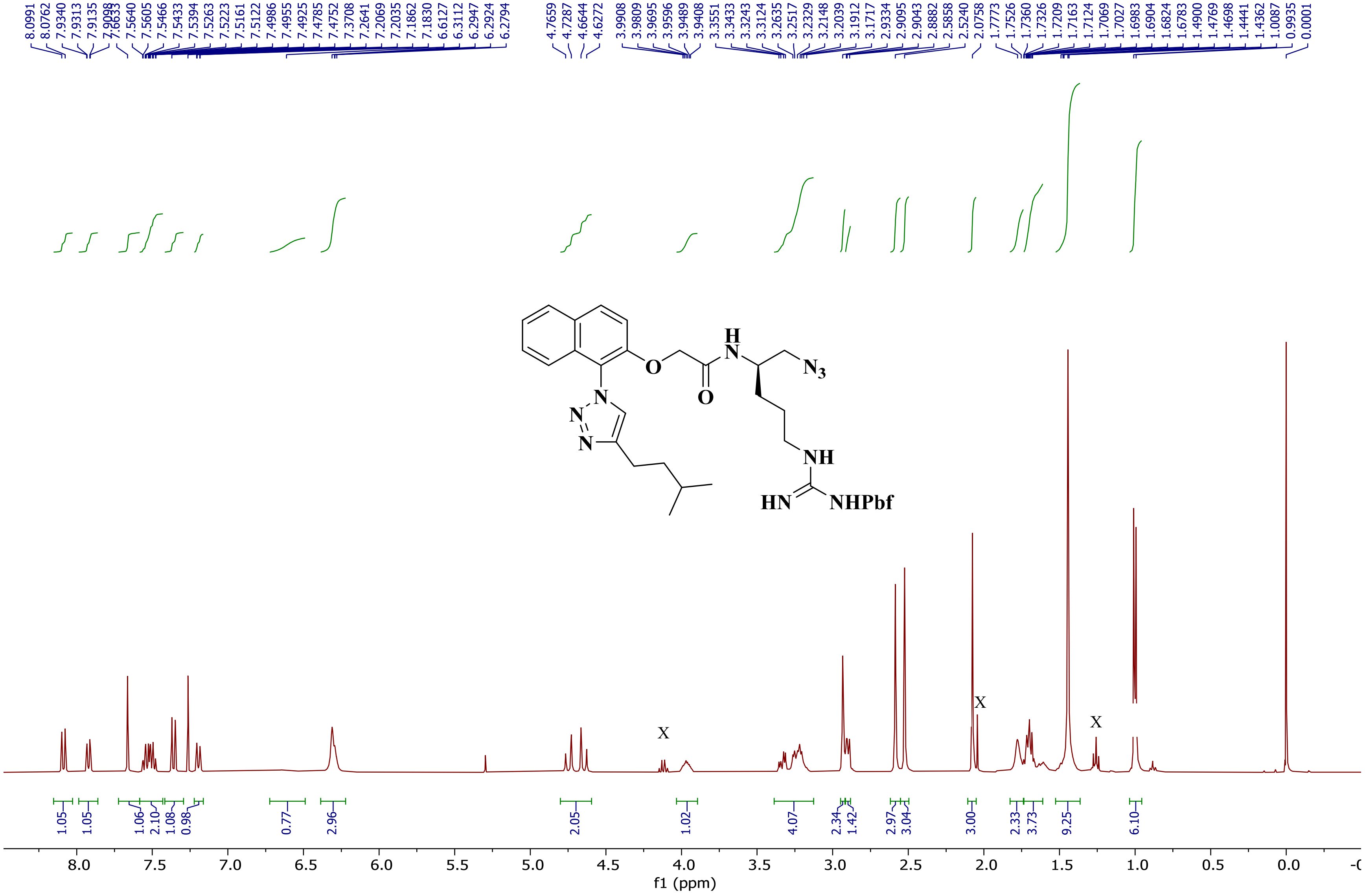

Figure S17: ^{1}H NMR of compound 19 (400 MHz, $CDCl_3$). X = ethyl acetate solvent

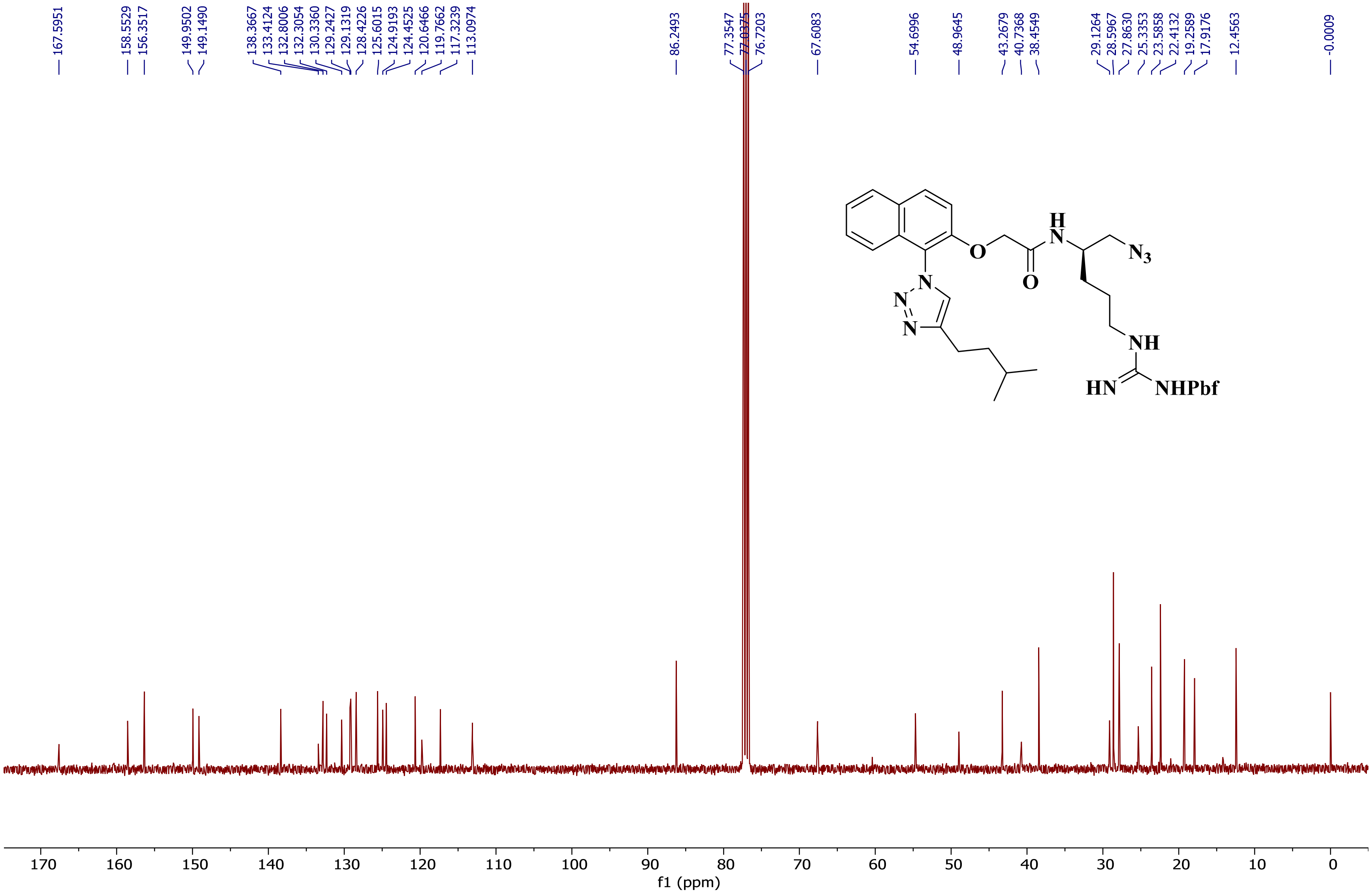

Figure S18: ^{13}C NMR of compound 19 (101 MHz, CDCl$_3$)

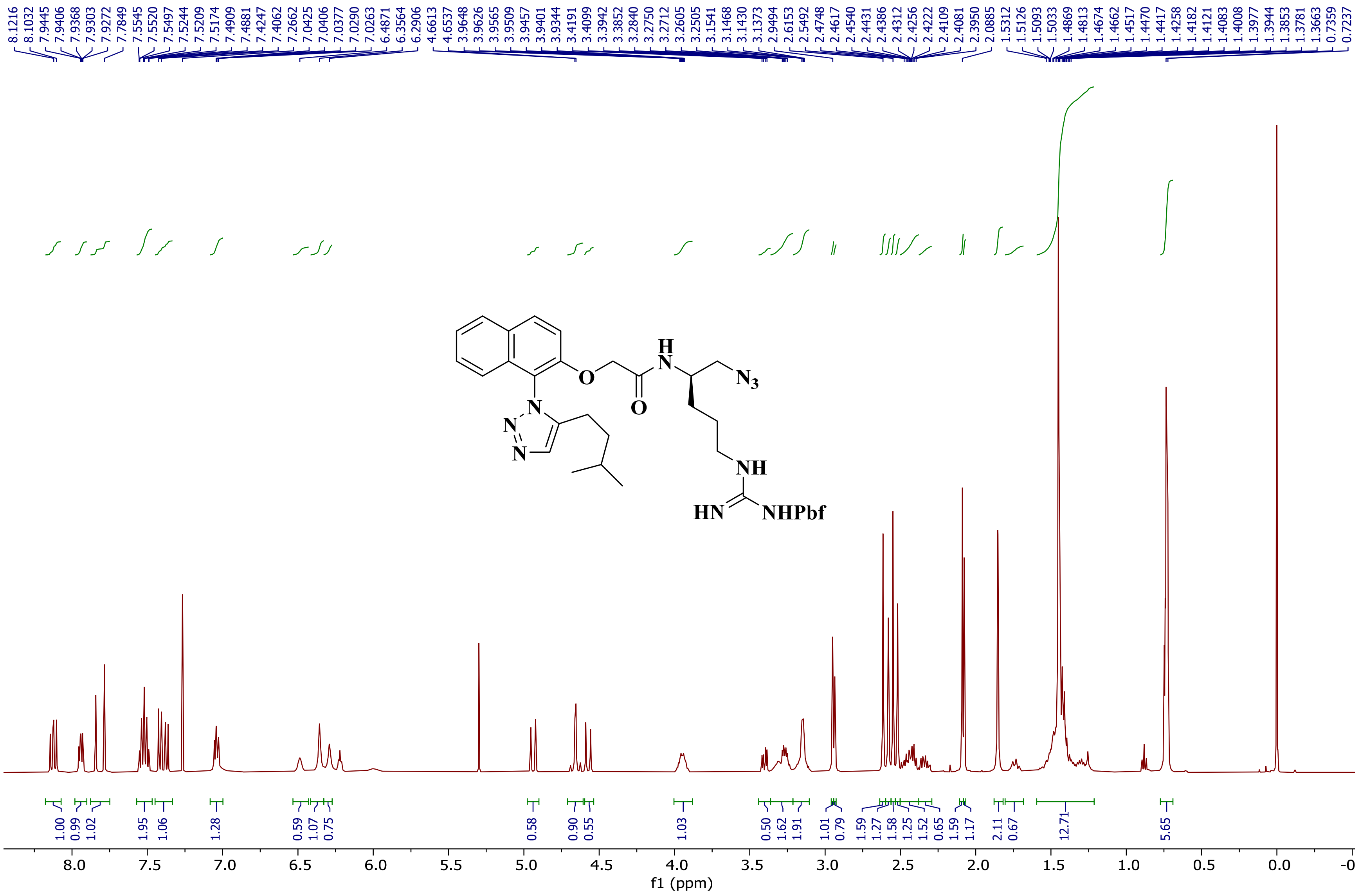

Figure S19: ^{1}H NMR of compound 20 (400 MHz, $CDCl_3$). Rotamers apparent in region 2.0 – 3.0 ppm.

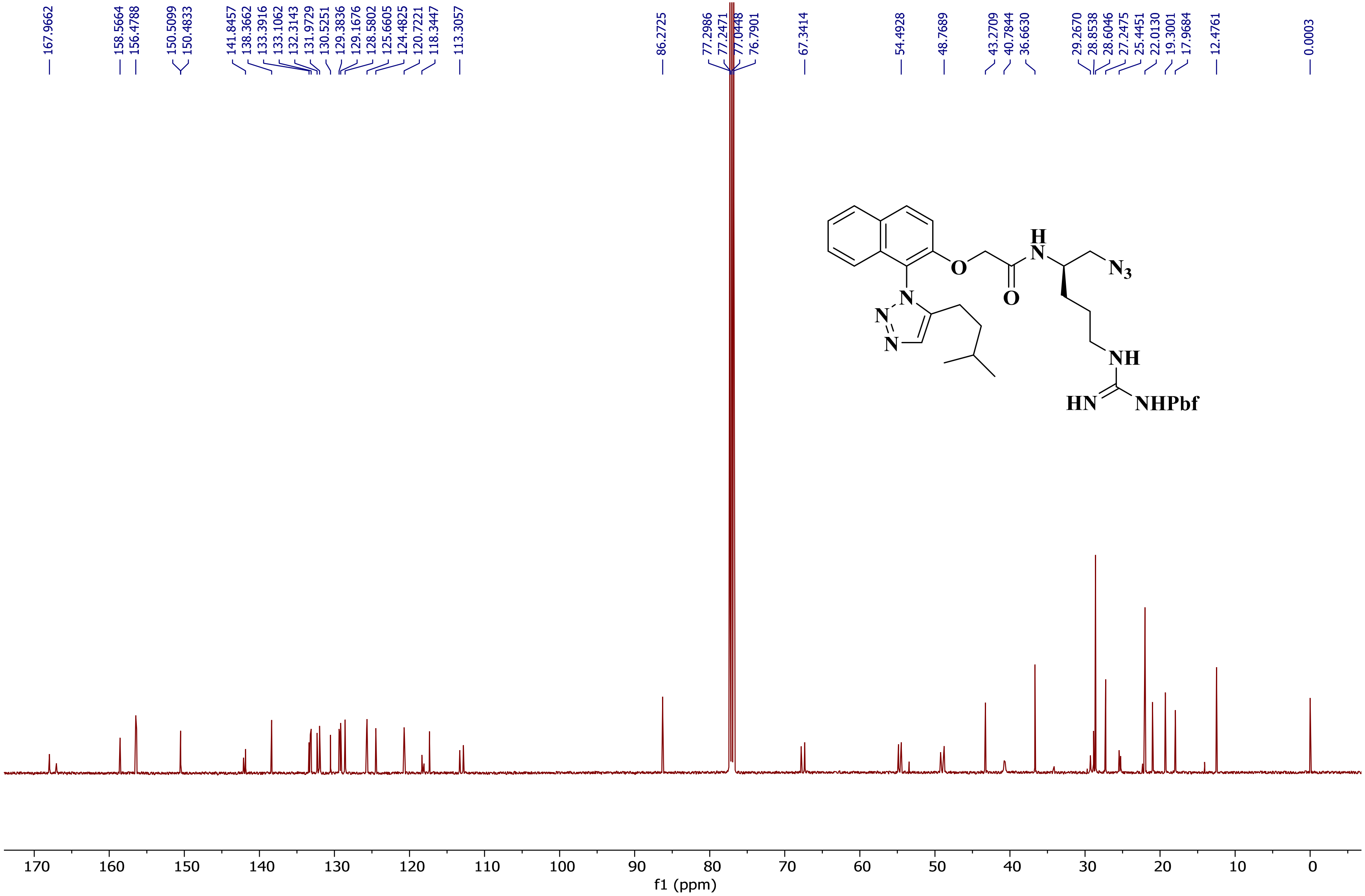

Figure S20: ^{13}C NMR of compound 20 (101 MHz, $CDCl_3$). Rotamers apparent in spectrum as evidenced by doubling of resonances.

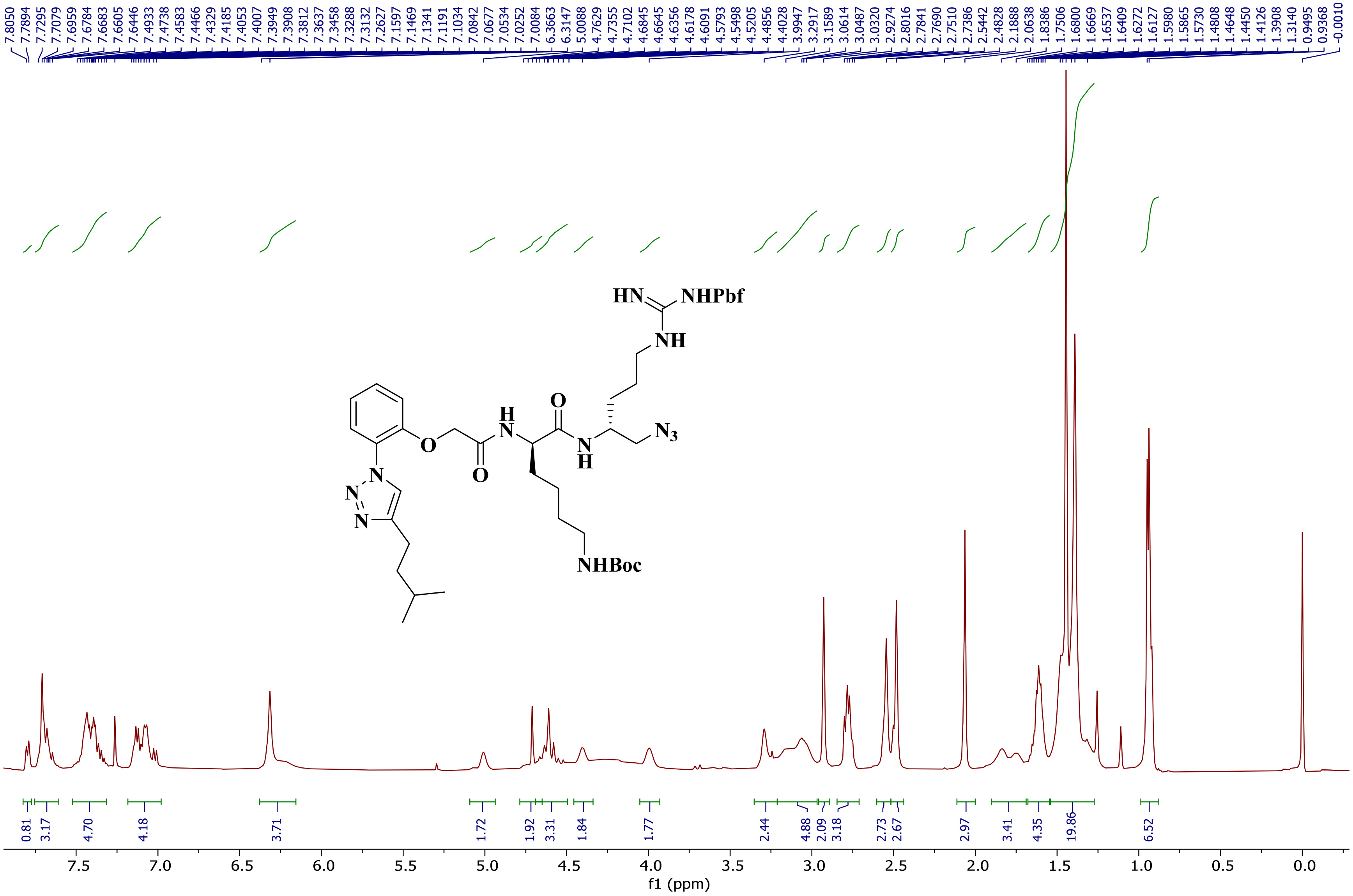

Figure S21: ^{1}H NMR of compound 30 (400 MHz, $CDCl_3$). Rotamers apparent in spectrum as evidenced by resonance broadening.

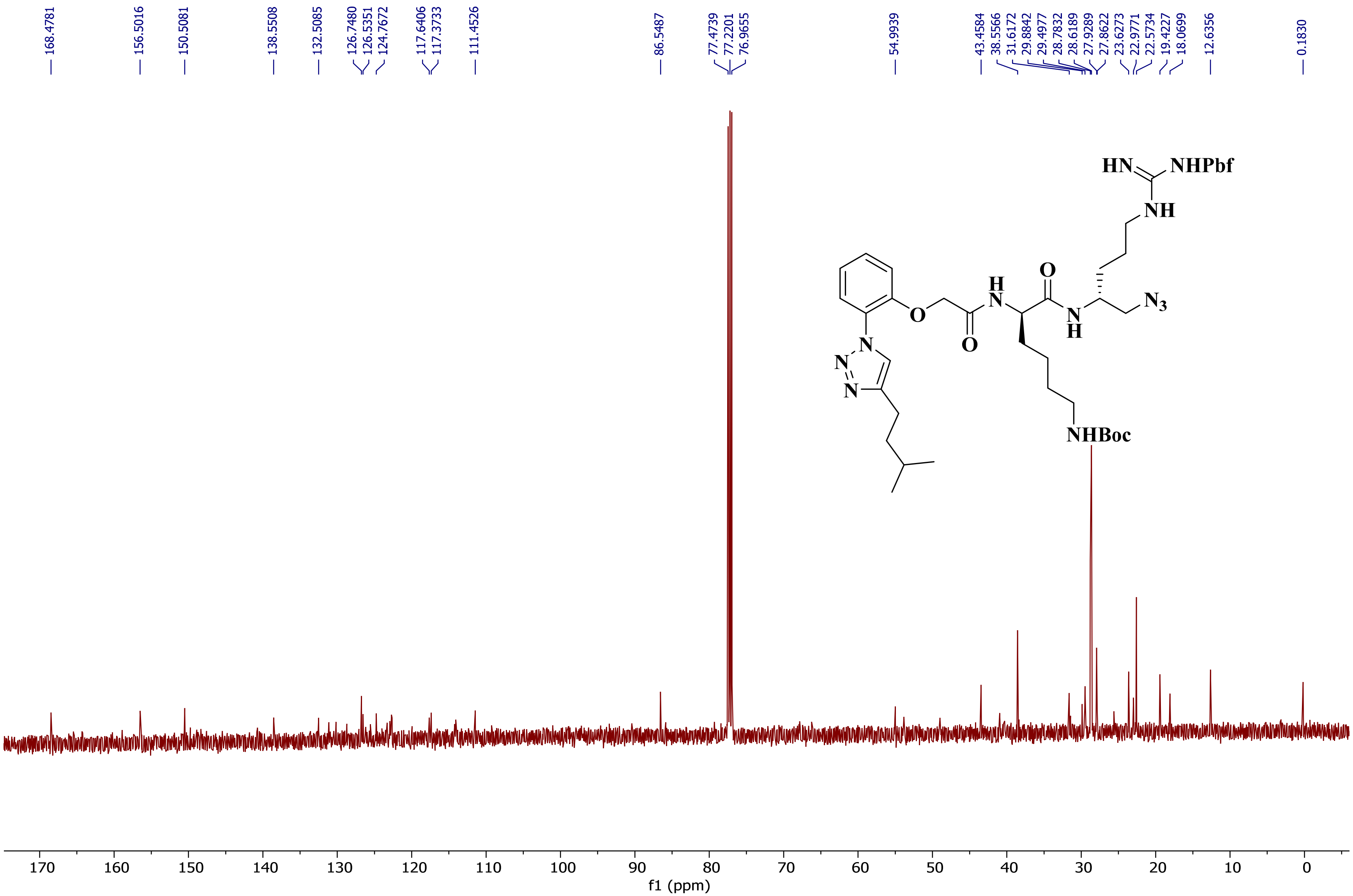

Figure S22: ^{13}C NMR of compound 30 (101 MHz, CDCl$_3$). Rotamers apparent in spectrum as evidenced by doubling of resonances.

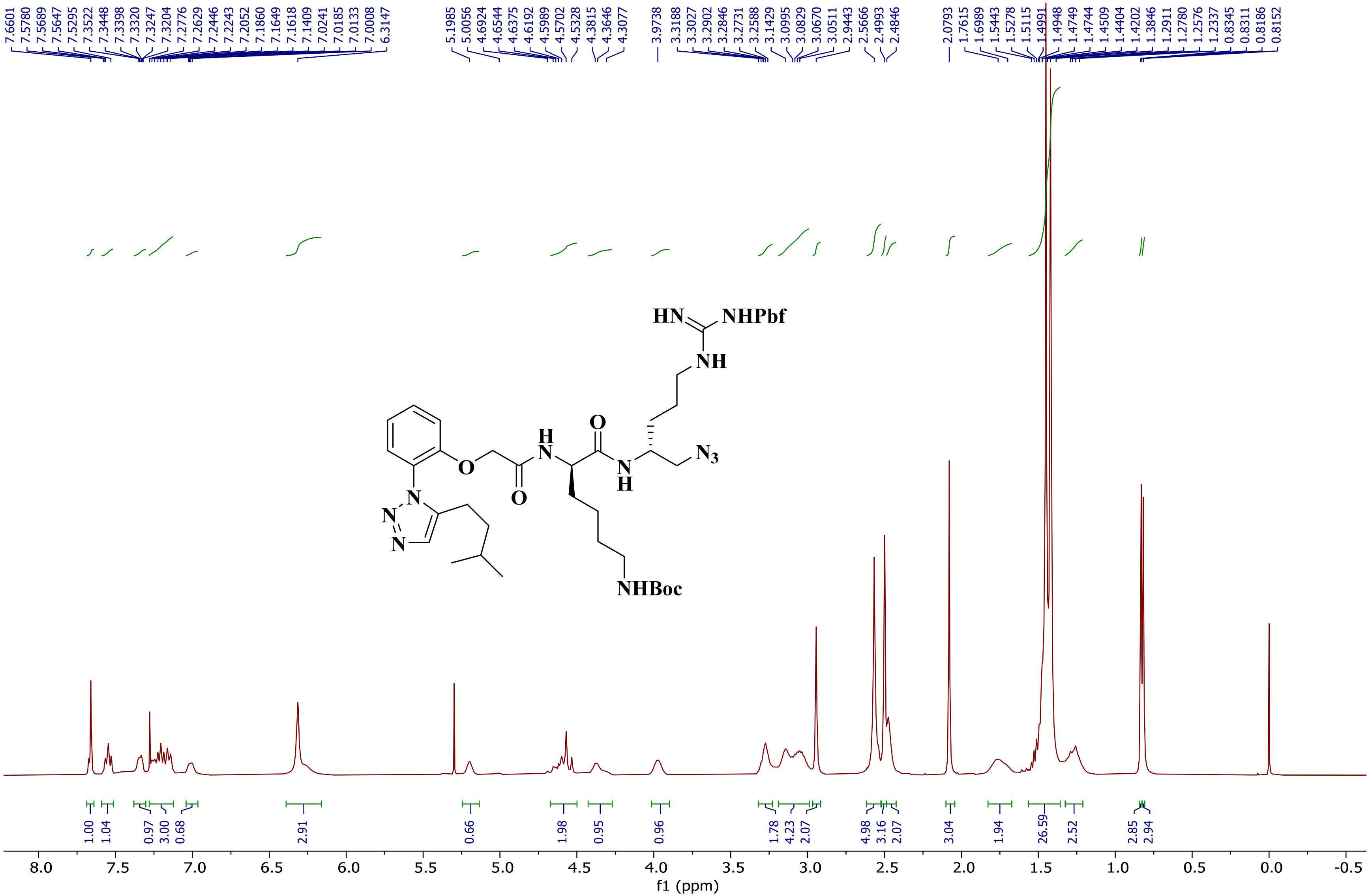

Figure S23: ^{1}H NMR of compound 31 (400 MHz, $CDCl_3$). Rotamers apparent in spectrum as evidenced by resonance broadening.

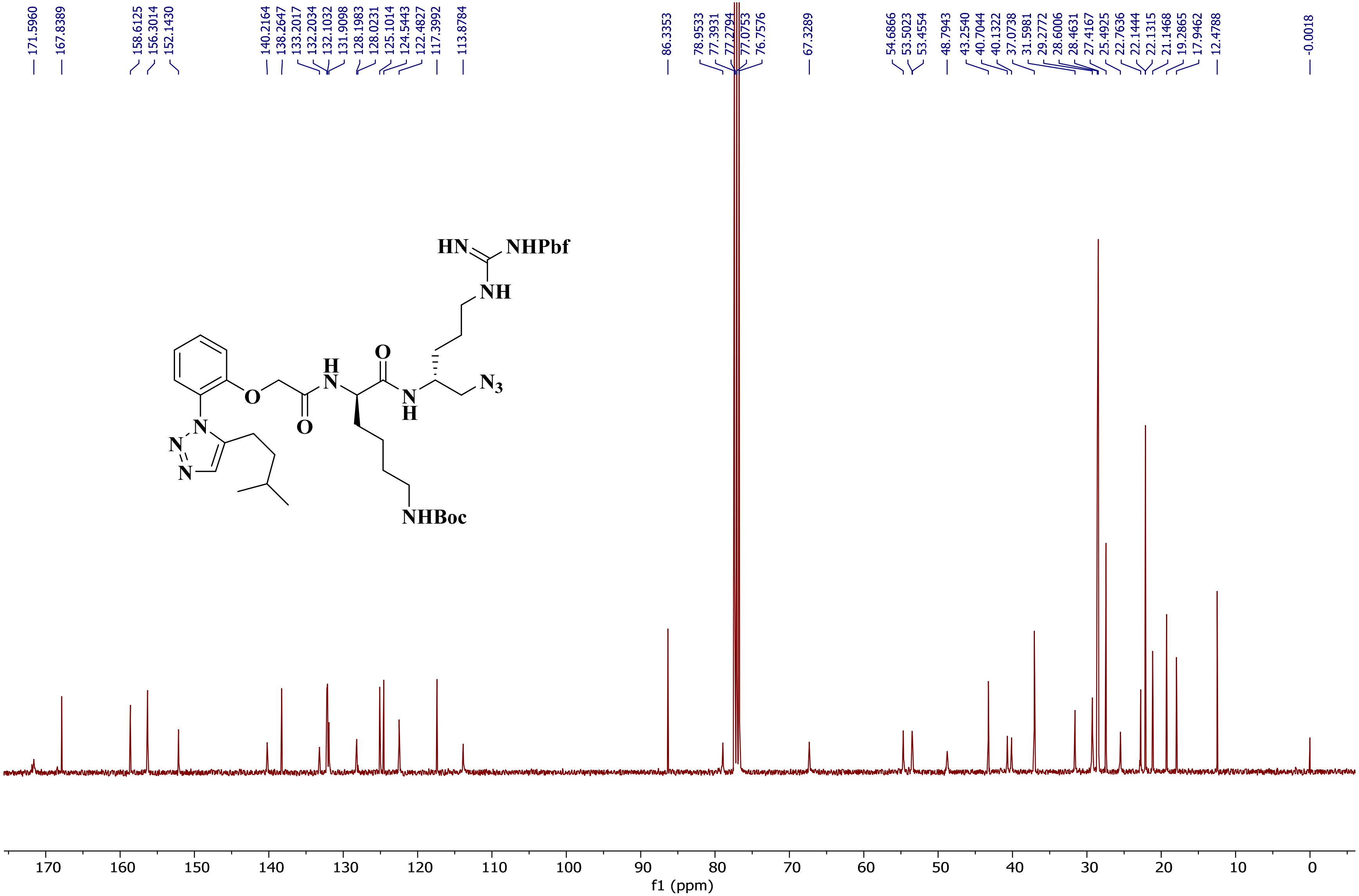

Figure S24: ^{13}C NMR of compound 31 (101 MHz, $CDCl_3$). Rotamers apparent in spectrum as evidenced by doubling of resonances.

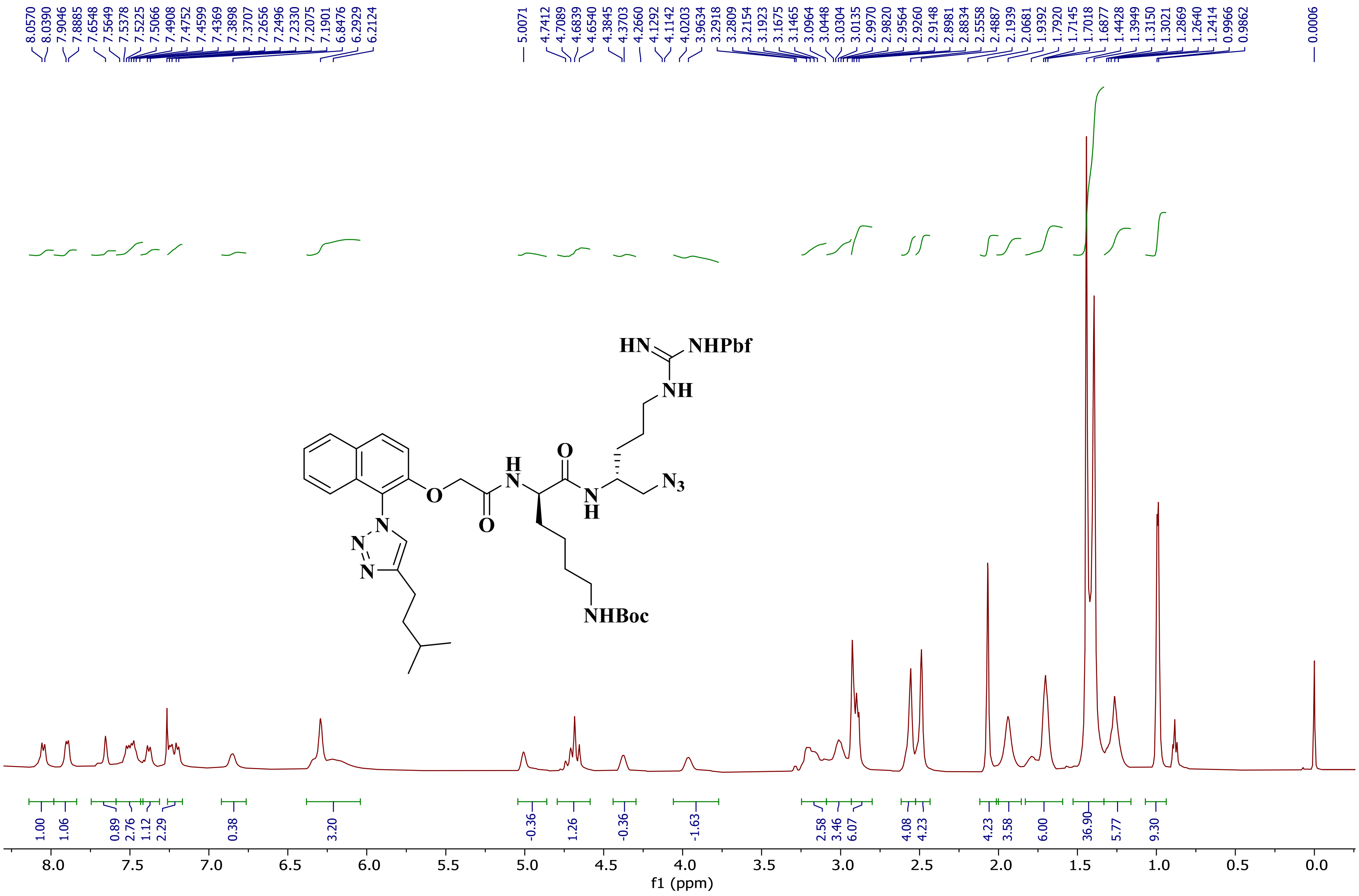

Figure S25: ^{1}H NMR of compound 32 (400 MHz, $CDCl_3$). Rotamers apparent in spectrum as evidenced by resonance broadening.

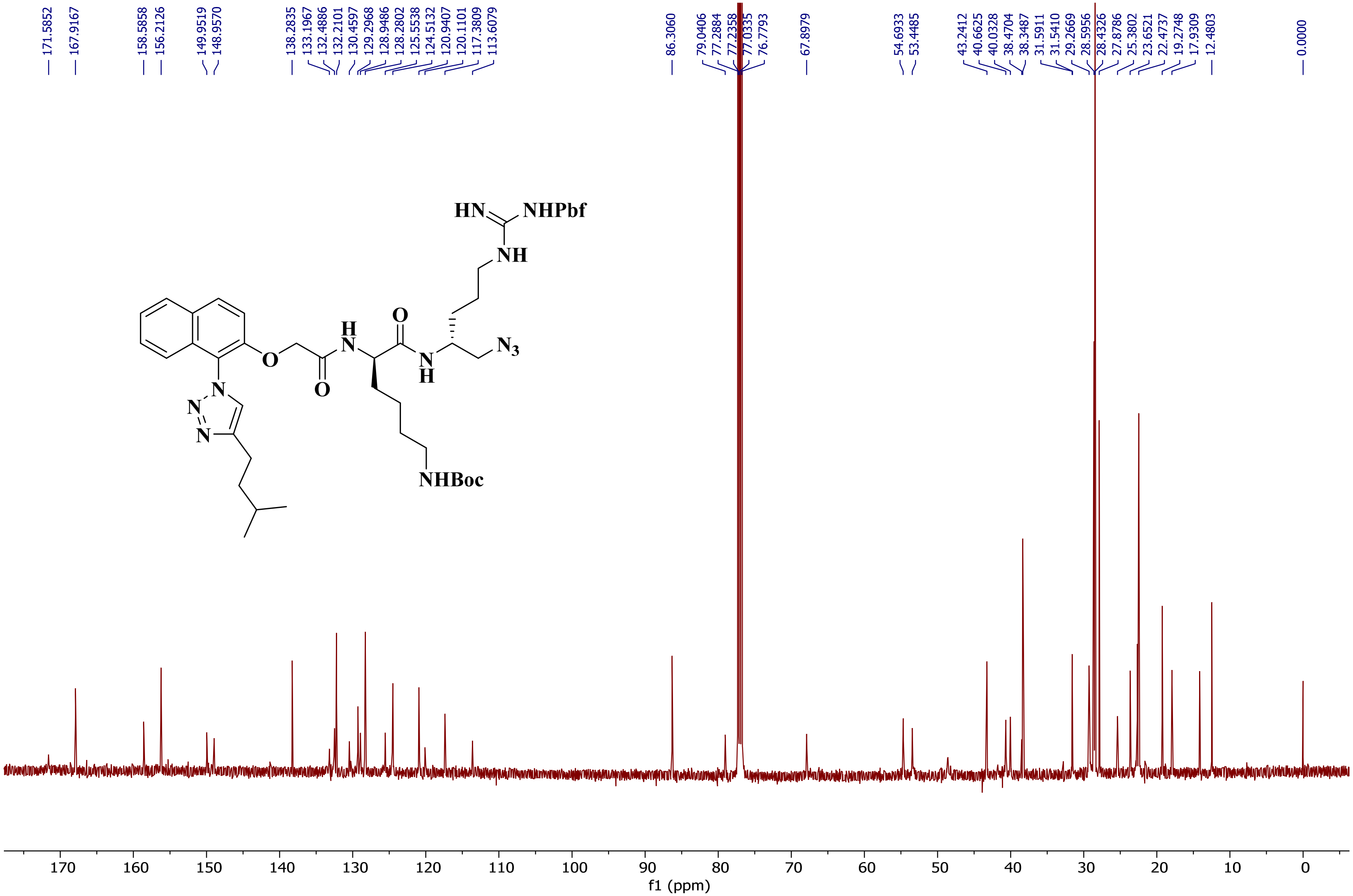

Figure S26: ^{13}C NMR of compound 32 (101 MHz, CDCl$_3$). Rotamers apparent in spectrum as evidenced by doubling of resonances.

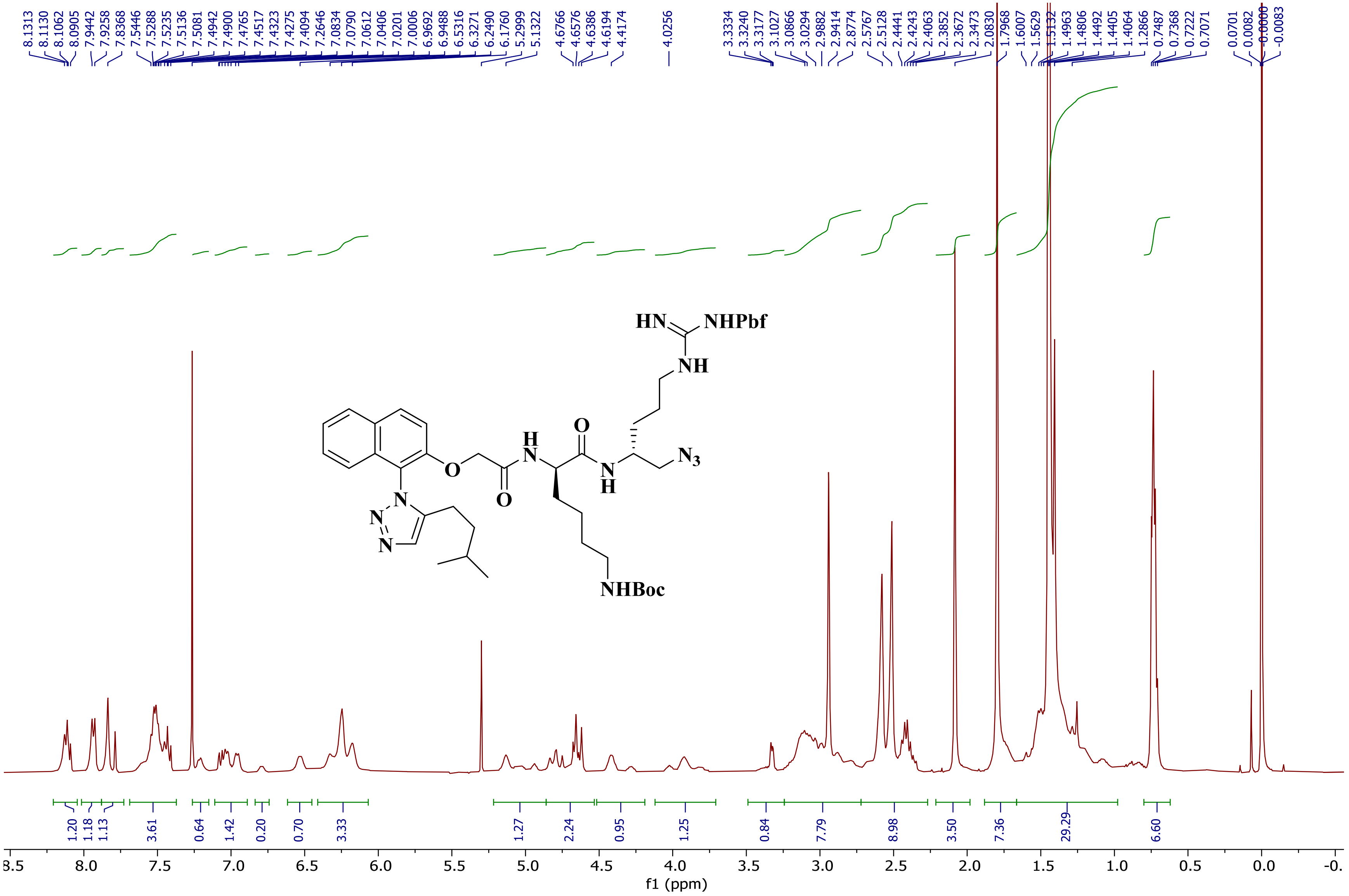

Figure S27: ^{1}H NMR of compound 33 (400 MHz, $CDCl_3$). Rotamers apparent in spectrum as evidenced by resonance broadening.

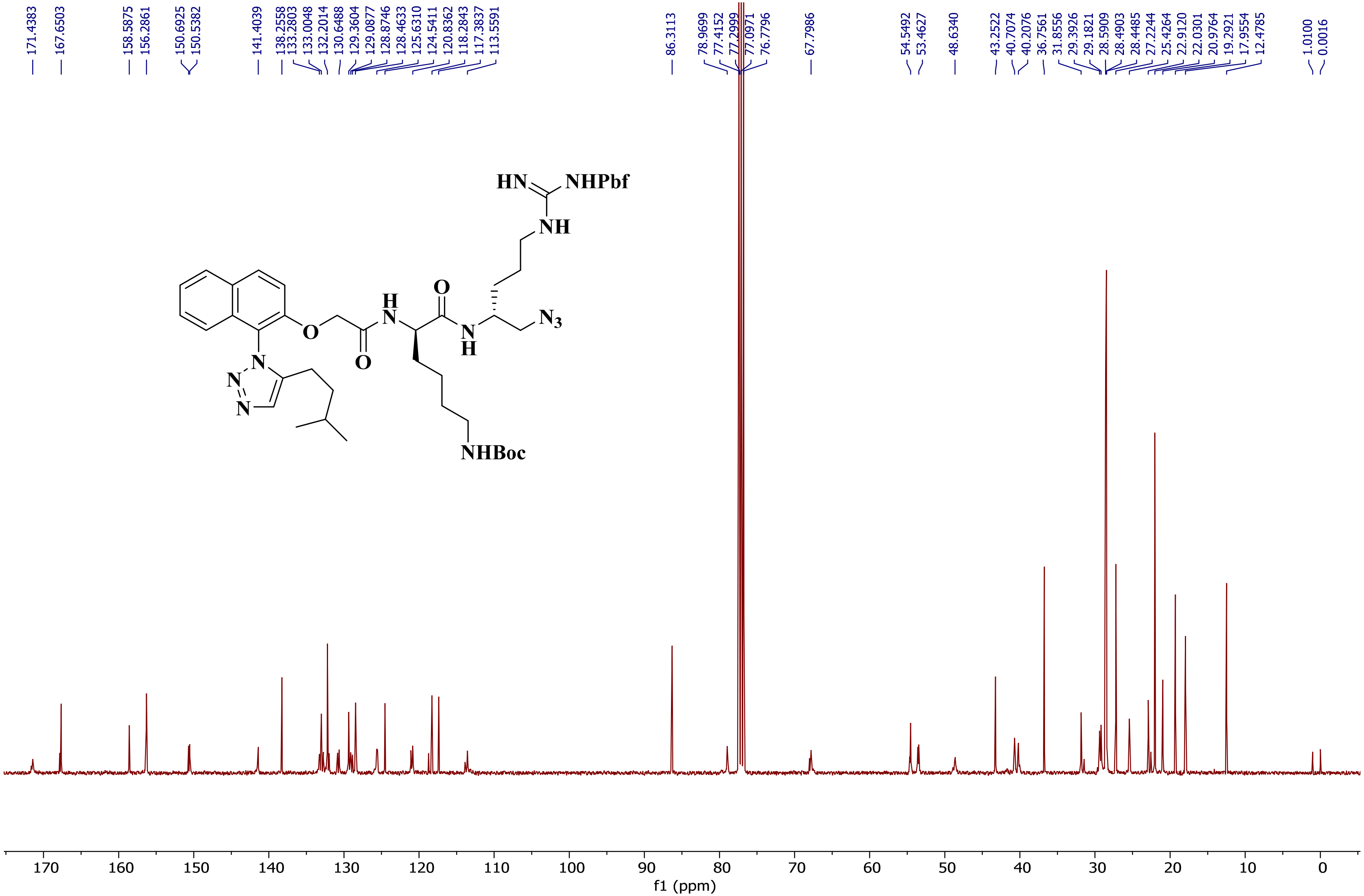

Figure S28: ^{13}C NMR of compound 33 (101 MHz, $CDCl_3$). Rotamers apparent in spectrum as evidenced by doubling of resonances.

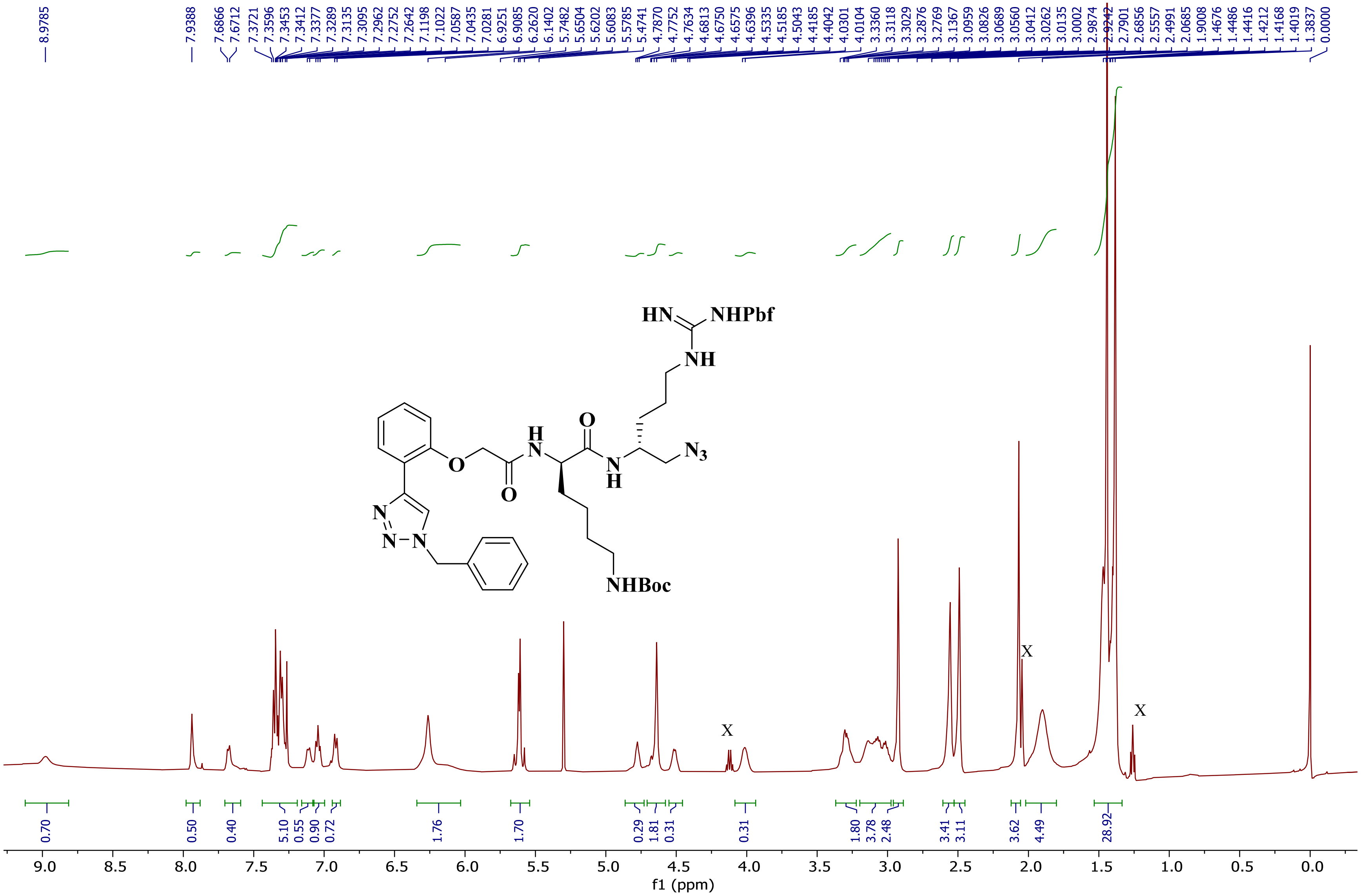

Figure S29: ^{1}H NMR of compound 34 (400 MHz, $CDCl_3$). X = ethyl acetate solvent. Rotamers apparent in spectrum as evidenced by resonance broadening.

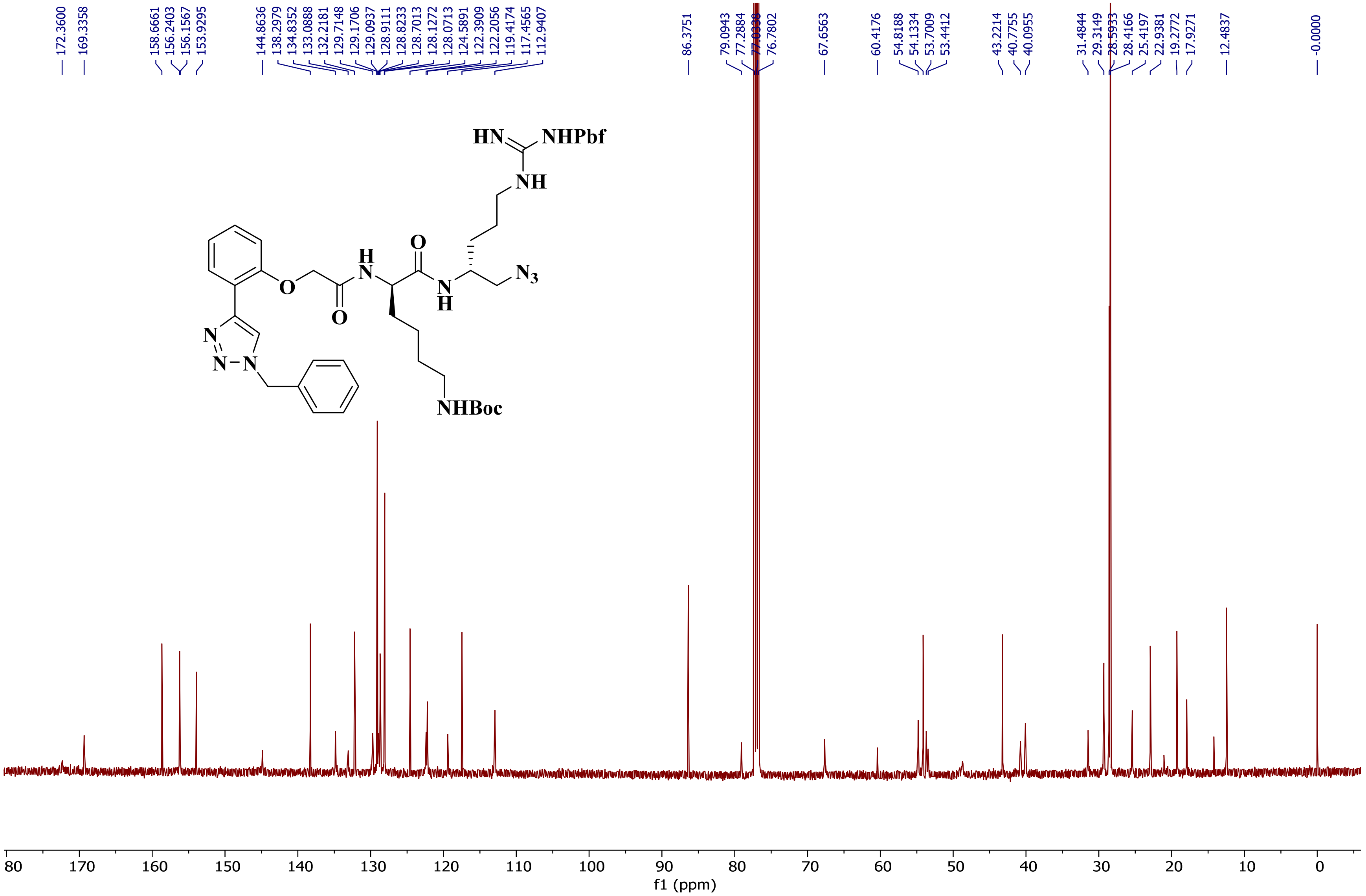

Figure S30: ^{13}C NMR of compound 34 (101 MHz, $CDCl_3$). Rotamers apparent in spectrum as evidenced by doubling of resonances.

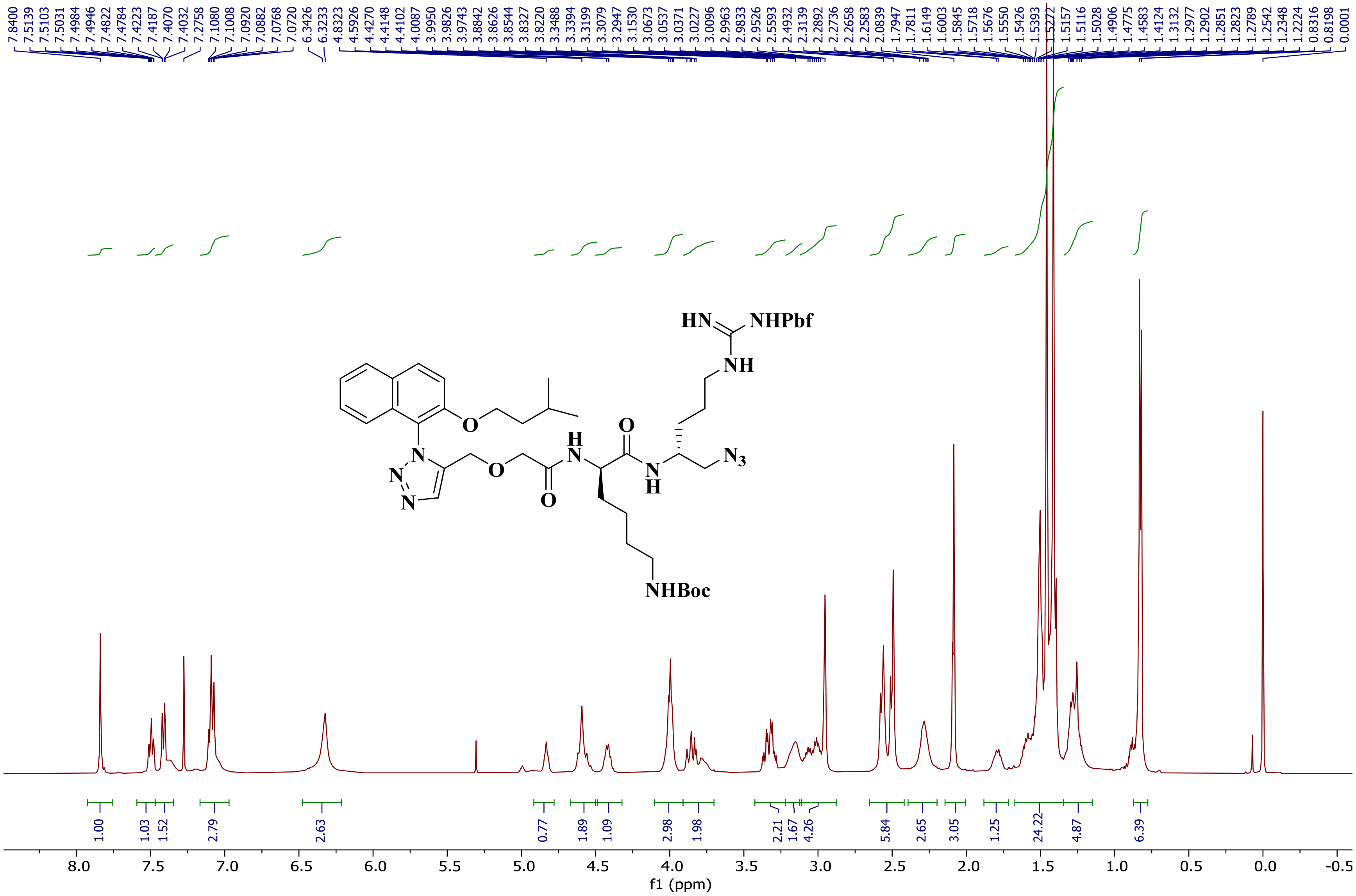

Figure S31: ^{1}H NMR of compound 35 (400 MHz, $CDCl_3$). Rotamers apparent in spectrum as evidenced by resonance broadening.

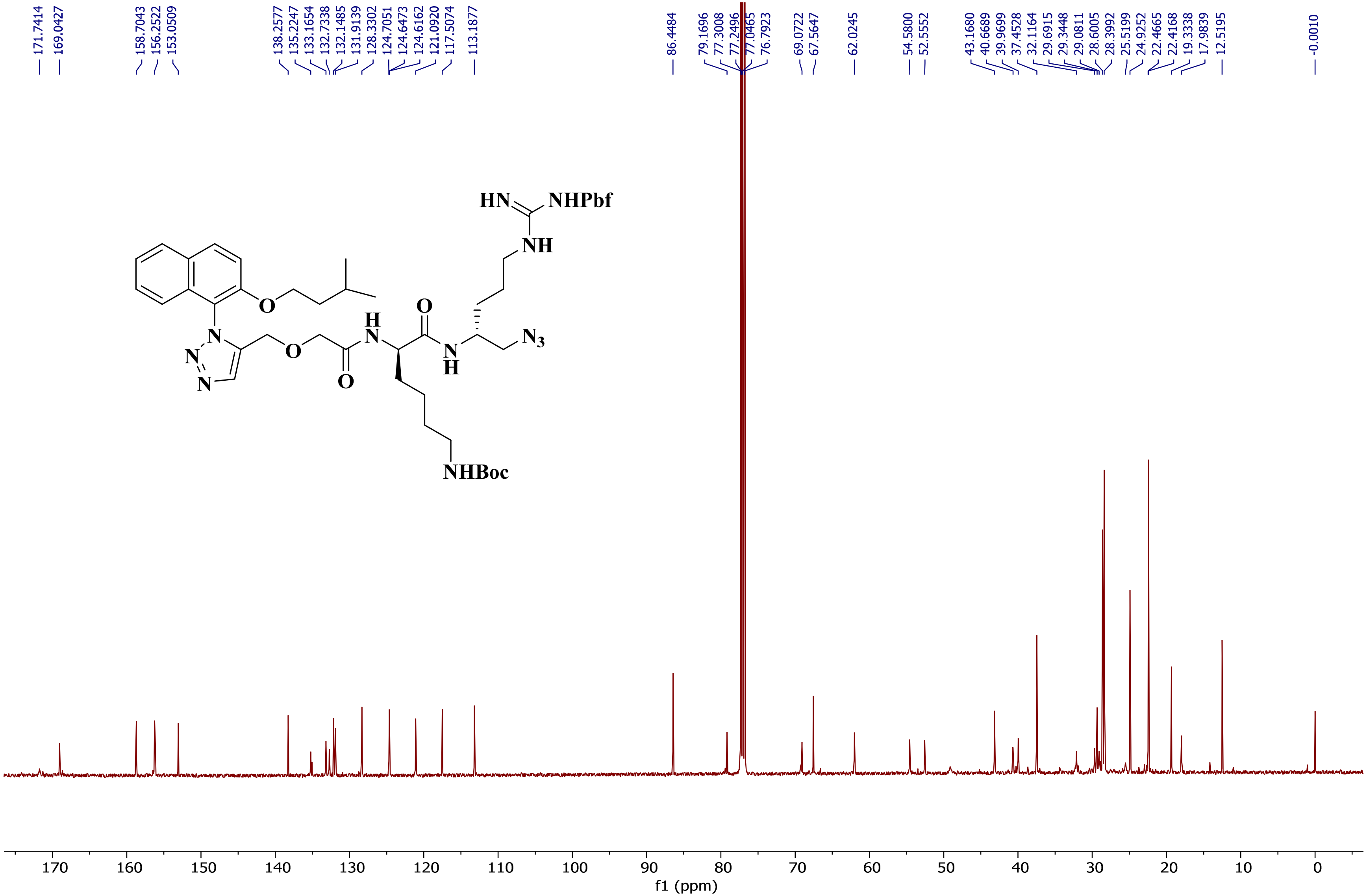

Figure S32: ^{13}C NMR of compound 35 (101 MHz, $CDCl_3$). Rotamers apparent in spectrum as evidenced by doubling of resonances.

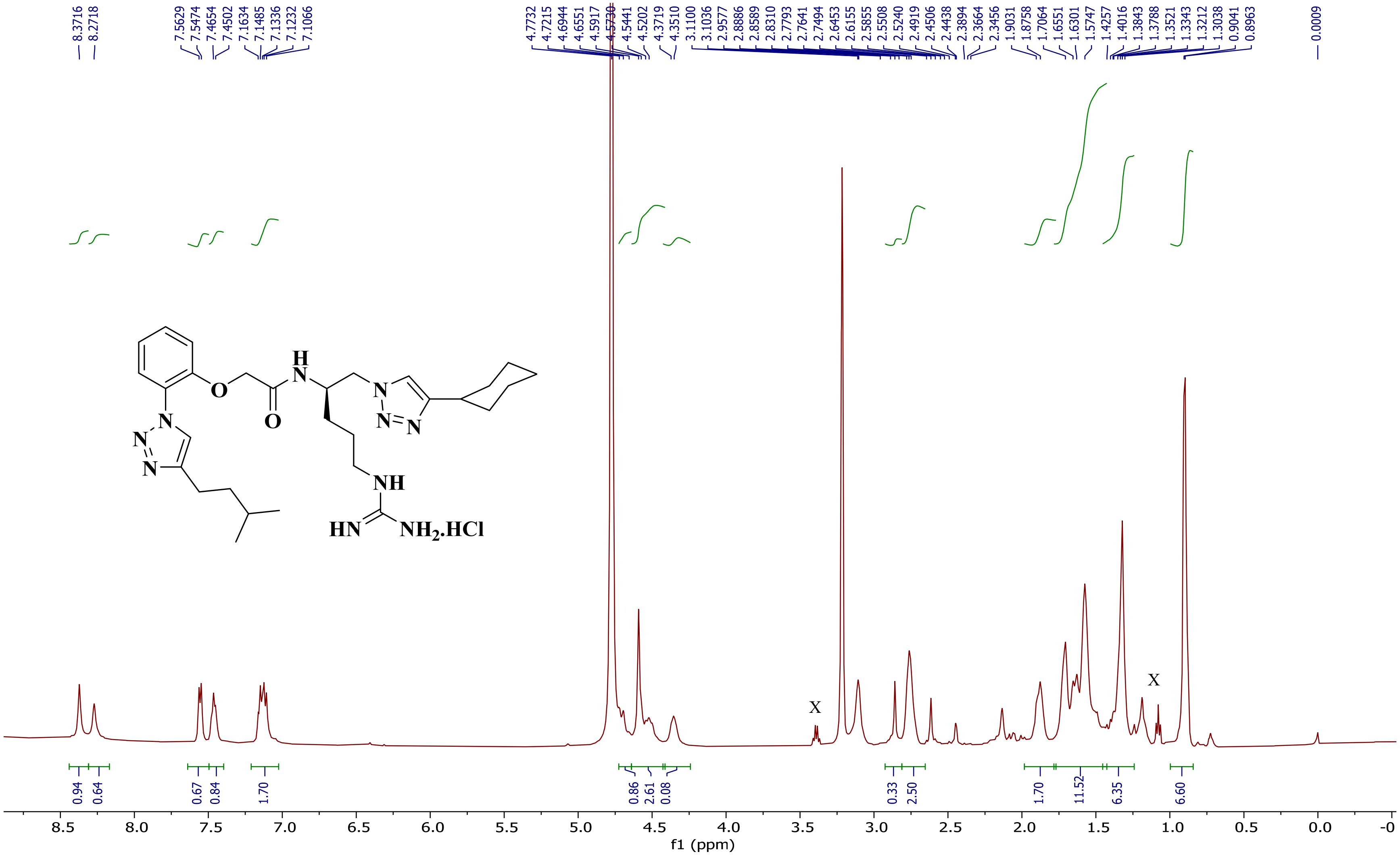

Figure S33: ^{1}H NMR of compound 10 (400 MHz, CD_3OD). X = diethyl ether solvent. Rotamers apparent in spectrum as evidenced by resonance broadening.

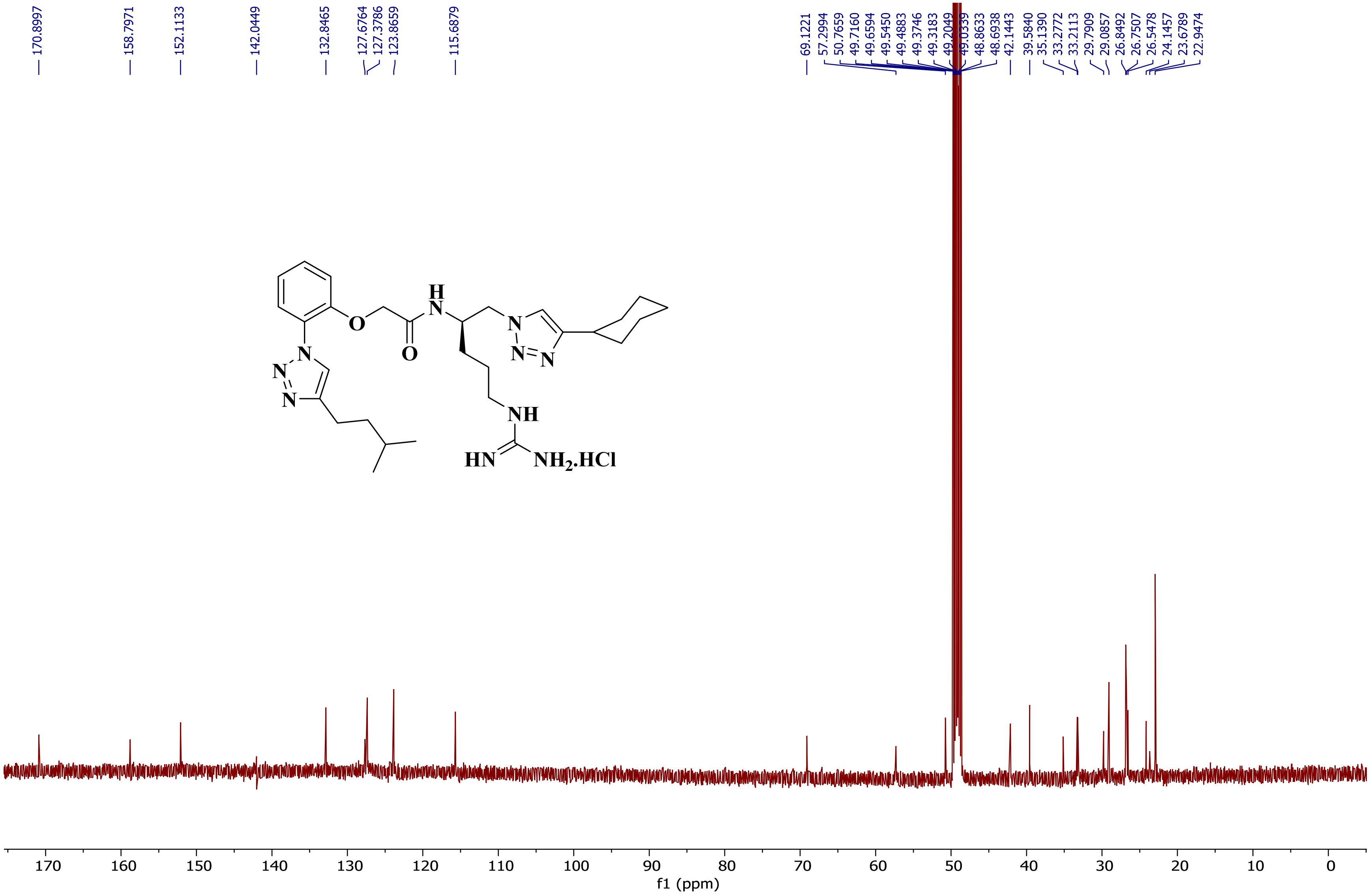

Figure S34: ^{13}C NMR of compound 10 (101 MHz, CD_3OD)

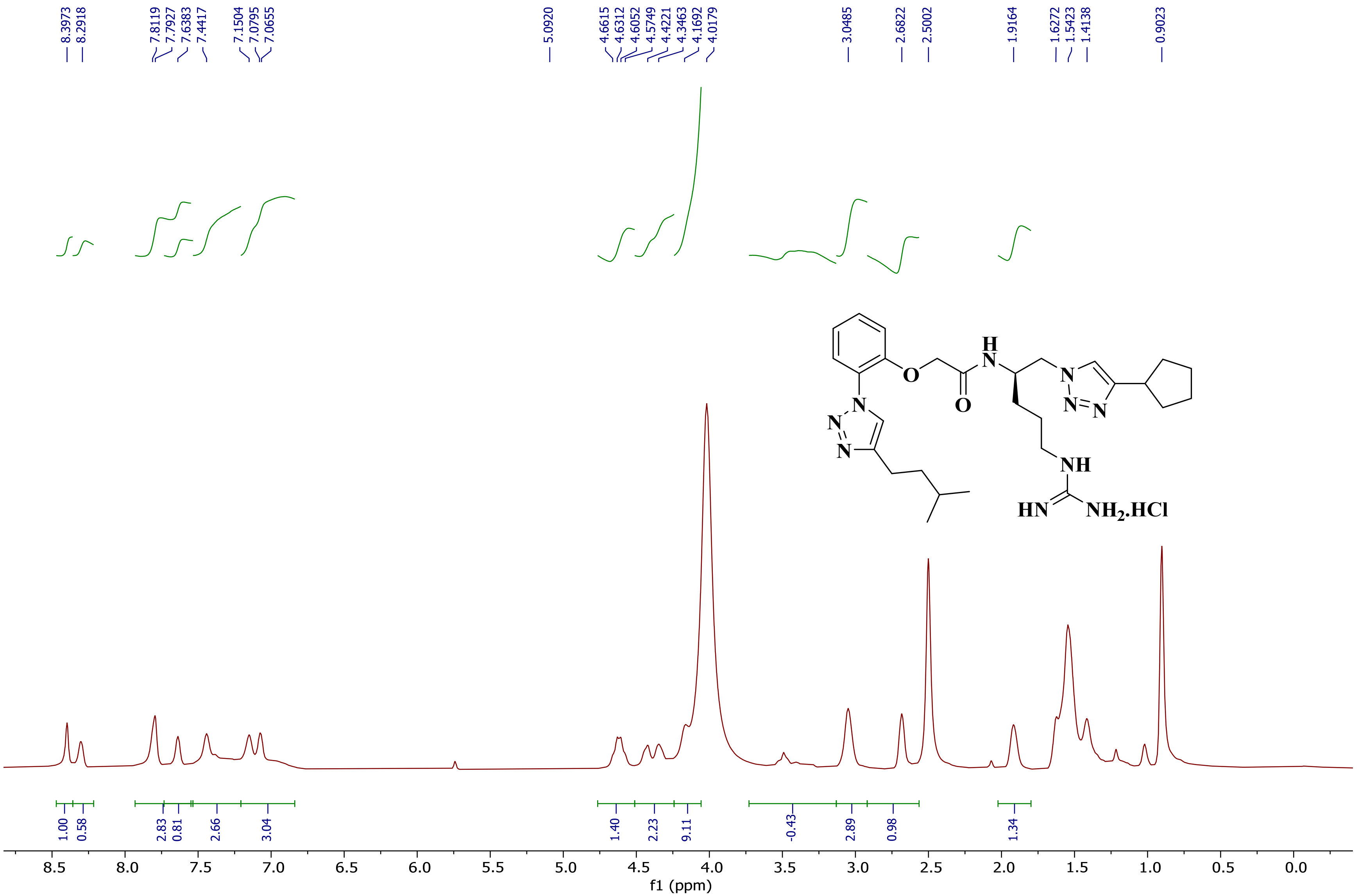

Figure S35: ^{1}H NMR of compound 11 (400 MHz, DMSO-d$_6$). Rotamers apparent in spectrum as evidenced by resonance broadening.

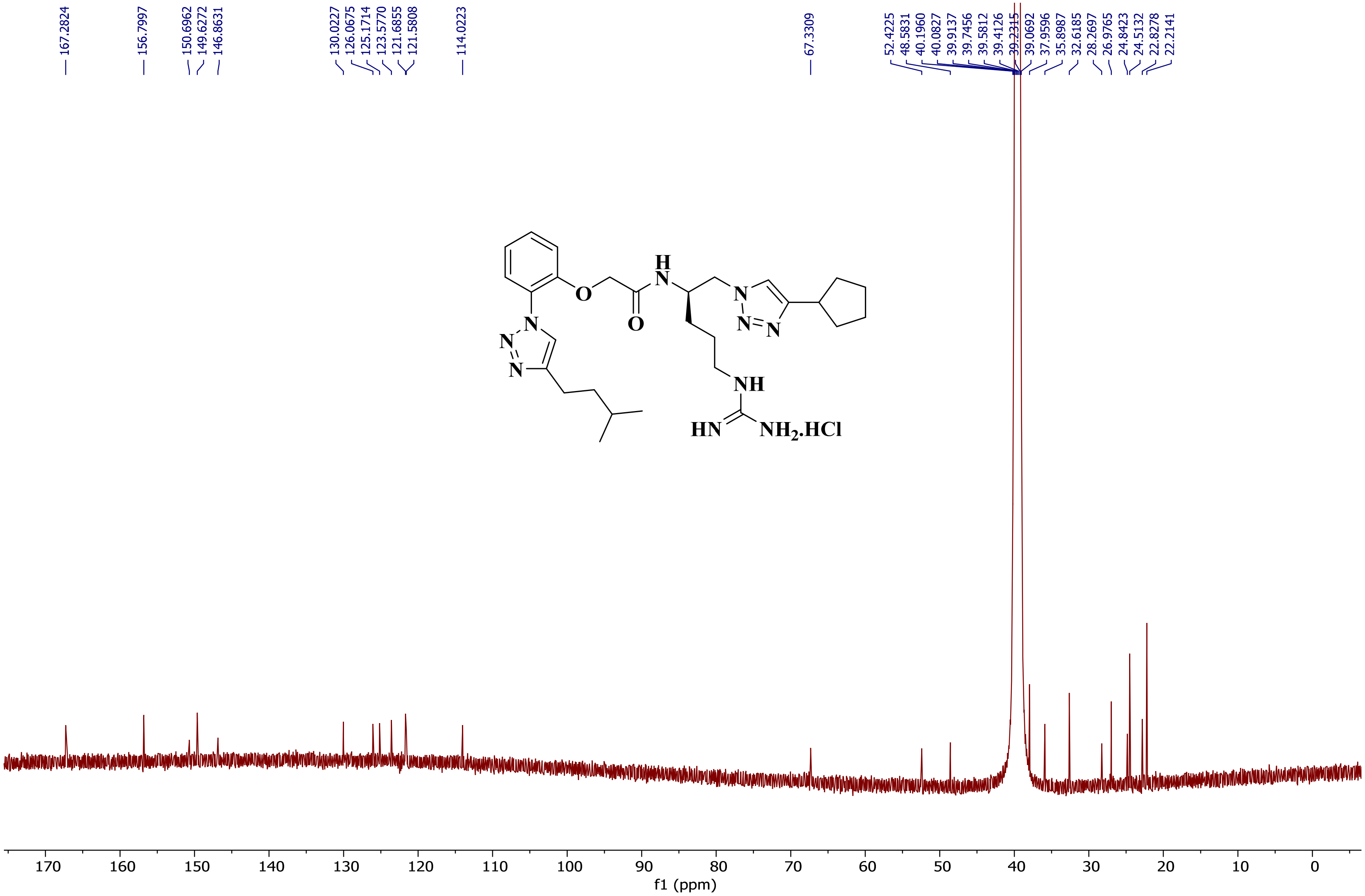

Figure S36: ^{13}C NMR of compound 11 (101 MHz, DMSO-d$_6$).

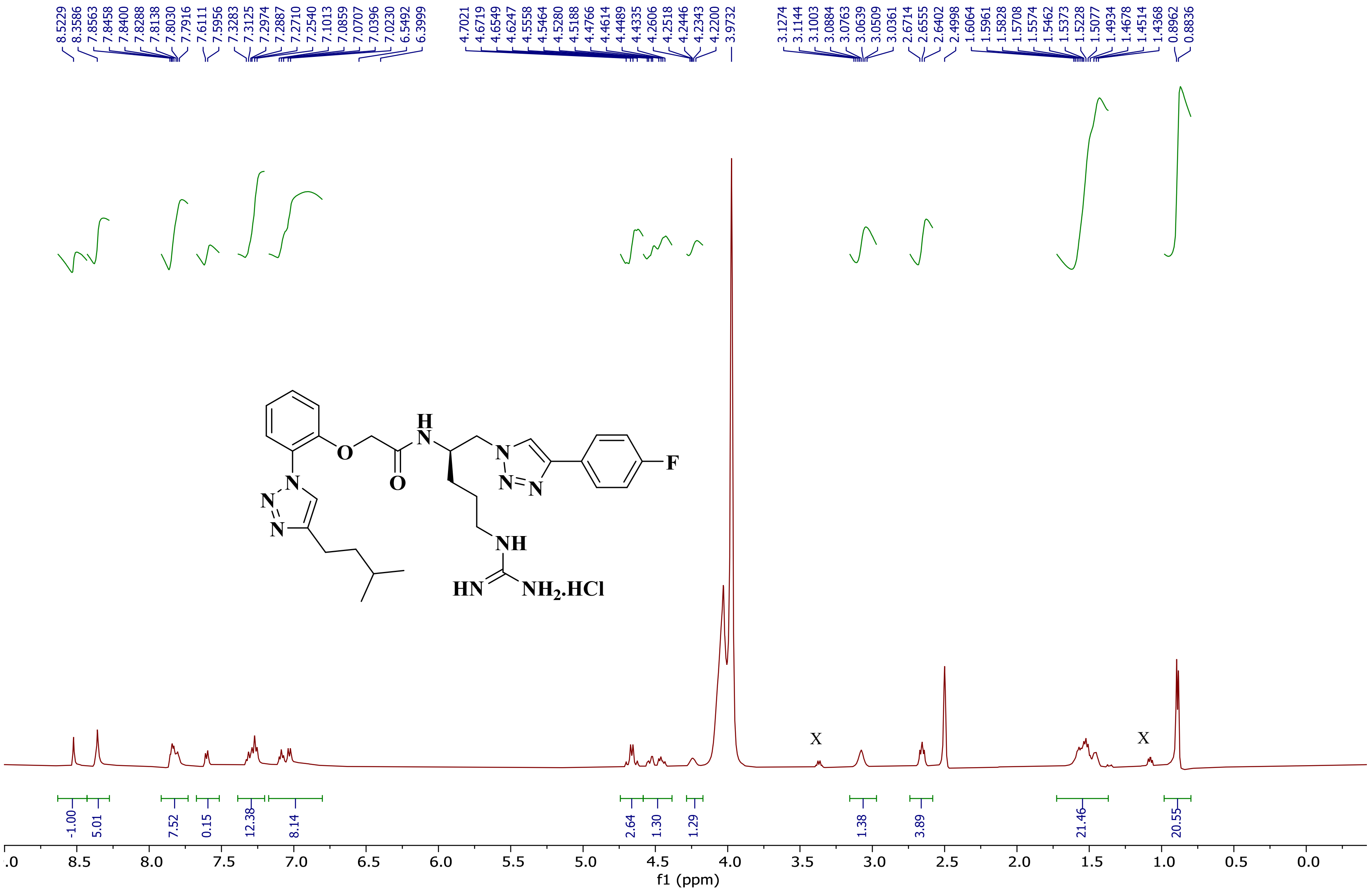

Figure S37: ^{1}H NMR of compound 12 (400 MHz, DMSO-d_6). X = diethyl ether solvent.

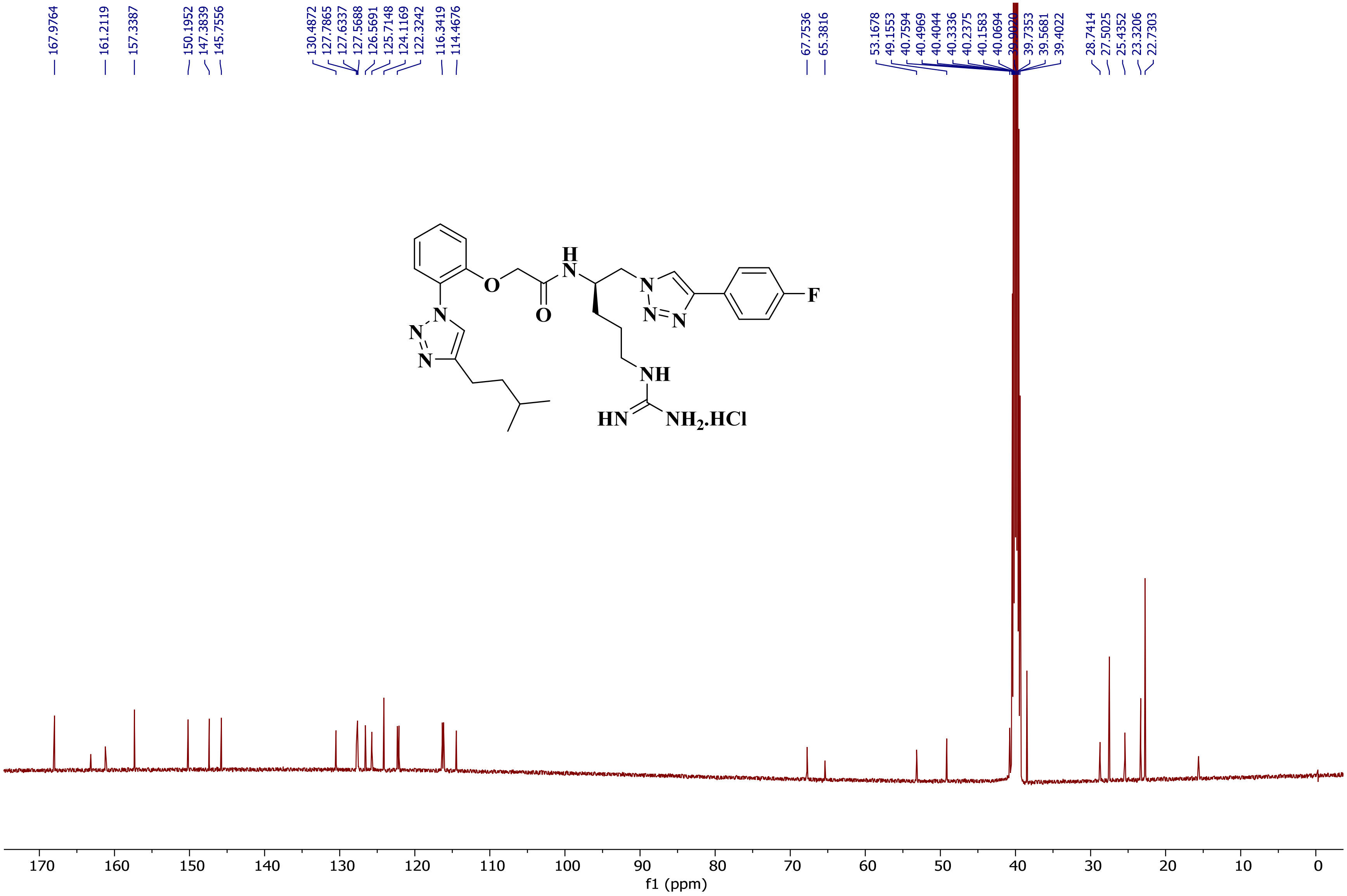

Figure S38: ^{13}C NMR of compound 12 (101 MHz, DMSO-d$_6$)

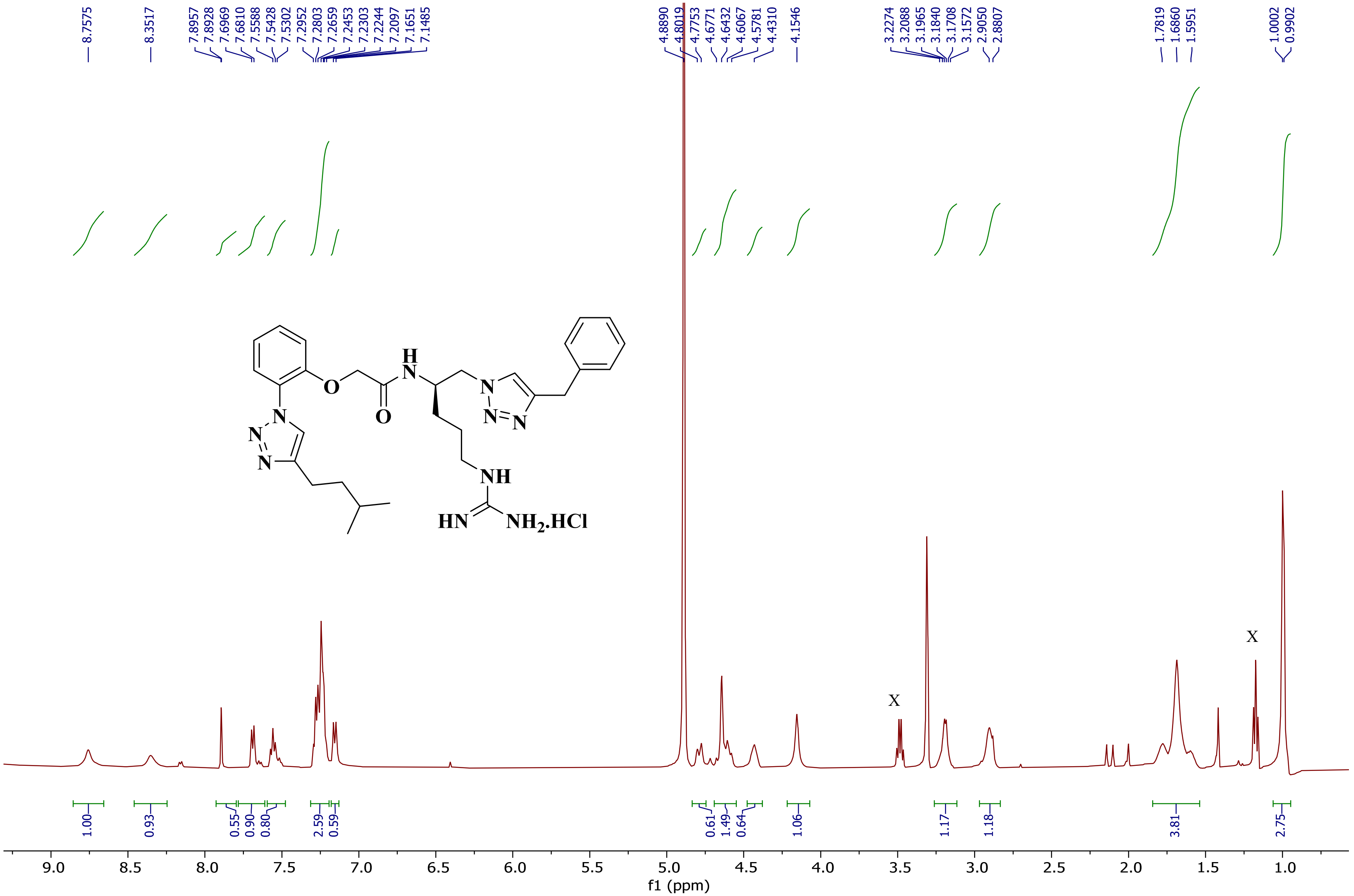

Figure S39: ^{1}H NMR of compound 13 (400 MHz, CD_3OD). X = diethyl ether solvent. Rotamers apparent in spectrum as evidenced by resonance broadening.

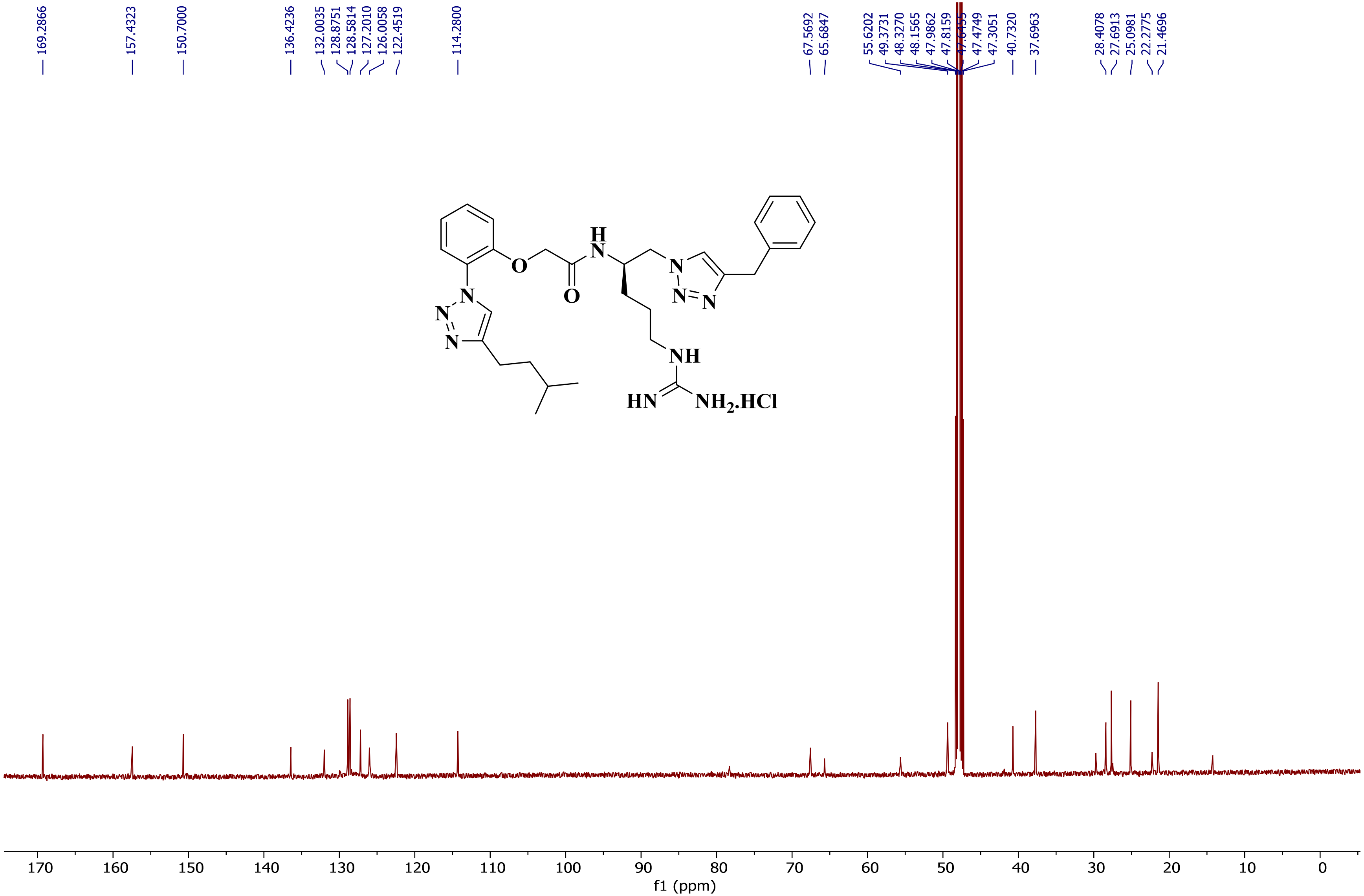

Figure S40: ^{13}C NMR of compound 13 (101 MHz, CD_3OD).

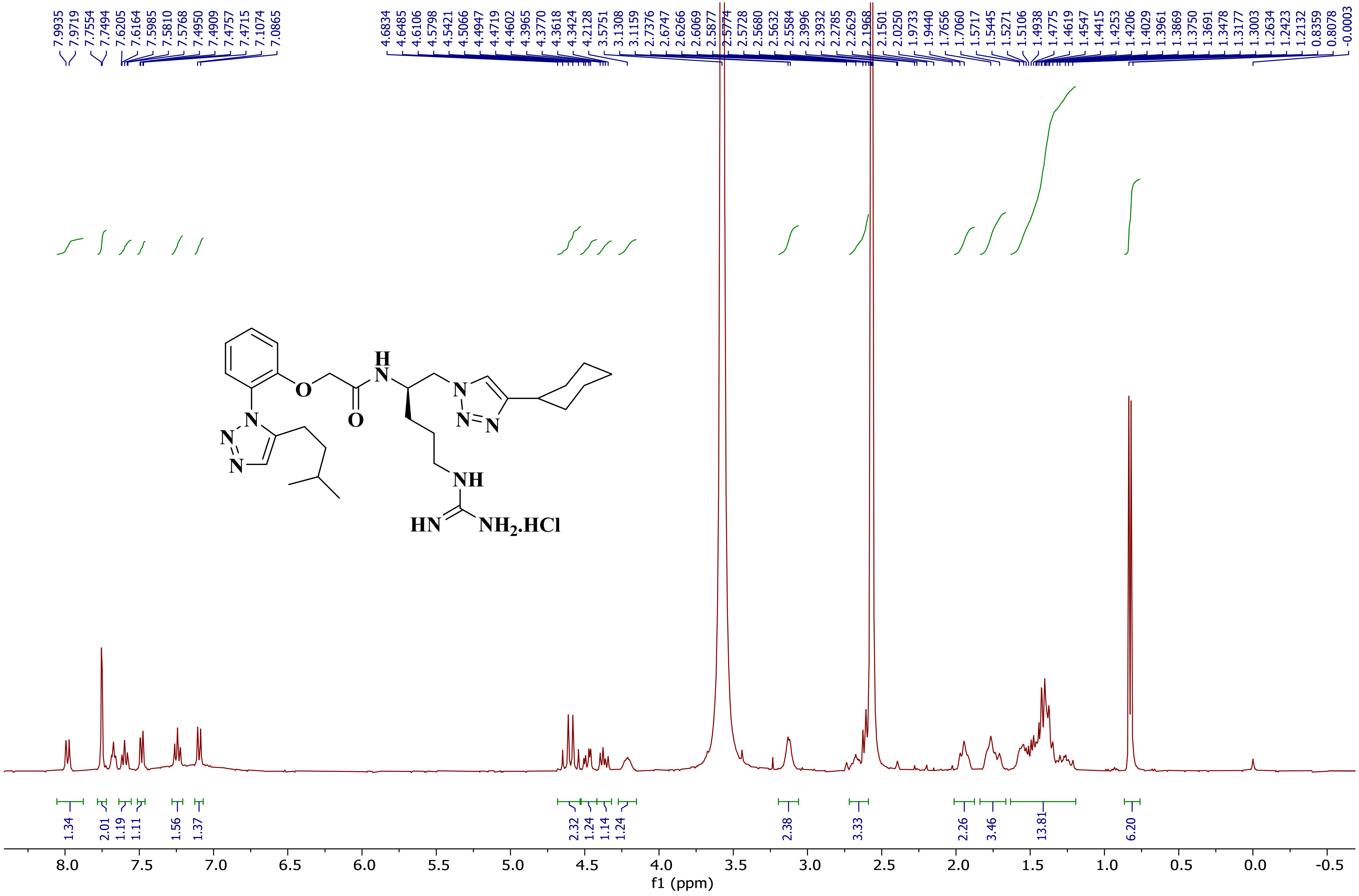

Figure S41: ^{1}H NMR of compound 14 (400 MHz, DMSO-d$_6$).

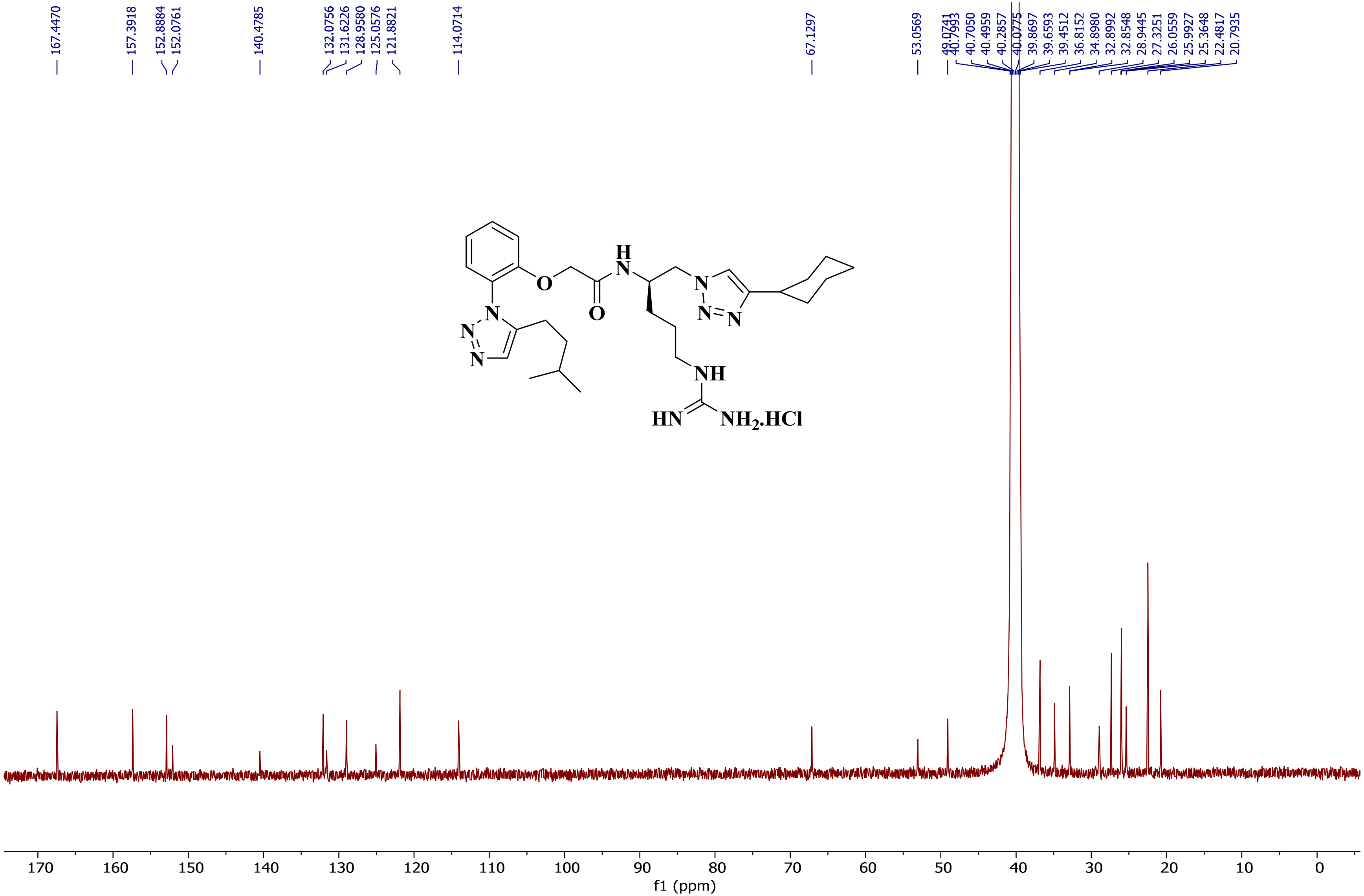

Figure S42: ^{13}C NMR of compound 14 (101 MHz, DMSO-d_6).

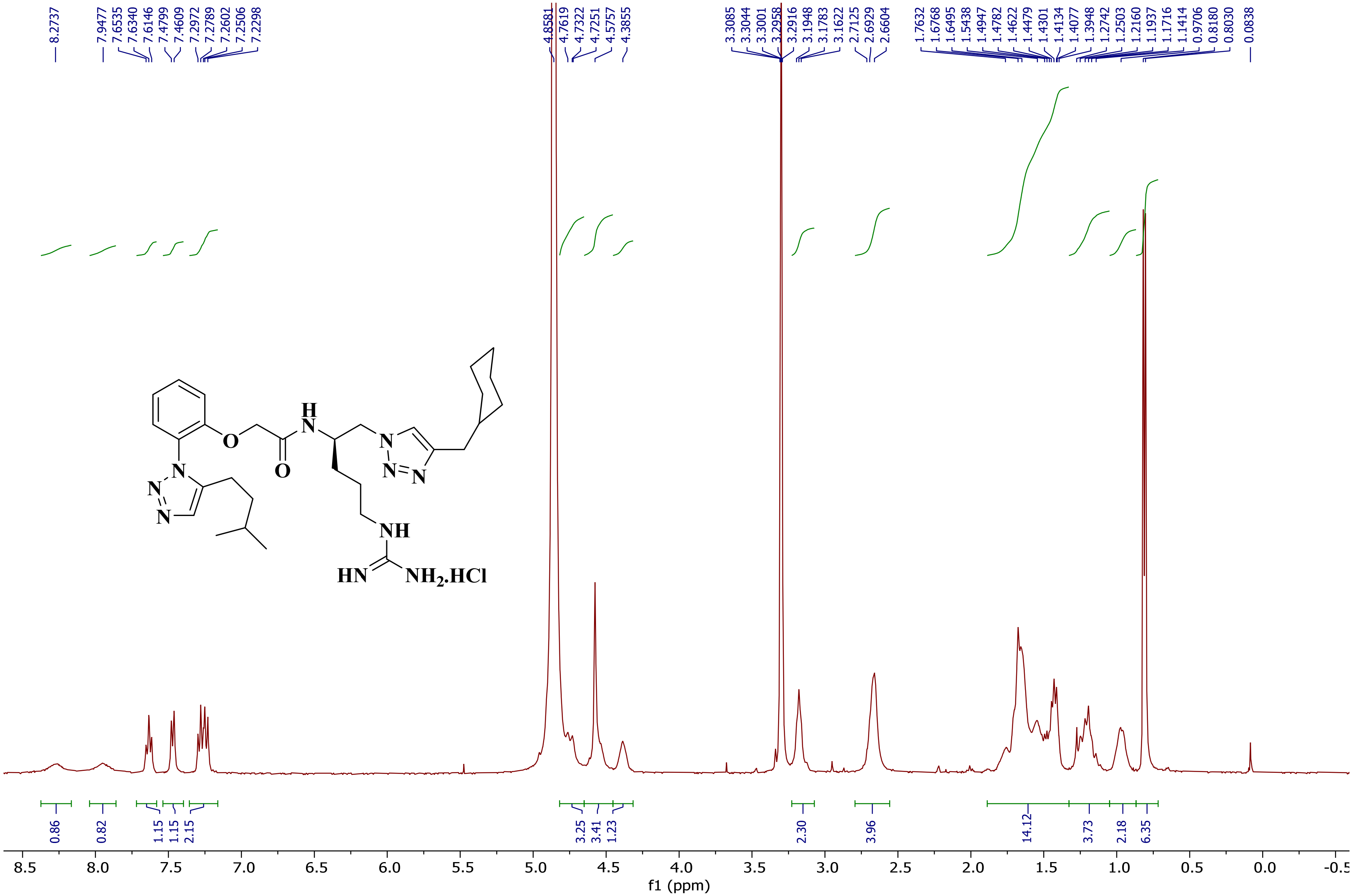

Figure S43: ^{1}H NMR of compound 15 (400 MHz, CD_3OD). Rotamers apparent in spectrum as evidenced by resonance broadening.

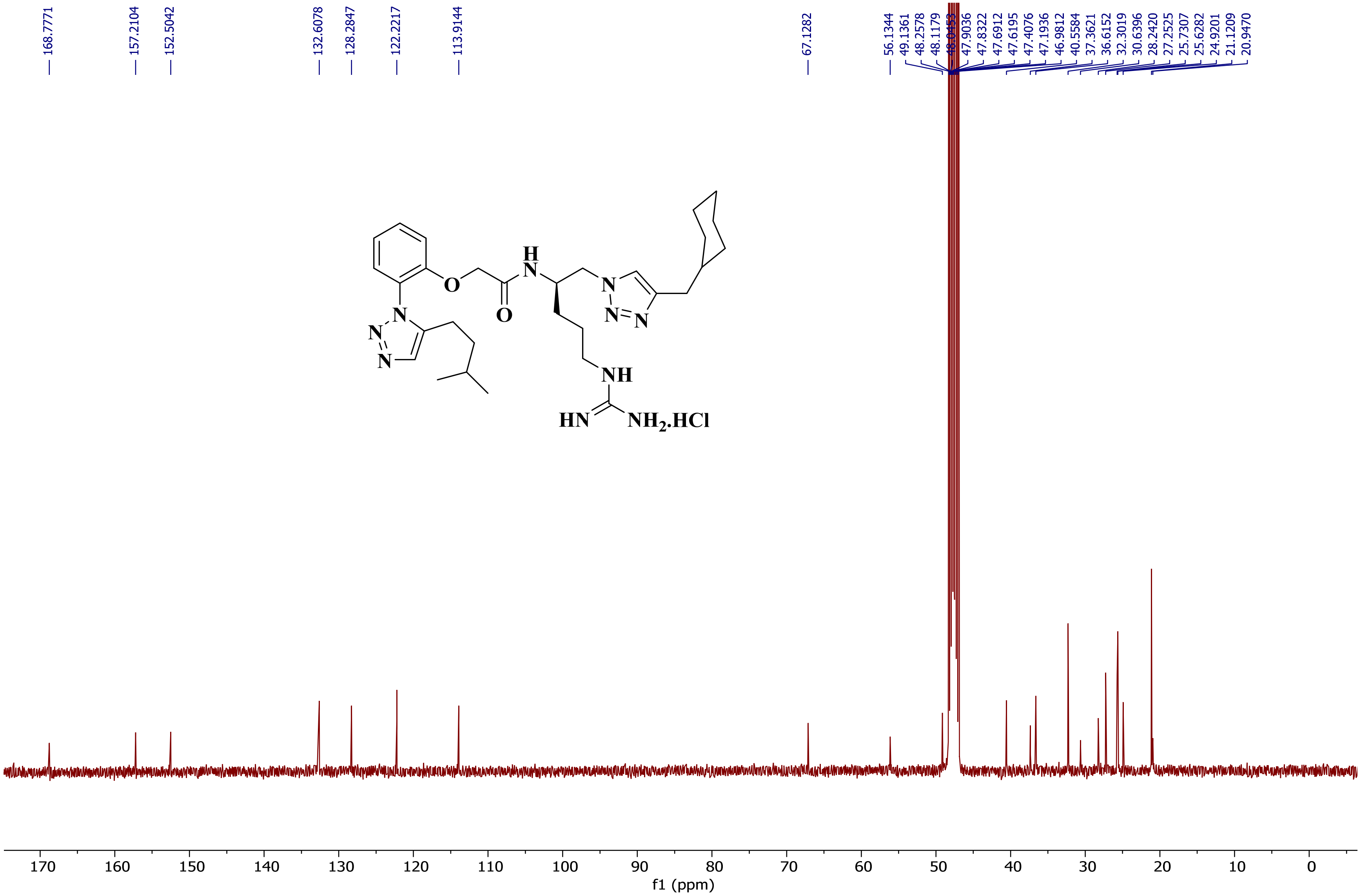

Figure S44: ^{13}C NMR of compound 15 (101 MHz, CD_3OD)

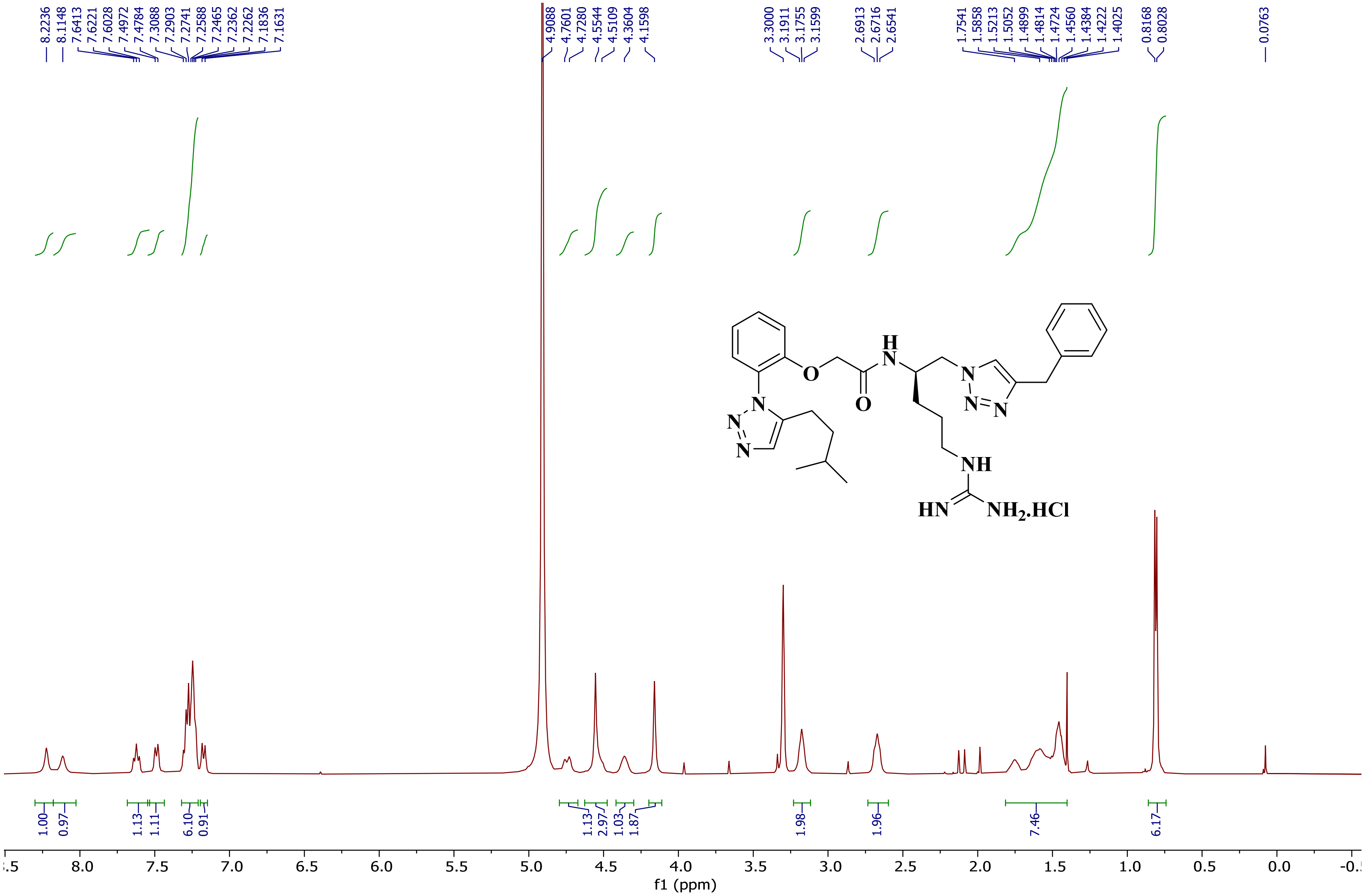

Figure S45: ^{1}H NMR of compound 16 (400 MHz, CD_3OD). Rotamers apparent in spectrum as evidenced by resonance broadening.

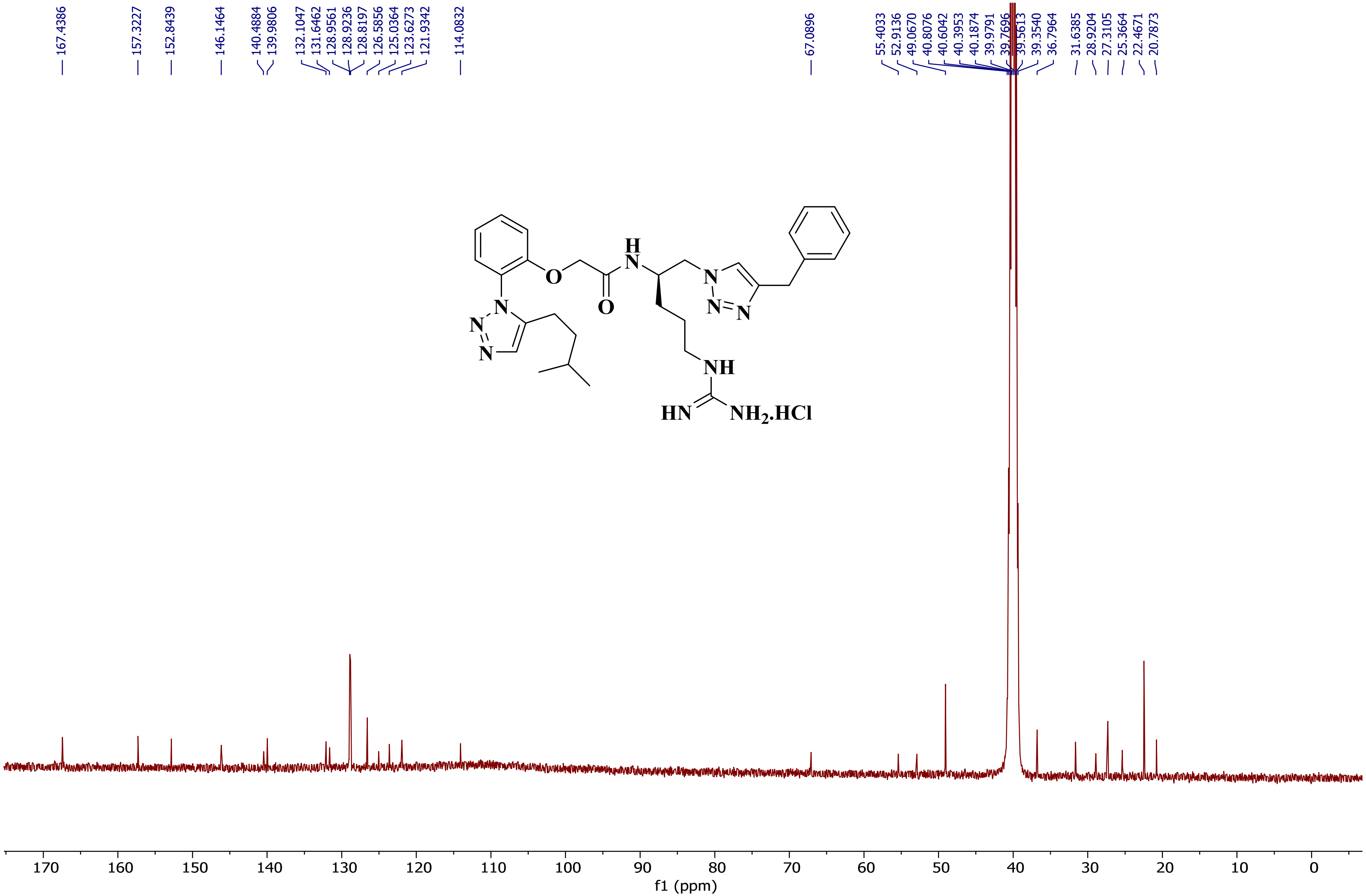

Figure S46: ^{13}C NMR of compound 16 (101 MHz, DMSO-d$_6$).

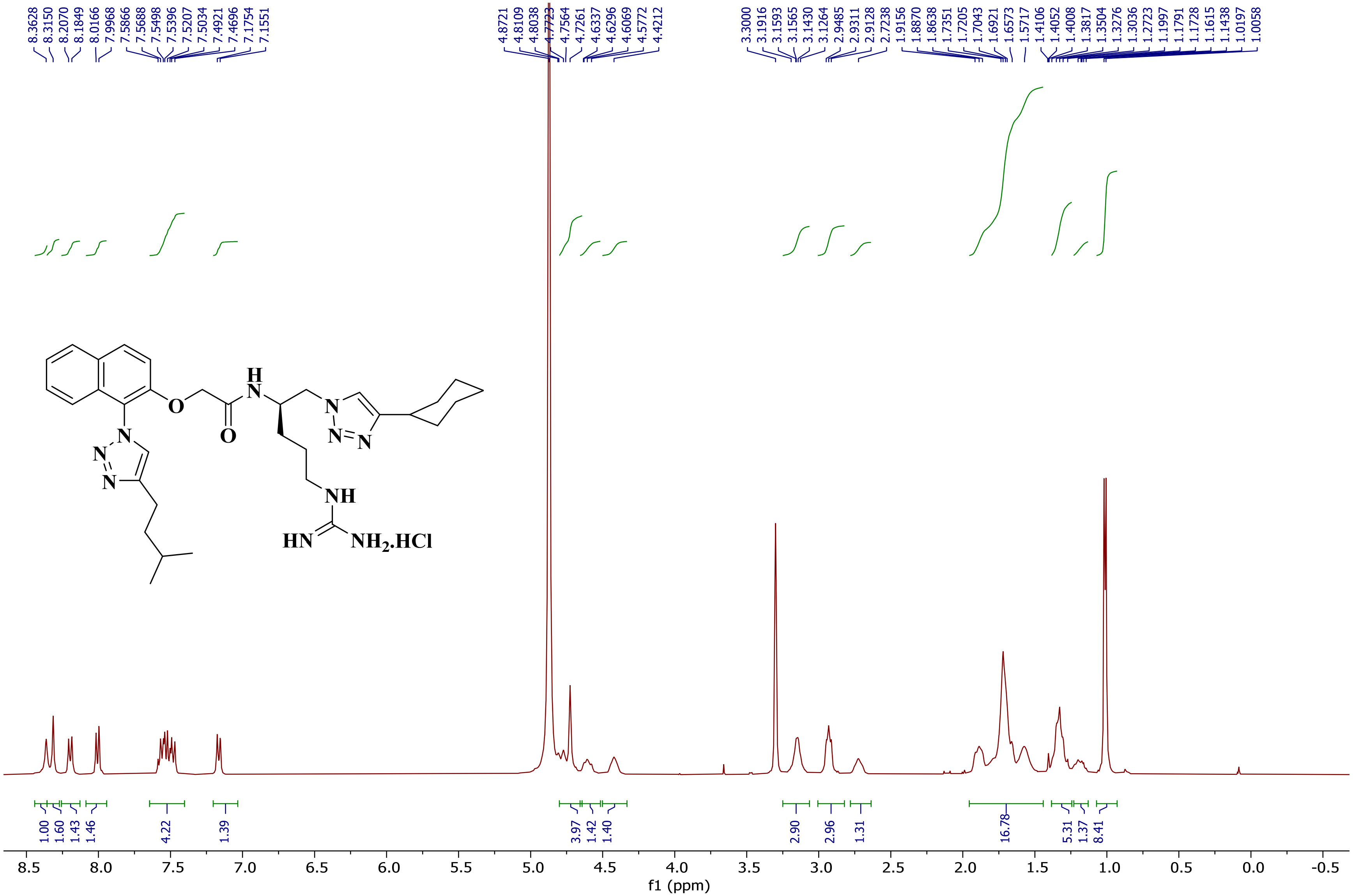

Figure S47: ^{1}H NMR of compound 21 (400 MHz, CD_3OD). Rotamers apparent in spectrum as evidenced by resonance broadening.

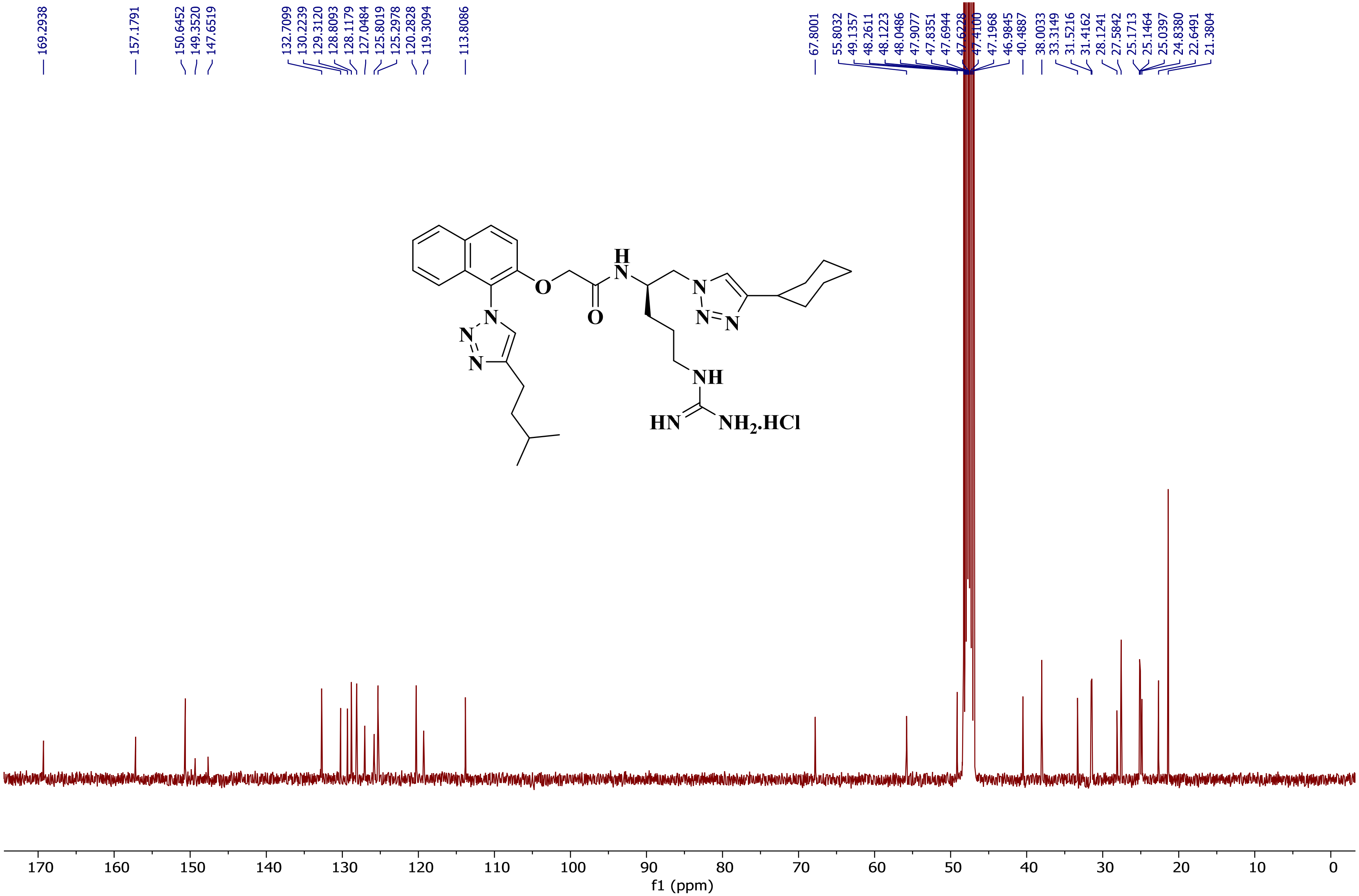

Figure S48: ^{13}C NMR of compound 21 (101 MHz, CD_3OD)

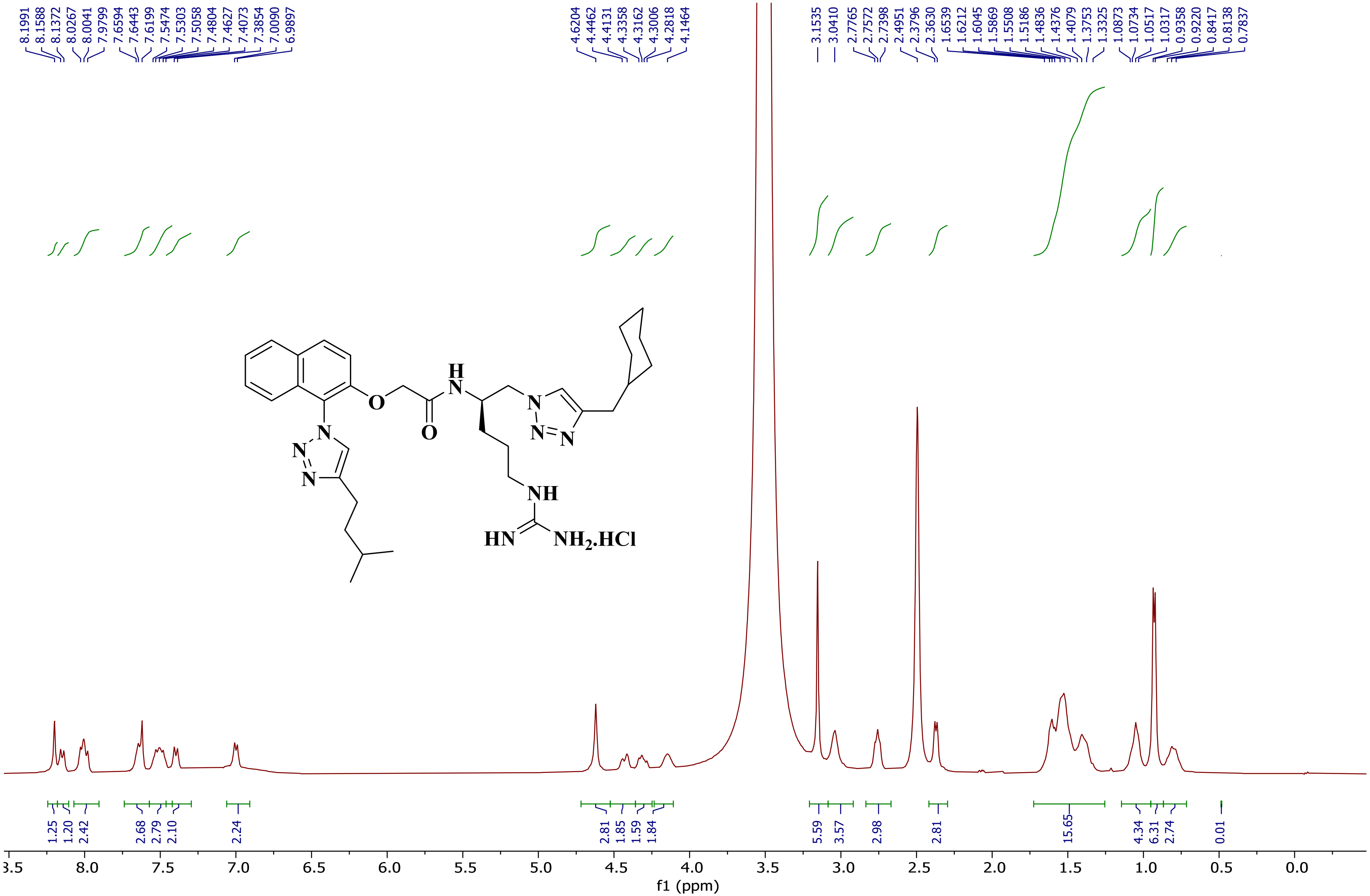

Figure S49: ^{1}H NMR of compound 22 (400 MHz, DMSO-d$_6$). Rotamers apparent in spectrum as evidenced by resonance broadening.

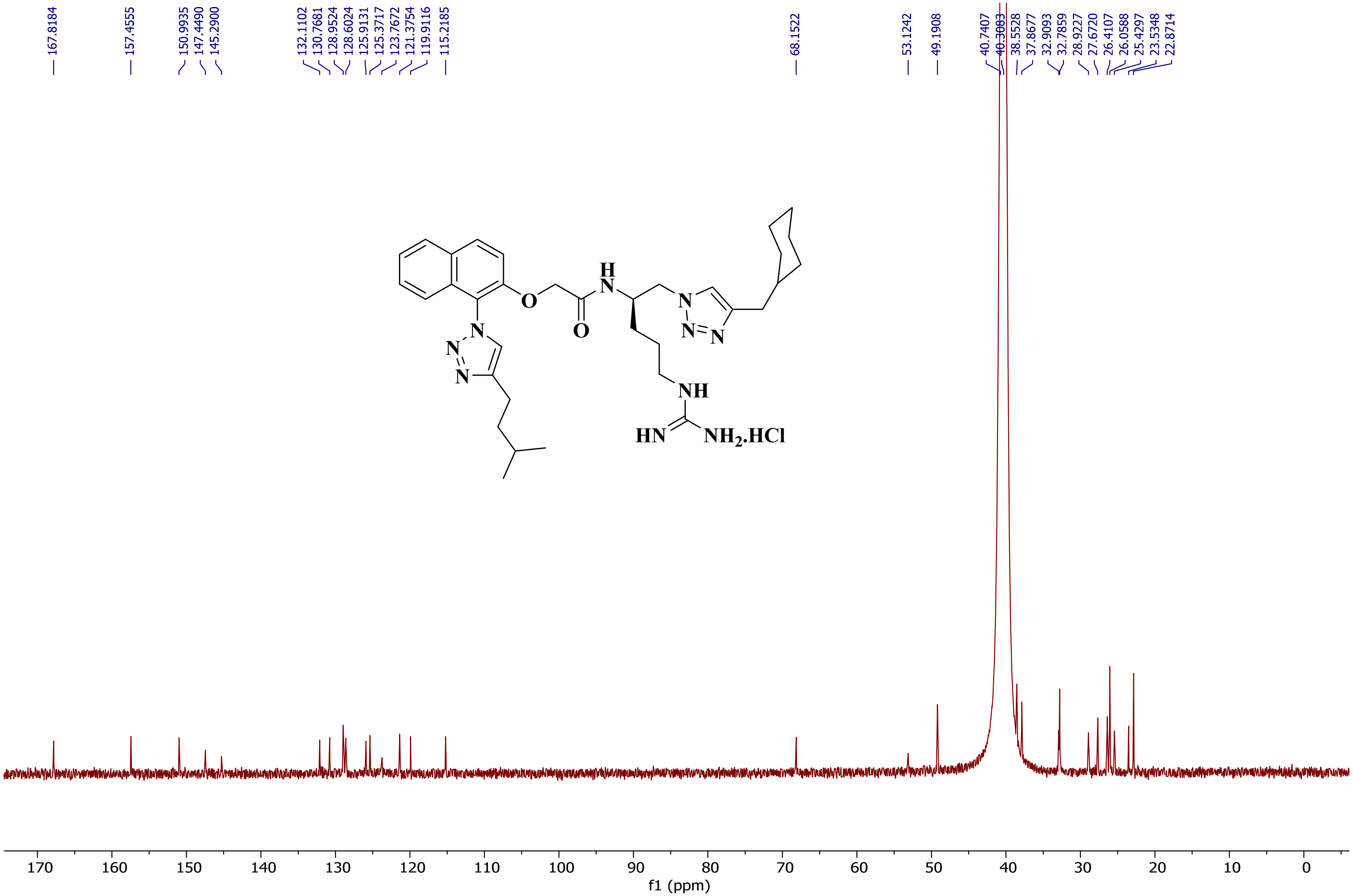

Figure S50: ^{13}C NMR of compound 22 (101 MHz, DMSO-d$_6$)

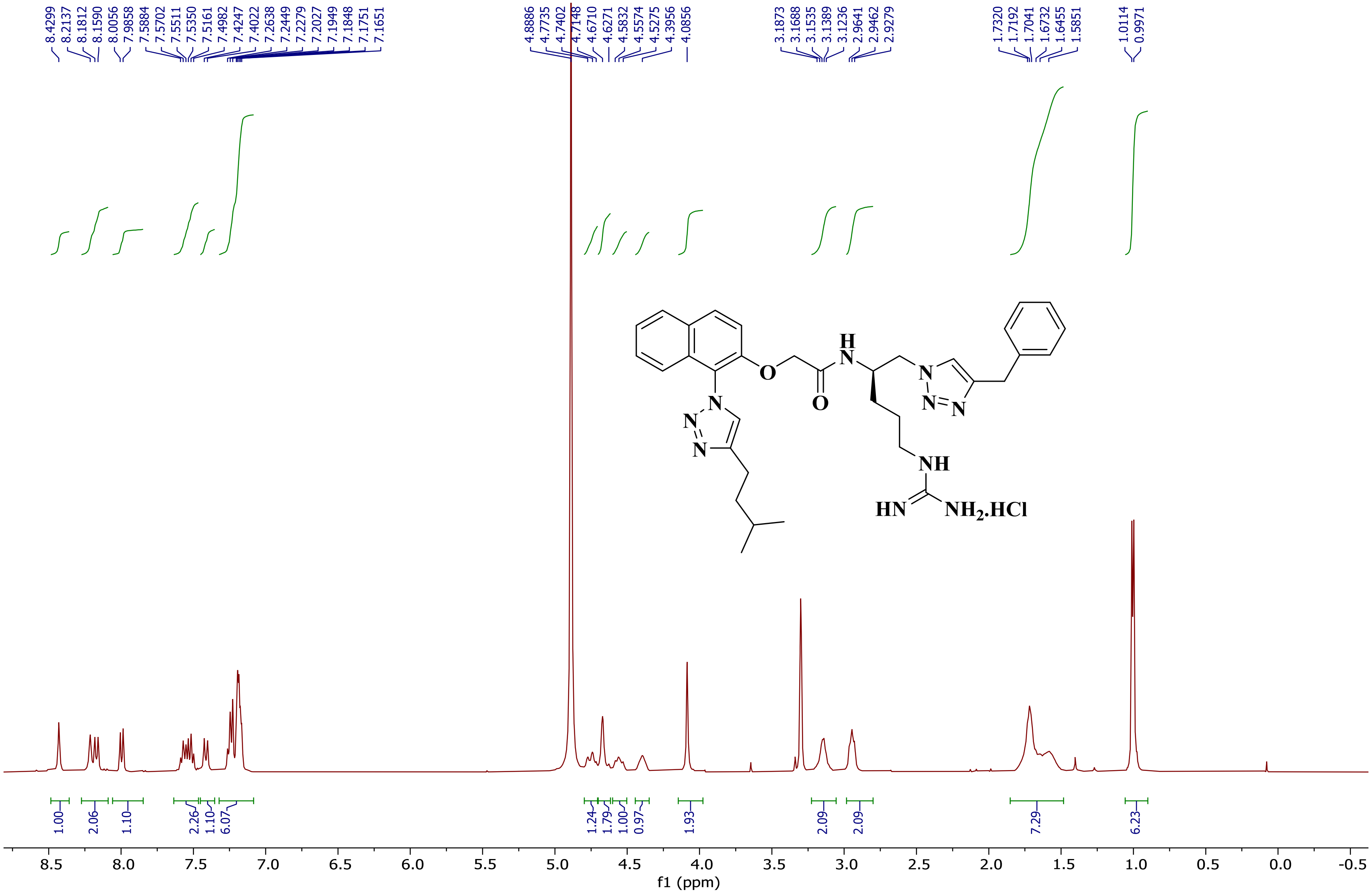

Figure S51: ^{1}H NMR of compound 23 (400 MHz, CD_3OD). Rotamers apparent in spectrum as evidenced by resonance broadening.

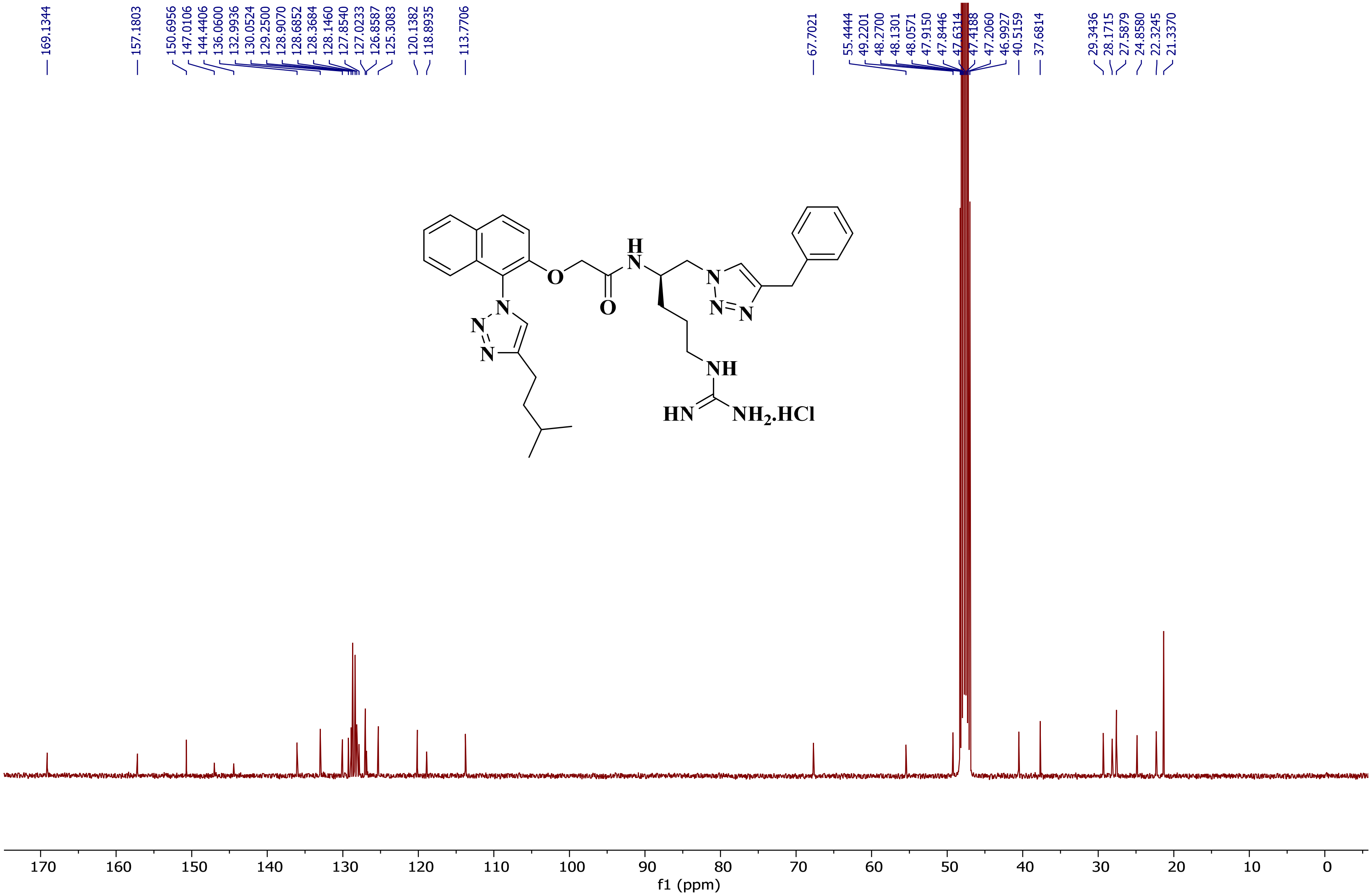

Figure S52: ^{13}C NMR of compound 23 (101 MHz, CD_3OD)

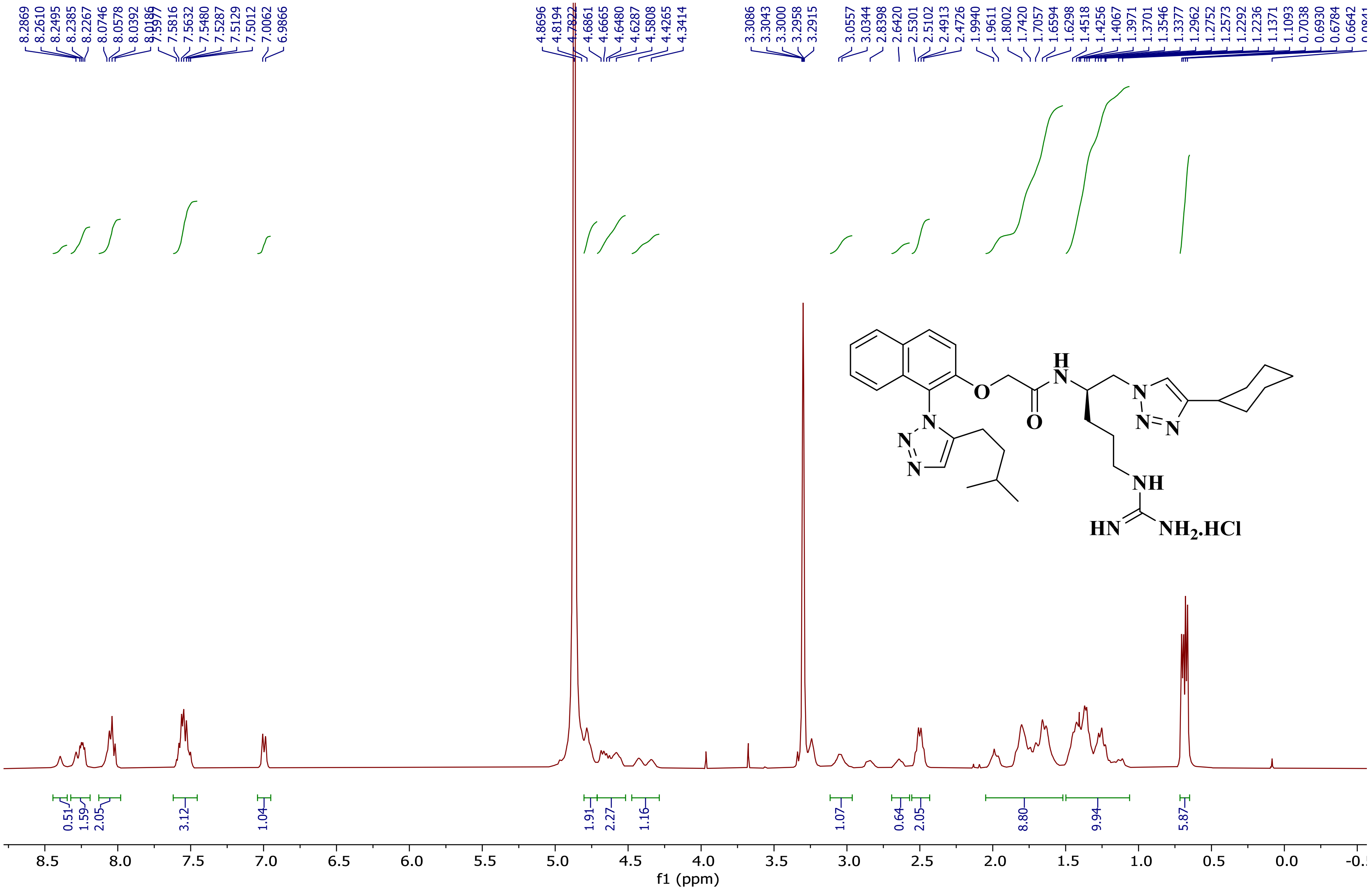

Figure S53: ^{1}H NMR of compound 24 (400 MHz, CD_3OD). Rotamers apparent in spectrum as evidenced by resonance broadening.

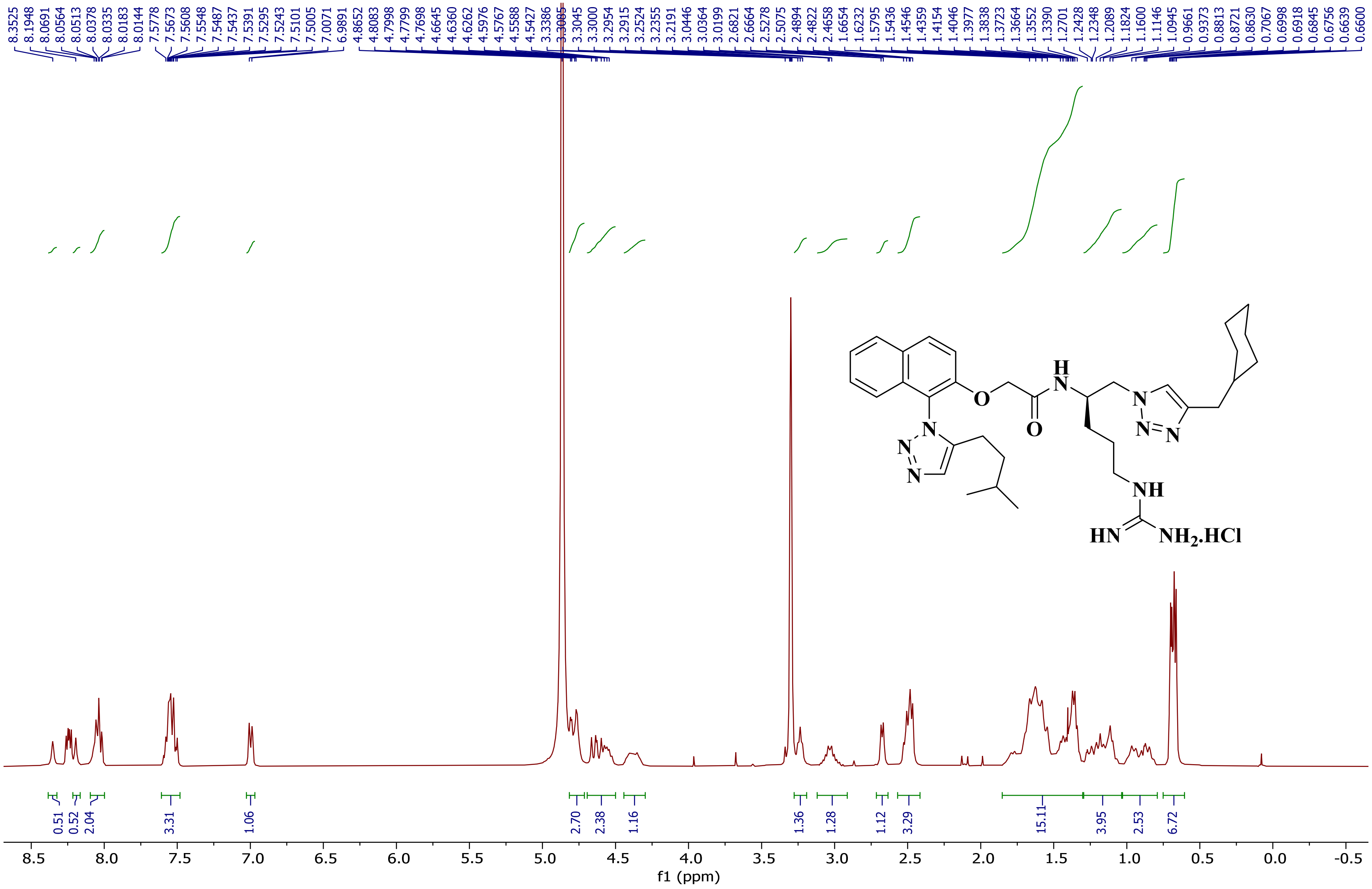

Figure S54: ^{1}H NMR of compound 25 (400 MHz, CD_3OD). Rotamers apparent in spectrum as evidenced by resonance broadening.

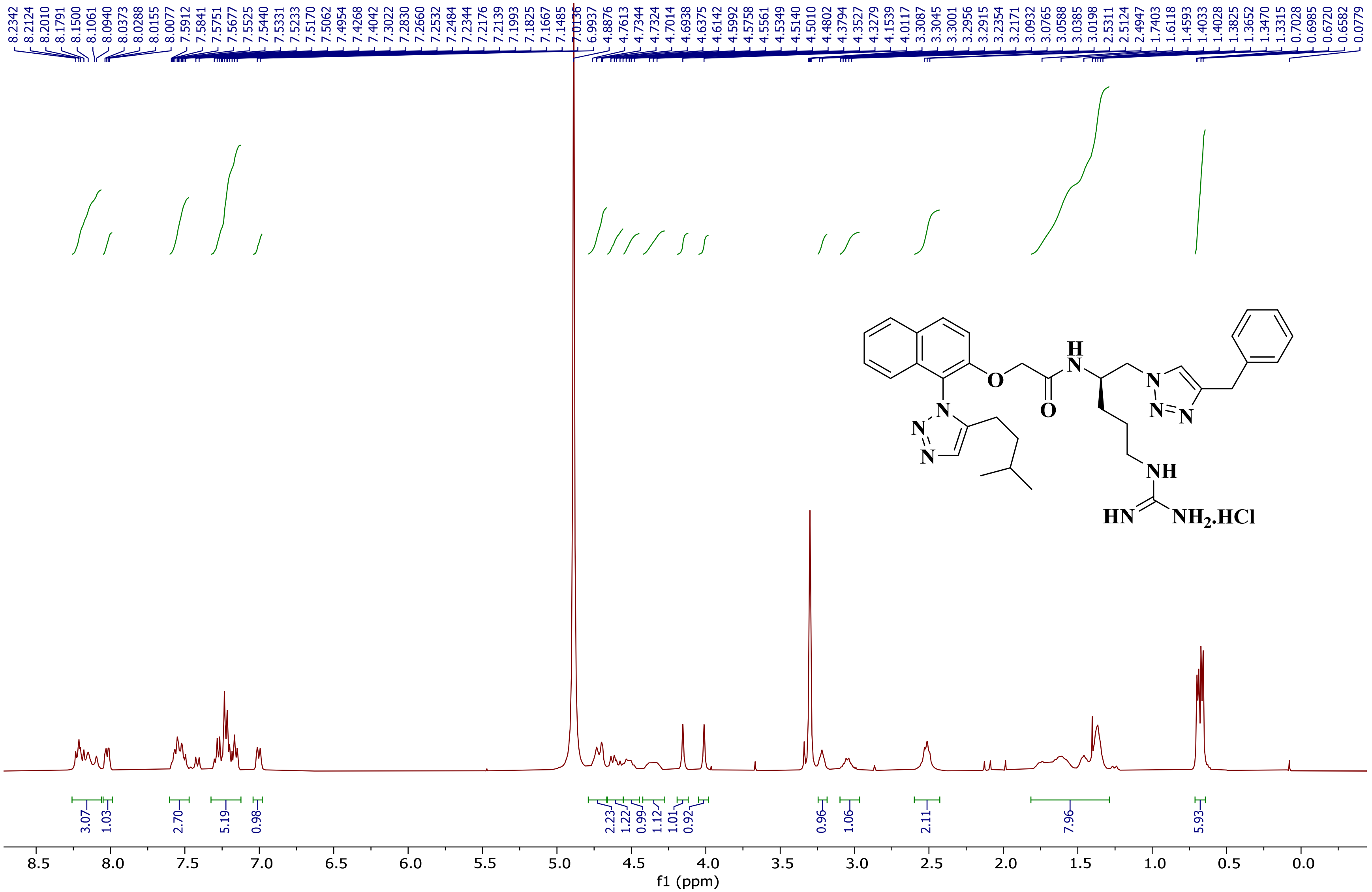

Figure S55: ^{1}H NMR of compound 26 (400 MHz, CD_3OD). Rotamers apparent in spectrum as evidenced by resonance broadening.

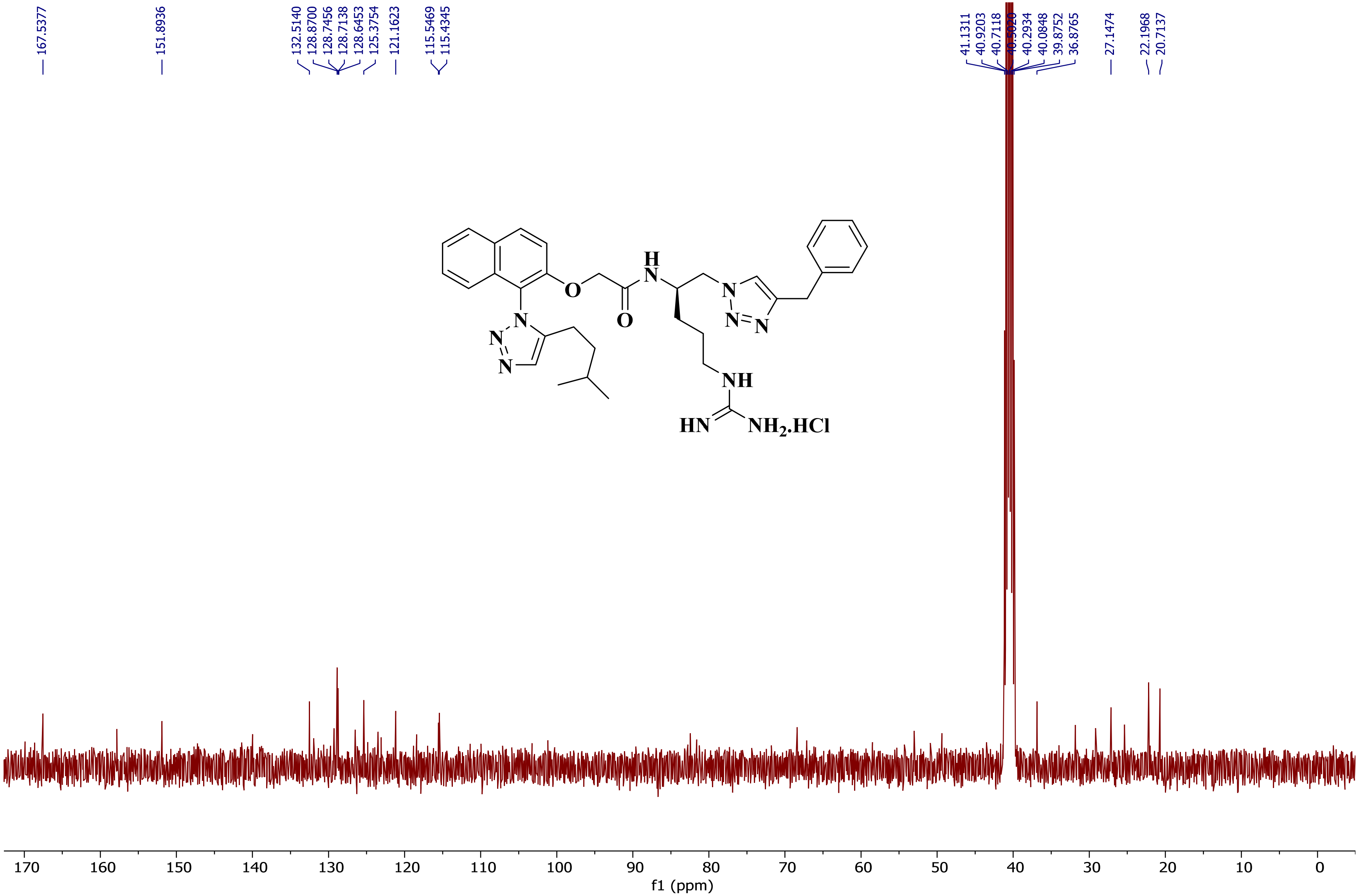

Figure S56: ^{13}C NMR of compound 26 (101 MHz, DMSO-d$_6$).

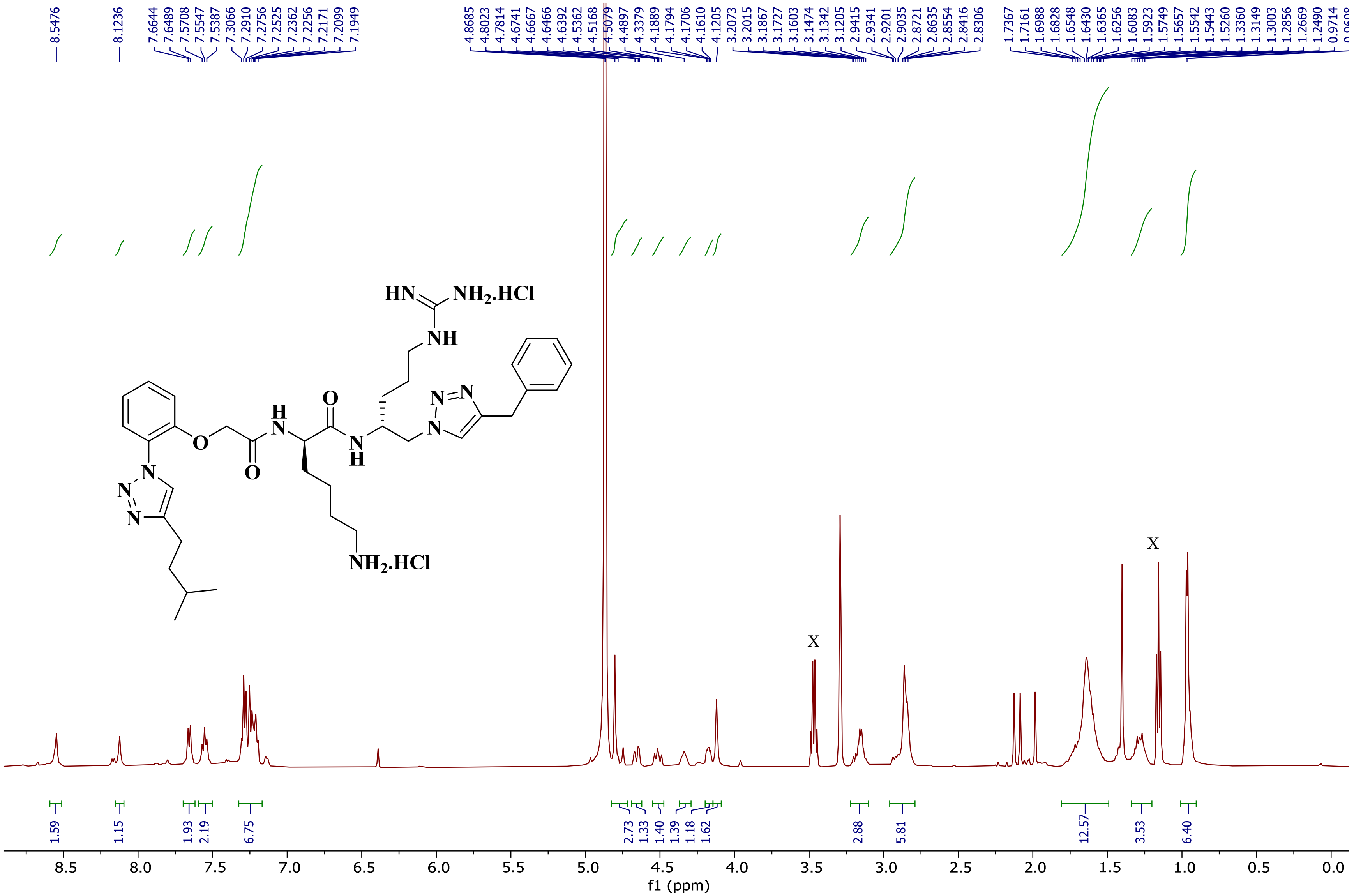

Figure S57: ^{1}H NMR of compound 36 (400 MHz, CD$_3$OD). X = diethyl ether solvent. Rotamers apparent in spectrum as evidenced by resonance broadening.

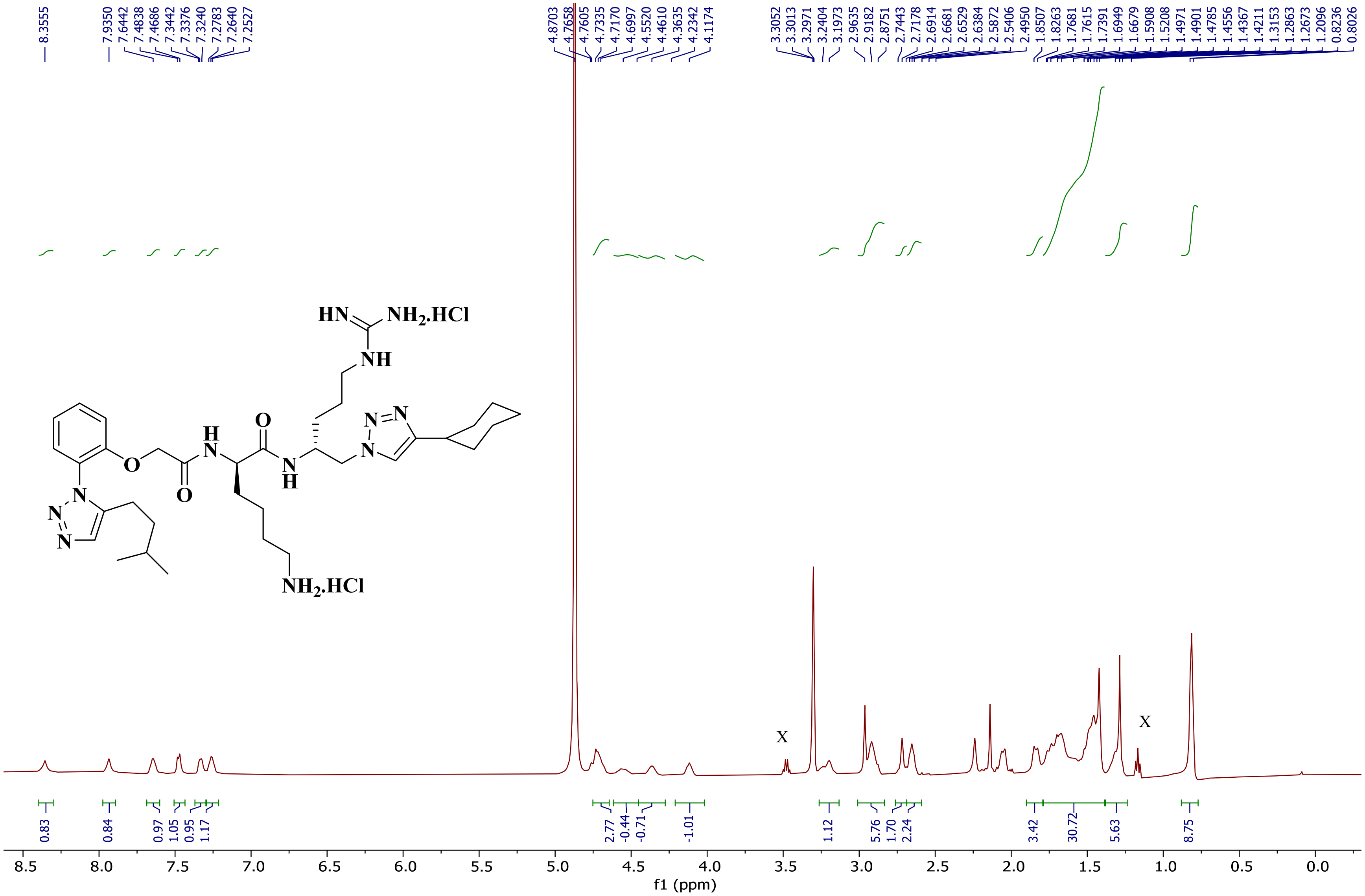

Figure S58: ^{1}H NMR of compound 37 (400 MHz, CD_3OD). X = diethyl ether solvent. Rotamers apparent in spectrum as evidenced by resonance broadening.

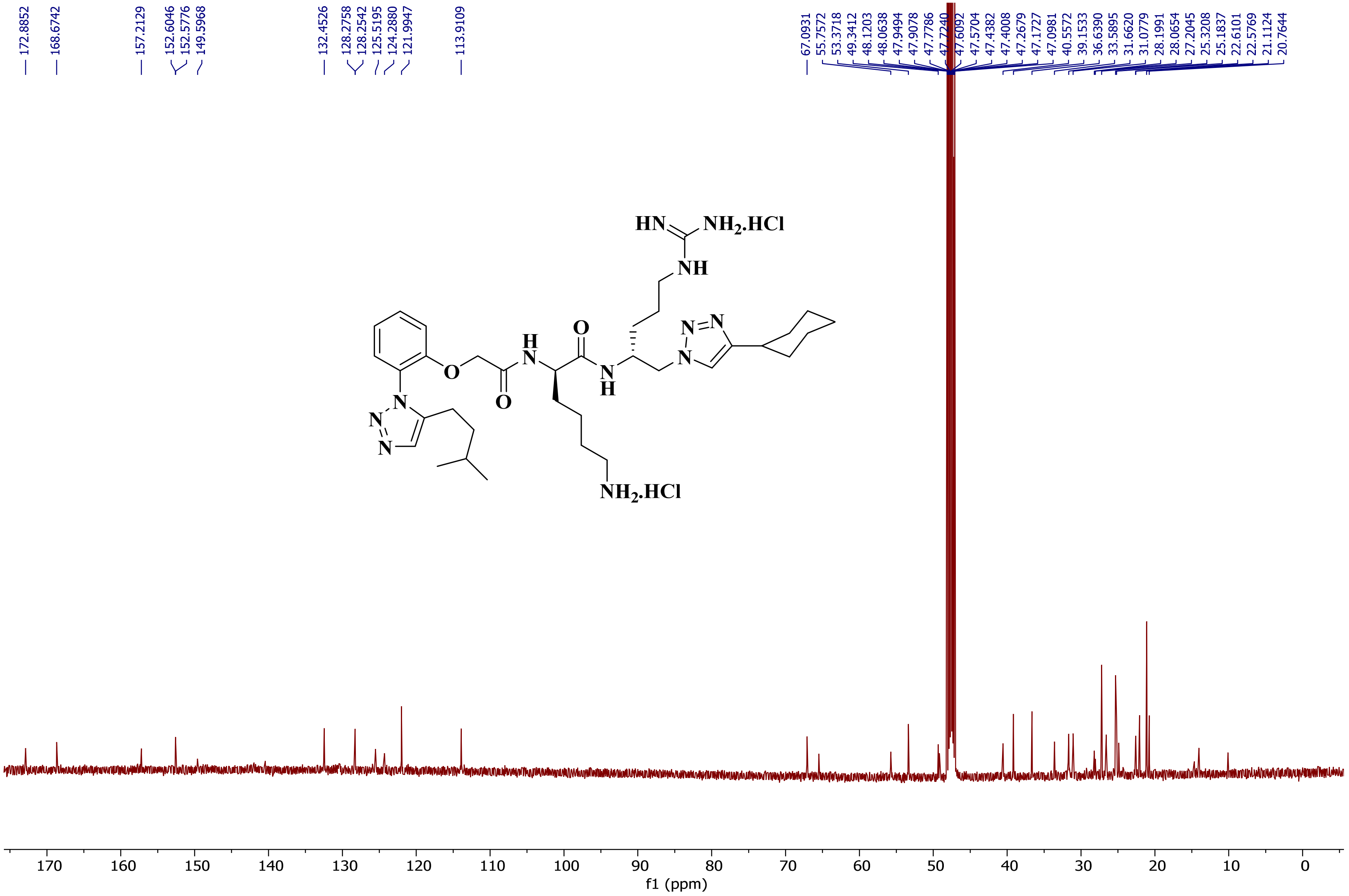

Figure S59: ^{13}C NMR of compound 37 (101 MHz, CD_3OD). Rotamers apparent in spectrum as evidenced by doubling of resonances.

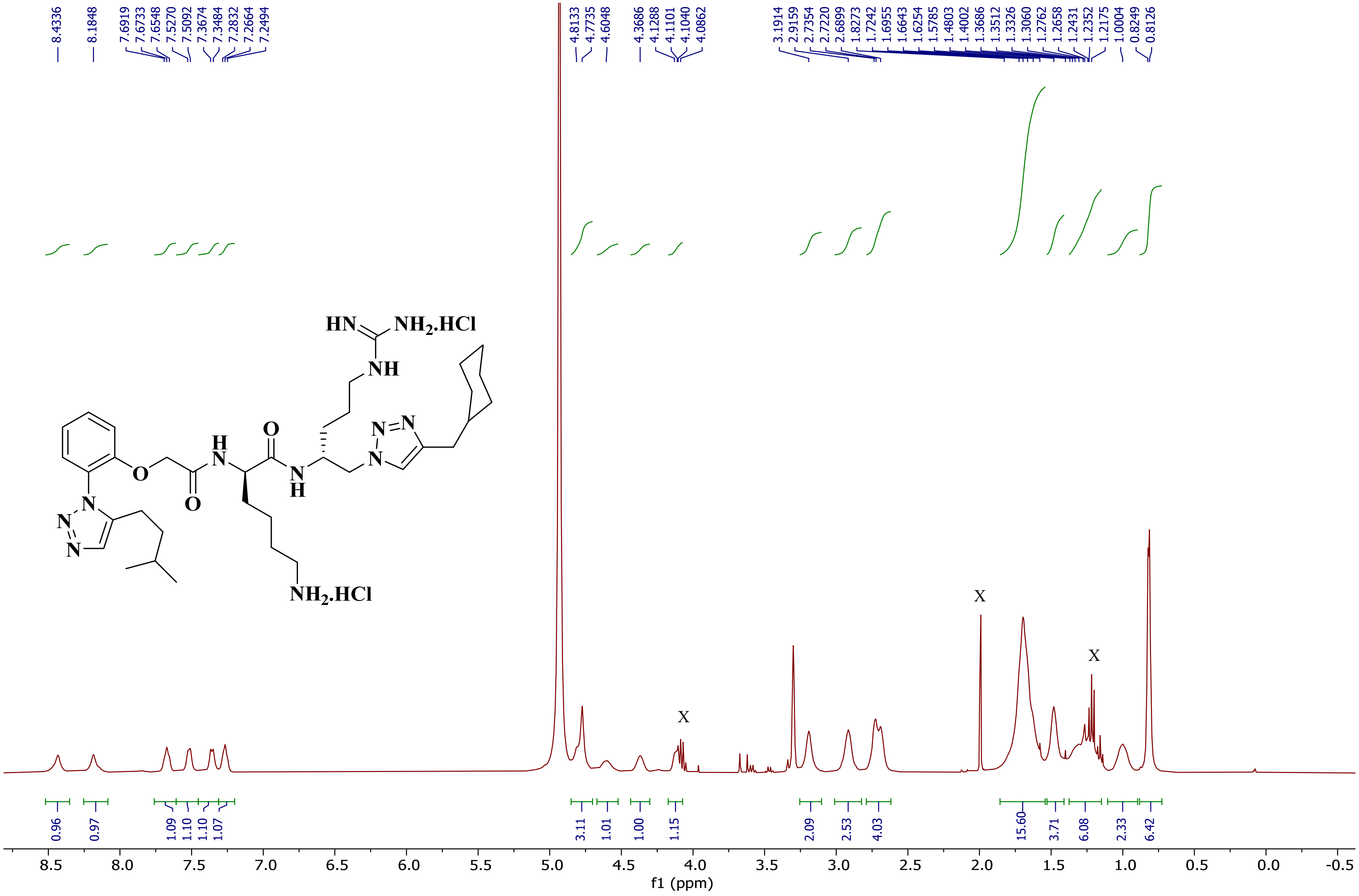

Figure S60: ^{1}H NMR of compound 38 (400 MHz, CD_3OD). X = ethyl acetate solvent. Rotamers apparent in spectrum as evidenced by resonance broadening.

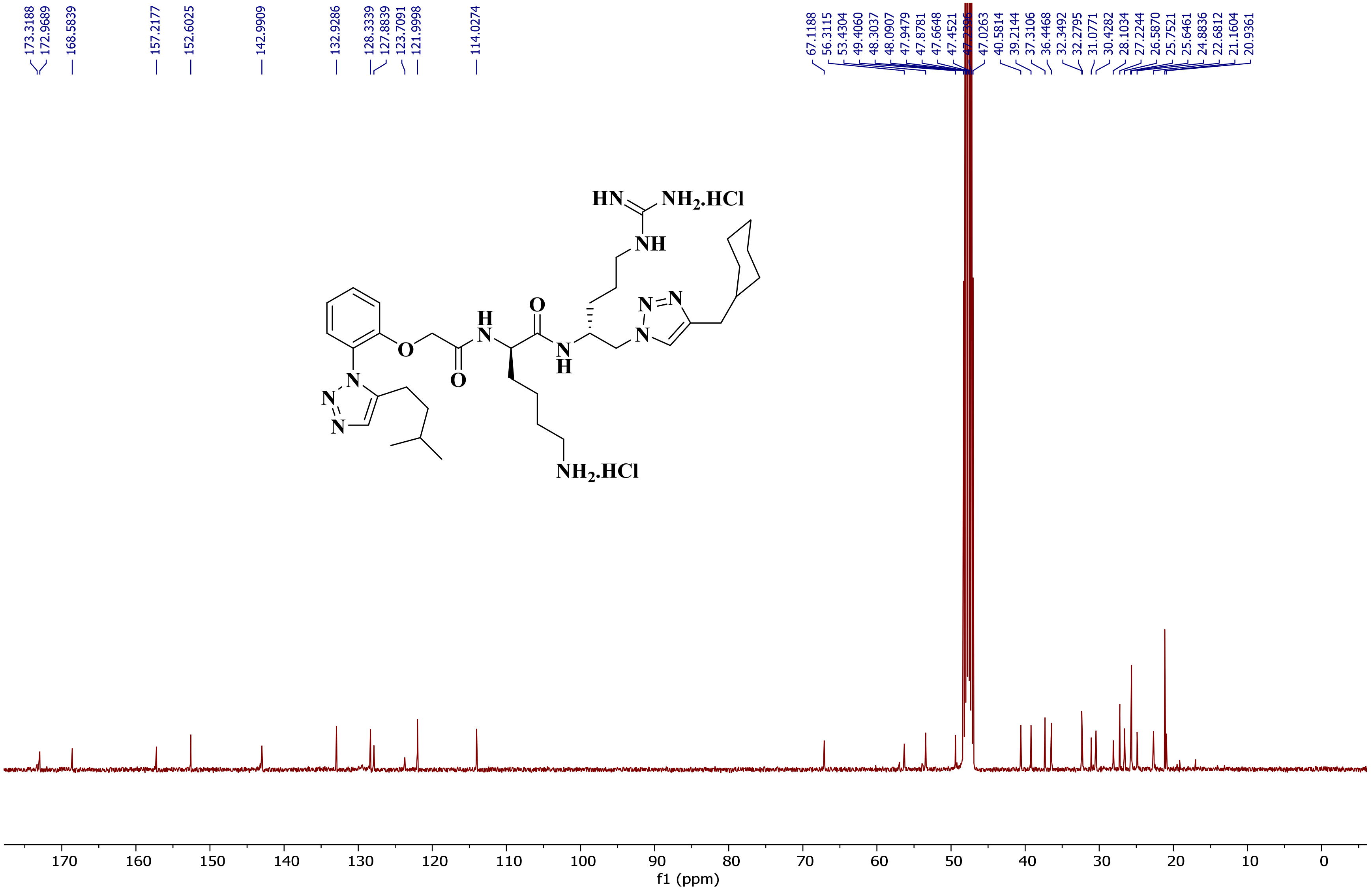

Figure S61: ^{13}C NMR of compound 38 (101 MHz, CD_3OD)

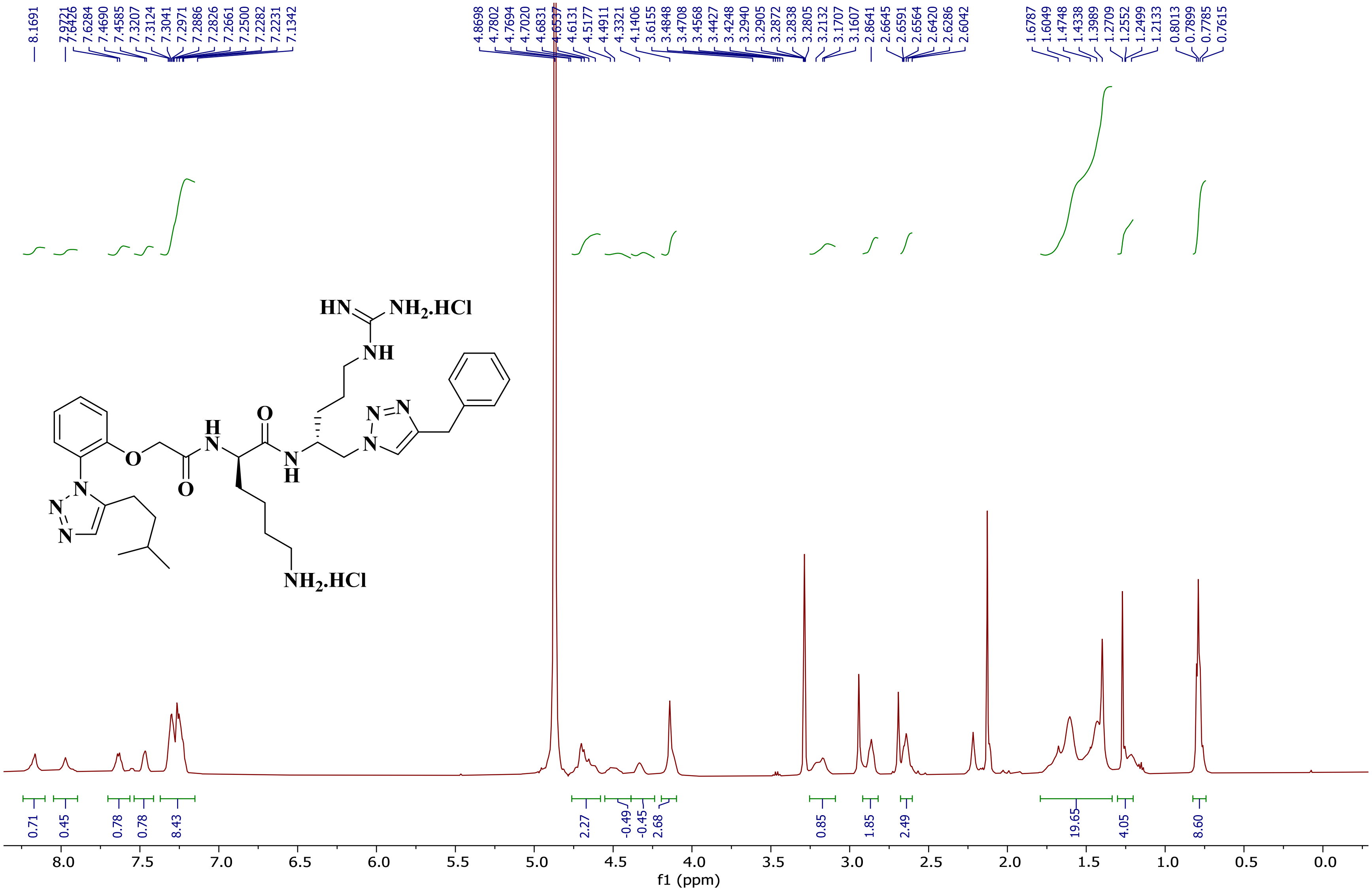

Figure S62: ^{1}H NMR of compound 39 (400 MHz, CD₃OD). Rotamers apparent in spectrum as evidenced by resonance broadening.

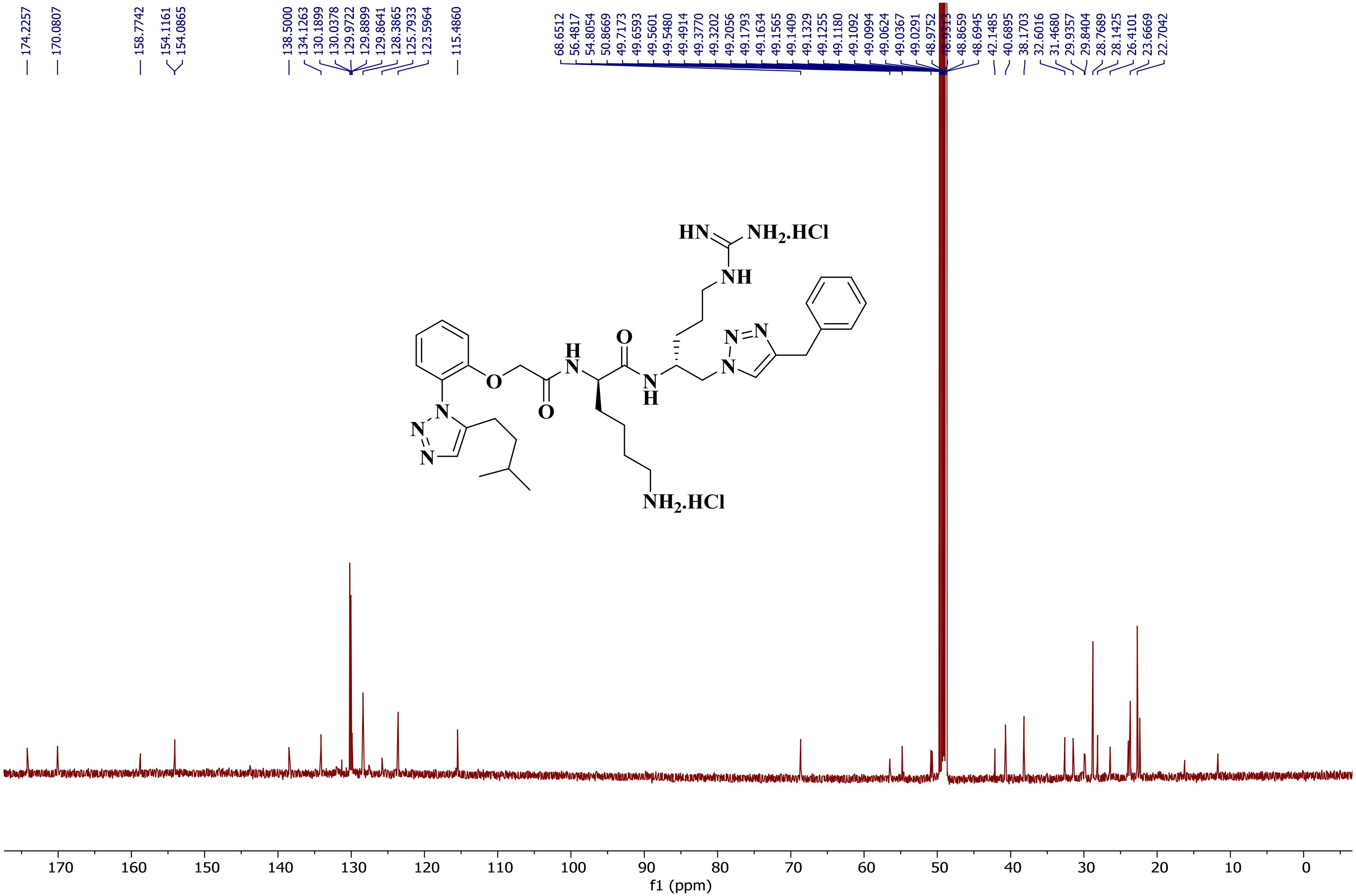

Figure S63: ^{13}C NMR of compound 39 (101 MHz, CD_3OD)

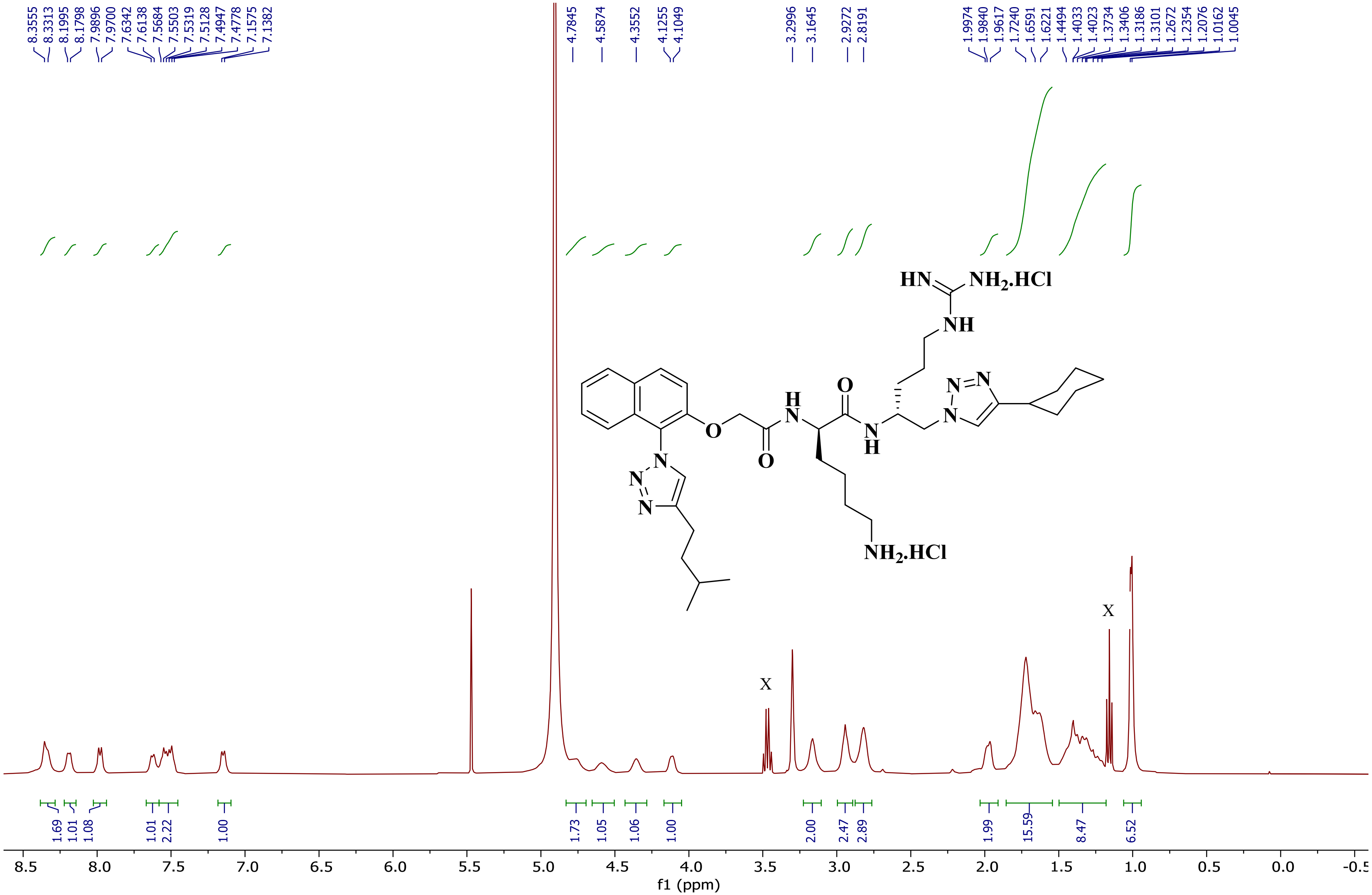

Figure S64: ^{1}H NMR of compound 40 (400 MHz, CD_3OD). X = diethyl ether solvent. Rotamers apparent in spectrum as evidenced by resonance broadening.

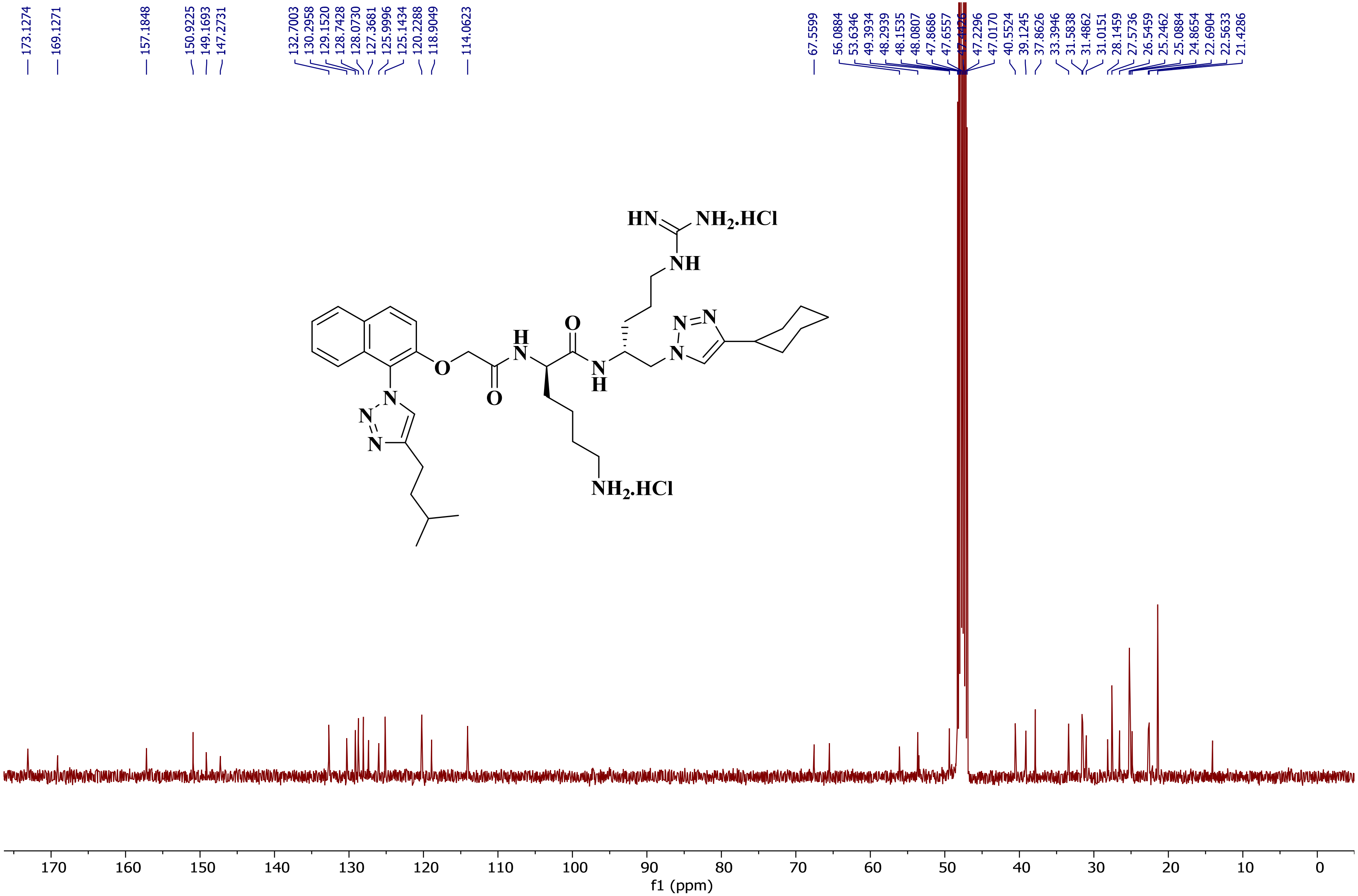

Figure S65: ^{13}C NMR of compound 40 (101 MHz, CD_3OD)

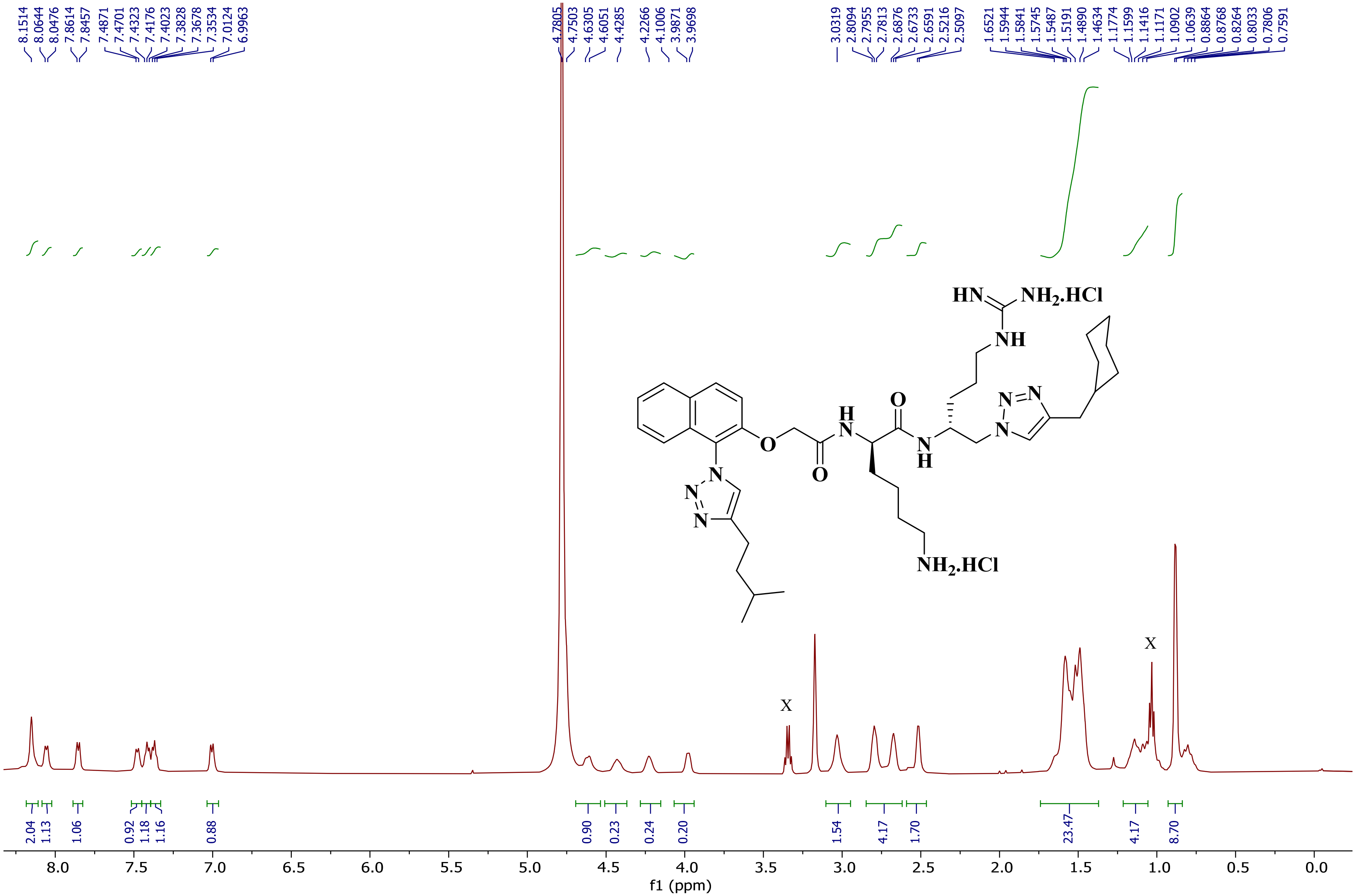

Figure S66: ^{1}H NMR of compound 41 (400 MHz, CD_3OD). X = diethyl ether solvent. Rotamers apparent in spectrum as evidenced by resonance broadening.

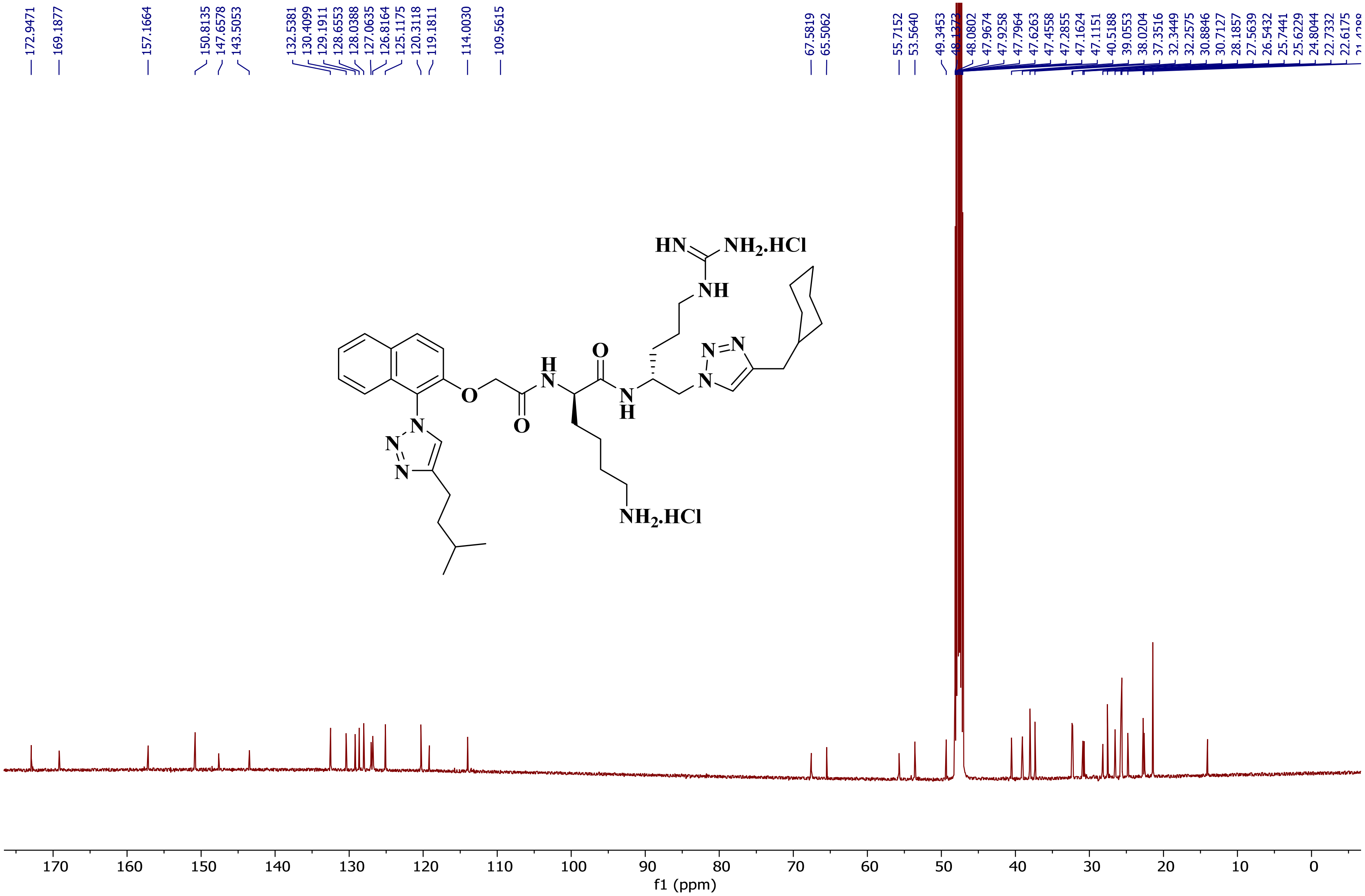

Figure S67: ^{13}C NMR of compound 41 (101 MHz, CD_3OD)

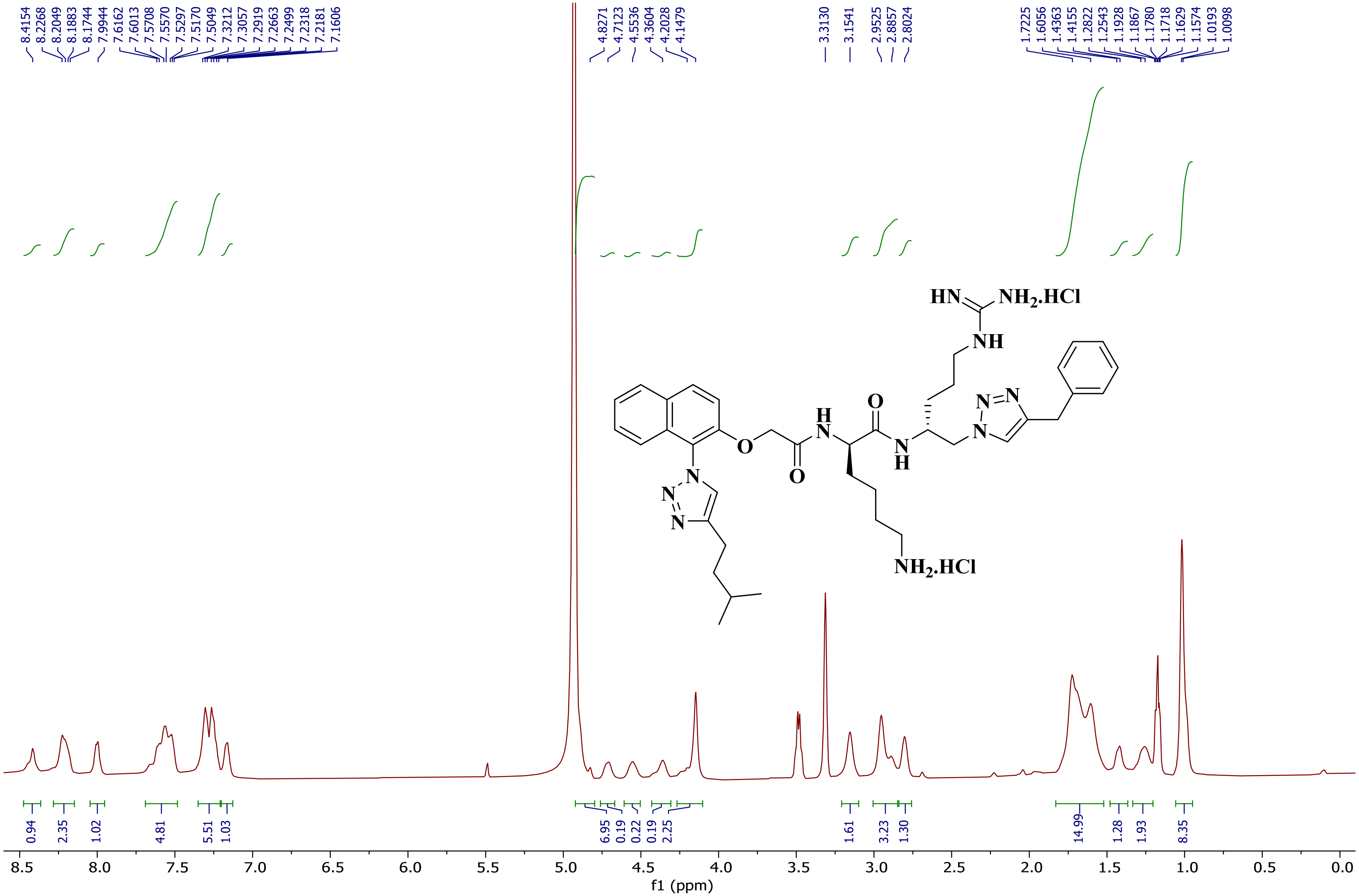

Figure S68: ^{1}H NMR of compound 42 (400 MHz, CD_3OD). Rotamers apparent in spectrum as evidenced by resonance broadening.

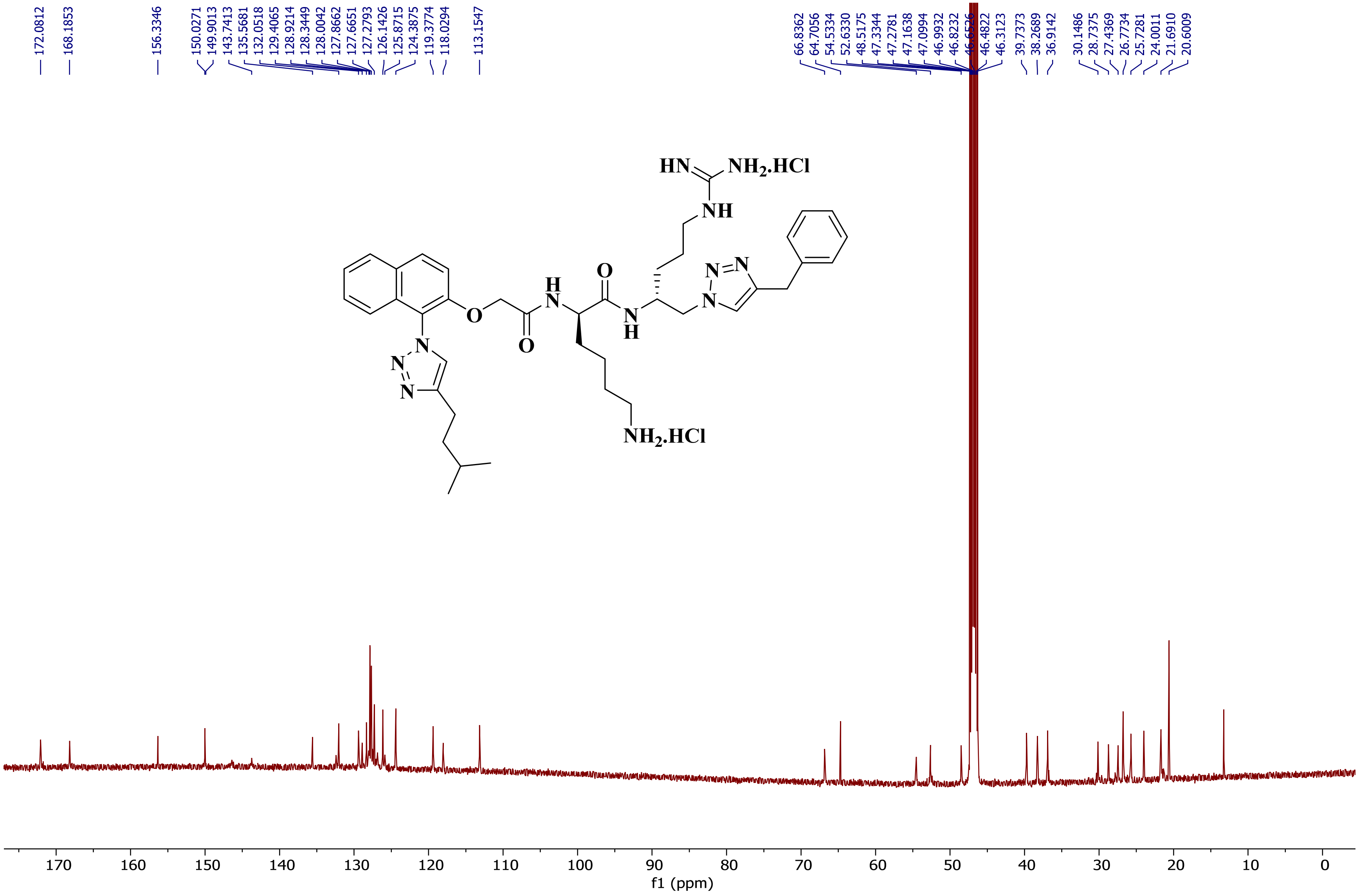

Figure S69: ^{13}C NMR of compound 42 (101 MHz, CD_3OD)

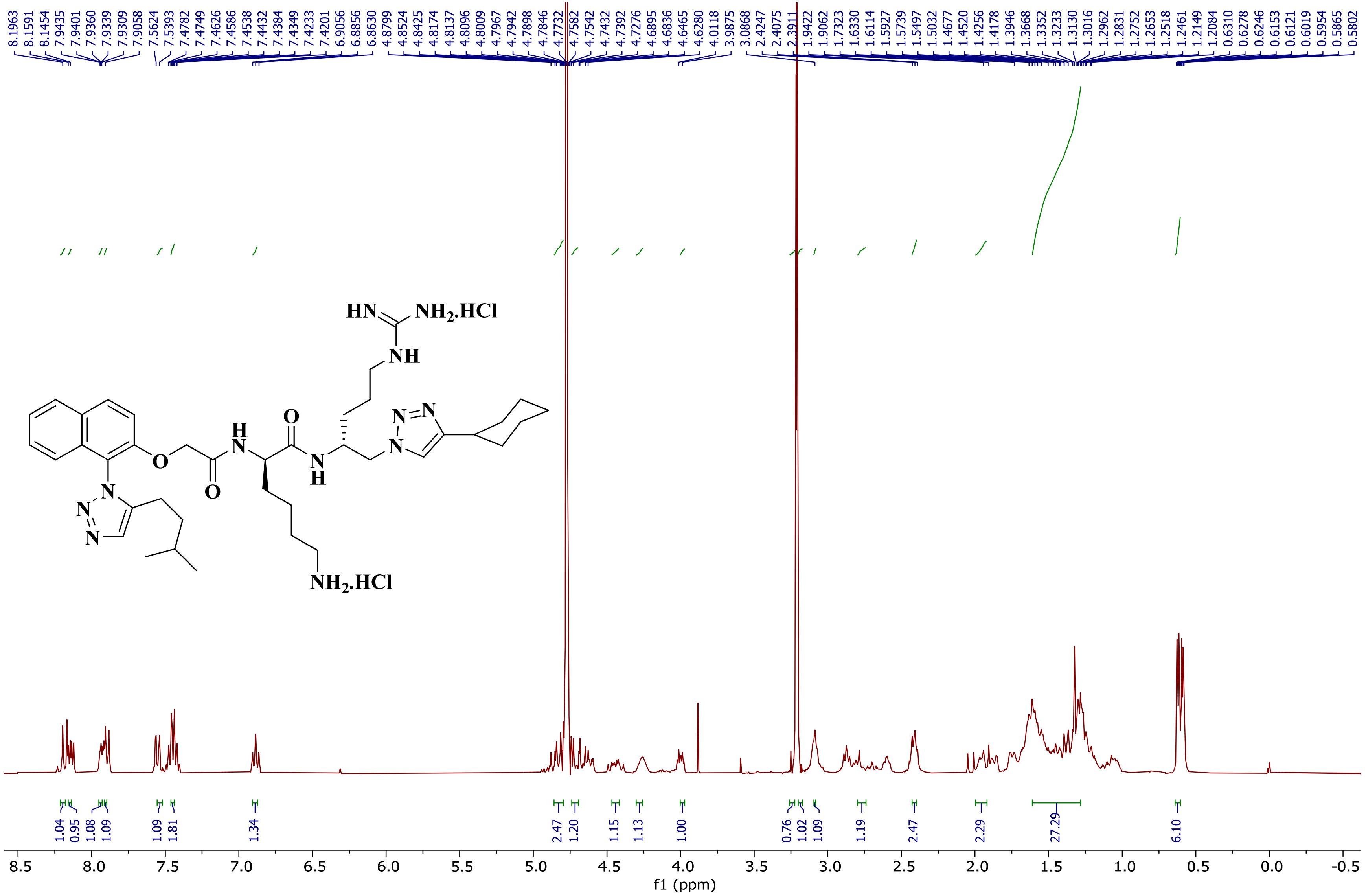

Figure S70: ^{1}H NMR of compound 43 (400 MHz, CD_3OD). Rotamers apparent in spectrum as evidenced by resonance broadening.

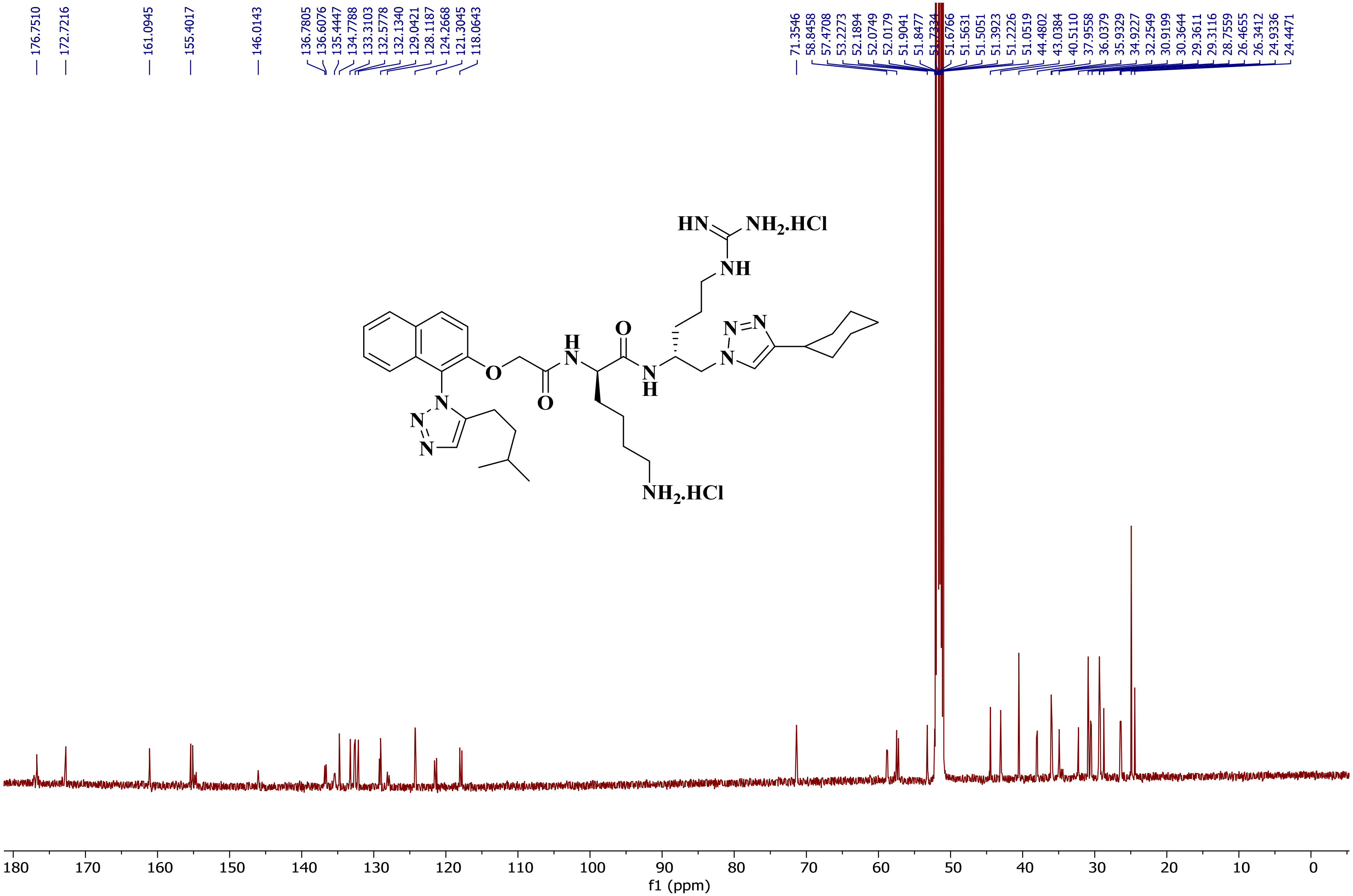

Figure S71: ^{13}C NMR of compound 43 (101 MHz, CD_3OD). Rotamers apparent in spectrum as evidenced by doubling of resonances.

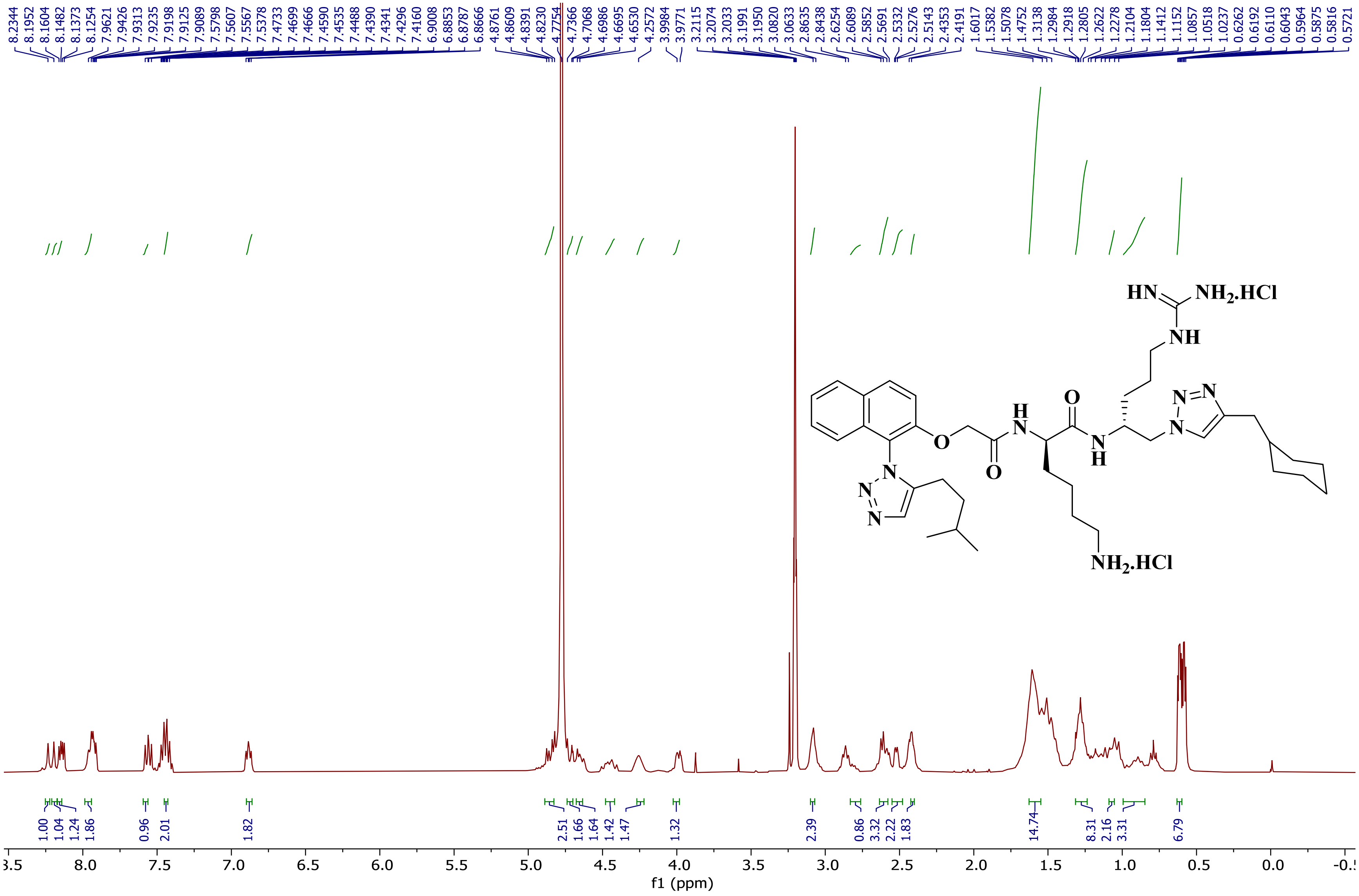

Figure S72: ^{1}H NMR of compound 44 (400 MHz, CD_3OD). Rotamers apparent in spectrum as evidenced by resonance broadening.

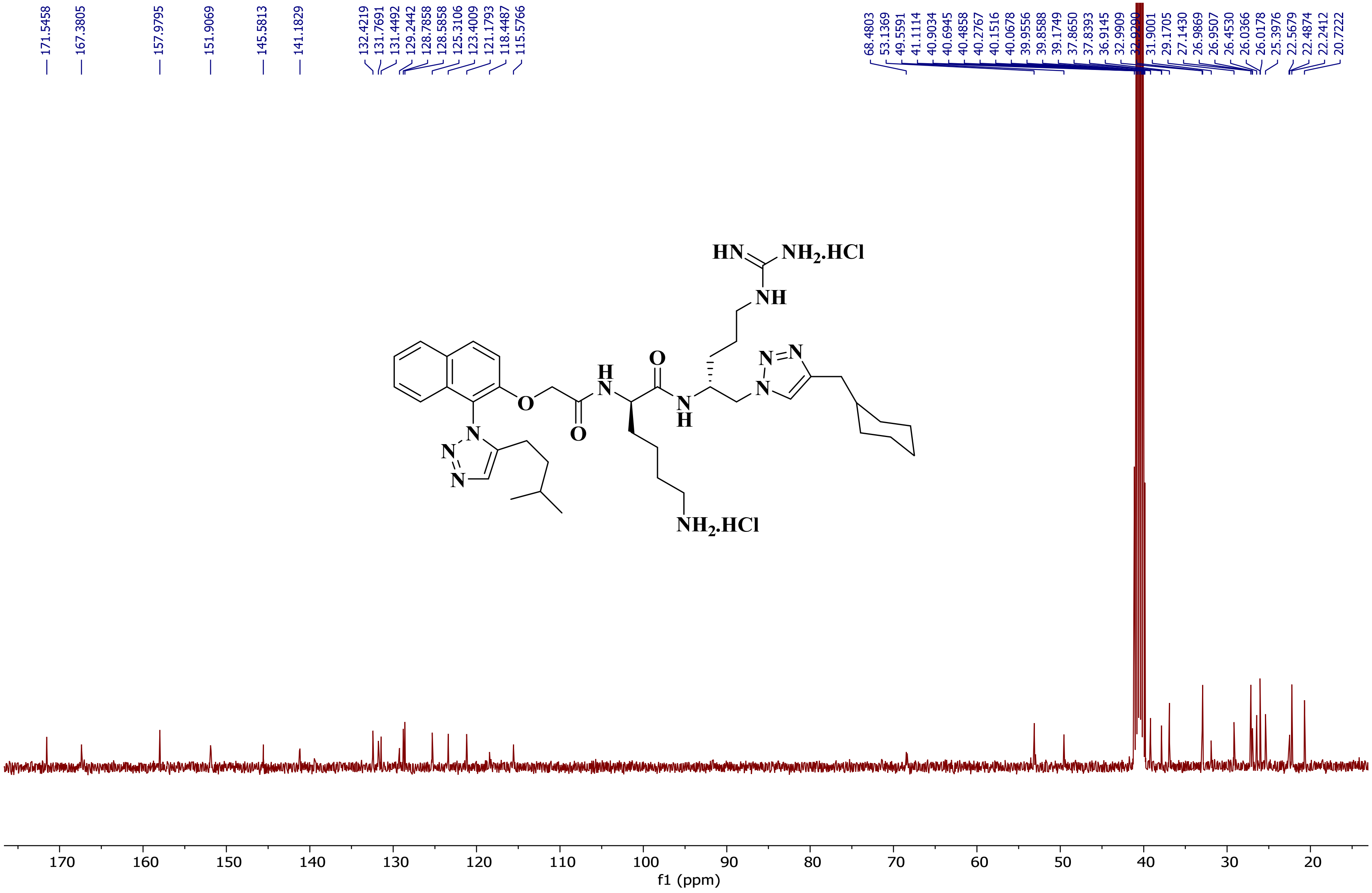

Figure S73: ^{13}C NMR of compound 44 (101 MHz, DMSO-d$_6$)

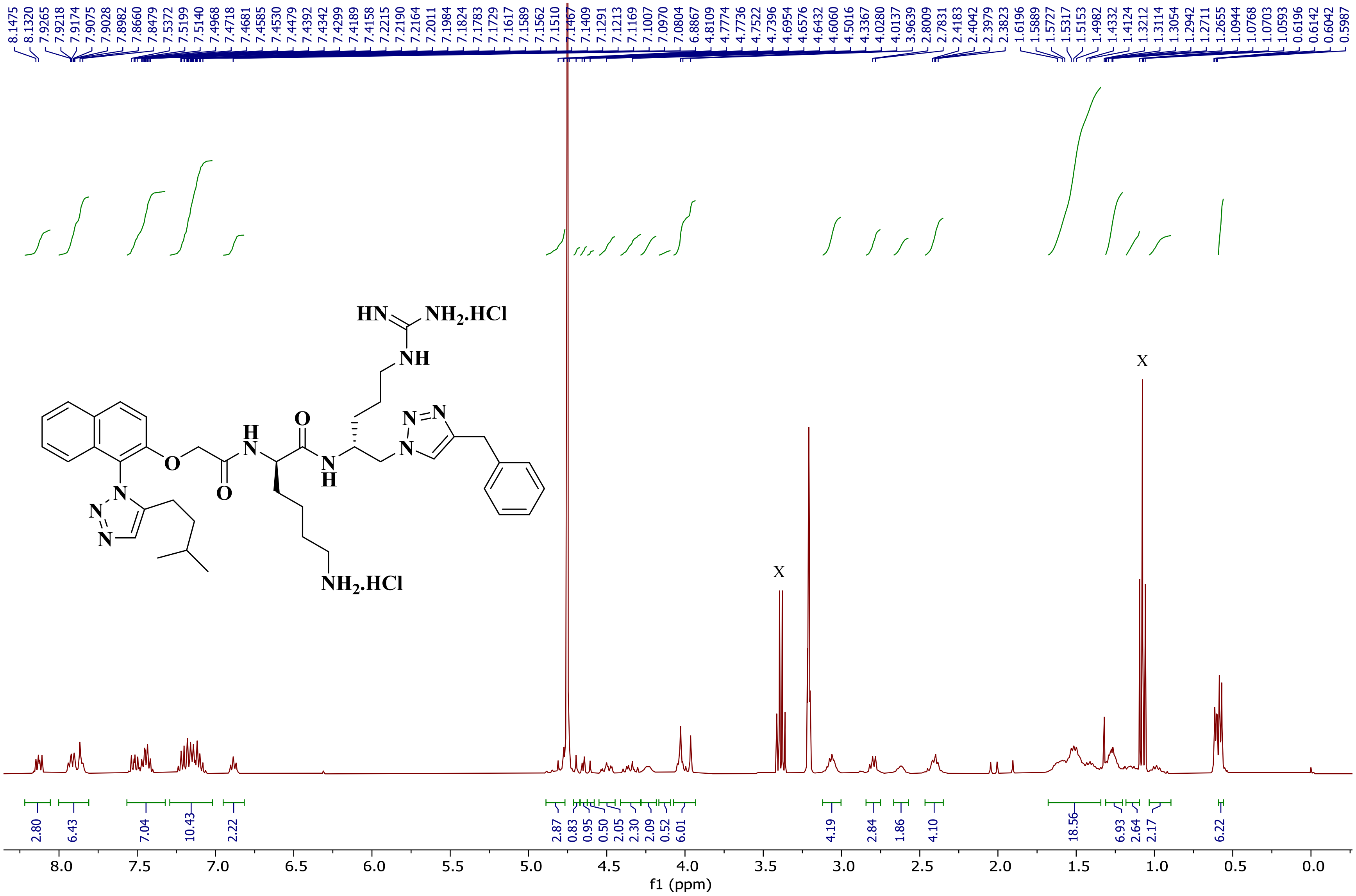

Figure S74: ^{1}H NMR of compound 45 (400 MHz, CD_3OD). X = diethyl ether solvent. Rotamers apparent in spectrum as evidenced by resonance broadening.

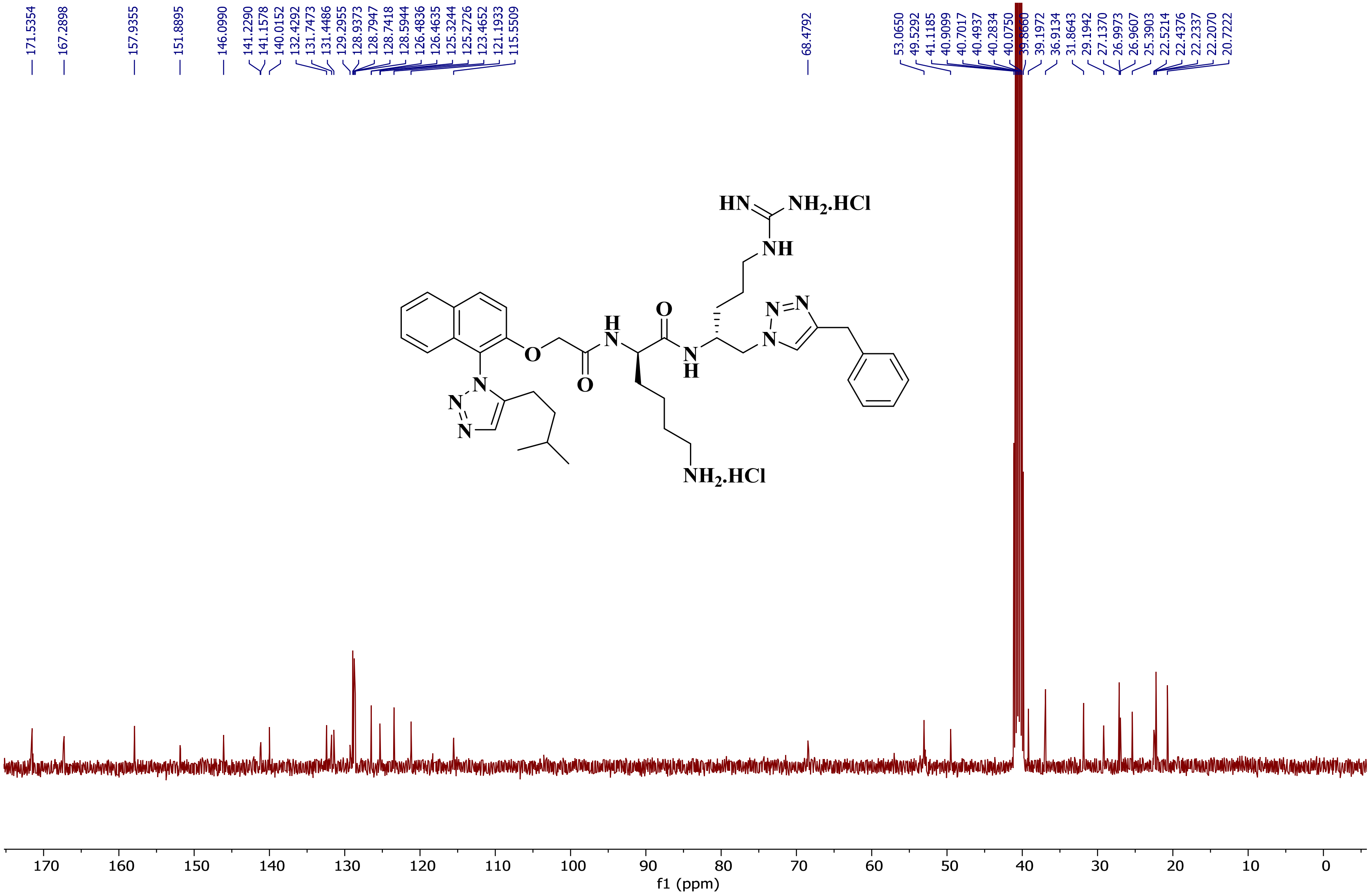

Figure S75: ^{13}C NMR of compound 45 (101 MHz, CD_3OD) Rotamers apparent in spectrum as evidenced by doubling of resonances.

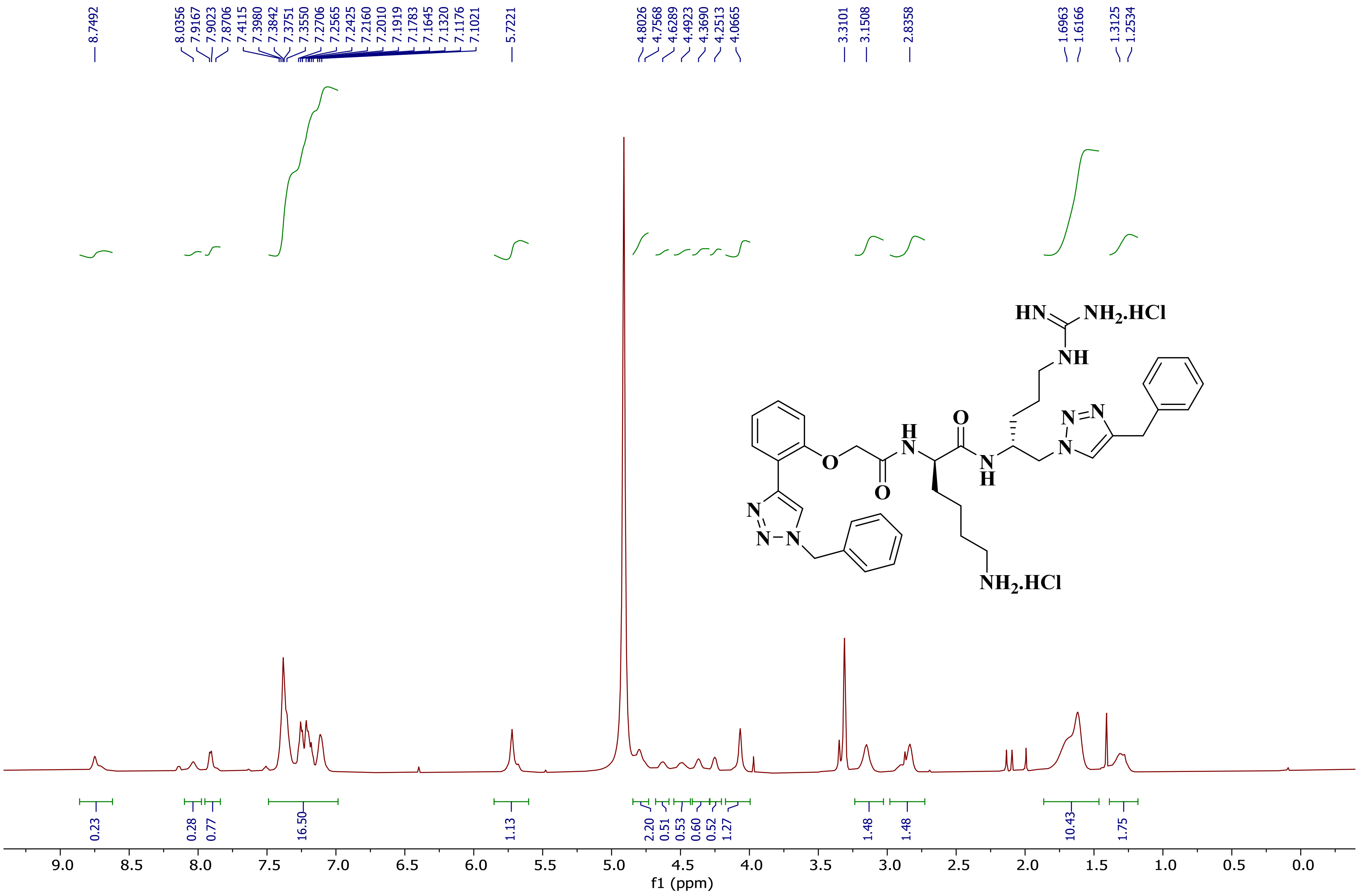

Figure S76: ^{1}H NMR of compound 46 (400 MHz, CD_3OD). Rotamers apparent in spectrum as evidenced by resonance broadening.

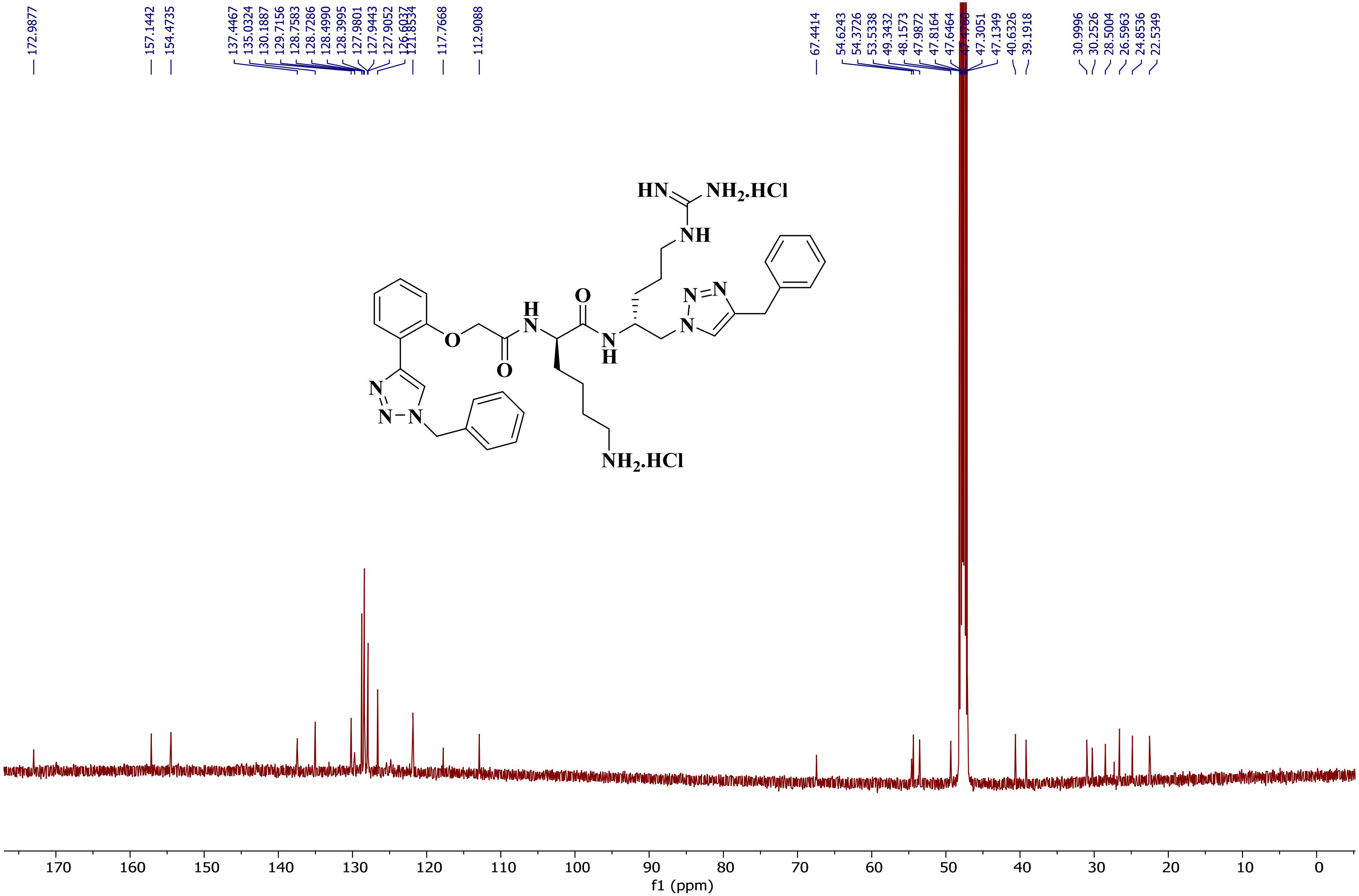

Figure S77: ^{13}C NMR of compound 46 (101 MHz, CD_3OD)

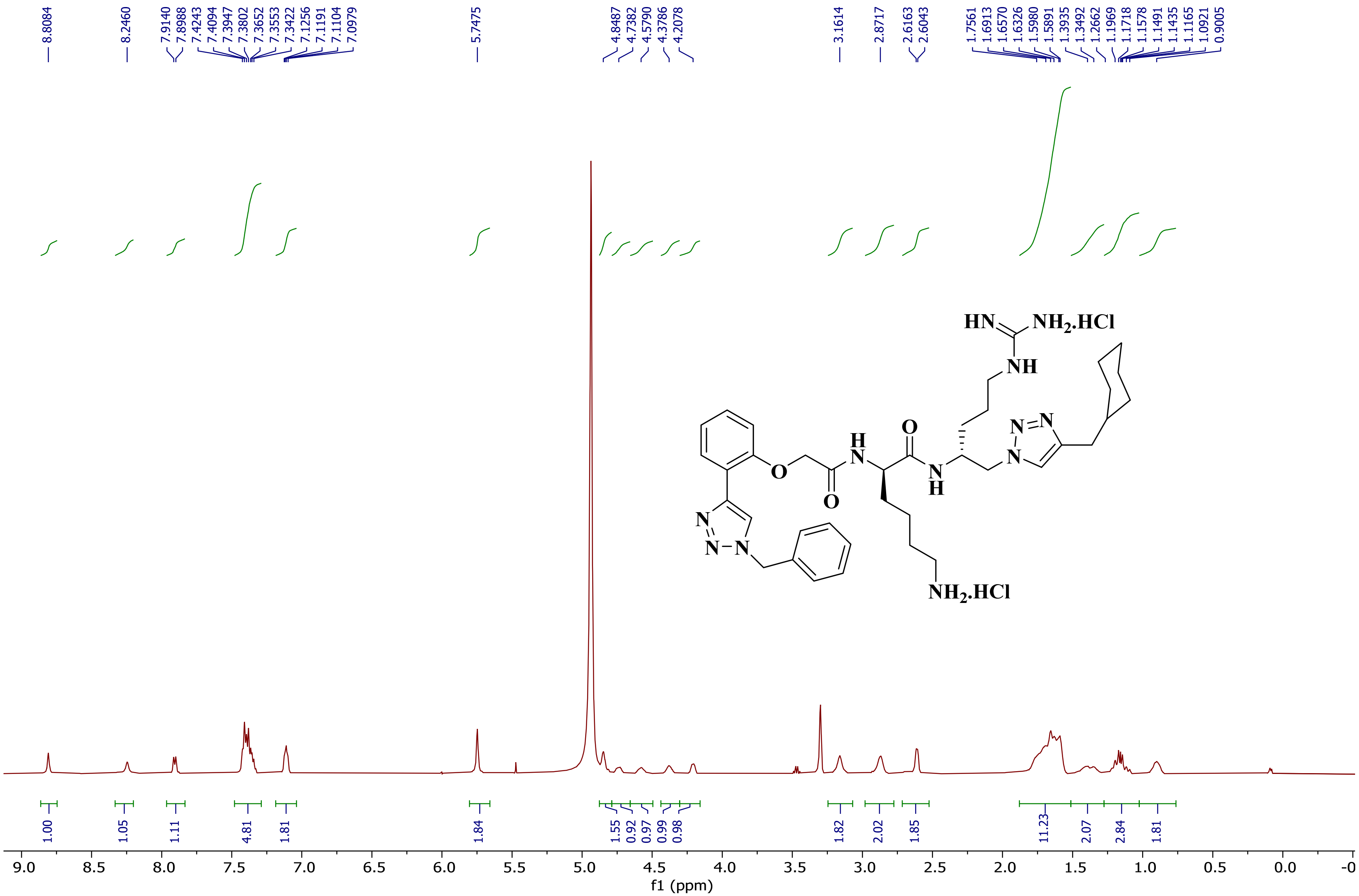

Figure S78: ^{1}H NMR of compound 47 (400 MHz, CD_3OD). Rotamers apparent in spectrum as evidenced by resonance broadening.

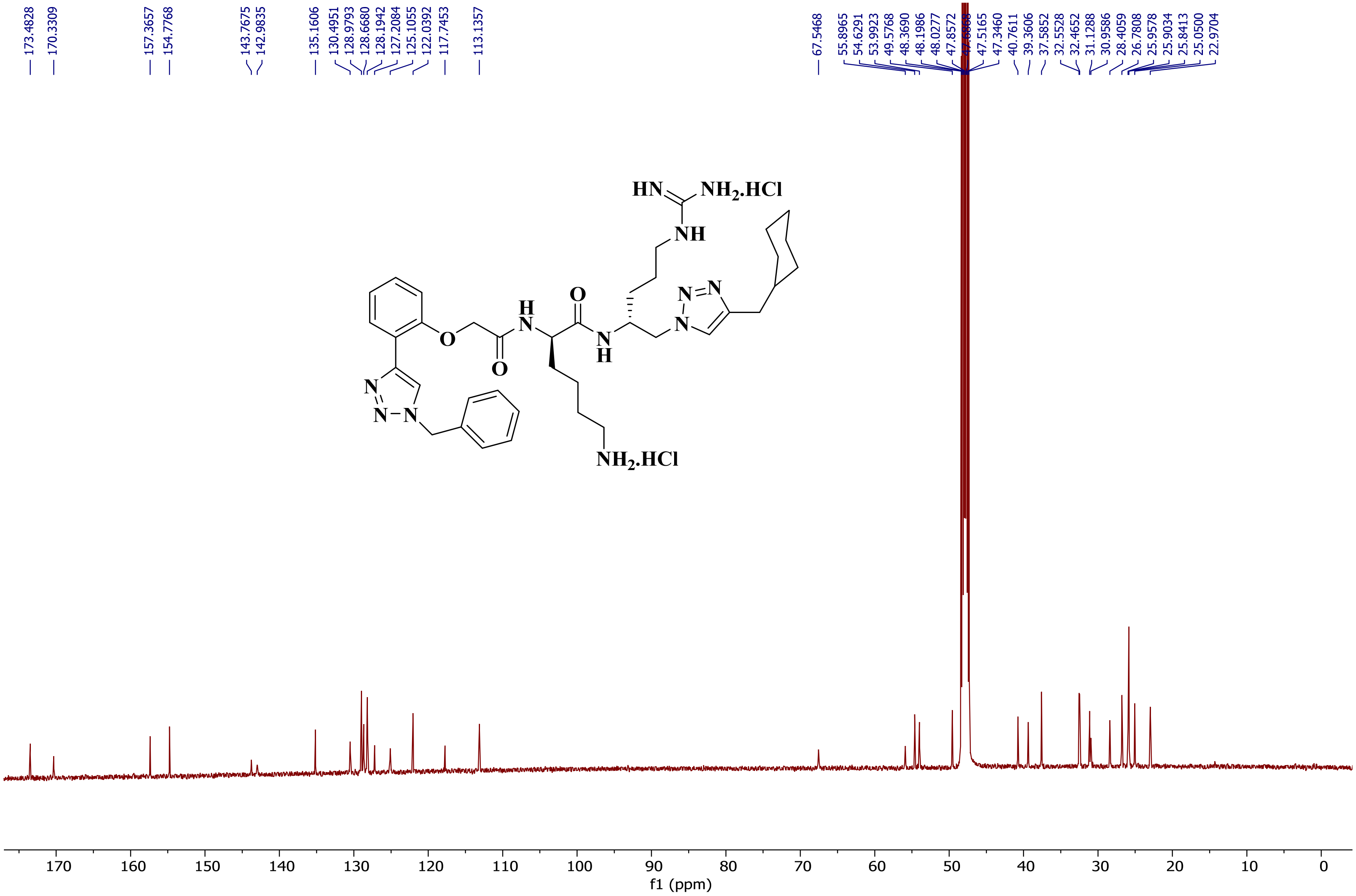

Figure S79: ^{13}C NMR of compound 47 (101 MHz, CD_3OD)

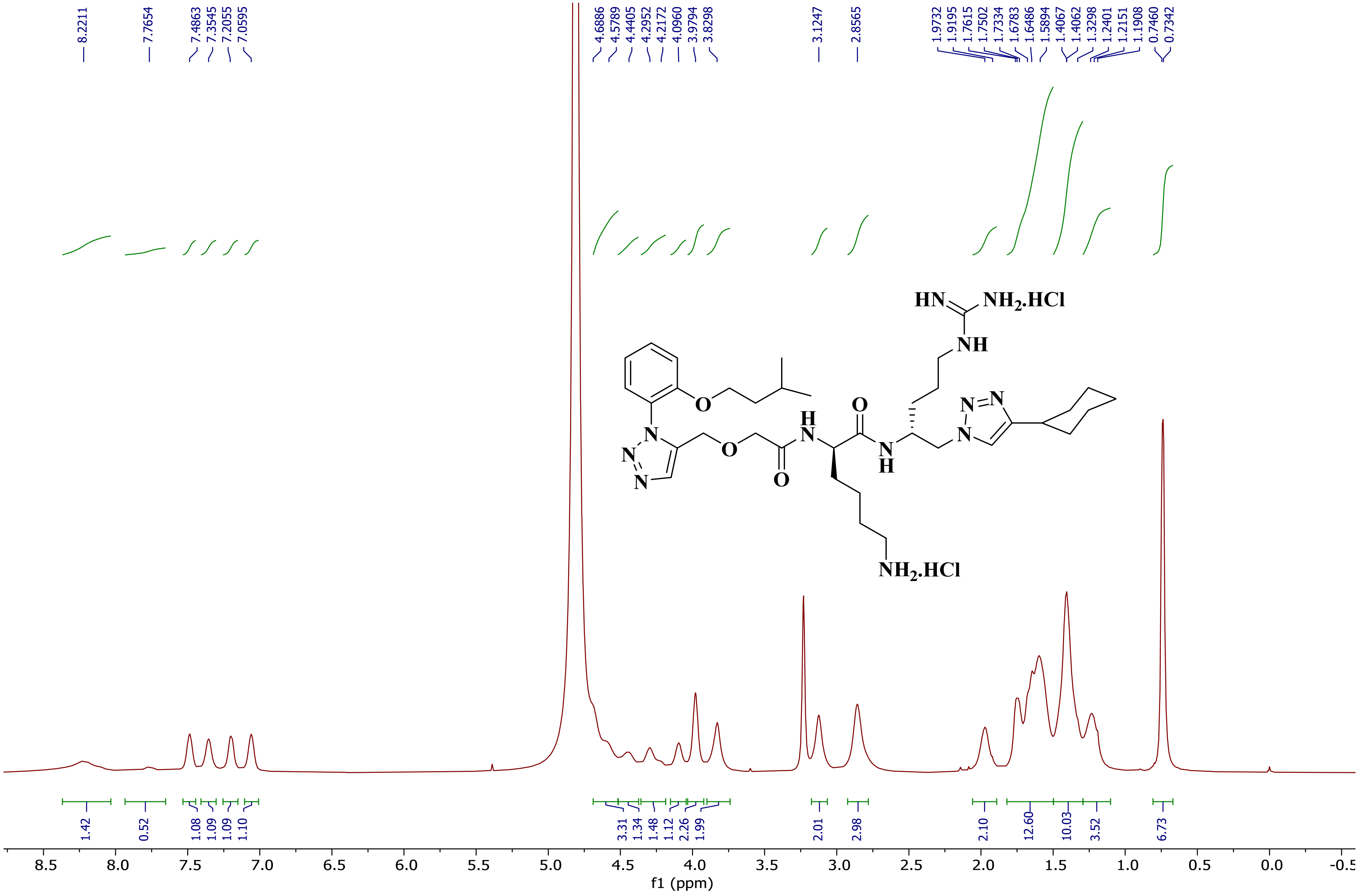

Figure S80: ^{1}H NMR of compound 48 (400 MHz, CD_3OD). Rotamers apparent in spectrum as evidenced by resonance broadening.

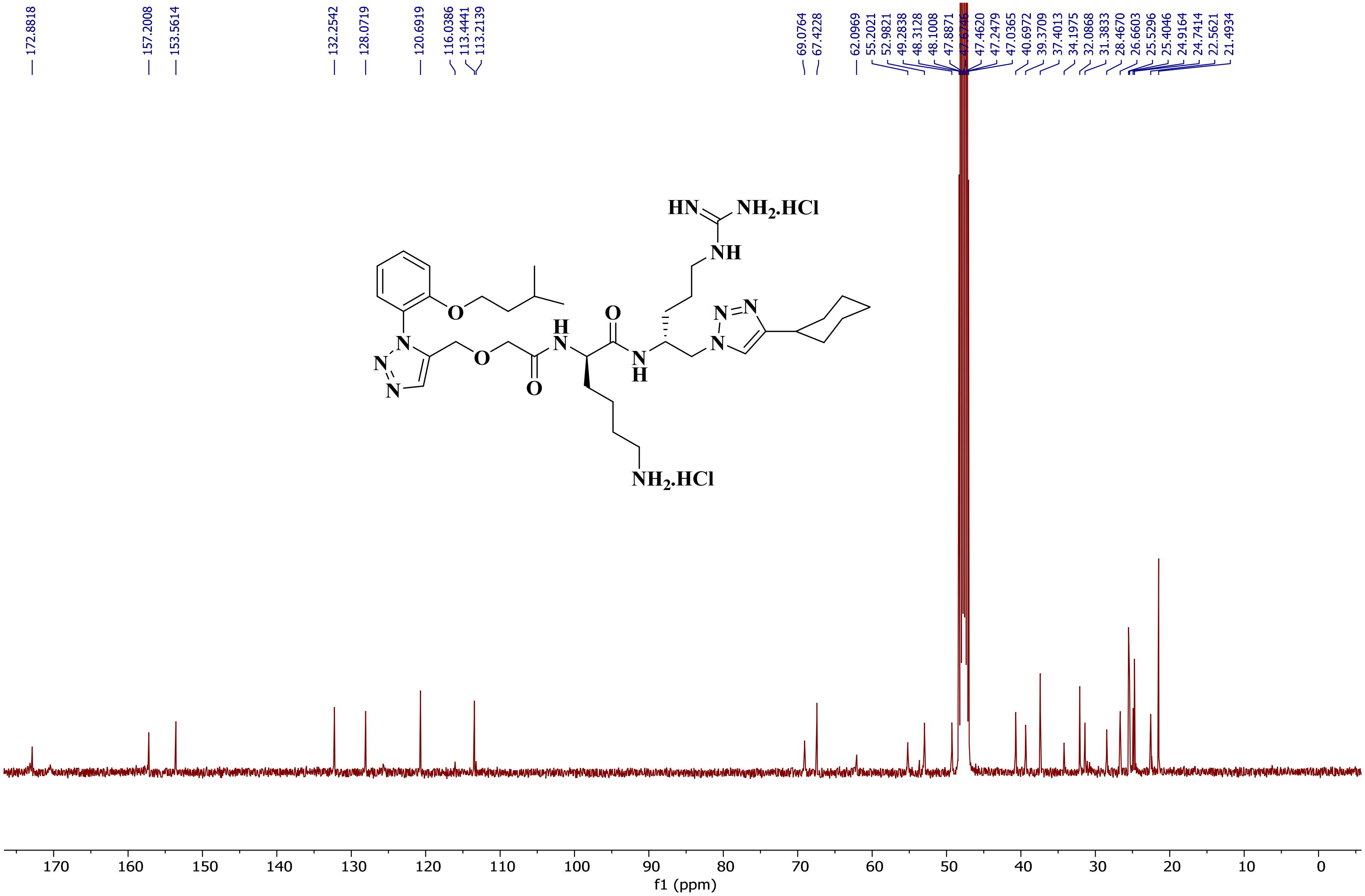

Figure S81: ^{13}C NMR of compound 48 (101 MHz, CD_3OD)

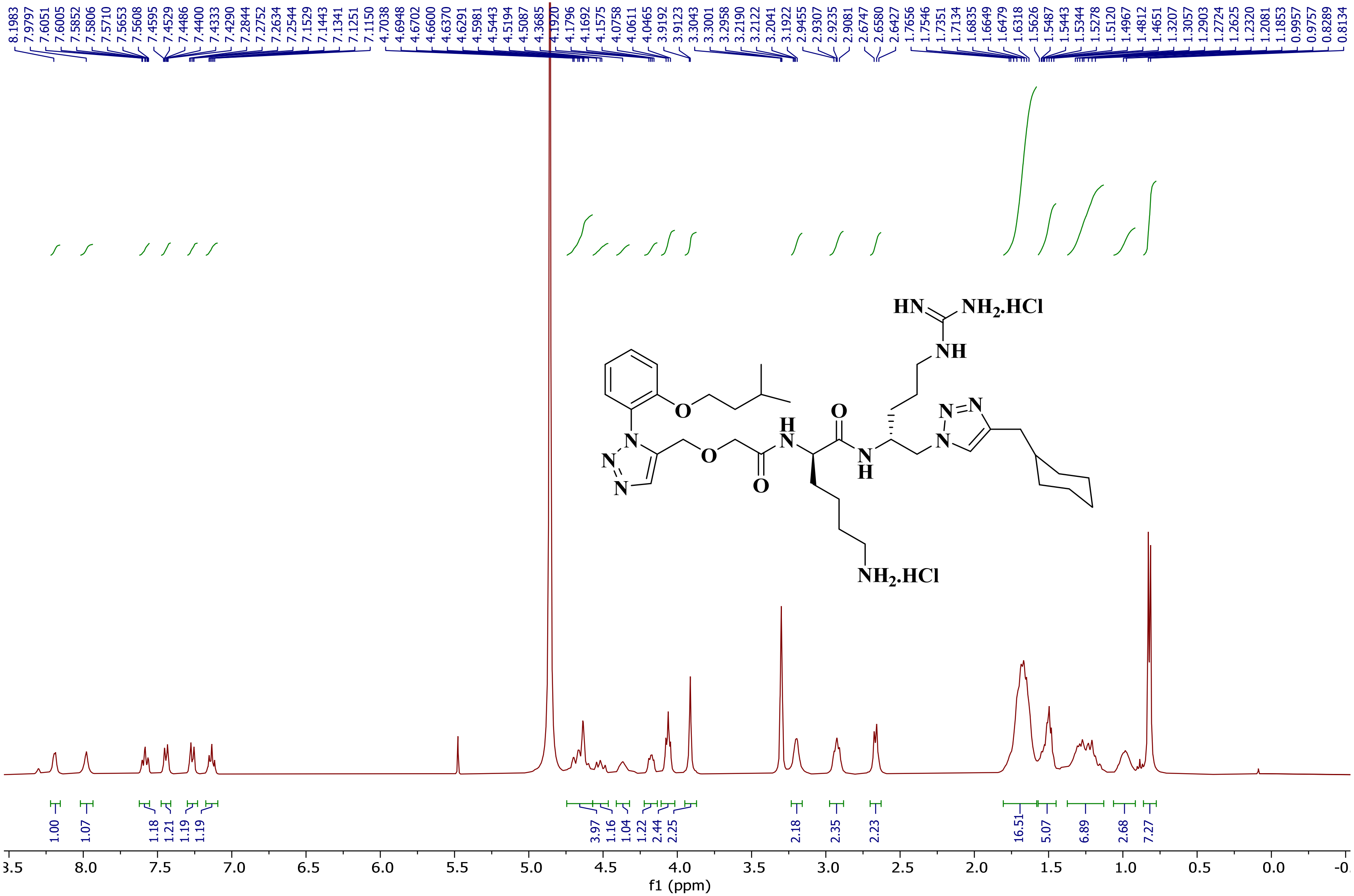

Figure S82: ^{1}H NMR of compound 49 (400 MHz, CD_3OD). Rotamers apparent in spectrum as evidenced by resonance broadening.

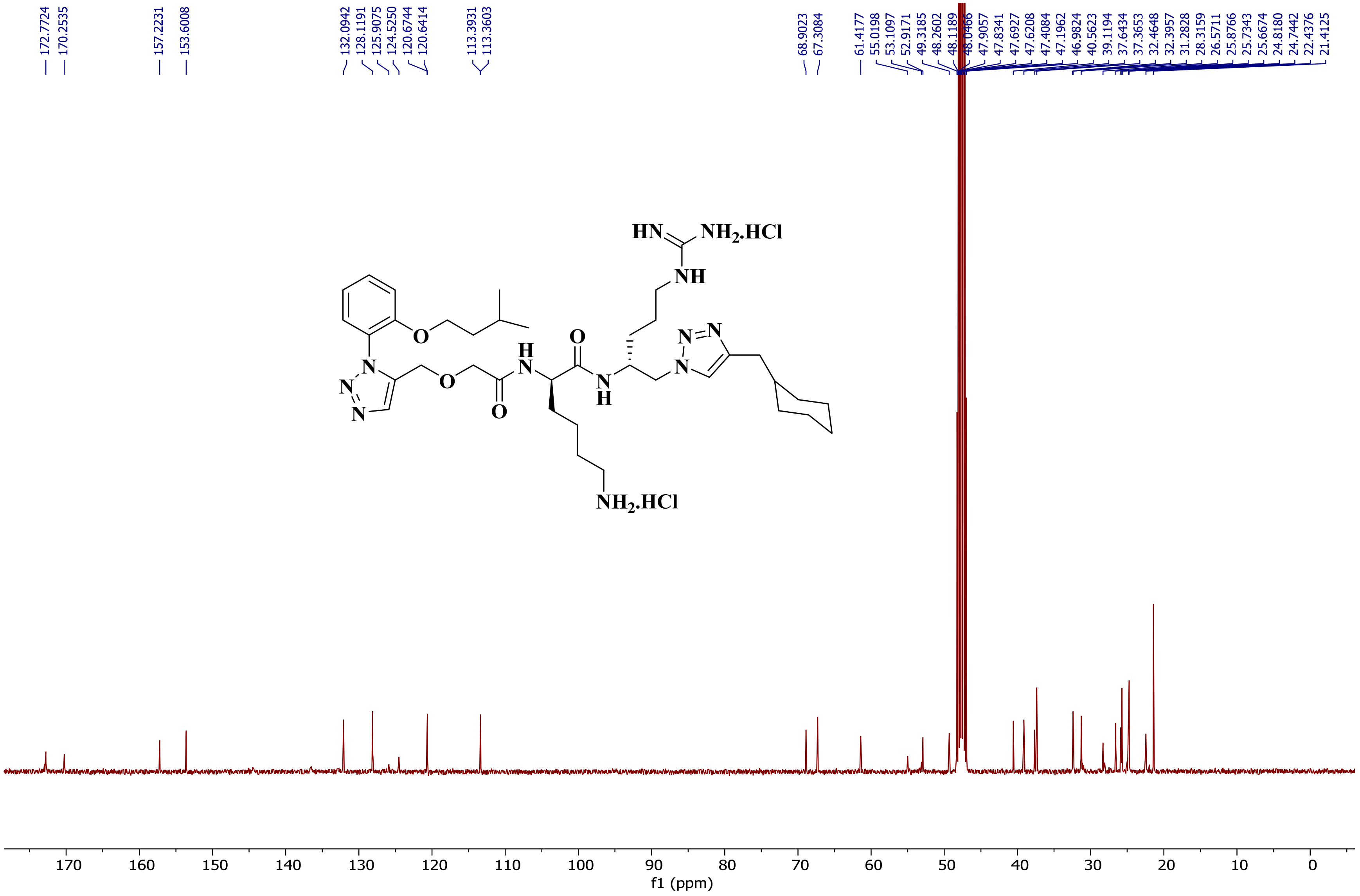

Figure S83: ^{13}C NMR of compound 49 (101 MHz, CD_3OD)

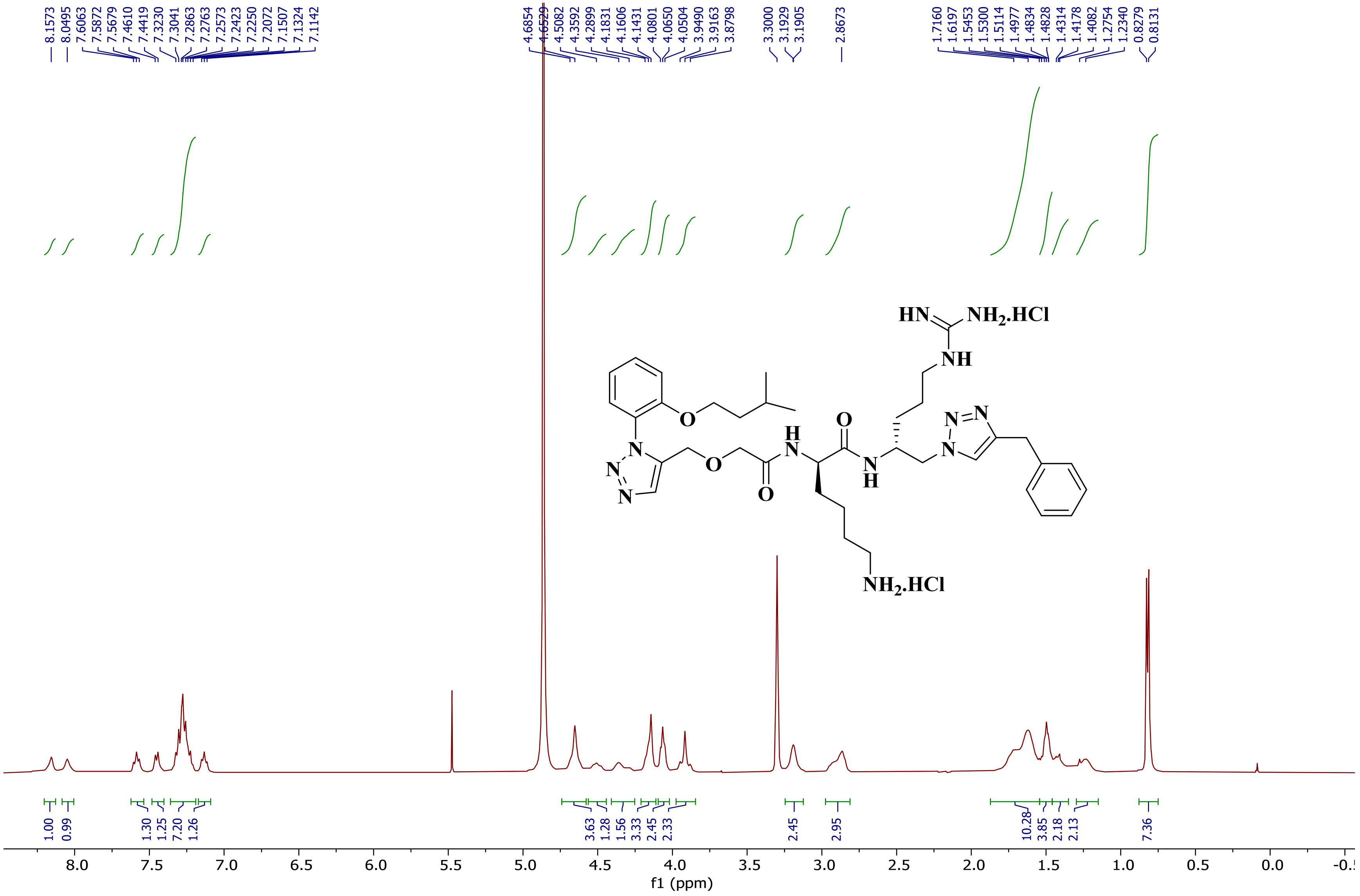

Figure S84: ^{1}H NMR of compound 50 (400 MHz, CD_3OD). Rotamers apparent in spectrum as evidenced by resonance broadening.

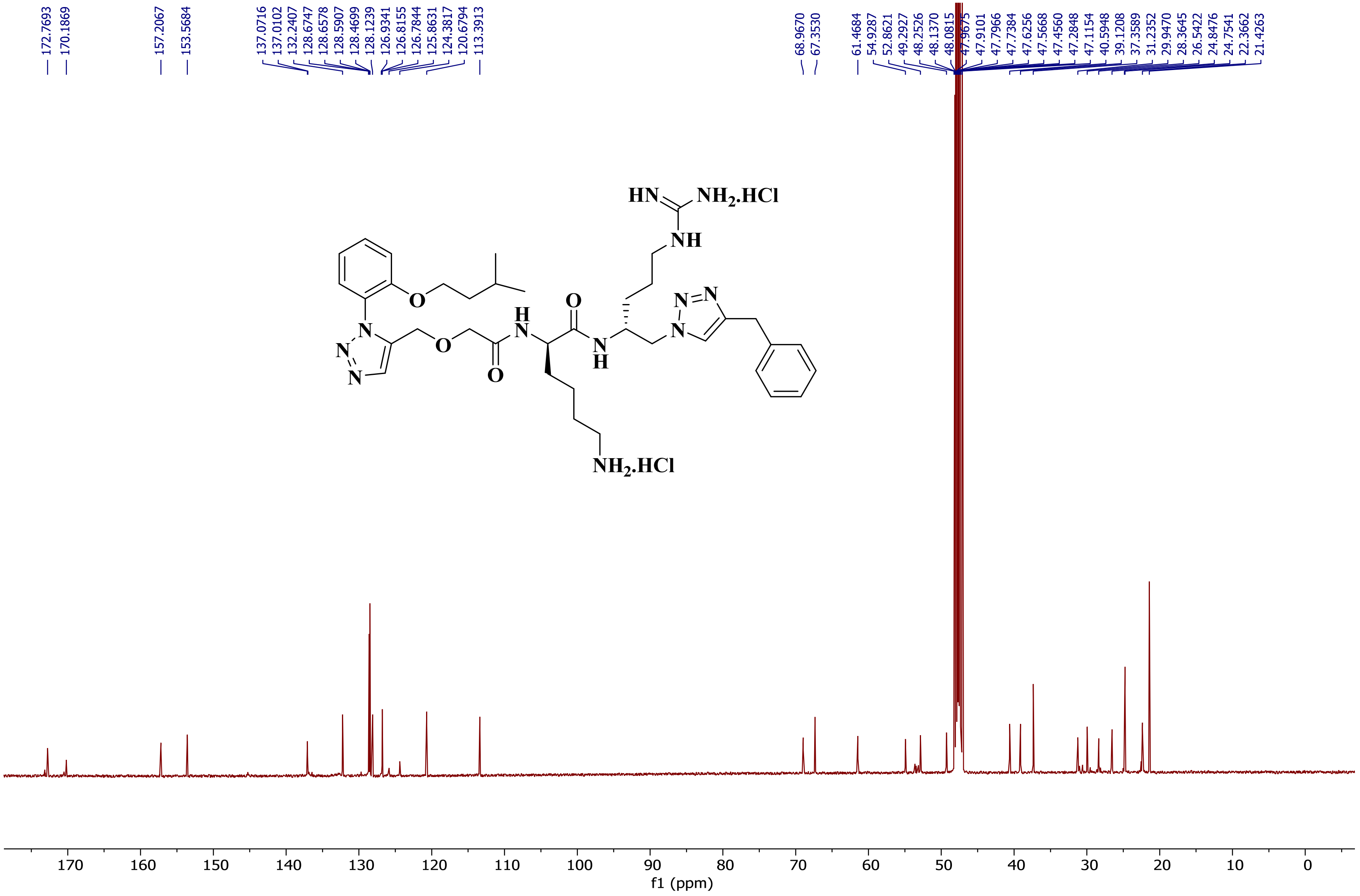

Figure S85: ^{13}C NMR of compound 50 (101 MHz, CD_3OD). Rotamers apparent in spectrum as evidenced by doubling of resonances.

Table S1. Secondary antimicrobial screening[a] – (bacteria and fungi)

Entry	Compound	*S. aureus* ATCC 43300[b]	*P. aeruginosa* ATCC 27853	*K. pneumoniae* ATCC 700603[c]	*A. baumannii* ATCC 19606	*E. coli* ATCC 25922	*C. albicans* ATCC 90028	*C. neoformans var. grubii* ATCC 208821	CC_{50}[d]	HC_{50}[e]
1	**21**	16	>32	>32	>32	>32	>32	32	>32	>32
2	**10**	32	>32	>32	>32	>32	>32	32	>32	>32
3	**11**	32	>32	>32	>32	>32	>32	32	>32	>32
4	**12**	32	>32	>32	>32	>32	>32	32	>32	>32
5	**13**	32	>32	>32	>32	>32	>32	32	>32	>32
6	**14**	32	>32	>32	>32	>32	>32	32	>32	>32
7	**15**	32	>32	>32	>32	>32	>32	32	>32	>32
8	**16**	32	>32	>32	>32	>32	>32	32	>32	>32
9	**21**	8	>32	>32	>32	32	32	8	>32	>32
10	**22**	4	>32	>32	>32	32	16	32	>32	>32
11	**23**	8	>32	>32	>32	32	32	16	>32	>32
12	**24**	8	32	>32	32	32	32	>32	21.9	>32
13	**25**	4	>32	>32	32	32	16	8	>32	10.6
14	**26**	16	>32	>32	>32	>32	32	>32	23.5	>32
15	**46**	16	>32	>32	>32	>32	32	>32	>32	>32
16	**47**	32	>32	>32	>32	>32	32	>32	>32	>32
17	**36**	16	>32	>32	>32	>32	16	32	>32	>32
18	**37**	16	>32	>32	>32	>32	16	16	>32	>32
19	**38**	32	>32	>32	>32	>32	>32	16	32	32
20	**39**	32	>32	>32	>32	>32	16	16	>32	>32
21	**40**	8	32	>32	32	32	>32	4	32	32
22	**41**	4	16	32	32	32	32	4	16	32
23	**42**	8	32	>32	32	32	>32	8	32	32
24	**43**	8	32	>32	32	32	>32	8	>32	>32
25	**44**	8	32	>32	32	32	32	8	>32	>32
26	**45**	16	32	>32	>32	32	>32	>32	>32	>32
27	**48**	>32	>32	>32	>32	>32	>32	>32	>32	24.5
28	**49**	32	>32	>32	>32	>32	>32	>32	>32	17.1
29	**50**	32	>32	>32	>32	>32	>32	>32	>32	19.8
30	vanomycin	1								
31	colistin		0.25	0.25	0.25	0.125				
32	fluconazole						0.125	0.25		

33	tamoxi-fen	13.1

[a] Testing performed by the Community for Open Antimicrobial Drug Discovery (CO-ADD) – values are reported as MIC values in µg/mL. [b] Methicillin resistant *S. aureus* (MRSA). [c] Muti-drug resistant *K. pneumonia*. [d] Cytotoxicity; determined on HEK293 cells. [e] Haemolysis; Determined by lysis of sheep erythrocytes.

General methodology

The murine model of CDI was conducted with project collaborators at Monash University. The murine model of CDI was performed by pretreating cohorts of five mice that had been earlier infected *via* oral gavage with hypervirulent M7404 *C. difficile* spores. The infection was allowed to develop for 12 h before the trial drug was administered every 12 h for five days. The mice received a single dosage of 2.5 mg of compound **41** (i.e. 100 mg/kg for an average 25 g mouse) administered in a solvent mixture of 10% DMSO and H_2O at each 12 h dosing period. The mice body weight was checked every day. If a mouse lost 10% of its body weight in the first 24 h, then the infection was developing, and the mouse was separated from the trial and culled for ethical reasons. The whole survival rate for every drug cohort was utilized to measure effectiveness of that drug. Other physiological parameters such as cage appearance scores and faecal consistency scores were additional measures for the efficacy of the drug in this murine trial. The experimental data of the *in vivo* CDI model can be found in Section 3.3.3 and the experimental procedures can be found in Section 6.4.3.

Preliminary trials

Compound **40** was selected for a murine model of CDI study because of its better antimicrobial potency against *C. difficile* and its better water solubility profile (Section 3.2.7). Compound **40** was prepared on a larger scale i.e. nearly 300 mg for the purpose of *in vivo* mouse model of CDI at Monash University. In the first trial, attempts were made to administer compound **40** through drinking water, but the mice refused to drink the adulterated water. As a result, the mice were dehydrated and lost weight due the effects of the infection.

Secondary trial

In the secondary CDI mouse model trial, the solution (10% v/v DMSO/H_2O) of the compound **40** was administered to mice by oral gavage.
